# Supplementary material for: Drug repurposing for aging research using model organisms
Source: Aging Cell. 2017 Jun 16;16(5):1006–15. doi: 10.1111/acel.12626 (PMC5595691; doi:10.1111/acel.12626)
Supplement: Supplementary file 7 — Data S1 Zip‐Archive of all report cards. [file ACEL-16-1006-s007.zip › RC_GOL.pdf]

GOL

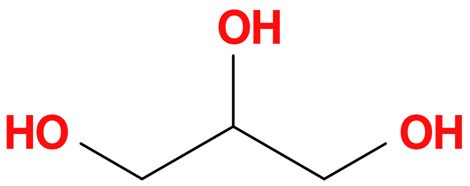

Database identifiers

ChEMBLCompound	CHEMBL692
DrugBank	DB04077
CHEBI	17522
CHEBI	17754
ZINC	ZINC00895048
eMolecules	476493

Ranking

	Rank	Score
Drosophila	372/697	0.399
C. elegans	472/591	0.098

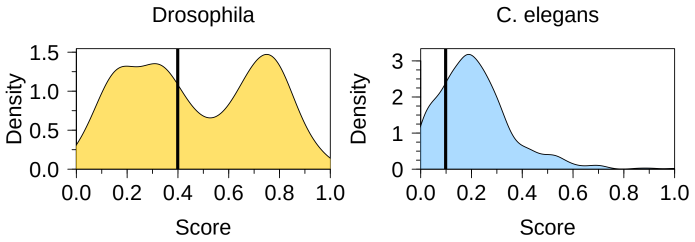

	Ageing implication	Domain conservation	Binding site conservation	Binding affinity	Bioavailability	Lipinski	Promiscuity	Purchasability	Drug approval	**Total**
Drosophila	1.0	0.982	1.0	0.452	(0.9)	0.0	-0.2	0.1	0.1	0.399
C. elegans	1.0	0.982	1.0	0.452	0.222	0.0	-0.2	0.1	0.1	0.098

Names

- **glycerol**
- glycyl alcohol
- Gro
- Monoctanoin Component D
- Oelsuess
- Ophthalgan
- Optim
- Osmoglyn
- Propanetriol
- Trihydroxypropane

Roles

solvent, human metabolite, osmolyte, detergent, algal metabolite

Status

Approved drug (according to ChEMBL)	Yes
Number of Rule of 5 violations	0
Binding affinity to original target in log units (RF-Score prediction)	4.81
Burns *C. elegans* bioavailability prediction	-4.07

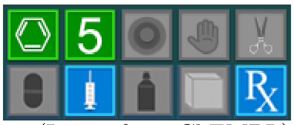

(Image from ChEMBL)

Compound Target Characteristics

tRNA (cytosine(38)-C(5))-methyltransferase

Best gene implication in ageing for this target family came from gene Q8MSZ1 annotated in UniProt release 2014_02. Annotation GO 8340 (determination of adult lifespan) was Inferred from Mutant Phenotype

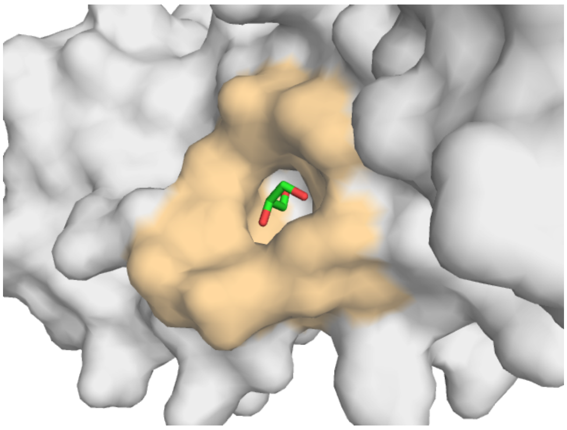

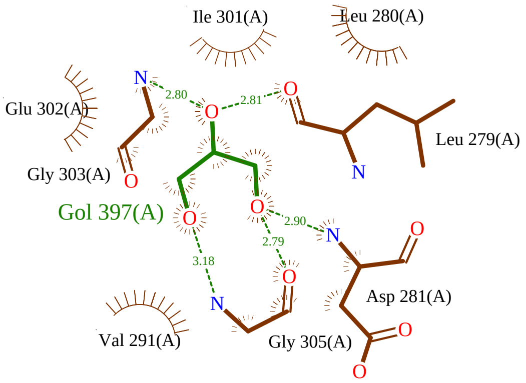

protein	amino acids contacts (binding site)
PDB:1g55:chainA:O14717	L L D V I E G T G
tr:B7Z8H2:B7Z8H2_HUMAN	L L D V I E G T G
tr:E7EMI8:E7EMI8_HUMAN	L L D V I E G T G
sp:O14717:TRDMT_HUMAN	L L D V I E G T G
tr:Q6ICS7:Q6ICS7_HUMAN	L L D V I E G T G
sp:Q4G073:TRDMT_RAT	L L D M I E G T G
tr:Q5I0V6:Q5I0V6_MOUSE	L L D M I E G T G
sp:O55055:TRDMT_MOUSE	L L D M I E G T G
tr:Q8MSZ1:Q8MSZ1_DROME	V M D M T E G T G
tr:Q9VKB3:Q9VKB3_DROME	V M D M T E G T G
tr:Q9U6H7:Q9U6H7_DROME	V M D M T E G T G

	whole protein		domain-based		contact-based	
protein	ident	simil	ident	simil	ident	simil
PDB:1g55:chainA:O14717	0.99	0.99	0.99	0.99	1.0	1.0
tr:B7Z8H2:B7Z8H2_HUMAN	0.61	0.63	0.87	0.92	1.0	1.0
tr:E7EMI8:E7EMI8_HUMAN	0.74	0.78	1.0	1.0	1.0	1.0
sp:O14717:TRDMT_HUMAN	1.0	1.0	1.0	1.0	1.0	1.0
tr:Q6ICS7:Q6ICS7_HUMAN	1.0	1.0	1.0	1.0	1.0	1.0
sp:Q4G073:TRDMT_RAT	0.81	0.94	0.83	0.94	0.89	0.98
tr:Q5I0V6:Q5I0V6_MOUSE	0.76	0.88	0.82	0.94	0.89	0.98
sp:O55055:TRDMT_MOUSE	0.76	0.88	0.82	0.94	0.89	0.98
tr:Q8MSZ1:Q8MSZ1_DROME	0.35	0.65	0.32	0.61	0.56	0.82
tr:Q9VKB3:Q9VKB3_DROME	0.37	0.68	0.32	0.61	0.56	0.82
tr:Q9U6H7:Q9U6H7_DROME	0.35	0.67	0.32	0.61	0.56	0.82

Mt2 (FBgn0028707) associated phenotypes

chemical resistant, chemical sensitive, conditional, dominant, heat stress response defective, immune response defective, long lived, melanotic mass phenotype, non-suppressor of variegation, short lived,

stress response defective, suppressor of variegation

(Information from FlyBase)

Serine/threonine-protein kinase Chk1

Best gene implication in ageing for this target family came from gene O14757 annotated in UniProt release 2014_02. Annotation GO subterm of 7568 (aging) was Non-traceable Author Statement

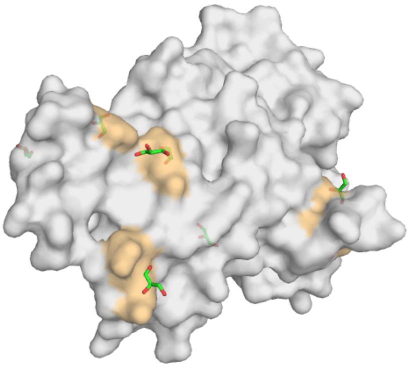

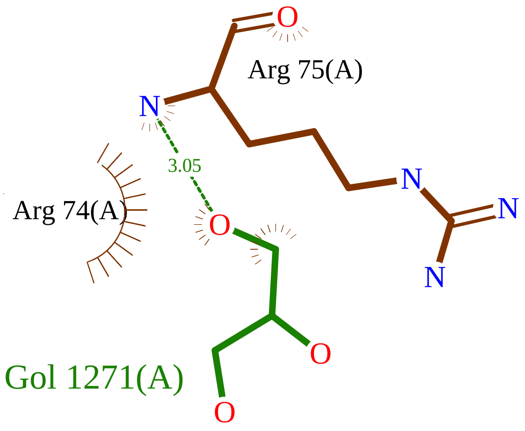

protein	amino acids contacts (binding site)
PDB:2x8i:chainA:O14757	D V V R E E Y R R F D I P P Q R H Q K E F R E T Y E A G E L D Q S S C W K K H
PDB:2ydk:chainA:O14757	D V V R E E Y R R F D I P P Q R H Q K E F R E T Y E A G E L D Q S S C W K K H
PDB:2yex:chainA:O14757	D V V R E E Y R R F D I P P Q R H Q K E F R E T Y E A G E L D Q S S C W K K H
PDB:3ot8:chainA:O14757	D V V R E E Y R R F D I P P Q R H Q K E F R E T Y E A G E L D Q S S C W K K H
PDB:3pa4:chainA:O14757	D V V R E E Y R R F D I P P Q R H Q K E F R E T Y E A G E L D Q S S C W K K H
PDB:3pa5:chainA:O14757	D V V R E E Y R - F D I P P Q R H Q K E F R E T Y E A G E L D Q S S C W K K H
PDB:4fsm:chainA:O14757	D V V R E E Y R R F D I P P Q R H Q K E F R E T Y E A G E L D Q S S C W K K H
PDB:4fst:chainA:O14757	D V V R E E Y R R F D I P P Q R H Q K E F R E T Y E A G E L D Q S S C W K K H
PDB:4fsu:chainA:O14757	D V V R E E Y R R F D I P P Q R H Q K E F R E T Y E A G E L D Q S S C W K K H
PDB:4fsy:chainA:O14757	D V V R E E Y R R F D I P P Q R H Q K E F R E T Y E A G E L D Q S S C W K K H
PDB:4ft3:chainA:O14757	D V V R E E Y R R F D I P P Q R H Q K E F R E T Y E A G E L D Q S S X W K K H
PDB:4ft5:chainA:O14757	D V V R E E Y R R F D I P P Q R H Q K E F R E T Y E A G E L D Q S S C W K K H
PDB:4ft7:chainA:O14757	D V V R E E Y R R F D I P P Q R H Q K E F R E T Y E A G E L D Q S S C W K K H
PDB:4fta:chainA:O14757	D V V R E E Y R R F D I P P Q R H Q K E F R E T Y E A G E L D Q S S C W K K H
PDB:4ftc:chainA:O14757	D V V R E E Y R R F D I P P Q R H Q K E F R E T Y E A G E L D Q S S C W K K H
PDB:4fti:chainA:O14757	D V V R E E Y R R F D I P P Q R H Q K E F R E T Y E A G E L D Q S S C W K K H
PDB:4ftm:chainA:O14757	D V V R E E Y R R F D I P P Q R H Q K E F R E T Y E A G E L D Q S S C W K K H
PDB:4ftn:chainA:O14757	D V V R E E Y R R F D I P P Q R H Q K E F R E T Y E A G E L D Q S S C W K K H
PDB:4fto:chainA:O14757	D V V R E E Y R - F D I P P Q R H Q K E F R E T Y E A G E L D Q S S C W K K H
PDB:4ftq:chainA:O14757	D V V R E E Y R R F D I P P Q R H Q K E F R E T Y E A G E L D Q S S C W K K H
PDB:4ftr:chainA:O14757	D V V R E E Y R R F D I P P Q R H Q K E F R E T Y E A G E L D Q S S C W K K H
PDB:4ftu:chainA:O14757	D V V R E E Y R R F D I P P Q R H Q K E F R E T Y E A G E L D Q S S C W K K H
PDB:4gh2:chainA:O14757	D V V R E E Y R R F D I P P Q R H Q K E F R E T Y E A G E L D Q S S X W K K H
PDB:4hyi:chainA:O14757	D V V R E E Y R R F D I P P Q R H Q K E F R E T Y E A G E L D Q S S C W K K H
tr:J3KN87:J3KN87_HUMAN	D V V R E E Y R R F D I P P Q R H Q K E F R E T Y E A G E L D Q S S C W K K H
sp:O14757:CHK1_HUMAN	D V V R E E Y R R F D I P P Q R H Q K E F R E T Y E A G E L D Q S S C W K K H
sp:Q91ZN7:CHK1_RAT	D V V R E E Y R R F D I P Q Q R H Q K E F R E T Y E A G E L D Q S S C W K K H
tr:G3UYB1:G3UYB1_MOUSE	D V V R E E Y R R F D I P Q Q R H Q K E F R E T Y E A G E L D Q S S C W K K H
tr:G3UYC5:G3UYC5_MOUSE	D V V R E E Y R R F D I P Q Q R H Q K E F R E T Y E A G E L D Q S S C W K K H
sp:O35280:CHK1_MOUSE	D V V R E E Y R R F D I P Q Q R H Q K E F R E T Y E A G E L D Q S S C W K K H
sp:O61661:CHK1_DROME	T A I R G E F R S F D I P H Q R T Q K E F R E T Y Q T G E L D Q S N C W R N R
sp:Q9N3Z3:CHK1_CAEEL	R V V T P E I R N F D I P V Q F K Q K E Y R E T Y P A G E L D R S A S W I N R
sp:P38147:CHK1_YEAST	V G H Q P S I N V F D I P D Q F Q Q K E F R L S Y D V G Q T E L S E N F I N R

protein	whole protein ident	simil	domain-based ident	simil	contact-based ident	simil
PDB:2x8i:chainA:O14757	1.0	1.0	1.0	1.0	0.98	0.96
PDB:2ydk:chainA:O14757	1.0	1.0	1.0	1.0	0.98	0.96
PDB:2yex:chainA:O14757	1.0	1.0	1.0	1.0	0.98	0.96
PDB:3ot8:chainA:O14757	1.0	1.0	1.0	1.0	1.0	1.0
PDB:3pa4:chainA:O14757	1.0	1.0	1.0	1.0	1.0	1.0
PDB:3pa5:chainA:O14757	1.0	1.0	1.0	1.0	0.98	0.96
PDB:4fsm:chainA:O14757	1.0	1.0	1.0	1.0	1.0	1.0
PDB:4fst:chainA:O14757	1.0	1.0	1.0	1.0	0.98	0.96
PDB:4fsu:chainA:O14757	0.99	1.0	0.99	1.0	1.0	1.0
PDB:4fsy:chainA:O14757	0.99	0.99	0.99	0.99	1.0	1.0
PDB:4ft3:chainA:O14757	0.99	0.99	0.99	0.99	0.98	0.97
PDB:4ft5:chainA:O14757	1.0	1.0	1.0	1.0	1.0	1.0
PDB:4ft7:chainA:O14757	1.0	1.0	1.0	1.0	1.0	1.0
PDB:4fta:chainA:O14757	1.0	1.0	1.0	1.0	1.0	1.0
PDB:4ftc:chainA:O14757	1.0	1.0	1.0	1.0	1.0	1.0
PDB:4fti:chainA:O14757	1.0	1.0	1.0	1.0	1.0	1.0
PDB:4ftm:chainA:O14757	1.0	1.0	1.0	1.0	1.0	1.0
PDB:4ftn:chainA:O14757	1.0	1.0	1.0	1.0	1.0	1.0
PDB:4fto:chainA:O14757	1.0	1.0	1.0	1.0	0.98	0.96
PDB:4ftq:chainA:O14757	1.0	1.0	1.0	1.0	0.98	0.96
PDB:4ftr:chainA:O14757	1.0	1.0	1.0	1.0	1.0	1.0
PDB:4ftu:chainA:O14757	1.0	1.0	1.0	1.0	1.0	1.0
PDB:4gh2:chainA:O14757	0.99	0.99	0.99	0.99	0.98	0.97
PDB:4hyi:chainA:O14757	1.0	1.0	1.0	1.0	1.0	1.0
tr:J3KN87:J3KN87_HUMAN	0.91	0.91	1.0	1.0	1.0	1.0
sp:O14757:CHK1_HUMAN	1.0	1.0	1.0	1.0	1.0	1.0
sp:Q91ZN7:CHK1_RAT	0.94	0.98	0.96	0.99	0.96	0.98
tr:G3UYB1:G3UYB1_MOUSE	0.84	0.89	0.95	0.99	0.96	0.98
tr:G3UYC5:G3UYC5_MOUSE	0.87	0.91	0.95	0.99	0.96	0.98
sp:O35280:CHK1_MOUSE	0.93	0.98	0.95	0.99	0.96	0.98
sp:O61661:CHK1_DROME	0.44	0.75	0.49	0.8	0.57	0.75
sp:Q9N3Z3:CHK1_CAEEL	0.31	0.62	0.41	0.69	0.5	0.69
sp:P38147:CHK1_YEAST	0.23	0.62	0.3	0.68	0.3	0.54

grp (FBgn0261278) associated phenotypes

cell cycle defective, chemical sensitive, decreased cell death, maternal effect, mitotic cell cycle defective, non-rescuable maternal effect, partially lethal - majority die, radiation resistant, radiation sensitive

(Information from FlyBase)

grp (UniProt:O61661) annotation

Function: Serine/threonine-protein kinase which is required for checkpoint-mediated cell cycle arrest and activation of DNA repair in response to the presence of DNA damage or unreplicated DNA. May also negatively regulate cell cycle progression during unperturbed cell cycles. May phosphorylate the CDC25 phosphatase stg, which promotes its degradation. This results in increased inhibitory tyrosine phosphorylation of CDC2-cyclin complexes and consequent inhibition of cell cycle progression. (PubMed:10209095, PubMed:10469601, PubMed:10980701, PubMed:12919679, PubMed:14711410, PubMed:15723794, PubMed:15860729, PubMed:16079276, PubMed:7925016, PubMed:9197245, PubMed:9214509).

Subcellular location: Nucleus ECO:0000269—PubMed:10980701, PubMed:16079276).

Developmental stage: Expressed both maternally and zygotically. Maternally supplied mRNA is degraded during progression from nuclear stage 12 to nuclear stage 13. Zygotic expression is seen at reduced levels later in embryogenesis and during larval development. Higher expression is seen in pupae, coincident with ovarian differentiation. May be activated during the syncytial blastoderm divisions which precede cellularization, the Drosophila equivalent of the midblastula transition (MBT). Developmentally regulated activation of the DNA replication checkpoint may occur as the nucleo-cytoplasmic ratio increases and maternal replication factors are depleted. Elongation of the embryonic cell cycle may allow time for the transcription of genes that initiate the switch from

maternal to zygotic control of embryogenesis. (PubMed:9197245, PubMed:9214509).
Ptm: Phosphorylated in a MEI-41/ATR dependent manner in response to DNA damage or the presence of unreplicated DNA. (PubMed:15860729).

(Information from UniProt)

chk-1 (WBGene00000498) associated phenotypes
lethal, locomotion variant, mid larval lethal, sterile

(Information from WormBase)

chk-1 (UniProt:Q9N3Z3) annotation
Function: Serine/threonine-protein kinase which is required for checkpoint-mediated cell cycle arrest and activation of DNA repair in response to the presence of DNA damage or unreplicated DNA. May also negatively regulate cell cycle progression during unperturbed cell cycles. Required for checkpoint mediated cell cycle arrest in response to DNA damage in germline cells. Essential for embryogenesis. (PubMed:15326393).
Subcellular location: Cytoplasm Nucleus
Tissue specificity: Expressed in the germline. (PubMed:15326393).
Developmental stage: Highly expressed in the embryo. (PubMed:15326393).

(Information from UniProt)

Histone deacetylase 3

Best gene implication in ageing for this target family came from gene P32561 annotated in UniProt release 2014_02. Annotation GO subterm of 7568 (aging) was Inferred from Mutant Phenotype

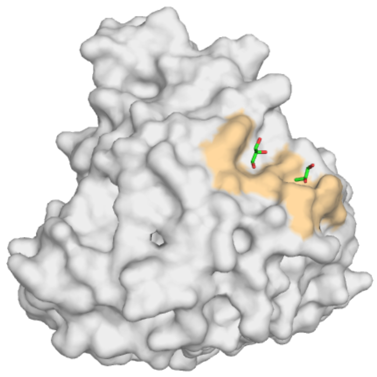

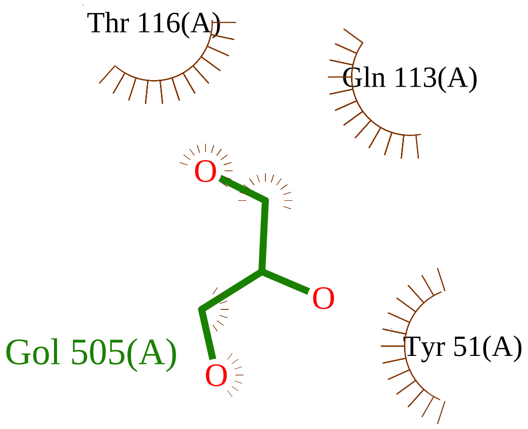

protein	amino acids contacts (binding site)
PDB:4a69:chainA:O15379	Y Q T N N E Y R
sp:O15379:HDAC3_HUMAN	Y Q T N N E Y R
tr:D4AEB0:D4AEB0_RAT	Y Q T N N E Y R
sp:O88895:HDAC3_MOUSE	Y Q T N N E Y R
tr:Q3UM33:Q3UM33_MOUSE	Y Q T N N E Y R
tr:Q7KTS4:Q7KTS4_DROME	Y E Q N H E Y R
tr:Q9VNC2:Q9VNC2_DROME	Y E Q N H E Y R
sp:P32561:RPD3_YEAST	K E A N R E Y R

protein	whole protein ident	simil	domain-based ident	simil	contact-based ident	simil
PDB:4a69:chainA:O15379	1.0	1.0	1.0	1.0	1.0	1.0
sp:O15379:HDAC3_HUMAN	1.0	1.0	1.0	1.0	1.0	1.0
tr:D4AEB0:D4AEB0_RAT	0.91	0.96	0.9	0.96	1.0	1.0
sp:O88895:HDAC3_MOUSE	0.99	0.99	1.0	1.0	1.0	1.0
tr:Q3UM33:Q3UM33_MOUSE	1.0	1.0	1.0	1.0	1.0	1.0
tr:Q7KTS4:Q7KTS4_DROME	0.65	0.88	0.7	0.91	0.63	0.84
tr:Q9VNC2:Q9VNC2_DROME	0.65	0.88	0.7	0.91	0.63	0.84
sp:P32561:RPD3_YEAST	0.52	0.79	0.6	0.88	0.5	0.7

Hdac3 (FBgn0025825) associated phenotypes
decreased cell growth, dominant, increased cell death, large body, lethal - all die before end of pupal stage, lethal - all die during larval stage, neuroanatomy defective, partially lethal, small body, somatic clone, some die during pupal stage

(Information from FlyBase)

Hdac3 (UniProt:Q7KTS4) annotation
Subcellular location: Nucleus ECO:0000256—PIRNR:PIRNR037913.

(Information from UniProt)

Hdac3 (UniProt:Q9VNC2) annotation
Subcellular location: Nucleus ECO:0000256—PIRNR:PIRNR037913.

(Information from UniProt)

3-phosphoinositide-dependent protein kinase 1

Best gene implication in ageing for this target family came from gene Q9Y1J3 via mapping the annotation from Ensembl WBGene00003965 via mapping the annotation from EntrezGene 180475 via mapping the annotation from GenAgeModels 0499 annotated in GenAge release 17.

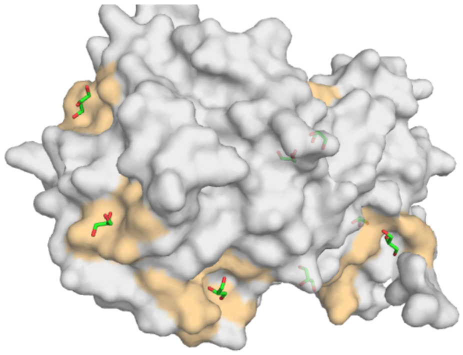

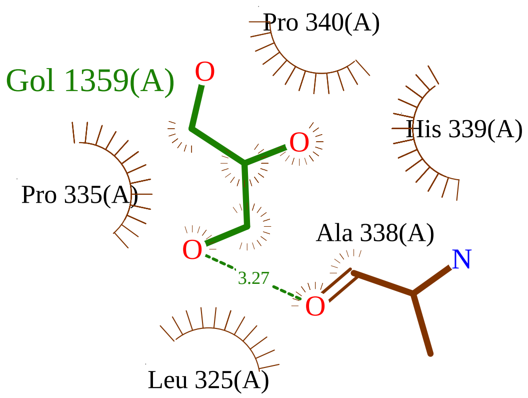

protein	amino acids contacts (binding site)
PDB:1h1w:chainA:O15530	P K P F K F G E G S F S V L R A T S R E Y K I L Y V E R H Y F T F Q K L Y F L
PDB:1oky:chainA:O15530	P K P F K F G E G S F S V L R A T S R E Y K I L Y V E R H Y F T F Q K L Y F L
PDB:1okz:chainA:O15530	P K P F K F G E G S F S V L R A T S R E Y K I L Y V E R H Y F T F Q K L Y F L
PDB:1uu3:chainA:O15530	P K P F K F G E G S F S V L R A T S R E Y K I L Y V E R H Y F T F Q K L Y F L
PDB:1uu7:chainA:O15530	P K P F K F G E G S F S V L R A T S R E Y K I L Y V E R H Y F T F Q K L Y F L
PDB:1uu8:chainA:O15530	P K P F K F G E G S F S V L R A T S R E Y K I L Y V E R H Y F T F Q K L Y F L
PDB:1uu9:chainA:O15530	P K P F K F G E G S F S V L R A T S R E Y K I L Y V E R H Y F T F Q K L Y F L
PDB:1uvr:chainA:O15530	P K P F K F G E G S F S V L R A T S R E Y K I L Y V E R H Y F T F Q K L Y F L
PDB:1w1d:chainA:O15530	- -
PDB:1w1h:chainA:O15530	- -
PDB:1w1h:chainC:O15530	- -
PDB:1z5m:chainA:O15530	P K P F K F G E G S F S V L R A T S R E Y K I L Y V E R H Y F T F Q K L Y F L
PDB:2biy:chainA:O15530	P K P F K F G E G S F S V L R A T S R E Y K I L Y V E R H Y F T F Q K L Y F L
PDB:2pe0:chainA:O15530	P K P F K F G E G S F S V L R A T S R E Y K I L Y V E R H Y F T F Q K L Y F L
PDB:2pe1:chainA:O15530	P K P F K F G E G S F S V L R A T S R E Y K I L Y V E R H Y F T F Q K L Y F L
PDB:2pe2:chainA:O15530	P K P F K F G E G S F S V L R A T S R E Y K I L Y V E R H Y F T F Q K L Y F L
PDB:2vki:chainA:O15530	- -
PDB:2xch:chainA:O15530	P K P F K F G E G S F S V L R A T S R E Y K I L Y V E R H Y F T F Q K L Y F L
PDB:2xck:chainA:O15530	- K P F K F G E G S F S T L R A T S R E Y K I L Y V E R H Y F T F Q K L Y F L
PDB:3nun:chainA:O15530	P K P F K F G E G S F S V L R A T S R E Y K I L Y V E R H Y F T F Q K L Y F L
PDB:3nus:chainA:O15530	P K P F K F G E G S F S V L R A T S R E Y K I L Y V E R H Y F T F Q K L Y F L
PDB:3nuu:chainA:O15530	P K P F K F G E G S F S V L R A T S R E Y K I L Y V E R H Y F T F Q K L Y F L
PDB:3nuy:chainA:O15530	P K P F K F G E G S F S V L R A T S R E Y K I L Y V E R H Y F T F Q K L Y F L
PDB:3qcq:chainA:O15530	P K P F K F G E G S F S V L R A T S R E Y K I L Y V E R H Y F T F Q K L Y F L
PDB:3qcs:chainA:O15530	P K P F K F G E G S F S V L R A T S R E Y K I L Y V E R H Y F T F Q K L Y F L
PDB:3qcx:chainA:O15530	P K P F K F G E G S F S V L R A T S R E Y K I L Y V E R H Y F T F Q K L Y F L
PDB:3qcy:chainA:O15530	P K P F K F G E G S F S V L R A T S R E Y K I L Y V E R H Y F T F Q K L Y F L
PDB:3qd0:chainA:O15530	P K P F K F G E G S F S V L R A T S R E Y K I L Y V E R H Y F T F Q K L Y F L
PDB:3qd3:chainA:O15530	P K P F K F G E G S F S V L R A T S R E Y K I L Y V E R H Y F T F Q K L Y F L
PDB:3rwp:chainA:O15530	P K P F K F G E G S F S V L R A T S R E Y K I L Y V E R H Y F T F Q K L Y F L
PDB:3rwq:chainA:O15530	P K P F K F G E G S F S V L R A T S R E Y K I L Y V E R H Y F T F Q K L Y F L
PDB:3sc1:chainA:O15530	P K P F K F G E G S F S V L R A T S R E Y K I L Y V E R H Y F T F Q K L Y F L
tr:E9PER6:E9PER6_HUMAN	P K P F K F G E G S F S V L R A T S R E Y K I L Y V E R H Y F T F Q K L Y F L
tr:C9JWR9:C9JWR9_HUMAN	P K P F K F G E G S F S V L R A T S R E Y K I L Y V E R H Y F T F Q K L Y F L
sp:O15530:PDPK1_HUMAN	P K P F K F G E G S F S V L R A T S R E Y K I L Y V E R H Y F T F Q K L Y F L
sp:O55173:PDPK1_RAT	P K P F K F G E G S F S V L R A T S R E Y K I L Y V E R H Y F T F Q K L Y F L
tr:F2Z3X6:F2Z3X6_MOUSE	P K P F K F G E G S F S V L R A T S R E Y K I L Y V E R H Y F T F Q K L Y F L
sp:Q9Z2A0:PDPK1_MOUSE	P K P F K F G E G S F S V L R A T S R E Y K I L Y V E R H Y F T F Q K L Y F L
tr:Q810Z4:Q810Z4_MOUSE	P K P F K F G E G S F S V L R A T S R E Y K I L Y V E R H Y F T F Q K L Y F L
tr:A4V164:A4V164_DROME	A R P F I F G E G S Y S V L V H S R R E Y K V C Y I E R N S C T F Q S L Y F M
tr:C7LAC8:C7LAC8_DROME	A R P F I F G E G S Y S V L V H S R R E Y K V C Y I E R N S C T F Q S L Y F M
tr:H5V8B9:H5V8B9_DROME	A R P F I F G E G S Y S V L V H S R R E Y K V C Y I E R N S C T F Q S L Y F M
sp:Q9W0V1:PDPK1_DROME	A R P F I F G E G S Y S V L V H S R R E Y K V C Y I E R N S C T F Q S L Y F M
sp:Q9Y1J3:PDPK1_CAEEL	C K S F M F G E G A Y S V R R A T D A M F K V L A I E K Q Y T H F H R I Y F I
sp:Q03306:PKH3_YEAST	S K P F I F G H G S Y S V K L K S P N K Y K V C Y V E K Q Y Y T F H N L Y F L

protein	whole protein ident	simil	domain-based ident	simil	contact-based ident	simil
PDB:1h1w:chainA:O15530	1.0	1.0	1.0	1.0	0.91	0.81
PDB:1oky:chainA:O15530	1.0	1.0	1.0	1.0	0.91	0.81
PDB:1okz:chainA:O15530	1.0	1.0	1.0	1.0	0.91	0.81
PDB:1uu3:chainA:O15530	1.0	1.0	1.0	1.0	0.91	0.81
PDB:1uu7:chainA:O15530	1.0	1.0	1.0	1.0	0.91	0.81
PDB:1uu8:chainA:O15530	1.0	1.0	1.0	1.0	0.91	0.81
PDB:1uu9:chainA:O15530	1.0	1.0	1.0	1.0	0.91	0.81
PDB:1uvr:chainA:O15530	1.0	1.0	1.0	1.0	0.91	0.81
PDB:1w1d:chainA:O15530	0.99	0.99	1.0	1.0	0.09	0.0
PDB:1w1h:chainA:O15530	1.0	1.0	1.0	1.0	0.09	0.0
PDB:1w1h:chainC:O15530	1.0	1.0	1.0	1.0	0.09	0.0
PDB:1z5m:chainA:O15530	1.0	1.0	1.0	1.0	0.91	0.81
PDB:2biy:chainA:O15530	1.0	1.0	1.0	1.0	0.91	0.81
PDB:2pe0:chainA:O15530	1.0	1.0	1.0	1.0	0.91	0.81
PDB:2pe1:chainA:O15530	1.0	1.0	1.0	1.0	0.91	0.81
PDB:2pe2:chainA:O15530	1.0	1.0	1.0	1.0	0.91	0.81
PDB:2vki:chainA:O15530	0.99	1.0	0.99	1.0	0.09	0.0
PDB:2xch:chainA:O15530	1.0	1.0	1.0	1.0	0.91	0.81
PDB:2xck:chainA:O15530	1.0	1.0	1.0	1.0	0.88	0.78
PDB:3nun:chainA:O15530	1.0	1.0	1.0	1.0	0.91	0.81
PDB:3nus:chainA:O15530	1.0	1.0	1.0	1.0	0.91	0.81
PDB:3nuu:chainA:O15530	1.0	1.0	1.0	1.0	0.91	0.81
PDB:3nuy:chainA:O15530	1.0	1.0	1.0	1.0	0.91	0.81
PDB:3qcq:chainA:O15530	1.0	1.0	1.0	1.0	0.91	0.81
PDB:3qcs:chainA:O15530	1.0	1.0	1.0	1.0	0.91	0.81
PDB:3qcx:chainA:O15530	1.0	1.0	1.0	1.0	0.91	0.81
PDB:3qcy:chainA:O15530	1.0	1.0	1.0	1.0	0.91	0.81
PDB:3qd0:chainA:O15530	1.0	1.0	1.0	1.0	0.91	0.81
PDB:3qd3:chainA:O15530	1.0	1.0	1.0	1.0	0.91	0.81
PDB:3rwp:chainA:O15530	1.0	1.0	1.0	1.0	0.91	0.81
PDB:3rwq:chainA:O15530	1.0	1.0	1.0	1.0	0.91	0.81
PDB:3sc1:chainA:O15530	1.0	1.0	1.0	1.0	0.91	0.81
tr:E9PER6:E9PER6_HUMAN	0.95	0.95	1.0	1.0	1.0	1.0
tr:C9JWR9:C9JWR9_HUMAN	0.95	0.96	0.95	0.95	1.0	1.0
sp:O15530:PDPK1_HUMAN	1.0	1.0	1.0	1.0	1.0	1.0
sp:O55173:PDPK1_RAT	0.95	0.98	0.97	0.99	0.99	1.0
tr:F2Z3X6:F2Z3X6_MOUSE	0.9	0.93	0.97	0.99	0.98	0.99
sp:Q9Z2A0:PDPK1_MOUSE	0.95	0.98	0.97	0.99	0.98	0.99
tr:Q810Z4:Q810Z4_MOUSE	0.93	0.97	0.97	0.99	0.98	0.99
tr:A4V164:A4V164_DROME	0.3	0.54	0.36	0.6	0.52	0.62
tr:C7LAC8:C7LAC8_DROME	0.28	0.51	0.36	0.6	0.52	0.62
tr:H5V8B9:H5V8B9_DROME	0.28	0.51	0.36	0.6	0.52	0.62
sp:Q9W0V1:PDPK1_DROME	0.28	0.51	0.36	0.6	0.52	0.62
sp:Q9Y1J3:PDPK1_CAEEL	0.26	0.55	0.3	0.61	0.39	0.53
sp:Q03306:PKH3_YEAST	0.15	0.36	0.2	0.45	0.44	0.53

Pdk1 (FBgn0020386) associated phenotypes

cell autonomous, chemical conditional, chemical resistant, chemical sensitive, decreased cell size, developmental rate defective, increased cell death, increased cell size, male limited, neuroanatomy defective, nutrition conditional, partially lethal - majority die, small body, somatic clone

(Information from FlyBase)

Pdk1 (UniProt:Q9W0V1) annotation

Function: Serine/threonine kinase required for embryonic development. Inhibits apoptosis. Acts in the insulin receptor transduction pathway which regulates cell growth and organ size, by phosphorylating and activating Akt1 and S6k. May be involved in axonal pathfinding and synaptogenesis, and in spermatogenesis. (PubMed:10336630, PubMed:11301252, PubMed:11344272, PubMed:11752451, PubMed:11862217, PubMed:15238523, PubMed:9368760).

Subcellular location: Cytoplasm

Developmental stage: Highly expressed in Malpighian tubule primordia from stage late 11 to 13. Expressed in hindgut at stage 13. After stage 14, ubiquitously expressed at low levels.

(PubMed:11301252).
Domain: The PIF-pocket is a small lobe in the catalytic domain required by the enzyme for the binding to the hydrophobic motif of its substrates. It is an allosteric regulatory site that can accommodate small compounds acting as allosteric inhibitors. (UniProtKB:O15530).
Disruption phenotype: Death during the second instar stage. (PubMed:11752451).

(Information from UniProt)

pdk-1 (WBGene00003965) associated phenotypes

DMPP resistant, carbon dioxide avoidance variant, dauer constitutive, dauer recovery inhibited, egg laying variant, extended life span, long, reduced brood size, shortened life span, social feeding increased

(Information from WormBase)

pdk-1 (UniProt:Q9Y1J3) annotation

Function: Involved in the daf-2/insulin receptor-like transduction pathway, which controls longevity and prevents developmental arrest at the dauer stage. Phosphorylates and activates sgk-1, akt-1 and akt-2. (PubMed:10364160, PubMed:15068796).
Subunit: Interacts directly with sgk-1, akt-1 and akt-2.(PubMed:15068796).
Subcellular location: Cytoplasm
Developmental stage: Expressed in late stage embryos and throughout life. At L1, expressed in neurons, intestinal cells and hypodermal cells. In adults, expressed in the somatic gonad. (PubMed:10364160).
Domain: The PIF-pocket is a small lobe in the catalytic domain required by the enzyme for the binding to the hydrophobic motif of its substrates. It is an allosteric regulatory site that can accommodate small compounds acting as allosteric inhibitors. (UniProtKB:O15530).

(Information from UniProt)

Phosphatidylinositol 4,5-bisphosphate 3-kinase catalytic subunit delta isoform

Best gene implication in ageing for this target family came from gene Q94125 annotated in UniProt release 2014_02. Annotation GO 8340 (determination of adult lifespan) was Inferred from Mutant Phenotype

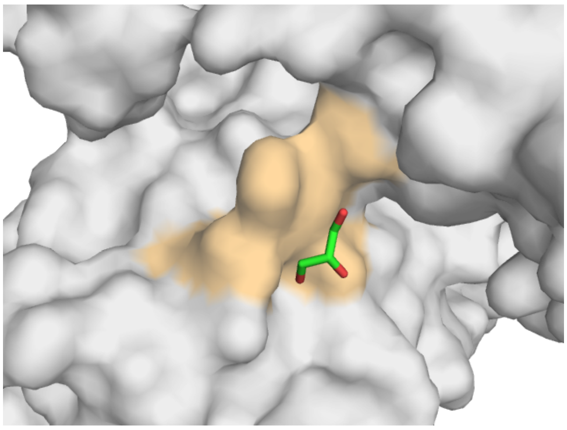

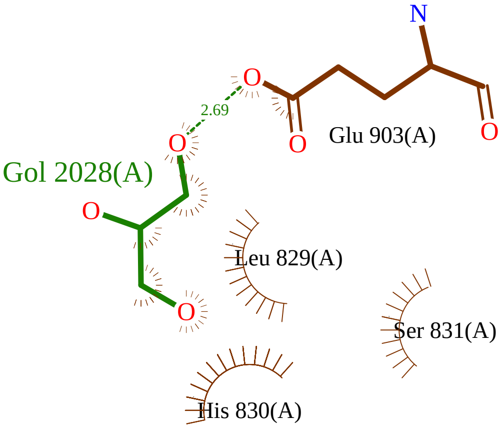

protein	amino acids contacts (binding site)			
PDB:2wxi:chainA:O35904	L	H	S	E
tr:B7ZM44:B7ZM44_HUMAN	L	R	S	E
sp:O00329:PK3CD_HUMAN	L	R	S	E
tr:Q5SR50:Q5SR50_HUMAN	L	R	S	E
tr:D4A5Q1:D4A5Q1_RAT	L	H	S	E
tr:Q3TBW3:Q3TBW3_MOUSE	L	H	S	E
tr:Q3T9Y0:Q3T9Y0_MOUSE	L	H	S	E
sp:O35904:PK3CD_MOUSE	L	H	S	E
tr:Q3UDT3:Q3UDT3_MOUSE	L	H	S	E
tr:Q8CI98:Q8CI98_MOUSE	L	H	S	E
tr:B0QZL5:B0QZL5_MOUSE	L	H	S	E
tr:P91634:P91634_DROME	R	H	A	R
tr:H1ZY88:H1ZY88_DROME	R	H	A	R
tr:H1ZY91:H1ZY91_DROME	R	H	A	R
sp:Q94125:AGE1_CAEEL	P	N	C	E

	whole protein		domain-based		contact-based	
protein	ident	simil	ident	simil	ident	simil
PDB:2wxi:chainA:O35904	1.0	1.0	1.0	1.0	1.0	1.0
tr:B7ZM44:B7ZM44_HUMAN	0.95	0.98	1.0	1.0	0.75	0.93
sp:O00329:PK3CD_HUMAN	0.95	0.98	1.0	1.0	0.75	0.93
tr:Q5SR50:Q5SR50_HUMAN	0.86	0.9	1.0	1.0	0.75	0.93
tr:D4A5Q1:D4A5Q1_RAT	0.89	0.9	0.87	0.88	1.0	1.0
tr:Q3TBW3:Q3TBW3_MOUSE	1.0	1.0	1.0	1.0	1.0	1.0
tr:Q3T9Y0:Q3T9Y0_MOUSE	1.0	1.0	1.0	1.0	1.0	1.0
sp:O35904:PK3CD_MOUSE	1.0	1.0	1.0	1.0	1.0	1.0
tr:Q3UDT3:Q3UDT3_MOUSE	1.0	1.0	1.0	1.0	1.0	1.0
tr:Q8CI98:Q8CI98_MOUSE	1.0	1.0	1.0	1.0	1.0	1.0
tr:B0QZL5:B0QZL5_MOUSE	1.0	1.0	1.0	1.0	1.0	1.0
tr:P91634:P91634_DROME	0.37	0.75	0.59	0.87	0.25	0.53
tr:H1ZY88:H1ZY88_DROME	0.37	0.75	0.59	0.87	0.25	0.53
tr:H1ZY91:H1ZY91_DROME	0.37	0.75	0.59	0.87	0.25	0.53
sp:Q94125:AGE1_CAEEL	0.23	0.62	0.39	0.72	0.25	0.51

Pi3K92E (FBgn0015279) associated phenotypes

RU486 conditional, cell autonomous, cell death defective, cell growth defective, cell non-autonomous, cell size defective, chemical resistant, chemical sensitive, decreased cell death, decreased cell growth, decreased cell number, decreased cell size, developmental rate defective, feeding behavior defective, heat sensitive, hyperplasia, increased cell growth, increased cell number, increased cell size, large body, locomotor behavior defective, long lived, neuroanatomy defective, neurophysiology defective, nutrition conditional, partially lethal - majority die, photoperiod response variant, planar polarity defective, short lived, size defective, small body, smell perception defective, somatic clone, some die during pupal stage, starvation stress response defective, stress response defective, wound healing defective

(Information from FlyBase)

age-1 (WBGene00000090) associated phenotypes

DMPP resistant, aging variant, brood size variant, cadmium resistant, dauer arrest variant, dauer constitutive, egg laying variant, extended life span, failure to hatch, fat content increased, hermaphrodite fertility variant, larval lethal, late stage egg laid, life span variant, organism physiology variant, organism temperature response variant, paraquat resistant, reproductive longevity extended, scrawny, sterile, transgene subcellular localization variant

(Information from WormBase)

age-1 (UniProt:Q94125) annotation

Function: Phosphatidylinositol 3-kinase homolog that regulates longevity and diapause. Could function in the development or neuroendocrine signaling of the dauer pathway (PubMed:8700226). Mediates susceptibility to enteropathogenic E.coli infection (PubMed:16091039). (PubMed:16091039,

PubMed:8700226).
Developmental stage: Expressed both maternally and zygotically. (PubMed:8700226).

(Information from UniProt)

Eukaryotic translation elongation factor 1 epsilon-1

Best gene implication in ageing for this target family came from gene B0LAB0 via mapping the annotation from Ensembl ENSMUSG00000001707 via mapping the annotation from EntrezGene 66143 via mapping the annotation from GenAgeModels 0816 annotated in GenAge release 17.

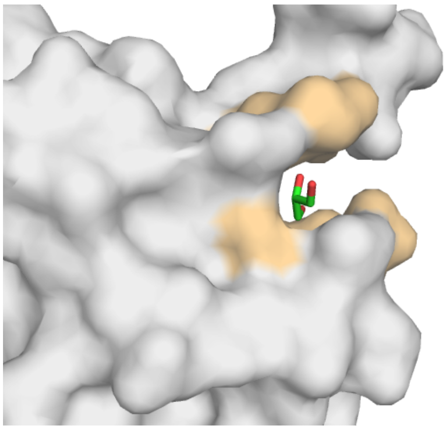

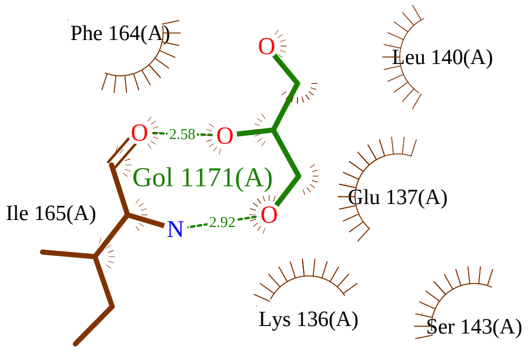

protein	amino acids contacts (binding site)
PDB:2uz8:chainA:043324	K E L S F I
sp:043324:MCA3_HUMAN	K E L S F I
tr:B2RYN3:B2RYN3_RAT	K E L S F I
tr:B0LAB0:B0LAB0_MOUSE	K E L S F I
sp:Q9D1M4:MCA3_MOUSE	K E L S F I
tr:Q8MKK1:Q8MKK1_DROME	K E L S F T
tr:Q8MTU4:Q8MTU4_DROME	K E L S F T

	whole protein		domain-based		contact-based	
protein	ident	simil	ident	simil	ident	simil
PDB:2uz8:chainA:043324	0.98	0.99	0.99	1.0	1.0	1.0
sp:043324:MCA3_HUMAN	1.0	1.0	1.0	1.0	1.0	1.0
tr:B2RYN3:B2RYN3_RAT	0.88	0.96	0.89	0.97	1.0	1.0
tr:B0LAB0:B0LAB0_MOUSE	0.29	0.3	0.48	0.5	1.0	1.0
sp:Q9D1M4:MCA3_MOUSE	0.89	0.97	0.89	0.97	1.0	1.0
tr:Q8MKK1:Q8MKK1_DROME	0.29	0.71	0.34	0.74	0.83	0.86
tr:Q8MTU4:Q8MTU4_DROME	0.27	0.64	0.34	0.74	0.83	0.86

E3 ubiquitin-protein ligase parkin

Best gene implication in ageing for this target family came from gene Q9XUS3 annotated in UniProt release 2014_02. Annotation GO 8340 (determination of adult lifespan) was Inferred from Mutant Phenotype

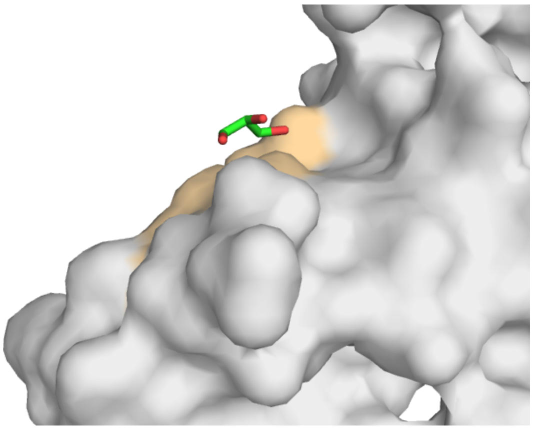

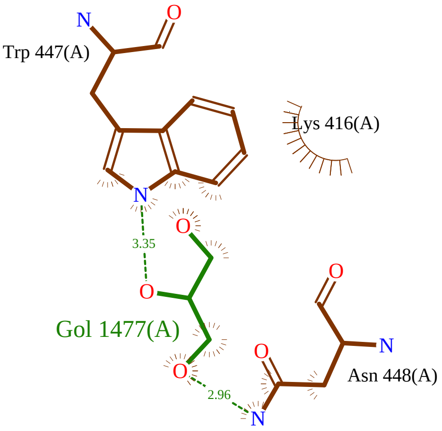

protein	amino acids contacts (binding site)
PDB:4bm9:chainA:O60260	K W N
tr:S4X465:S4X465_HUMAN	K W N
sp:O60260:PRKN2_HUMAN	K W N
tr:F1M5Q1:F1M5Q1_RAT	- - -
tr:E9PSU3:E9PSU3_RAT	- - -
tr:E9PTJ7:E9PTJ7_RAT	- - -
tr:F1M9E4:F1M9E4_RAT	- - -
tr:Q7TPF7:Q7TPF7_MOUSE	- - -
tr:Q7TPF8:Q7TPF8_MOUSE	- - -
tr:F6TNF2:F6TNF2_MOUSE	- - -
tr:F6TE01:F6TE01_MOUSE	- - -
tr:Q86LE7:Q86LE7_DROME	K W V
tr:Q7KTX7:Q7KTX7_DROME	K W V
tr:Q95TI4:Q95TI4_DROME	K W V
tr:Q9XUS3:Q9XUS3_CAEEL	R F K

	whole protein		domain-based		contact-based	
protein	ident	simil	ident	simil	ident	simil
PDB:4bm9:chainA:O60260	1.0	1.0	1.0	1.0	1.0	1.0
tr:S4X465:S4X465_HUMAN	0.77	0.77	1.0	1.0	1.0	1.0
sp:O60260:PRKN2_HUMAN	1.0	1.0	1.0	1.0	1.0	1.0
tr:F1M5Q1:F1M5Q1_RAT	0.45	0.53	0.0	0.0	0.0	0.0
tr:E9PSU3:E9PSU3_RAT	0.47	0.55	0.0	0.0	0.0	0.0
tr:E9PTJ7:E9PTJ7_RAT	0.48	0.56	0.0	0.0	0.0	0.0
tr:F1M9E4:F1M9E4_RAT	0.7	0.8	0.0	0.0	0.0	0.0
tr:Q7TPF7:Q7TPF7_MOUSE	0.06	0.08	0.0	0.0	0.0	0.0
tr:Q7TPF8:Q7TPF8_MOUSE	0.17	0.23	0.0	0.0	0.0	0.0
tr:F6TNF2:F6TNF2_MOUSE	0.41	0.49	0.0	0.0	0.0	0.0
tr:F6TE01:F6TE01_MOUSE	0.5	0.58	0.0	0.0	0.0	0.0
tr:Q86LE7:Q86LE7_DROME	0.2	0.3	0.58	0.87	0.67	0.38
tr:Q7KTX7:Q7KTX7_DROME	0.4	0.72	0.58	0.87	0.67	0.38
tr:Q95TI4:Q95TI4_DROME	0.4	0.72	0.58	0.87	0.67	0.38
tr:Q9XUS3:Q9XUS3_CAEEL	0.26	0.59	0.47	0.8	0.0	0.56

park (FBgn0041100) associated phenotypes

body size defective, chemical conditional, chemical sensitive, cold sensitive, courtship behavior defective, decreased cell death, decreased cell number, decreased cell size, developmental rate defective, drug conditional, flight defective, flightless, gravitaxis defective, hypoactive, increased cell death, jumping defective, locomotor behavior defective, long lived, male semi-sterile, neuroanatomy defective, nutrition conditional, oxidative stress response defective, partially lethal - majority die, progressive, short lived, small body, smell perception defective, some die during pupal stage, starvation stress response defective, sterile

(Information from FlyBase)

park (UniProt:Q7KTX7) annotation
Function: Functions within a multiprotein E3 ubiquitin ligase complex, catalyzing the covalent attachment of ubiquitin moieties onto substrate proteins. (PIRNR:PIRNR037880).
Pathway: Protein modification; protein ubiquitination. (PIRNR:PIRNR037880).
Subunit: Forms an E3 ubiquitin ligase complex with UBE2L3 or UBE2L6. (PIRNR:PIRNR037880).
(Information from UniProt)

park (UniProt:Q95TI4) annotation
Function: Functions within a multiprotein E3 ubiquitin ligase complex, catalyzing the covalent attachment of ubiquitin moieties onto substrate proteins. (PIRNR:PIRNR037880).
Pathway: Protein modification; protein ubiquitination. (PIRNR:PIRNR037880).
Subunit: Forms an E3 ubiquitin ligase complex with UBE2L3 or UBE2L6. (PIRNR:PIRNR037880).
(Information from UniProt)

pdr-1 (WBGene00003967) associated phenotypes
miRNA expression variant
(Information from WormBase)

Glutathione reductase, mitochondrial

Best gene implication in ageing for this target family came from gene P91938 annotated in UniProt release 2014_02. Annotation GO 8340 (determination of adult lifespan) was Inferred from Mutant Phenotype

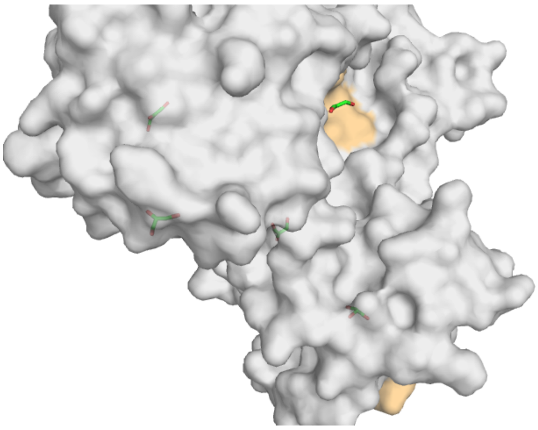

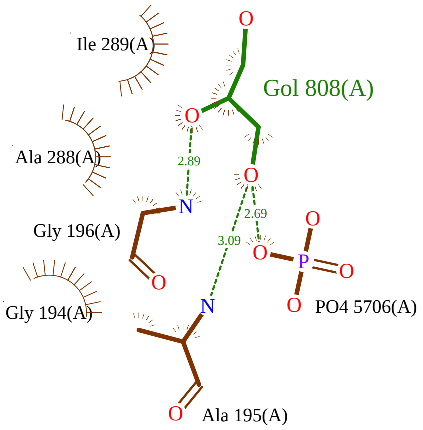

protein	amino acids contacts (binding site)
PDB:2aaq:chainA:P00390	E N R G A G Y I A I G V Q G C G K P I R Y H F T R T L T R
PDB:2gh5:chainA:P00390	E N R G A G Y I A I G V Q G C G K P I R Y H F T R T L T R
sp:P00390:GSHR_HUMAN	E N R G A G Y I A I G V Q G C G K P I R Y H F T R T L T R
tr:F1LRE1:F1LRE1_RAT	E N H G A G Y I A I G V Q G C G K P I R Y H F T R T I T R
sp:P47791:GSHR_MOUSE	E S H G A G Y I A I G V Q G C G K P I R Y H F T R T I T R
sp:P91938:TRXR1_DROME	E D H G A G Y I A I G V E G I G K P V R Y P Y K K V P R R
tr:Q86DB5:Q86DB5_CAEEL	E D K G A G Y I A I G V Q G T G K P I R Y H F N H E V T R
tr:Q93379:Q93379_CAEEL	E D K G A G Y I A I G V Q G T G K P I R Y H F N H E V T R
sp:P41921:GSHR_YEAST	T N P G A G Y I T I G A Q G V G K P I R Y H F T E S D T R

protein	whole protein ident	simil	domain-based ident	simil	contact-based ident	simil
PDB:2aaq:chainA:P00390	1.0	1.0	1.0	1.0	1.0	1.0
PDB:2gh5:chainA:P00390	1.0	1.0	1.0	1.0	1.0	1.0
sp:P00390:GSHR_HUMAN	1.0	1.0	1.0	1.0	1.0	1.0
tr:F1LRE1:F1LRE1_RAT	0.68	0.76	0.78	0.86	0.93	0.99
sp:P47791:GSHR_MOUSE	0.8	0.89	0.88	0.97	0.9	0.97
sp:P91938:TRXR1_DROME	0.28	0.64	0.34	0.71	0.59	0.75
tr:Q86DB5:Q86DB5_CAEEL	0.51	0.74	0.57	0.83	0.76	0.87
tr:Q93379:Q93379_CAEEL	0.5	0.73	0.57	0.83	0.76	0.87
sp:P41921:GSHR_YEAST	0.44	0.73	0.5	0.8	0.72	0.75

Trxr-1 (FBgn0020653) associated phenotypes

aging defective, developmental rate defective, lethal - all die before end of P-stage, lethal - all die before end of pupal stage, neuroanatomy defective, partially, partially lethal - majority die, some die during pharate adult stage, some die during pupal stage, stress response defective

(Information from FlyBase)

Trxr-1 (UniProt:P91938) annotation

Function: Thioredoxin system is a major player in glutathione metabolism, due to the demonstrated absence of a glutathione reductase. Functionally interacts with the Sod/Cat reactive oxidation species (ROS) defense system and thereby has a role in preadult development and life span. Lack of a glutathione reductase suggests antioxidant defense in Drosophila, and probably in related insects, differs fundamentally from that in other organisms. (PubMed:11158675, PubMed:11525742, PubMed:11796729).

Cofactor: FADNote=Binds 1 FAD per subunit.;

Biophysicochemical properties: Kinetic parameters: KM=6.5 uM for NADPH (at pH 7.4) (PubMed:11158675, PubMed:11782468, PubMed:11796729, PubMed:11877442, PubMed:17385893, PubMed:18211101, PubMed:18991392); KM=1 uM for NADPH (at pH 7.4, 2 mM EDTA, 100 mM KPO(4)) (PubMed:11158675, PubMed:11782468, PubMed:11796729, PubMed:11877442, PubMed:17385893, PubMed:18211101, PubMed:18991392); KM=1 uM for NADPH (isoform B at pH 7.4, 2 mM EDTA, 100 mM KPO(4)) (PubMed:11158675, PubMed:11782468, PubMed:11796729, PubMed:11877442, PubMed:17385893, PubMed:18211101, PubMed:18991392); KM=7.0 uM for dhd (at pH 7.4, 200 uM NADPH, 100 mM KPO(4)) (PubMed:11158675, PubMed:11782468, PubMed:11796729, PubMed:11877442, PubMed:17385893, PubMed:18211101, PubMed:18991392); KM=141 uM for dhd (at pH 7.0, 0.15 mM NADPH, 1 mM EDTA, 1 mg/ml insulin, 50 mM KPO(4), 25 degrees Celsius) (PubMed:11158675, PubMed:11782468, PubMed:11796729, PubMed:11877442, PubMed:17385893, PubMed:18211101, PubMed:18991392); KM=7 uM for dhd (at pH 7.4, 2 mM EDTA, 100 mM KPO(4)) (PubMed:11158675, PubMed:11782468, PubMed:11796729, PubMed:11877442, PubMed:17385893, PubMed:18211101, PubMed:18991392); KM=19 uM for dhd (isoform B at pH 7.4, 2 mM EDTA, 100 mM KPO(4)) (PubMed:11158675, PubMed:11782468, PubMed:11796729, PubMed:11877442, PubMed:17385893, PubMed:18211101, PubMed:18991392); KM=5.9 uM for Trx-2 (at pH 7.4, 100 uM NADPH, 2 mM EDTA, 100 mM KPO(4)) (PubMed:11158675, PubMed:11782468, PubMed:11796729, PubMed:11877442, PubMed:17385893, PubMed:18211101, PubMed:18991392); KM=310 uM for 5,5'-dithiobis-2-nitrobenzoic acid (DTNB) (at pH 7.4, 100 uM NADPH, 100 mM KPO(4)) (PubMed:11158675, PubMed:11782468, PubMed:11796729, PubMed:11877442, PubMed:17385893, PubMed:18211101, PubMed:18991392); KM=0.17 mM for DTNB (at pH 7.0, 0.2 mM NADPH, 10 mM EDTA, 100 mM KPO(4), 25 degrees Celsius) (PubMed:11158675, PubMed:11782468, PubMed:11796729, PubMed:11877442, PubMed:17385893, PubMed:18211101, PubMed:18991392); KM=380 uM for DTNB (at pH 7.4, 2 mM EDTA, 100 mM KPO(4)) (PubMed:11158675, PubMed:11782468, PubMed:11796729, PubMed:11877442, PubMed:17385893, PubMed:18211101, PubMed:18991392); KM=410 uM for DTNB (isoform B at pH 7.4, 2 mM EDTA, 100 mM KPO(4)) (PubMed:11158675, PubMed:11782468, PubMed:11796729, PubMed:11877442, PubMed:17385893, PubMed:18211101, PubMed:18991392); KM=675 uM for methylseleninate (100 uM NADPH) (PubMed:11158675, PubMed:11782468, PubMed:11796729, PubMed:11877442, PubMed:17385893, PubMed:18211101, PubMed:18991392); Vmax=24.3 umol/min/mg enzyme toward NADPH (at pH

7.4) (PubMed:11158675, PubMed:11782468, PubMed:11796729, PubMed:11877442, PubMed:17385893, PubMed:18211101, PubMed:18991392); Vmax=16 umol/min/mg enzyme toward Trx-2 (at pH 7.4, 100 uM NADPH, 2 mM EDTA, 100 mM KPO(4), 25 degrees Celsius) (PubMed:11158675, PubMed:11782468, PubMed:11796729, PubMed:11877442, PubMed:17385893, PubMed:18211101, PubMed:18991392); Note=Measurements were conducted with isoform A unless noted otherwise.; pH dependence: Optimum pH is 7.6 for isoform A with Trx-2 and NADPH as substrates. (PubMed:11158675, PubMed:11782468, PubMed:11796729, PubMed:11877442, PubMed:17385893, PubMed:18211101, PubMed:18991392);
Subunit: Homodimer. (PubMed:17385893).
Subcellular location: Isoform B: Mitochondrion.
Subcellular location: Isoform A: Cytoplasm.
Tissue specificity: During embryogenesis, expression is seen in germ cell progenitors, developing midgut, hindgut and proventriculus. (PubMed:11525742).
Developmental stage: Expressed both maternally and zygotically during all stages of development, highest expression during adult stages. (PubMed:11525742, PubMed:11796729).
Miscellaneous: The active site is a redox-active disulfide bond.
Caution: Was originally thought to be a glutathione reductase. (PubMed:9056265).

(Information from UniProt)

gsr-1 (WBGene00008117) associated phenotypes
lethal, sterile

(Information from WormBase)

Superoxide dismutase [Cu-Zn]

Best gene implication in ageing for this target family came from gene P61851 annotated in UniProt release 2014_02. Annotation GO 8340 (determination of adult lifespan) was Inferred from Mutant Phenotype

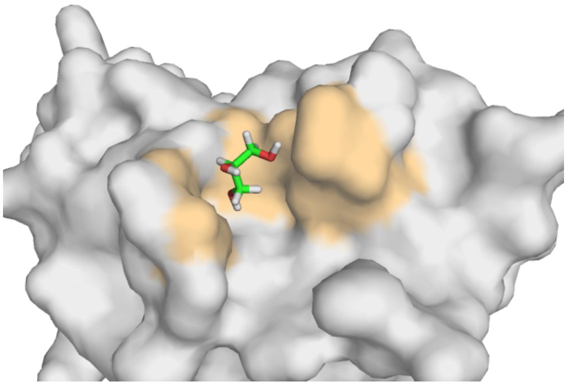

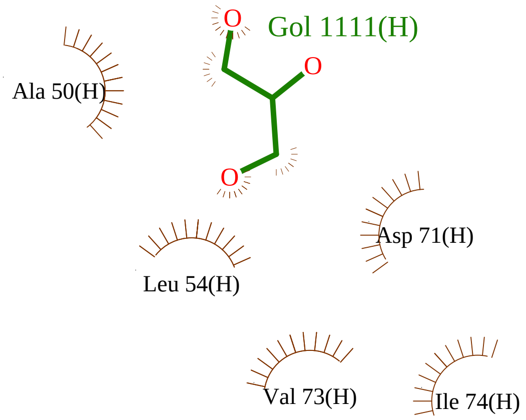

protein	amino acids contacts (binding site)
PDB:4bd4:chainA:P00441	A G D L D V I
PDB:4bd4:chainF:P00441	A G D L D V I
PDB:4bd4:chainH:P00441	A G D L D V I
tr:H7BYH4:H7BYH4_HUMAN	F G D L D V I
sp:P00441:SODC_HUMAN	F G D L D V I
sp:P07632:SODC_RAT	Y G D L D V I
tr:Q6LDS4:Q6LDS4_RAT	Y G D L D V I
sp:P08228:SODC_MOUSE	Y G D L D V I
sp:P61851:SODC_DROME	F G D L D K I
tr:A1Z850:A1Z850_DROME	S G D L D V L
tr:Q9BLY4:Q9BLY4_DROME	S G D L D V L
tr:HORNH3:HORNH3_DROME	S G D L D V L
tr:C6TP82:C6TP82_DROME	S G D L D V L
tr:G5ECR5:G5ECR5_CAEEL	K G D L D L A
sp:P34461:SODE_CAEEL	K G D L D L A
sp:P00445:SODC_YEAST	F G D M D L I

	whole protein		domain-based		contact-based	
protein	ident	simil	ident	simil	ident	simil
PDB:4bd4:chainA:P00441	0.92	0.96	0.92	0.96	1.0	1.0
PDB:4bd4:chainF:P00441	0.94	0.97	0.94	0.97	1.0	1.0
PDB:4bd4:chainH:P00441	0.94	0.97	0.94	0.96	1.0	1.0
tr:H7BYH4:H7BYH4_HUMAN	0.88	0.88	0.87	0.87	0.86	0.82
sp:P00441:SODC_HUMAN	1.0	1.0	1.0	1.0	0.86	0.82
sp:P07632:SODC_RAT	0.83	0.94	0.83	0.94	0.86	0.83
tr:Q6LDS4:Q6LDS4_RAT	0.82	0.93	0.82	0.93	0.86	0.83
sp:P08228:SODC_MOUSE	0.84	0.94	0.83	0.94	0.86	0.83
sp:P61851:SODC_DROME	0.61	0.84	0.6	0.85	0.71	0.67
tr:A1Z850:A1Z850_DROME	0.27	0.46	0.43	0.7	0.71	0.84
tr:Q9BLY4:Q9BLY4_DROME	0.27	0.46	0.43	0.7	0.71	0.84
tr:HORNH3:HORNH3_DROME	0.26	0.44	0.43	0.7	0.71	0.84
tr:C6TP82:C6TP82_DROME	0.26	0.44	0.43	0.7	0.71	0.84
tr:G5ECR5:G5ECR5_CAEEL	0.35	0.57	0.44	0.71	0.57	0.64
sp:P34461:SODE_CAEEL	0.35	0.57	0.44	0.71	0.57	0.64
sp:P00445:SODC_YEAST	0.54	0.83	0.55	0.84	0.57	0.75

Sod (FBgn0003462) associated phenotypes

calorie restriction conditional, chemical conditional, chemical sensitive, heat sensitive, increased cell number, locomotor behavior defective, long lived, male limited, oxidative stress response defective, partially, partially lethal - majority die, progressive, radiation sensitive, reduced, short lived

(Information from FlyBase)

Sod (UniProt:P61851) annotation

Function: Destroys radicals which are normally produced within the cells and which are toxic to biological systems.
Cofactor:Cu cationNote=Binds 1 copper ion per subunit.;
Cofactor: Zn(2+)Note=Binds 1 zinc ion per subunit.;
Subunit: Homodimer.
Subcellular location: Cytoplasm.

(Information from UniProt)

Ccs (FBgn0010531) associated phenotypes

chemical sensitive, short lived

(Information from FlyBase)

Ccs (UniProt:A1Z850) annotation

Function: Destroys radicals which are normally produced within the cells and which are toxic to biological systems. (RuleBase:RU000393).

Cofactor: Cu cation Evidence=(RuleBase:RU000393); Note=Binds 1 copper ion per subunit. (RuleBase:RU000393);
Cofactor: Zn(2+) Evidence=(RuleBase:RU000393); Note=Binds 1 zinc ion per subunit. (RuleBase:RU000393);

(Information from UniProt)

Ccs (UniProt:Q9BLY4) annotation
Function: Destroys radicals which are normally produced within the cells and which are toxic to biological systems. (RuleBase:RU000393).
Cofactor: Cu cation Evidence=(RuleBase:RU000393); Note=Binds 1 copper ion per subunit. (RuleBase:RU000393);
Cofactor: Zn(2+) Evidence=(RuleBase:RU000393); Note=Binds 1 zinc ion per subunit. (RuleBase:RU000393);

(Information from UniProt)

CCS-RB (UniProt:H0RNH3) annotation
Function: Destroys radicals which are normally produced within the cells and which are toxic to biological systems. (RuleBase:RU000393).
Cofactor: Cu cation Evidence=(RuleBase:RU000393); Note=Binds 1 copper ion per subunit. (RuleBase:RU000393);
Cofactor: Zn(2+) Evidence=(RuleBase:RU000393); Note=Binds 1 zinc ion per subunit. (RuleBase:RU000393);

(Information from UniProt)

CCS-RA (UniProt:C6TP82) annotation
Function: Destroys radicals which are normally produced within the cells and which are toxic to biological systems. (RuleBase:RU000393).
Cofactor: Cu cation Evidence=(RuleBase:RU000393); Note=Binds 1 copper ion per subunit. (RuleBase:RU000393);
Cofactor: Zn(2+) Evidence=(RuleBase:RU000393); Note=Binds 1 zinc ion per subunit. (RuleBase:RU000393);

(Information from UniProt)

sod-4 (UniProt:G5ECR5) annotation
Function: Destroys radicals which are normally produced within the cells and which are toxic to biological systems. (RuleBase:RU000393).
Cofactor: Cu cation Evidence=(RuleBase:RU000393); Note=Binds 1 copper ion per subunit. (RuleBase:RU000393);
Cofactor: Zn(2+) Evidence=(RuleBase:RU000393); Note=Binds 1 zinc ion per subunit. (RuleBase:RU000393);

(Information from UniProt)

sod-4 (UniProt:P34461) annotation
Function: Destroys radicals which are normally produced within the cells and which are toxic to biological systems.
Cofactor:Cu cationNote=Binds 1 copper ion per subunit. ;
Cofactor: Zn(2+)Note=Binds 1 zinc ion per subunit. ;
Subcellular location: Isoform 1: Secreted, extracellular space.
Subcellular location: Isoform 2: Membrane ; Peripheral membrane protein
Tissue specificity: Isoform 2 is preferentially expressed in eggs.

(Information from UniProt)

Tyrosine-protein kinase ABL1

Best gene implication in ageing for this target family came from gene P00519 via mapping the annotation from Ensembl ENSG00000097007 via mapping the annotation from EntrezGene 25 via mapping the annotation from GenAgeHuman 0078 annotated in GenAge release 17.

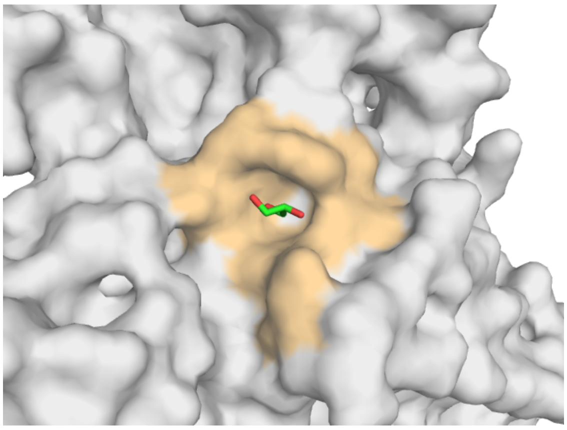

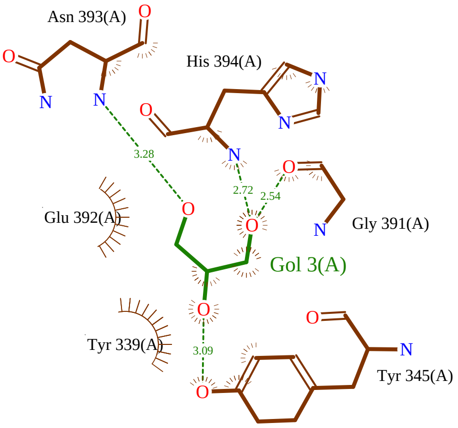

protein	amino acids contacts (binding site)						
PDB:1opk:chainA:P00520	Y	Y	E	G	E	N	H
sp:P00519:ABL1_HUMAN	Y	Y	E	G	E	N	H
tr:Q59FK4:Q59FK4_HUMAN	Y	Y	E	G	E	N	H
tr:F1M0A6:F1M0A6_RAT	Y	Y	E	G	E	N	H
tr:D3ZGM3:D3ZGM3_RAT	Y	Y	E	G	E	N	H
tr:E9PT20:E9PT20_RAT	Y	Y	E	G	E	N	H
tr:Q3V2W1:Q3V2W1_MOUSE	Y	Y	E	G	E	N	H
sp:P00520:ABL1_MOUSE	Y	Y	E	G	E	N	H
tr:Q3SYK5:Q3SYK5_MOUSE	Y	Y	E	G	E	N	H
sp:P00522:ABL_DROME	H	F	T	G	D	N	K
tr:A8JNU2:A8JNU2_DROME	H	F	T	G	D	N	K
sp:P03949:ABL1_CAEEL	N	Y	L	S	E	H	N

	whole protein		domain-based		contact-based	
protein	ident	simil	ident	simil	ident	simil
PDB:1opk:chainA:P00520	1.0	1.0	1.0	1.0	1.0	1.0
sp:P00519:ABL1_HUMAN	0.88	0.95	1.0	1.0	1.0	1.0
tr:Q59FK4:Q59FK4_HUMAN	0.83	0.92	1.0	1.0	1.0	1.0
tr:F1M0A6:F1M0A6_RAT	0.93	0.96	1.0	1.0	1.0	1.0
tr:D3ZGM3:D3ZGM3_RAT	0.96	0.99	1.0	1.0	1.0	1.0
tr:E9PT20:E9PT20_RAT	0.93	0.96	1.0	1.0	1.0	1.0
tr:Q3V2W1:Q3V2W1_MOUSE	0.33	0.33	1.0	1.0	1.0	1.0
sp:P00520:ABL1_MOUSE	1.0	1.0	1.0	1.0	1.0	1.0
tr:Q3SYK5:Q3SYK5_MOUSE	0.96	0.98	1.0	1.0	1.0	1.0
sp:P00522:ABL_DROME	0.29	0.51	0.78	0.93	0.29	0.7
tr:A8JNU2:A8JNU2_DROME	0.29	0.5	0.78	0.93	0.29	0.7
sp:P03949:ABL1_CAEEL	0.22	0.43	0.68	0.9	0.29	0.46

Abl (FBgn0000017) associated phenotypes

cell migration defective, cell shape defective, germline clone, heat sensitive, lethal - all die before end of pupal stage, maternal effect, mitotic cell cycle defective, neuroanatomy defective, neurophysiology defective, partially lethal - majority die, partially lethal - majority live, reduced, rescuable maternal effect, size defective, somatic clone, some die during pupal stage

(Information from FlyBase)

Abl (UniProt:P00522) annotation

Function: Arm and Abl proteins function cooperatively at adherens junctions in both the CNS and epidermis; critical for embryonic epithelial morphogenesis regulating cell shape changes and cell

migration. Plays a critical role in transducing embryonic midline repulsive cues; may regulate cytoskeletal dynamics underlying a growth cone's response to midline cues. The ability of pCC/MP2 axons to correctly interpret midline repulsive cues and stay on the ipsilateral side is dependent on the strength of both Slit/robo and Abl-dependent signaling pathways. (PubMed:11756472, PubMed:12973825, PubMed:9635189).
Subcellular location: Cytoplasm.
Tissue specificity: Arm and ena colocalize with Abl at adherens junctions throughout development. (PubMed:11756472, PubMed:9635189).
Developmental stage: Expressed both maternally and zygotically. (PubMed:2832740).
Disruption phenotype: Both loss- and gain-of-function mutants exhibit neurons within the pCC/MP2 pathway that incorrectly project across the midline. Loss of Abl disrupts cell migration and cell shape changes during dorsal closure. (PubMed:11756472, PubMed:9635189).

(Information from UniProt)

abl-1 (WBGene00000018) associated phenotypes
apoptosis variant, gamma ray induced apoptosis increased, lipid synthesis increased, protein subcellular localization variant

(Information from WormBase)

Adenosine deaminase

Best gene implication in ageing for this target family came from gene Q920P6 annotated in UniProt release 2014_02. Annotation GO 7568 (aging) was Inferred from Expression Pattern

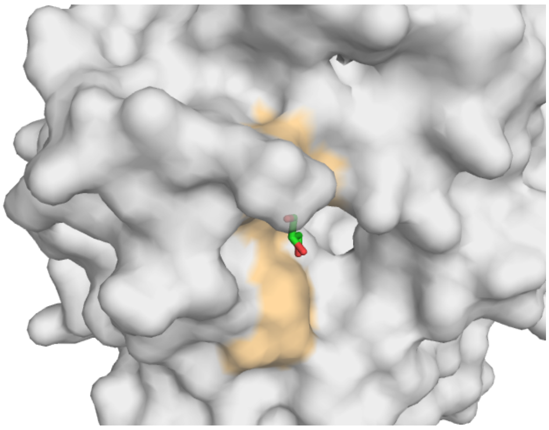

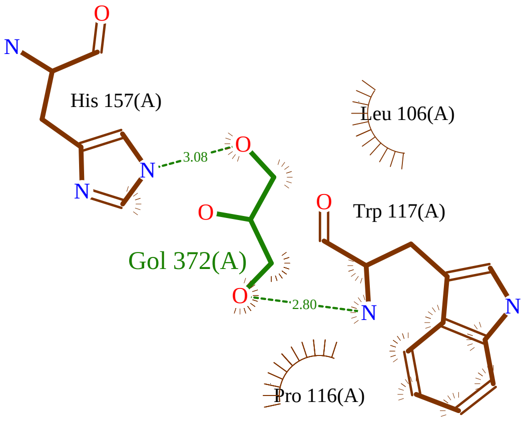

protein	amino acids contacts (binding site)
PDB:3iar:chainA:P00813	L P W H
tr:F5GXW0:F5GXW0_HUMAN	L P W H
tr:F5GWI4:F5GWI4_HUMAN	L P W H
sp:P00813:ADA_HUMAN	L P W H
sp:Q920P6:ADA_RAT	L P W H
sp:P03958:ADA_MOUSE	L P W H
tr:Q4FK28:Q4FK28_MOUSE	L P W H
tr:Q86NI2:Q86NI2_CAEEL	L - - G
tr:H2KYI5:H2KYI5_CAEEL	L - - G
sp:P53909:ADE_YEAST	- - - H

protein	whole protein ident	simil	domain-based ident	simil	contact-based ident	simil
PDB:3iar:chainA:P00813	1.0	1.0	1.0	1.0	1.0	1.0
tr:F5GXW0:F5GXW0_HUMAN	0.72	0.72	0.73	0.74	1.0	1.0
tr:F5GWI4:F5GWI4_HUMAN	0.93	0.93	0.93	0.93	1.0	1.0
sp:P00813:ADA_HUMAN	1.0	1.0	1.0	1.0	1.0	1.0
sp:Q920P6:ADA_RAT	0.83	0.93	0.86	0.96	1.0	1.0
sp:P03958:ADA_MOUSE	0.81	0.93	0.83	0.96	1.0	1.0
tr:Q4FK28:Q4FK28_MOUSE	0.81	0.93	0.83	0.96	1.0	1.0
tr:Q86NI2:Q86NI2_CAEEL	0.37	0.76	0.39	0.78	0.25	0.0
tr:H2KYI5:H2KYI5_CAEEL	0.34	0.69	0.39	0.78	0.25	0.0
sp:P53909:ADE_YEAST	0.24	0.67	0.25	0.69	0.25	0.0

Triosephosphate isomerase

Best gene implication in ageing for this target family came from gene P29613 annotated in UniProt release 2014_02. Annotation GO 8340 (determination of adult lifespan) was Inferred from Mutant Phenotype

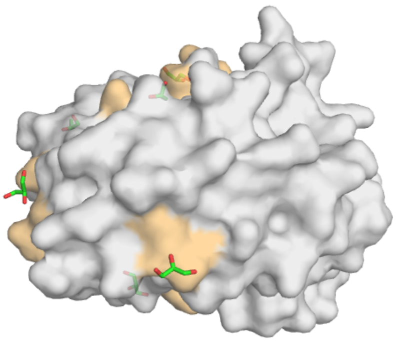

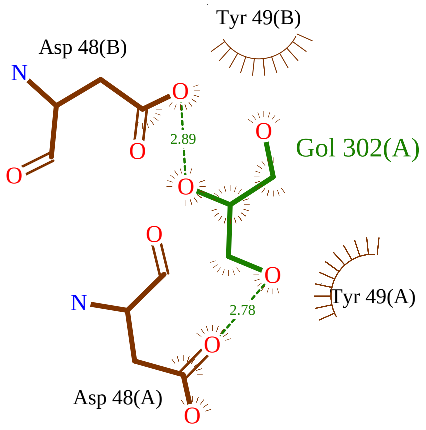

protein	amino acids contacts (binding site)																				
PDB:4ff7:chainA:P00942	K	R	D	Y	K	A	S	K	W	Y	F	H	F	F	G	E	V	K	D	N	P
sp:P60174:TPIS_HUMAN	K	T	D	F	V	T	N	T	W	V	F	G	L	H	G	N	V	K	D	K	P
tr:M0R4U4:M0R4U4_RAT	K	T	D	F	V	T	N	T	W	I	F	G	L	H	G	N	V	K	D	K	P
sp:P48500:TPIS_RAT	K	T	D	F	V	T	N	T	W	I	F	G	L	H	G	N	V	K	D	K	P
sp:P17751:TPIS_MOUSE	K	T	D	F	V	T	N	T	W	V	F	G	L	H	G	N	V	K	D	K	P
sp:P29613:TPIS_DROME	Q	T	M	Y	V	A	K	D	W	I	F	G	L	H	K	K	I	K	D	N	P
sp:Q10657:TPIS_CAEEL	Y	F	A	Y	V	P	K	E	W	V	F	G	L	H	K	K	G	V	S	N	P
sp:P00942:TPIS_YEAST	K	R	D	Y	K	A	S	K	W	Y	F	H	F	F	G	E	V	K	D	N	P

protein	whole protein ident	simil	domain-based ident	simil	contact-based ident	simil
PDB:4ff7:chainA:P00942	1.0	1.0	1.0	1.0	1.0	1.0
sp:P60174:TPIS_HUMAN	0.46	0.72	0.53	0.84	0.43	0.66
tr:M0R4U4:M0R4U4_RAT	0.46	0.75	0.49	0.79	0.43	0.67
sp:P48500:TPIS_RAT	0.51	0.82	0.52	0.83	0.43	0.67
sp:P17751:TPIS_MOUSE	0.43	0.69	0.52	0.83	0.43	0.66
sp:P29613:TPIS_DROME	0.56	0.85	0.56	0.85	0.38	0.54
sp:Q10657:TPIS_CAEEL	0.54	0.84	0.54	0.84	0.24	0.51
sp:P00942:TPIS_YEAST	1.0	1.0	1.0	1.0	1.0	1.0

Tpi (FBgn0086355) associated phenotypes

bang sensitive, chemical conditional, chemical resistant, chemical sensitive, fast, heat sensitive, heat stress response defective, neuroanatomy defective, oxidative stress response defective, short lived, stress response defective

(Information from FlyBase)

Tpi (UniProt:P29613) annotation
Pathway: Carbohydrate biosynthesis; gluconeogenesis.
Pathway: Carbohydrate degradation; glycolysis; D-glyceraldehyde 3- phosphate from glycerone phosphate: step 1/1.
Subunit: Homodimer.
Developmental stage: Present in substantial amounts in oocytes, declines in abundance in early embryos, and begins to increase during mid-embryogenesis. Levels peak in the third instar larva then decline during pupal stages, rising again near the time of eclosion. (PubMed:1720860).

(Information from UniProt)

tpi-1 (UniProt:Q10657) annotation
Pathway: Carbohydrate biosynthesis; gluconeogenesis.
Pathway: Carbohydrate degradation; glycolysis; D-glyceraldehyde 3- phosphate from glycerone phosphate: step 1/1.
Subunit: Homodimer.

(Information from UniProt)

GTPase HRas

Best gene implication in ageing for this target family came from gene P01112 annotated in UniProt release 2014_02. Annotation GO subterm of 7568 (aging) was Inferred from Direct Assay

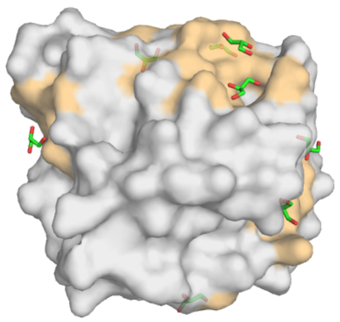

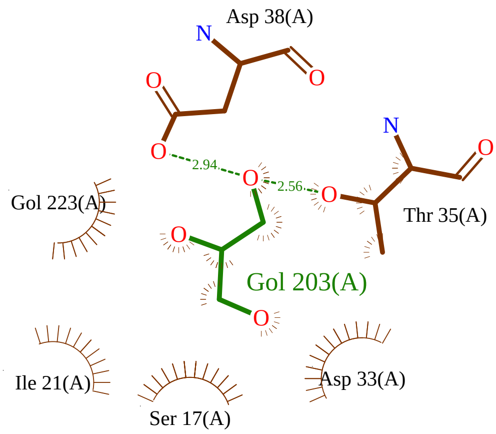

protein	amino acids contacts (binding site)
PDB:2c5l:chainA:P01112	T E Y A V S I Q L I Q N H V D P T D S Y R K V Q Y M E N N K H K L L Y K R E Y
PDB:3rrz:chainA:P01112	T E Y A G S I Q L I Q N H V D P T D S Y R K V Q Y M E N N K H K L L Y K R E Y
PDB:4k81:chainB:P01112	T E Y A V S I Q L I Q N H V D P T D S Y R K V Q Y M E N N K H K L L Y K R E Y
tr:L7RSL8:L7RSL8_HUMAN	T E Y A G S I Q L I Q N H V D P T D S Y R K V Q Y M E N N K H K L L Y K R E Y
sp:P01112:RASH_HUMAN	T E Y A G S I Q L I Q N H V D P T D S Y R K V Q Y M E N N K H K L L Y K R E Y
sp:P01116:RASK_HUMAN	T E Y A G S I Q L I Q N H V D P T D S Y R K V Q Y M E N N K H K L L Y K R E Y
tr:I1SRC5:I1SRC5_HUMAN	T E Y A G S I Q L I Q N H V D P T D S Y R K V Q Y M E N N K H K L L Y K R E Y
sp:P20171:RASH_RAT	T E Y A G S I Q L I Q N H V D P T D S Y R K V Q Y M E N N K H K L L Y K R E Y
sp:P08644:RASK_RAT	T E Y A G S I Q L I Q N H V D P T D S Y R K V Q Y M E N N K H K L L Y K R E Y
sp:Q61411:RASH_MOUSE	T E Y A G S I Q L I Q N H V D P T D S Y R K V Q Y M E N N K H K L L Y K R E Y
sp:P32883:RASK_MOUSE	T E Y A G S I Q L I Q N H V D P T D S Y R K V Q Y M E N N K H K L L Y K R E Y
tr:Q0VDV7:Q0VDV7_MOUSE	T E Y A G S I Q L I Q N H V D P T D S Y R K V Q Y M E N N K H K L L Y K R E Y
sp:P08646:RAS1_DROME	T E Y A G S I Q L I Q N H V D P T D S Y R K V Q Y M E N S K G K L V Y K R D Y
sp:P22981:LET60_CAEEL	T E Y D G S I Q L I Q N H V D P T D S Y R K V Q Y M E N E K A K L T Y K R D Y
sp:P01120:RAS2_YEAST	R E Y G G S I Q L T Q S H V D P T D S Y R K V Q Y M E T S S M K L M M K A E Y

protein	whole protein ident	simil	domain-based ident	simil	contact-based ident	simil
PDB:2c5l:chainA:P01112	0.99	1.0	0.99	1.0	1.0	1.0
PDB:3rrz:chainA:P01112	1.0	1.0	1.0	1.0	0.97	0.97
PDB:4k81:chainB:P01112	0.96	0.97	0.99	1.0	1.0	1.0
tr:L7RSL8:L7RSL8_HUMAN	0.85	0.95	0.93	0.98	0.97	0.97
sp:P01112:RASH_HUMAN	1.0	1.0	1.0	1.0	0.97	0.97
sp:P01116:RASK_HUMAN	0.85	0.95	0.93	0.98	0.97	0.97
tr:I1SRC5:I1SRC5_HUMAN	0.54	0.61	0.93	0.98	0.97	0.97
sp:P20171:RASH_RAT	1.0	1.0	1.0	1.0	0.97	0.97
sp:P08644:RASK_RAT	0.85	0.95	0.92	0.98	0.97	0.97
sp:Q61411:RASH_MOUSE	1.0	1.0	1.0	1.0	0.97	0.97
sp:P32883:RASK_MOUSE	0.85	0.95	0.92	0.98	0.97	0.97
tr:Q0VDV7:Q0VDV7_MOUSE	0.85	0.95	0.92	0.98	0.97	0.97
sp:P08646:RAS1_DROME	0.77	0.91	0.85	0.95	0.87	0.92
sp:P22981:LET60_CAEEL	0.74	0.89	0.81	0.94	0.85	0.88
sp:P01120:RAS2_YEAST	0.34	0.5	0.62	0.88	0.69	0.79

Ras85D (FBgn0003205) associated phenotypes

RU486 conditional, cell autonomous, cell death defective, cell non-autonomous, cell polarity defective, cell size defective, conditional, decreased cell death, decreased cell number, decreased cell size, developmental rate defective, dominant, endocytosis defective, female semi-sterile, germline clone, heat sensitive, hyperplasia, increased cell death, increased cell growth, increased cell number, increased cell size, large body, learning defective, lethal - all die before end of P-stage, lethal - all die before end of pupal stage, majority die during P-stage, maternal effect, melanotic mass phenotype, mitotic cell cycle defective, neoplasia, neuroanatomy defective, neurophysiology defective, partially, partially lethal - majority die, short lived, size defective, small body, somatic clone, some die during P-stage, some die during pharate adult stage, some die during pupal stage, temperature conditional, terminal phenotype, tumorigenic

(Information from FlyBase)

Ras85D (UniProt:P08646) annotation

Function: May mediate a signal that determines the fate of photoreceptor cells in the developing compound eye. Ras proteins bind GDP/GTP and possess intrinsic GTPase activity.

Enzyme regulation: Alternates between an inactive form bound to GDP and an active form bound to GTP. Activated by a guanine nucleotide-exchange factor (GEF) and inactivated by a GTPase- activating protein (GAP).

Subcellular location: Cell membrane (PubMed:18503409); Lipid-anchor (PubMed:18503409); Cytoplasmic side (PubMed:18503409). Note=Loss of prenylation causes protein location to the cytoplasm.

Tissue specificity: Expressed in the posterior termini of the embryo, restricted mainly to the embryonic central nervous system, and in the eye imaginal disk. (PubMed:7873789).

(Information from UniProt)

let-60 (WBGene00002335) associated phenotypes

AWA odorant chemotaxis defective, AWC odorant chemotaxis defective, L2 arrest, L3 arrest, antibody staining reduced, antibody staining variant, axon outgrowth variant, bloated, cell differentiation variant, egg laying defective, egg laying imipramine resistant, egg laying variant, germ cell development variant, gonad arm morphology variant, lethal, locomotion variant, male mating defective, mid larval lethal, multivulva, neuron degeneration, oocyte accumulation, oocytes small, organism starvation response variant, protein expression absent, reduced brood size, rod like larval lethal, sterile, unfertilized oocytes laid, vulval cell induction increased, vulvaless

(Information from WormBase)

let-60 (UniProt:P22981) annotation

Function: The level of let-60 controls the switch between vulval and hypodermal cell fates during C.elegans vulval induction. May stimulate the guanine nucleotide exchange factor (GEF) activity

of rap-1.
Subunit: Interacts with soc-2. (PubMed:9674433).
Subcellular location: Cell membrane; Lipid-anchor.

(Information from UniProt)

GTPase KRas

Best gene implication in ageing for this target family came from gene P01119 annotated in UniProt release 2014_02. Annotation GO subterm of 7568 (aging) was Inferred from Mutant Phenotype

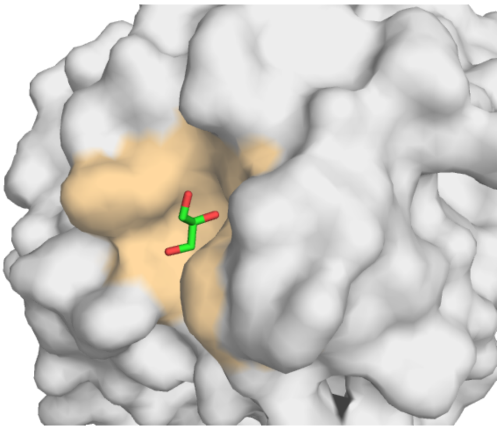

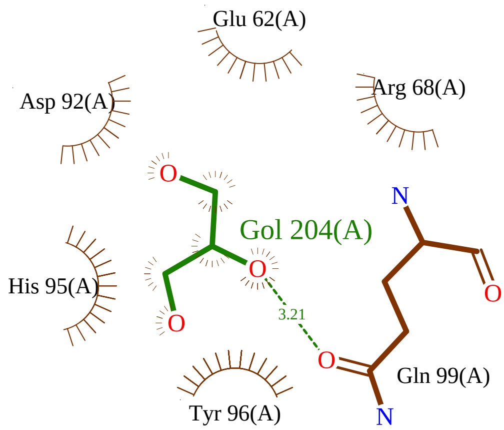

protein	amino acids contacts (binding site)
PDB:4dso:chainA:P01116	E R D H Y Q
PDB:4dst:chainA:P01116	E R D H Y Q
tr:L7RSL8:L7RSL8_HUMAN	E R D H Y Q
sp:P01116:RASK_HUMAN	E R D H Y Q
tr:I1SRC5:I1SRC5_HUMAN	E R D H Y Q
sp:P08644:RASK_RAT	E R D H Y Q
tr:A0JN17:A0JN17_RAT	E R D H Y Q
sp:P32883:RASK_MOUSE	E R D H Y Q
tr:Q0VDV7:Q0VDV7_MOUSE	E R D H Y Q
tr:Q5J7N1:Q5J7N1_MOUSE	E R D H Y Q
sp:P08646:RAS1_DROME	E R D T Y Q
sp:P22981:LET60_CAEEL	E R N N Y Q
sp:P01119:RAS1_YEAST	E R E S Y Q
sp:P01120:RAS2_YEAST	E R E T Y Q

	whole protein		domain-based		contact-based	
protein	ident	simil	ident	simil	ident	simil
PDB:4dso:chainA:P01116	0.91	0.96	0.95	0.98	1.0	1.0
PDB:4dst:chainA:P01116	0.91	0.96	0.95	0.98	1.0	1.0
tr:L7RSL8:L7RSL8_HUMAN	1.0	1.0	1.0	1.0	1.0	1.0
sp:P01116:RASK_HUMAN	1.0	1.0	1.0	1.0	1.0	1.0
tr:I1SRC5:I1SRC5_HUMAN	0.64	0.64	1.0	1.0	1.0	1.0
sp:P08644:RASK_RAT	0.99	1.0	0.99	1.0	1.0	1.0
tr:A0JN17:A0JN17_RAT	0.89	0.95	0.94	0.98	1.0	1.0
sp:P32883:RASK_MOUSE	0.99	1.0	0.99	1.0	1.0	1.0
tr:Q0VDV7:Q0VDV7_MOUSE	0.99	1.0	0.99	1.0	1.0	1.0
tr:Q5J7N1:Q5J7N1_MOUSE	0.88	0.95	0.94	0.98	1.0	1.0
sp:P08646:RAS1_DROME	0.76	0.91	0.81	0.93	0.83	0.93
sp:P22981:LET60_CAEEL	0.75	0.89	0.77	0.92	0.67	0.86
sp:P01119:RAS1_YEAST	0.35	0.51	0.6	0.86	0.67	0.79
sp:P01120:RAS2_YEAST	0.34	0.5	0.61	0.86	0.67	0.86

Ras85D (FBgn0003205) associated phenotypes

RU486 conditional, cell autonomous, cell death defective, cell non-autonomous, cell polarity defective, cell size defective, conditional, decreased cell death, decreased cell number, decreased cell size, developmental rate defective, dominant, endocytosis defective, female semi-sterile, germline clone, heat sensitive, hyperplasia, increased cell death, increased cell growth, increased cell number, increased cell size, large body, learning defective, lethal - all die before end of P-stage, lethal - all die before end of pupal stage, majority die during P-stage, maternal effect, melanotic mass phenotype, mitotic cell cycle defective, neoplasia, neuroanatomy defective, neurophysiology defective, partially, partially lethal - majority die, short lived, size defective, small body, somatic clone, some die during P-stage, some die during pharate adult stage, some die during pupal stage, temperature conditional, terminal phenotype, tumorigenic

(Information from FlyBase)

Ras85D (UniProt:P08646) annotation

Function: May mediate a signal that determines the fate of photoreceptor cells in the developing compound eye. Ras proteins bind GDP/GTP and possess intrinsic GTPase activity.
Enzyme regulation: Alternates between an inactive form bound to GDP and an active form bound to GTP. Activated by a guanine nucleotide-exchange factor (GEF) and inactivated by a GTPase- activating protein (GAP).
Subcellular location: Cell membrane (PubMed:18503409); Lipid-anchor (PubMed:18503409); Cytoplasmic side (PubMed:18503409). Note=Loss of prenylation causes protein location to the cytoplasm.
Tissue specificity: Expressed in the posterior termini of the embryo, restricted mainly to the embryonic central nervous system, and in the eye imaginal disk. (PubMed:7873789).

(Information from UniProt)

let-60 (WBGene00002335) associated phenotypes

AWA odorant chemotaxis defective, AWC odorant chemotaxis defective, L2 arrest, L3 arrest, antibody staining reduced, antibody staining variant, axon outgrowth variant, bloated, cell differentiation variant, egg laying defective, egg laying imipramine resistant, egg laying variant, germ cell development variant, gonad arm morphology variant, lethal, locomotion variant, male mating defective, mid larval lethal, multivulva, neuron degeneration, oocyte accumulation, oocytes small, organism starvation response variant, protein expression absent, reduced brood size, rod like larval lethal, sterile, unfertilized oocytes laid, vulval cell induction increased, vulvaless

(Information from WormBase)

let-60 (UniProt:P22981) annotation

Function: The level of let-60 controls the switch between vulval and hypodermal cell fates during C.elegans vulval induction. May stimulate the guanine nucleotide exchange factor (GEF) activity of rap-1.
Subunit: Interacts with soc-2. (PubMed:9674433).
Subcellular location: Cell membrane; Lipid-anchor.

(Information from UniProt)

Collagen alpha-1(III) chain

Best gene implication in ageing for this target family came from gene P13941 annotated in UniProt release 2014_02. Annotation GO 7568 (aging) was Inferred from Expression Pattern

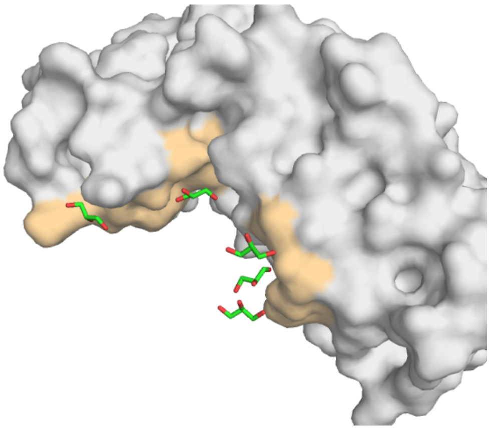

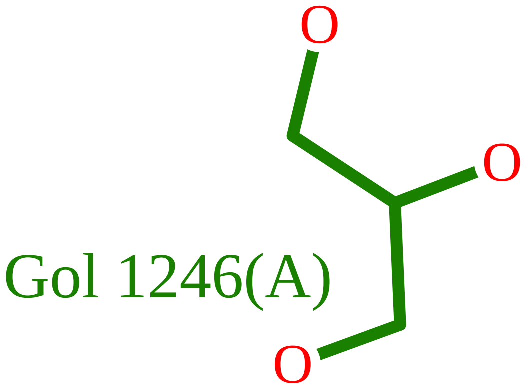

protein	amino acids contacts (binding site)
PDB:4aej:chainA:P02461	K S F N E C L E K V C
tr:E7ENY8:E7ENY8_HUMAN	K S F N E C L E K V C
sp:P02461:CO3A1_HUMAN	K S F N E C L E K V C
sp:P13941:CO3A1_RAT	K S F N E C L E K V C
tr:Q8BMR6:Q8BMR6_MOUSE	K S F N E C L E K V C
tr:Q8BJU6:Q8BJU6_MOUSE	K S F N E C L E K V C
tr:Q3TVI5:Q3TVI5_MOUSE	K S F N E C L E K V C
tr:Q5DTG2:Q5DTG2_MOUSE	K S F N E C L E K V C
sp:P08121:CO3A1_MOUSE	K S F N E C L E K V C
tr:B7FNQ3:B7FNQ3_DROME	S T L V Q N E C T T C
sp:P08120:CO4A1_DROME	S T L V Q N E C T T C

protein	whole protein ident	whole protein simil	domain-based ident	domain-based simil	contact-based ident	contact-based simil
PDB:4aej:chainA:P02461	0.99	1.0	1.0	1.0	1.0	1.0
tr:E7ENY8:E7ENY8_HUMAN	0.79	0.79	1.0	1.0	1.0	1.0
sp:P02461:CO3A1_HUMAN	1.0	1.0	1.0	1.0	1.0	1.0
sp:P13941:CO3A1_RAT	0.91	0.97	0.92	0.98	1.0	1.0
tr:Q8BMR6:Q8BMR6_MOUSE	0.14	0.15	0.89	0.95	1.0	1.0
tr:Q8BJU6:Q8BJU6_MOUSE	0.21	0.23	0.92	0.98	1.0	1.0
tr:Q3TVI5:Q3TVI5_MOUSE	0.91	0.97	0.92	0.98	1.0	1.0
tr:Q5DTG2:Q5DTG2_MOUSE	0.9	0.97	0.92	0.98	1.0	1.0
sp:P08121:CO3A1_MOUSE	0.91	0.97	0.92	0.98	1.0	1.0
tr:B7FNQ3:B7FNQ3_DROME	0.32	0.58	0.12	0.45	0.09	0.35
sp:P08120:CO4A1_DROME	0.32	0.58	0.12	0.45	0.09	0.35

Cg25C (FBgn0000299) associated phenotypes

cold sensitive, dominant, heat sensitive, locomotor behavior defective, majority die during embryonic stage, neuroanatomy defective, partially, partially lethal - majority die, partially lethal - majority live, small body

(Information from FlyBase)

Cg25C (UniProt:P08120) annotation

Function: Collagen type IV is specific for basement membranes.
Subunit: Trimers of two alpha 1(IV) and one alpha 2(IV) chain. Type IV collagen forms a mesh-like network linked through intermolecular interactions between 7S domains and between NC1 domains.
Subcellular location: Secreted, extracellular space, extracellular matrix, basement membrane.
Domain: Alpha chains of type IV collagen have a non-collagenous domain (NC1) at their C-terminus, frequent interruptions of the G- X-Y repeats in the long central triple-helical domain (which may cause flexibility in the triple helix), and a short N-terminal triple-helical 7S domain.
Ptm: Prolines at the third position of the tripeptide repeating unit (G-X-Y) are hydroxylated in some or all of the chains.
Ptm: Type IV collagens contain numerous cysteine residues which are involved in inter- and in-

tramolecular disulfide bonding. 12 of these, located in the NC1 domain, are conserved in all known type IV collagens.

(Information from UniProt)

Prelamin-A/C

Best gene implication in ageing for this target family came from gene P08928 annotated in UniProt release 2014_02. Annotation GO subterm of 7568 (aging) was Inferred from Mutant Phenotype

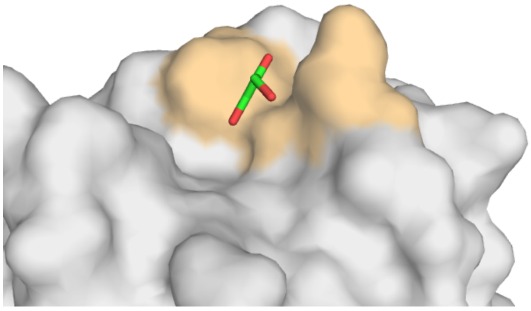

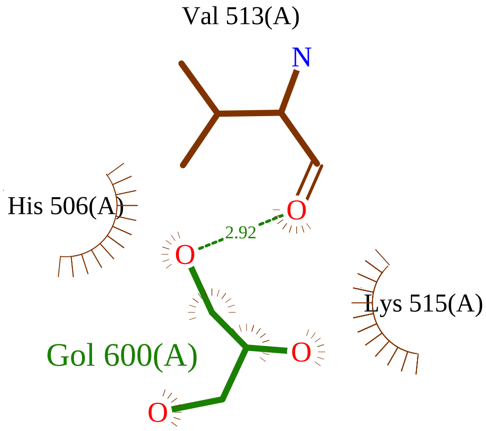

protein	amino acids contacts (binding site)
PDB:1ifr:chainA:P02545	H V K
tr:B4DFR3:B4DFR3_HUMAN	H V K
tr:Q5TCI8:Q5TCI8_HUMAN	H V K
sp:Q03252:LMNB2_HUMAN	H V K
tr:J9JID7:J9JID7_HUMAN	H V K
sp:P02545:LMNA_HUMAN	H V K
tr:MORCR1:MORCR1_RAT	H V K
tr:D3ZLC1:D3ZLC1_RAT	H V K
tr:G3V8L3:G3V8L3_RAT	H V K
tr:Q9CTL2:Q9CTL2_MOUSE	H V K
tr:Q3V159:Q3V159_MOUSE	H V K
sp:P21619:LMNB2_MOUSE	H V K
sp:P48678:LMNA_MOUSE	H V K
sp:Q03427:LAMC_DROME	H V K
sp:P08928:LAMO_DROME	H V K
sp:Q21443:LMN1_CAEEL	H V K

	whole protein		domain-based		contact-based	
protein	ident	simil	ident	simil	ident	simil
PDB:1ifr:chainA:P02545	0.99	1.0	0.99	1.0	1.0	1.0
tr:B4DFR3:B4DFR3_HUMAN	0.34	0.34	1.0	1.0	1.0	1.0
tr:Q5TCI8:Q5TCI8_HUMAN	0.69	0.72	1.0	1.0	1.0	1.0
sp:Q03252:LMNB2_HUMAN	0.46	0.71	0.46	0.74	1.0	1.0
tr:J9JID7:J9JID7_HUMAN	0.45	0.7	0.46	0.74	1.0	1.0
sp:P02545:LMNA_HUMAN	1.0	1.0	1.0	1.0	1.0	1.0
tr:MORCR1:MORCR1_RAT	0.29	0.51	0.45	0.75	1.0	1.0
tr:D3ZLC1:D3ZLC1_RAT	0.44	0.7	0.45	0.75	1.0	1.0
tr:G3V8L3:G3V8L3_RAT	0.97	0.99	0.95	0.99	1.0	1.0
tr:Q9CTL2:Q9CTL2_MOUSE	0.14	0.15	0.52	0.54	1.0	1.0
tr:Q3V159:Q3V159_MOUSE	0.3	0.54	0.47	0.77	1.0	1.0
sp:P21619:LMNB2_MOUSE	0.45	0.71	0.47	0.77	1.0	1.0
sp:P48678:LMNA_MOUSE	0.96	0.99	0.96	0.99	1.0	1.0
sp:Q03427:LAMC_DROME	0.3	0.62	0.31	0.66	1.0	1.0
sp:P08928:LAMO_DROME	0.3	0.64	0.31	0.69	1.0	1.0
sp:Q21443:LMN1_CAEEL	0.24	0.6	0.27	0.66	1.0	1.0

LamC (FBgn0010397) associated phenotypes
developmental rate defective, dominant, heat sensitive, increased cell death, lethal - all die before end of P-stage, lethal - all die before end of first instar larval stage, lethal - all die before end of prepupal stage, locomotor behavior defective, melanotic mass phenotype, partially lethal - majority die, partially lethal - majority live, some die during P-stage, some die during prepupal stage, some die during pupal stage, some die during third instar larval stage, suppressor of variegation, temperature conditional

(Information from FlyBase)

LamC (UniProt:Q03427) annotation
Function: Lamins are components of the nuclear lamina, a fibrous layer on the nucleoplasmic side of the inner nuclear membrane, which is thought to provide a framework for the nuclear envelope and may also interact with chromatin.
Subcellular location: Nucleus (PubMed:7593280). Note=Nuclear periphery.
Tissue specificity: First detected from late stage 12 in the oenocytes, abdominal segments, hindgut and posterior spiracles, with expression increasing in stage 13. In stage 14, also becomes detectable in the foregut. Stage 15 shows expression in the epidermis, dorsal longitudinal trunk, pharynx, esophagus and proventriculus, with the dorsal pharyngeal musculature showing expression in late stage 15. In stage 16 embryos, also detected in the exit glia with increasing expression in the somatic musculature. Also detected in the visceral mesoderm but not in the midgut or central nervous system until the end of embryogenesis. In third instar larvae, detectable at varying levels in all cell types. (PubMed:7593280).
Developmental stage: Expressed in 12-22 hours embryos. Expression peaks in larvae and declines in pupae and adults. (PubMed:7593280).

(Information from UniProt)

Lam (FBgn0002525) associated phenotypes
cell growth defective, developmental rate defective, flight defective, flightless, germline clone, gravitaxis defective, hyperplasia, locomotor behavior defective, partially, partially lethal - majority die, partially lethal - majority live, short lived, some die during pupal stage, some die during third instar larval stage, sterile, temperature conditional, uncoordinated

(Information from FlyBase)

Lam (UniProt:P08928) annotation
Function: Lamins are components of the nuclear lamina, a fibrous layer on the nucleoplasmic side of the inner nuclear membrane, which is thought to provide a framework for the nuclear envelope and may also interact with chromatin. (PubMed:3126192).
Subunit: Interacts directly with LBR. (PubMed:15054108).
Subcellular location: Nucleus (PubMed:7593280). Note=Nuclear periphery.
Tissue specificity: Constitutively expressed in all tissues. (PubMed:7593280).
Developmental stage: Constitutively expressed in all developmental stages, especially during the first 6-9 hours. (PubMed:7593280).
Ptm: Three forms of lamin have been identified in D.melanogaster, lamin Dm0 is rapidly processed to lamin Dm1 in the cytoplasm, Dm1 is then assembled in the nuclear envelope and is then phosphorylated, forming lamin Dm2. (PubMed:18327897, PubMed:3126192).

(Information from UniProt)

lmn-1 (WBGene00003052) associated phenotypes
lethal, sterile, transgene expression increased

(Information from WormBase)

lmn-1 (UniProt:Q21443) annotation

Function: Major component of the nuclear lamina, a fibrous layer on the nucleoplasmic side of the inner nuclear membrane. Provides a framework for the nuclear envelope and probably also interacts with chromatin. Essential to maintain the shape and integrity of the nucleus, and for DNA replication. Involved in spatial organization of nuclear pore complexes. It is not a target for ced-3 during apoptosis, suggesting that lamin cleavage is not essential for apoptosis in C.elegans. (PubMed:11071918, PubMed:12064941).
Subunit: Interacts with LEM domain proteins lem-2 and emr-1. (PubMed:11870211, PubMed:12684533).
Subcellular location: Nucleus inner membrane (PubMed:10982402, PubMed:11071918, PubMed:12490171); Lipid-anchor (PubMed:10982402, PubMed:11071918, PubMed:12490171); Nucleoplasmic side (PubMed:10982402, PubMed:11071918, PubMed:12490171). Note=Remains in the nuclear envelope until late anaphase in early embryos.
Tissue specificity: Ubiquitous. Expressed in all cells, except in cells undergoing spermatogenesis. (PubMed:11071918).
Developmental stage: Expressed throughout the development and in adults. (PubMed:11071918).
Miscellaneous: It is the only lamin protein in C.elegans.

(Information from UniProt)

Fibronectin

Best gene implication in ageing for this target family came from gene Q3UZF9 via mapping the annotation from Ensembl ENSMUSG00000026193 via mapping the annotation from EntrezGene 14268 via mapping the annotation from GenAgeModels 0818 annotated in GenAge release 17.

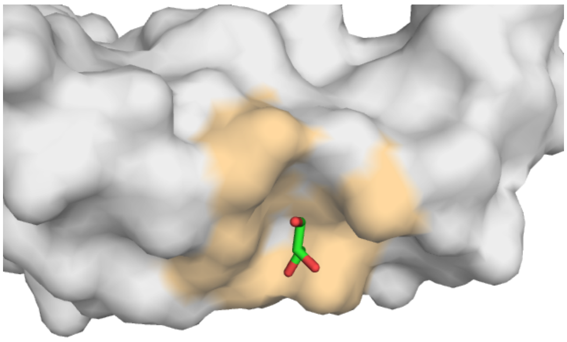

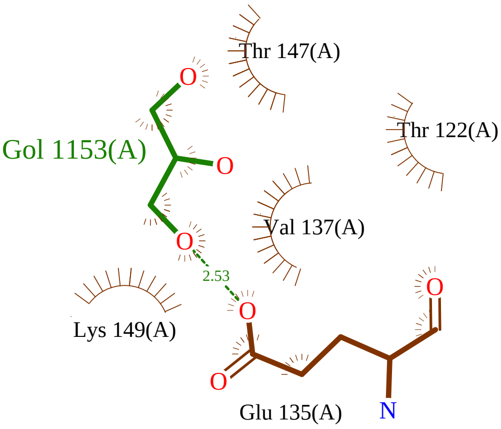

protein	amino acids contacts (binding site)
PDB:3zrz:chainA:P02751	- - - T E V T K
PDB:4mmz:chainC:P02751	T N E - - - - -
tr:H0Y7Z1:H0Y7Z1_HUMAN	K N Q K S T K D
tr:F8W7G7:F8W7G7_HUMAN	K N Q K S T K D
sp:P02751:FINC_HUMAN	K N Q K S T K D
tr:F1LST1:F1LST1_RAT	K N Q K S T K D
tr:Q3UZF9:Q3UZF9_MOUSE	K N Q K S T K D
sp:P11276:FINC_MOUSE	K N Q K S T K D
tr:H2KYR5:H2KYR5_CAEEL	N N D - - - - -
tr:Q18914:Q18914_CAEEL	N N D - - - - -

protein	whole protein ident	simil	domain-based ident	simil	contact-based ident	simil
PDB:3zrz:chainA:P02751	0.4	0.77	0.55	0.83	0.63	0.25
PDB:4mmz:chainC:P02751	0.77	0.89	0.48	0.76	0.38	0.0
tr:H0Y7Z1:H0Y7Z1_HUMAN	0.44	0.46	1.0	1.0	0.13	0.61
tr:F8W7G7:F8W7G7_HUMAN	0.93	0.93	1.0	1.0	0.13	0.61
sp:P02751:FINC_HUMAN	1.0	1.0	1.0	1.0	0.13	0.61
tr:F1LST1:F1LST1_RAT	0.65	0.69	0.97	0.99	0.13	0.61
tr:Q3UZF9:Q3UZF9_MOUSE	0.4	0.43	0.96	0.99	0.13	0.61
sp:P11276:FINC_MOUSE	0.88	0.94	0.96	0.99	0.13	0.61
tr:H2KYR5:H2KYR5_CAEEL	0.02	0.12	0.03	0.11	0.13	0.0
tr:Q18914:Q18914_CAEEL	0.02	0.12	0.03	0.11	0.13	0.0

ATP-dependent molecular chaperone HSP82

Best gene implication in ageing for this target family came from gene Q18688 annotated in UniProt release 2014_02. Annotation GO 8340 (determination of adult lifespan) was Inferred from Genetic Interaction

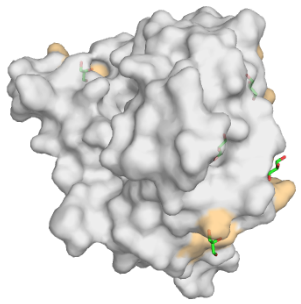

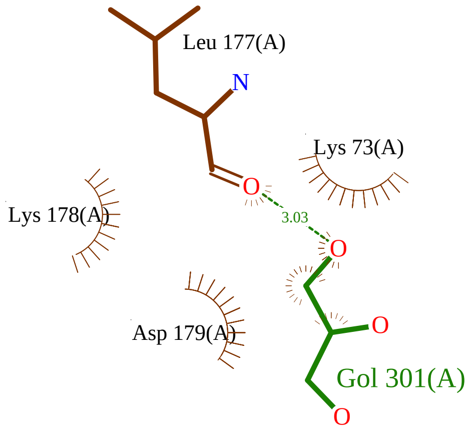

protein	amino acids contacts (binding site)
PDB:1ah8:chainA:P02829	S K R E S N D K Q T E R T P K E Q K R S G Q G F D N E S N S F T R G L R L K D
PDB:1usu:chainA:P02829	- -
PDB:2fxs:chainA:P02829	S K R E S N D K Q T E R T P K E Q K R S G Q G F D N E S N S F T R G L R L K D
PDB:2vwc:chainA:P02829	S K R E S N D K Q T E R T P K E Q K R S G Q G F D N E S N S F T R G L R L K D
PDB:2weq:chainA:P02829	S K R E S N D K Q T E R T P K E Q K R S G Q G F D N E S N S F T R G L R L K D
PDB:2xd6:chainA:P02829	S K R E S N D K Q T E R T P K E Q K R S G Q G F D N E S N S F T R G L R L K D
PDB:2xx2:chainA:P02829	S K R E S N D K Q T E R T P K E Q K R S G Q G F D N E S N S F T R G L R L K D
PDB:2xx5:chainA:P02829	S K R E S N D K Q T E R T P K E Q K R S G Q G F D N E S N S F T R G L R L K D
PDB:2yge:chainA:P02829	S K R E S N D K Q T E R T P K E Q K R S G Q G F D N E S N S F T R G L R L K D
PDB:2ygf:chainA:P02829	S K R E S N D K Q T E R T P K E Q K R S G Q G F D N E S N S F T R G L R L K D
PDB:3c11:chainA:P02829	S K R E S N D K Q T E R T P K E Q K R S G Q G F D N E S N S F T R G L R L K D
tr:K9JA46:K9JA46_HUMAN	S K R E S N D S K S G N I P N Q D R V S G Q G F E N E S S S F T P G V I L K E
sp:P07900:HS90A_HUMAN	S K R E S N D S K S G N I P N Q D R V S G Q G F E N E S S S F T P G V I L K E
sp:P82995:HS90A_RAT	S K R E S N D S K S G N I P N Q D R V S G Q G F E N E S S S F T P G V I L K E
tr:Q3TKB9:Q3TKB9_MOUSE	S K R E S N D S K S G N I P S Q D R V S G Q G F E N E S S S F T P G V I L K E
tr:Q3TKG0:Q3TKG0_MOUSE	S K R E S N D S K S G N I P S Q D R V S G Q G F E N E S S S F T P G V I L K E
sp:P07901:HS90A_MOUSE	S K R E S N D S K S G N I P S Q D R V S G Q G F E N E S S S F T P G V I L K E
tr:Q80Y52:Q80Y52_MOUSE	S K R E S N D S K S G N I P S Q D R V S G Q G F E N E S S S F T P G V I L K E
sp:P02828:HSP83_DROME	S K R E S N D S K S G K I P N T A G I S G Q G F D N E S S S F T P G I V I K E
sp:Q18688:HSP90_CAEEL	S K R E S N D S E T G K T P N E E K M S G Q G F D N E S S S F V E T I V I K E
sp:P02829:HSP82_YEAST	S K R E S N D K Q T E R T P K E Q K R S G Q G F D N E S N S F T R G L R L K D

protein	whole protein ident	simil	domain-based ident	simil	contact-based ident	simil
PDB:1ah8:chainA:P02829	1.0	1.0	1.0	1.0	0.92	0.84
PDB:1usu:chainA:P02829	0.99	1.0	0.99	1.0	0.08	0.0
PDB:2fxs:chainA:P02829	1.0	1.0	1.0	1.0	0.92	0.84
PDB:2vwc:chainA:P02829	1.0	1.0	1.0	1.0	0.92	0.84
PDB:2weq:chainA:P02829	0.99	1.0	0.99	1.0	0.92	0.84
PDB:2xd6:chainA:P02829	1.0	1.0	1.0	1.0	0.92	0.84
PDB:2xx2:chainA:P02829	1.0	1.0	1.0	1.0	0.92	0.84
PDB:2xx5:chainA:P02829	1.0	1.0	1.0	1.0	0.92	0.84
PDB:2yge:chainA:P02829	0.99	1.0	0.99	1.0	0.92	0.84
PDB:2ygf:chainA:P02829	0.99	1.0	0.99	1.0	0.92	0.84
PDB:3c11:chainA:P02829	0.99	1.0	0.99	1.0	0.92	0.84
tr:K9JA46:K9JA46_HUMAN	0.6	0.85	0.67	0.88	0.59	0.73
sp:P07900:HS90A_HUMAN	0.6	0.85	0.67	0.88	0.59	0.73
sp:P82995:HS90A_RAT	0.59	0.85	0.66	0.88	0.59	0.73
tr:Q3TKB9:Q3TKB9_MOUSE	0.48	0.65	0.66	0.88	0.59	0.73
tr:Q3TKG0:Q3TKG0_MOUSE	0.48	0.65	0.66	0.88	0.59	0.73
sp:P07901:HS90A_MOUSE	0.59	0.85	0.66	0.88	0.59	0.73
tr:Q80Y52:Q80Y52_MOUSE	0.59	0.85	0.66	0.88	0.59	0.73
sp:P02828:HSP83_DROME	0.6	0.86	0.67	0.9	0.55	0.71
sp:Q18688:HSP90_CAEEL	0.6	0.85	0.66	0.89	0.57	0.77
sp:P02829:HSP82_YEAST	1.0	1.0	1.0	1.0	1.0	1.0

Hsp83 (FBgn0001233) associated phenotypes
cell cycle defective, cell polarity defective, developmental rate defective, female semi-fertile, lethal - all die before end of pupal stage, lethal - all die during third instar larval stage, partially lethal, partially lethal - majority die, short lived

(Information from FlyBase)

Hsp83 (UniProt:P02828) annotation
Function: Molecular chaperone that promotes the maturation, structural maintenance and proper regulation of specific target proteins involved for instance in cell cycle control and signal transduction. Undergoes a functional cycle that is linked to its ATPase activity. This cycle probably induces conformational changes in the client proteins, thereby causing their activation. Interacts dynamically with various co-chaperones that modulate its substrate recognition, ATPase cycle and chaperone function. Together with Hop and piwi, mediates canalization, also known as developmental robustness, likely via epigenetic silencing of existing genetic variants and suppression of transposon-induced new genetic variation. Required for piRNA biogenesis by facilitating loading of piRNAs into PIWI proteins. (PubMed:21186352, PubMed:22902557).
Subunit: Homodimer (By similarity). Forms a complex with Hop and piwi; probably Hop mediates the interaction between piwi and Hsp83. Interacts with shu. (, PubMed:21186352, PubMed:22902557).
Subcellular location: Cytoplasm.
Induction: In contrast to other major heat shock proteins, this one is also expressed at normal growth temperatures. It is also developmentally expressed during oogenesis.
Domain: The TPR repeat-binding motif mediates interaction with TPR repeat-containing proteins.

(Information from UniProt)

daf-21 (WBGene00000915) associated phenotypes
chemotaxis variant, dauer constitutive, dauer recovery variant, larval lethal, lethal, odorant chemosensory response variant, sluggish, sterile

(Information from WormBase)

daf-21 (UniProt:Q18688) annotation
Function: Molecular chaperone that promotes the maturation, structural maintenance and proper regulation of specific target proteins involved for instance in cell cycle control and signal transduction. Undergoes a functional cycle that is linked to its ATPase activity. This cycle proba-

bly induces conformational changes in the client proteins, thereby causing their activation. Interacts dynamically with various co-chaperones that modulate its substrate recognition, ATPase cycle and chaperone function. By stabilizing the receptor-type guanylate cyclase daf-11 or another signal transduction component that regulates cGMP levels, plays a role in dauer formation and chemotaxis to non-volatile and volatile attractants detected by AWC sensory neurons (PubMed:10790386, PubMed:7828815). Participates in the control of cell cycle progression at the prophase/metaphase transition in oocyte development by ensuring the activity of wee-1.3 kinase, which negatively regulates cdk-1 through its phosphorylation (PubMed:16466390). Regulates yap-1 nuclear export after heat shock treatment (PubMed:23396260). (PubMed:10790386, PubMed:16466390, PubMed:23396260, PubMed:7828815).
Subunit: Homodimer. (UniProtKB:P08238).
Subcellular location: Cytoplasm, perinuclear region (PubMed:12950278). Note=Perinuclear region of somatic cells.
Tissue specificity: In the embryo comma stage, expression is strongly detected in cells of the head region and less so in other areas. In early larvae, expressed in postembryonic germ cells derived from Z2 and Z3 cells and the head region, in both hermaphrodites and males. Under heat stress conditions, larval expression is not only detected in germ cells, but also all over the body. In adult hermaphrodites, expression is localized uniquely in the germ cells. (PubMed:12950278).
Developmental stage: Highly expressed throughout development. (PubMed:10790386).
Domain: The TPR repeat-binding motif mediates interaction with TPR repeat-containing proteins.

(Information from UniProt)

Adenosine deaminase

Best gene implication in ageing for this target family came from gene Q920P6 annotated in UniProt release 2014_02. Annotation GO 7568 (aging) was Inferred from Expression Pattern

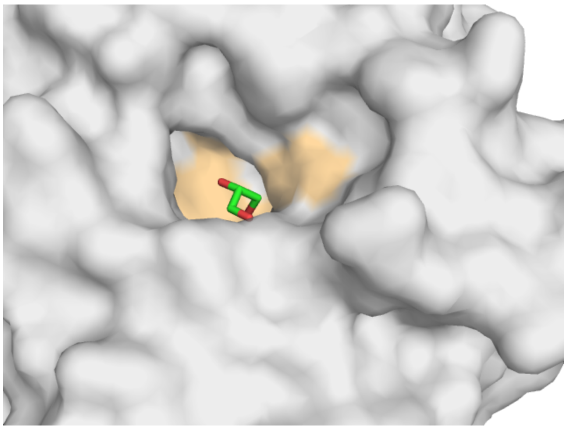

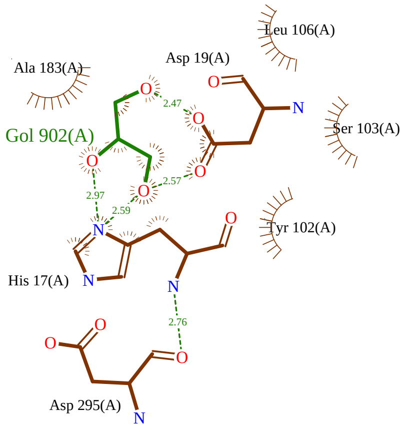

protein	amino acids contacts (binding site)
PDB:3mvi:chainA:P03958	H D F Y S L A
PDB:3mvt:chainA:P03958	H D F Y S L A
tr:F5GXW0:F5GXW0_HUMAN	H D F Y S L A
tr:F5GWI4:F5GWI4_HUMAN	H D F Y S L A
sp:P00813:ADA_HUMAN	H D F Y S L A
sp:Q920P6:ADA_RAT	H D F Y S L A
sp:P03958:ADA_MOUSE	H D F Y S L A
tr:Q4FK28:Q4FK28_MOUSE	H D F Y S L A
tr:Q86NI2:Q86NI2_CAEEL	H D F Y S L A
tr:H2KYI5:H2KYI5_CAEEL	H D F Y S L A
sp:P53909:ADE_YEAST	H E - Y D - D

protein	whole protein ident	simil	domain-based ident	simil	contact-based ident	simil
PDB:3mvi:chainA:P03958	1.0	1.0	1.0	1.0	1.0	1.0
PDB:3mvt:chainA:P03958	1.0	1.0	1.0	1.0	1.0	1.0
tr:F5GXW0:F5GXW0_HUMAN	0.61	0.71	0.61	0.71	1.0	1.0
tr:F5GWI4:F5GWI4_HUMAN	0.75	0.87	0.77	0.89	1.0	1.0
sp:P00813:ADA_HUMAN	0.81	0.93	0.83	0.96	1.0	1.0
sp:Q920P6:ADA_RAT	0.94	0.99	0.94	0.99	1.0	1.0
sp:P03958:ADA_MOUSE	1.0	1.0	1.0	1.0	1.0	1.0
tr:Q4FK28:Q4FK28_MOUSE	1.0	1.0	1.0	1.0	1.0	1.0
tr:Q86NI2:Q86NI2_CAEEL	0.39	0.76	0.4	0.78	1.0	1.0
tr:H2KYI5:H2KYI5_CAEEL	0.35	0.69	0.4	0.78	1.0	1.0
sp:P53909:ADE_YEAST	0.26	0.68	0.27	0.68	0.29	0.14

High affinity nerve growth factor receptor

Best gene implication in ageing for this target family came from gene P35739 annotated in UniProt release 2014_02. Annotation GO 7568 (aging) was Inferred from Expression Pattern

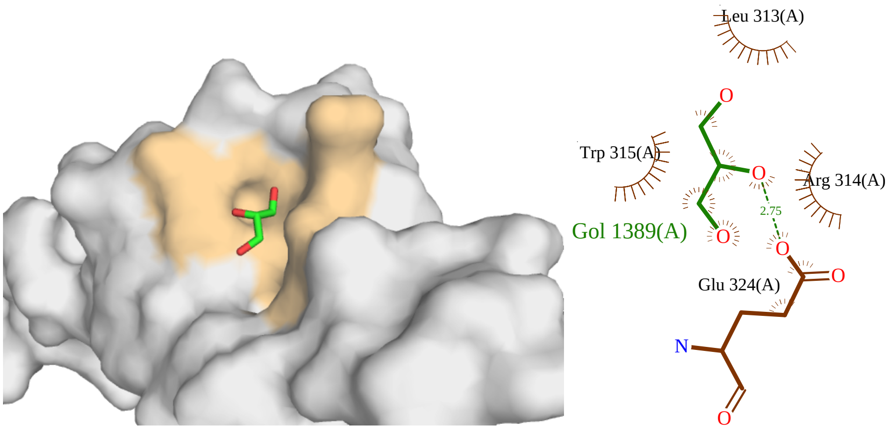

protein	amino acids contacts (binding site)
PDB:1he7:chainA:P04629	L R W L E - -
PDB:4pmm:chainA:P04629	- - - - - P E
sp:P04629:NTRK1_HUMAN	L R W L E P E
tr:J3KP20:J3KP20_HUMAN	L R W L E P E
sp:P35739:NTRK1_RAT	L R W L E P D
sp:Q3UFB7:NTRK1_MOUSE	L R W L E P D
tr:G4S8E3:G4S8E3_CAEEL	I - - - - Q D
tr:G4RZJ9:G4RZJ9_CAEEL	I - - - - Q D

protein	whole protein ident	simil	domain-based ident	simil	contact-based ident	simil
PDB:1he7:chainA:P04629	0.98	0.99	1.0	1.0	0.71	0.33
PDB:4pmm:chainA:P04629	0.98	0.99	1.0	1.0	0.29	0.0
sp:P04629:NTRK1_HUMAN	1.0	1.0	1.0	1.0	1.0	1.0
tr:J3KP20:J3KP20_HUMAN	0.99	0.99	1.0	1.0	1.0	1.0
sp:P35739:NTRK1_RAT	0.86	0.95	0.95	0.98	0.86	0.93
sp:Q3UFB7:NTRK1_MOUSE	0.87	0.95	0.95	0.98	0.86	0.93
tr:G4S8E3:G4S8E3_CAEEL	0.11	0.4	0.14	0.37	0.0	0.0
tr:G4RZJ9:G4RZJ9_CAEEL	0.16	0.49	0.29	0.66	0.0	0.0

Myeloperoxidase

Best gene implication in ageing for this target family came from gene D3ZGE2 annotated in UniProt release 2014_02. Annotation GO 7568 (aging) was Inferred from Expression Pattern

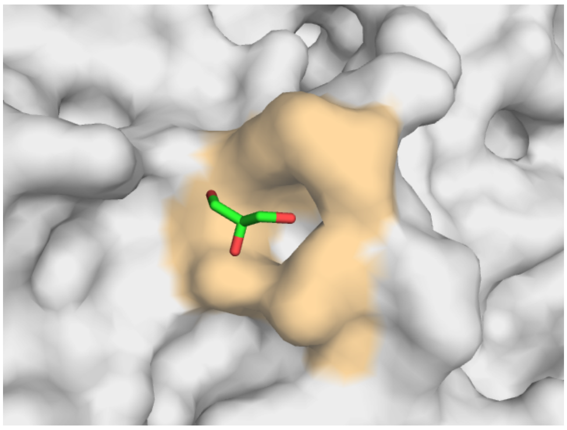

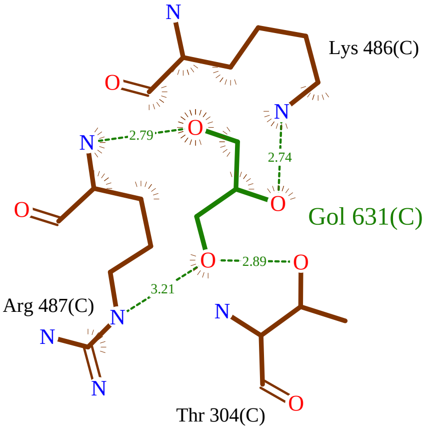

protein	amino acids contacts (binding site)
PDB:4c1m:chainC:P05164	T K R
sp:P05164:PERM_HUMAN	T K R
tr:D3ZGE2:D3ZGE2_RAT	A E P
tr:D3ZYH8:D3ZYH8_RAT	A E P
sp:P11247:PERM_MOUSE	A E P
tr:Q8MT50:Q8MT50_DROME	E L P
sp:Q01603:PERO_DROME	E V A
tr:E1JIN0:E1JIN0_DROME	E V A
tr:Q9VEJ9:Q9VEJ9_DROME	E L P
sp:Q9VEG6:PERC_DROME	Q V E
sp:Q23490:MLT7_CAEEL	S V I
tr:O01892:O01892_CAEEL	K L A
tr:O17241:O17241_CAEEL	K I P

	whole protein		domain-based		contact-based	
protein	ident	simil	ident	simil	ident	simil
PDB:4c1m:chainC:P05164	1.0	1.0	1.0	1.0	1.0	1.0
sp:P05164:PERM_HUMAN	1.0	1.0	1.0	1.0	1.0	1.0
tr:D3ZGE2:D3ZGE2_RAT	0.54	0.59	0.53	0.57	0.0	0.52
tr:D3ZYH8:D3ZYH8_RAT	0.61	0.68	0.79	0.87	0.0	0.52
sp:P11247:PERM_MOUSE	0.83	0.93	0.87	0.96	0.0	0.52
tr:Q8MT50:Q8MT50_DROME	0.21	0.49	0.28	0.63	0.0	0.5
sp:Q01603:PERO_DROME	0.23	0.59	0.29	0.7	0.0	0.48
tr:E1JIN0:E1JIN0_DROME	0.23	0.59	0.29	0.7	0.0	0.48
tr:Q9VEJ9:Q9VEJ9_DROME	0.25	0.6	0.32	0.71	0.0	0.5
sp:Q9VEG6:PERC_DROME	0.22	0.55	0.29	0.67	0.0	0.55
sp:Q23490:MLT7_CAEEL	0.25	0.59	0.34	0.73	0.0	0.55
tr:O01892:O01892_CAEEL	0.21	0.56	0.29	0.68	0.0	0.48
tr:O17241:O17241_CAEEL	0.16	0.41	0.29	0.68	0.0	0.52

Pxd (FBgn0004577) associated phenotypes

chemical conditional, chemical sensitive, neuroanatomy defective, partially lethal, partially lethal - majority die, some die during pupal stage

(Information from FlyBase)

Pxd (UniProt:Q01603) annotation

Function: Involved in the chorion hardening process, through protein cross-linking mediated by the formation of di- and tri- tyrosine bonds. (PubMed:15979004).
Cofactor: heme bNote=Binds 1 heme b (iron(II)-protoporphyrin IX) group per subunit. ;
Subcellular location: Secreted

(Information from UniProt)

Pxt (FBgn0261987) associated phenotypes

female semi-sterile

(Information from FlyBase)

Pxt (UniProt:Q9VEG6) annotation

Function: Required for ovarian follicle maturation. Involved in the formation of a rigid and insoluble egg chorion by catalyzing chorion protein cross-linking through dityrosine formation and phenol oxidase-catalyzed chorion melanization. (PubMed:18216169).
Cofactor: heme bNote=Binds 1 heme b (iron(II)-protoporphyrin IX) group per heterodimer. ;
Subunit: Heterodimer. (UniProtKB:P05164,).
Subcellular location: Secreted (PubMed:18216169). Note=In the chorion layer of the mature eggs.
Tissue specificity: Expressed at low levels in the germarium and early follicles. Expression becomes progressively stronger during vitellogenesis, and is highly expressed in germ cells and somatic cells. A subset of follicle cells, termed border cells (BC), exhibit a high level of expression. (PubMed:18216169).
Developmental stage: Expressed both maternally and zygotically. (PubMed:12459925).
Disruption phenotype: Females are sterile, and maturing follicles show defects in actin filament formation, nurse cell membrane stability and border cell migration. (PubMed:18216169).

(Information from UniProt)

mlt-7 (WBGene00022743) associated phenotypes

dumpy, lethal, sterile

(Information from WormBase)

mlt-7 (UniProt:Q23490) annotation

Function: Plays an essential role in cuticle biogenesis. Required in combination with bli-3 for correct formation of cross-links in cuticle collagens. (PubMed:19406744).
Tissue specificity: Expressed in the hypodermal cells, specifically the head and seam/body. (PubMed:19406744).
Developmental stage: Expressed in all adult and larval stages. Expression is cyclical and coincides with each of the larval molts, peaking at 12, 18, 24, and 30h post-hatching. (PubMed:19406744).
Disruption phenotype: Gross morphological abnormalities causing larval arrest with associated molt defects and low levels of embryonic lethality. Cuticle function and integrity are also impaired. (PubMed:19406744).

(Information from UniProt)

Integrin beta-1

Best gene implication in ageing for this target family came from gene P11584 annotated in UniProt release 2014_02. Annotation GO 8340 (determination of adult lifespan) was Inferred from Mutant Phenotype

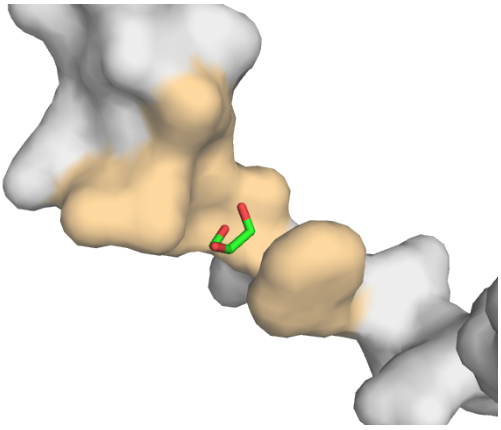

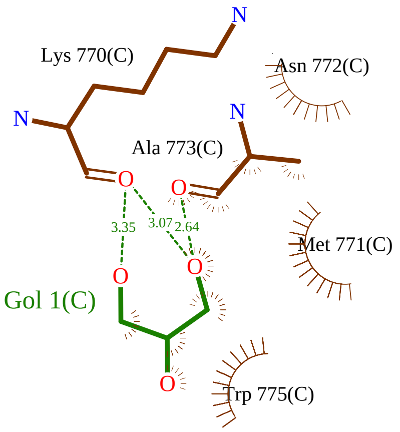

protein	amino acids contacts (binding site)
PDB:3g9w:chainC:P05556	K M N A W
sp:P05556:ITB1_HUMAN	K M N A W
sp:P49134:ITB1_RAT	K M N A W
sp:P09055:ITB1_MOUSE	K M N A W
sp:P11584:ITBX_DROME	R M N A W
sp:Q27874:PAT3_CAEEL	R L M A W

	whole protein		domain-based		contact-based	
protein	ident	simil	ident	simil	ident	simil
PDB:3g9w:chainC:P05556	0.9	0.98	0.87	0.97	1.0	1.0
sp:P05556:ITB1_HUMAN	1.0	1.0	1.0	1.0	1.0	1.0
sp:P49134:ITB1_RAT	0.92	0.98	1.0	1.0	1.0	1.0
sp:P09055:ITB1_MOUSE	0.92	0.98	1.0	1.0	1.0	1.0
sp:P11584:ITBX_DROME	0.4	0.72	0.79	0.94	0.8	0.94
sp:Q27874:PAT3_CAEEL	0.38	0.74	0.63	0.88	0.4	0.61

mys (FBgn0004657) associated phenotypes

cell shape defective, flightless, germline clone, heat sensitive, lethal - all die before end of first instar larval stage, lethal - all die before end of pupal stage, neuroanatomy defective, neurophysiology defective, partially, partially lethal - majority die, reduced, rescuable maternal effect, smell perception defective, somatic clone, some die during pupal stage

(Information from FlyBase)

mys (UniProt:P11584) annotation

Function: Integrin alpha-PS1/beta-PS is a receptor for laminin. Integrin alpha-PS2/beta-PS is a receptor for Tig, wb and Ten-m. Contributes to endodermal integrity and adhesion between the midgut epithelium and the surrounding visceral muscle. Essential for migration of the primordial midgut cells and for maintaining, but not establishing, cell polarity in the midgut epithelium. The two beta subunits mediate midgut migration by distinct mechanisms: beta-PS requires rhea/talin and Itgbetanu does not. Required for rhea/talin correct cellular localization in the midgut. Required for many embryonic (dorsal closure and somatic muscle attachments) and post-embryonic developmental processes (attachment between cell layers of imaginal disks, organization of ommatidial arrays and flight muscle development). Involved in the function and/or development of the olfactory system. Plays a role in timely border cell migration during oogenesis. (PubMed:10821184, PubMed:15469969, PubMed:19035354, PubMed:7924982, PubMed:7972082, PubMed:8119134, PubMed:9660786).
Subunit: Heterodimer of an alpha and a beta subunit. Beta-PS associates with either alpha-PS1, alpha-PS2, alpha-PS3, alpha-PS4 or alpha-PS5.
Subcellular location: Apical cell membrane (PubMed:19035354); Single-pass type I membrane protein (PubMed:19035354). Lateral cell membrane (PubMed:19035354); Single-pass type I membrane protein (PubMed:19035354). Basal cell membrane (PubMed:19035354); Single-pass type I membrane protein (PubMed:19035354). Note=In ovary, localizes to the apical, lateral and basal

membranes of follicle cells through oogenesis stage 10A. Apical membrane expression peaks at oogenesis stages 9 and 10A in columnar follicle cells overlying the oocyte but decreases in the most posterior follicle cells. Thereafter, it is down-regulated. Localization to lateral and basal membranes persists during dorsal appendage morphogenesis.
Tissue specificity: In ovaries, strongly expressed in follicle cells. In oocytes, expressed in the forming dorsal appendages (at protein level). Expressed in the embryonic dorsal cuticle, the larval eye and the wing imaginal disk. (PubMed:19035354, PubMed:8119134).
Disruption phenotype: In zygotic mutant embryos, midgut forms primary constrictions but fails to elongate and the visceral muscle does not flatten but remains attached to the midgut epithelium. Embryos lacking maternal and zygotic mys show a delay in midgut migration. Mutant larvae present an olfatory phenotype, showing reduced response to isoamyl acetate but normal response to ethyl acetate. (PubMed:10821184, PubMed:15469969).
Miscellaneous: The absence of the beta-PS subunit results in detachment and rounding up of the muscles, thus the gene encoding beta-PS is called myospheroid.

(Information from UniProt)

pat-3 (WBGene00003930) associated phenotypes
body wall muscle actin organization defective, body wall muscle cell polarization variant, body wall muscle myosin organization defective, cell migration variant, embryonic lethal, excretory canal short, paralyzed arrested elongation two fold, protein expression absent, sterile

(Information from WormBase)

pat-3 (UniProt:Q27874) annotation
Function: Possible role in cell-cell interactions. Integrin alpha pat-1/beta pat-3 is a receptor for laminin. Integrin alpha pat- 2/beta pat-3 recognizes the sequence R-G-D in its ligands (By similarity). During gonad morphogenesis, involved in distal tip cell (DTC)-mediated guidance of gonad elongation, in maintaining their sharp tapering morphology and in their migration (PubMed:19023419). (, PubMed:19023419).
Subunit: Heterodimer of an alpha and a beta subunit. Beta pat-3 associates with either alpha pat-1 or alpha pat-2.
Subcellular location: Membrane ; Single-pass type I membrane protein
Disruption phenotype: RNAi-mediated knockdown in distal tip cell (DTC) causes DTC migration and guidance defects during the second phase of gonad elongation resulting in a triangular shaped gonad. (PubMed:19023419).

(Information from UniProt)

Insulin receptor

Best gene implication in ageing for this target family came from gene P09208 annotated in UniProt release 2014_02. Annotation GO 8340 (determination of adult lifespan) was Inferred from Mutant Phenotype

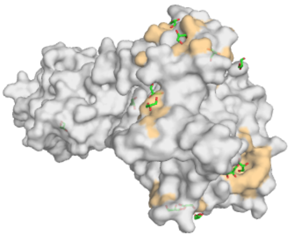

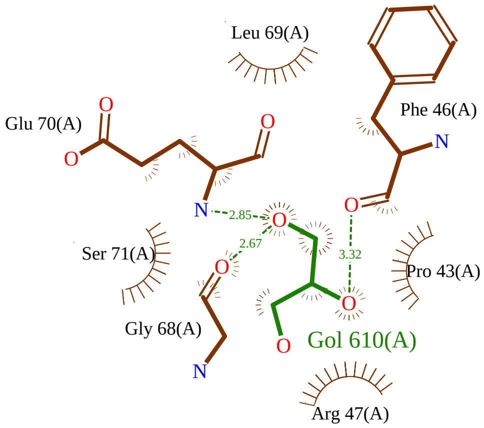

protein	amino acids contacts (binding site)
PDB:2hr7:chainA:P06213	P F R R G L E S D L F E V R K N S R L N K D N T R K S L C E S D P T K C V Y G
sp:P06213:INSR_HUMAN	P F R R G L E S D L F E V R K N S R L N K D N T R K S L C E S D P T K C V Y G
tr:F1LN53:F1LN53_RAT	- - - - - - - - - - - - - R K N S R L N K D N T R K S L C E S D P T K C V Y G
tr:F1LPL6:F1LPL6_RAT	- - - - - - - - - - - - - R K N S R L N K D N T R K S L C E S D P T K C V Y G
sp:P15208:INSR_MOUSE	P F R R G L E S D L F E V R K N S R L N K D N T R K S L C E S D P T K C V Y G
tr:M4TKR7:M4TKR7_MOUSE	P F R R G L E S D L F E V R K N S R L N K D N T R K S L C E S D P T K C V Y G
tr:D0UY96:D0UY96_DROME	P - L R G L H S K L D S F R K H L E L T E G K T S - R T C D V K N E S C I S N
tr:D0UY86:D0UY86_DROME	P - L R G L H S K L D S F R K H L E L T E G K T S - R T C D V K N E S C I S N
tr:D0UY84:D0UY84_DROME	P - L R G L H S K L D S F R K H L E L T E G K T S - R T C D V K N E S C I S N
tr:D0UY85:D0UY85_DROME	P - L R G L H S K L D S F R K H L E L T E G K T S - R T C D V K N E S C I S N
tr:D0UY80:D0UY80_DROME	P - L R G L H S K L D S F R K H L E L T E G K T S - R T C D V K N E S C I S N
tr:D0UY81:D0UY81_DROME	P - L R G L H S K L D S F R K H L E L T E G K T S - R T C D V K N E S C I S N
tr:D0UY88:D0UY88_DROME	P - L R G L H S K L D S F R K H L E L T E G K T S - R T C D V K N E S C I S N
tr:D0UY89:D0UY89_DROME	P - L R G L H S K L D S F R K H L E L T E G K T S - R T C D V K N E S C I S N
tr:D0UYA1:D0UYA1_DROME	P - L R G L H S K L D S F R K H L E L T E G K T S - R T C D V K N E S C I S N
tr:D0UYA0:D0UYA0_DROME	P - L R G L H S K L D S F R K H L E L T E G K T S - R T C D V K N E S C I S N
tr:D0UYA6:D0UYA6_DROME	P - L R G L H S K L D S F R K H L E L T E G K T S - R T C D V K N E S C I S N
tr:D0UY91:D0UY91_DROME	P - L R G L H S K L D S F R K H L E L T E G K T S - R T C D V K N E S C I S N
tr:D0UY97:D0UY97_DROME	P - L R G L H S K L D S F R K H L E L T E G K P S - R T C D V K N E S C I S N
tr:D0UY78:D0UY78_DROME	P - L R G L H S K L D S F R K H L E L T E G K T S - R T C D V K N E S C I S N
tr:D0UY95:D0UY95_DROME	P - L R G L H S K L D S F R K H L E L T E G K T S - R T C D V K N E S C T S N
tr:D0UY94:D0UY94_DROME	P - L R G L H S K L D S F R K H L E L T E G K T S - R T C D V K N E S C I S N
tr:D0UY75:D0UY75_DROME	P - L R G L H S K L D S F R K H L E L T E G K T S - R T C D V K N E S C I S N
tr:D0UY77:D0UY77_DROME	P - L R G L H S K L D S F R K H L E L T E G K T S - R T C D V K N E S C I S N
tr:D0UY70:D0UY70_DROME	P - L R G L H S K L D S F R K H L E L T E G K T S - R T C D V K N E S C I S N
tr:D0UYA5:D0UYA5_DROME	P - L R G L H S K L D S F R K H L E L T E G K T S - R T C D V K N E S C I S N
tr:D0UYA9:D0UYA9_DROME	P - L R G L H S K L D S F R K H L E L T E G K T S - R T C D V K N E S C I S N
tr:D0UY99:D0UY99_DROME	P - L R G L H S K L D S F R K H L E L T E G K T S - R T C D V K N E S C I S N
tr:D0UYA4:D0UYA4_DROME	P - L R G L H S K L D S F R K H L E L T E G K T S - R T C D V K N E S C I S N
tr:D0UYA8:D0UYA8_DROME	P - L R G L H S K L D S F R K H L E L T E G K T S - R T C D V K N E S C I S N
tr:D0UY98:D0UY98_DROME	P - L R G L H S K L D S F R K H L E L T E G K T S - R T C D V K N E S C I S N
tr:D0UYA7:D0UYA7_DROME	P - L R G L H S K L D S F R K H L E L T E G K T S - R T C D V K N E S C I S N
tr:C7C412:C7C412_DROME	P - L R G L H S K L D S F R K H L E L T E G K T S - R T C D V K N E S C I S N
tr:C7C413:C7C413_DROME	P - L R G L H S K L D S F R K H L E L T E G K T S - R T C D V K N E S C I S N
tr:C7C410:C7C410_DROME	P - L R G L H S K L D S F R K H L E L T E G K T S - R T C D V K N E S C I S N
tr:C7C411:C7C411_DROME	P - L R G L H S K L D S F R K H L E L T E G K T S - R T C D V K N E S C I S N
tr:C7C414:C7C414_DROME	P - L R G L H S K L D S F R K H L E L T E G K T S - R T C D V K N E S C I S N
tr:C7C415:C7C415_DROME	P - L R G L H S K L D S F R K H L E L T E G K T S - R T C D V K N E S C I S N
tr:C7C417:C7C417_DROME	P - L R G L H S K L D S F R K H L E L T E G K T S - R T C D V K N E S C I S N
sp:P09208:INSR_DROME	P - L R G L H S K L D S F R K H L E L T E G K T S - R T C D V K N E S C I S N
tr:C7C416:C7C416_DROME	P - L R G L H S K L D S F R K H L E L T E G K T S - R T C D V K N E S C I S N
sp:Q968Y9:INSR_CAEEL	T Q E E G L V D K L Q R P N D R K H D N A E Y K Q I G D R Q E N A T A C H Y G

protein	whole protein ident	simil	domain-based ident	simil	contact-based ident	simil
PDB:2hr7:chainA:P06213	1.0	1.0	1.0	1.0	1.0	1.0
sp:P06213:INSR_HUMAN	1.0	1.0	1.0	1.0	1.0	1.0
tr:F1LN53:F1LN53_RAT	0.85	0.88	0.73	0.75	0.72	0.43
tr:F1LPL6:F1LPL6_RAT	0.86	0.89	0.73	0.75	0.72	0.43
sp:P15208:INSR_MOUSE	0.94	0.98	0.98	1.0	1.0	1.0
tr:M4TKR7:M4TKR7_MOUSE	0.95	0.98	0.98	1.0	1.0	1.0
tr:D0UY96:D0UY96_DROME	0.22	0.49	0.38	0.74	0.3	0.49
tr:D0UY86:D0UY86_DROME	0.22	0.48	0.38	0.74	0.3	0.49
tr:D0UY84:D0UY84_DROME	0.22	0.49	0.38	0.74	0.3	0.49
tr:D0UY85:D0UY85_DROME	0.22	0.48	0.38	0.74	0.3	0.49
tr:D0UY80:D0UY80_DROME	0.22	0.49	0.38	0.74	0.3	0.49
tr:D0UY81:D0UY81_DROME	0.22	0.49	0.38	0.74	0.3	0.49
tr:D0UY88:D0UY88_DROME	0.22	0.49	0.38	0.74	0.3	0.49
tr:D0UY89:D0UY89_DROME	0.22	0.49	0.38	0.74	0.3	0.49
tr:D0UYA1:D0UYA1_DROME	0.22	0.49	0.38	0.74	0.3	0.49
tr:D0UYA0:D0UYA0_DROME	0.22	0.49	0.38	0.74	0.3	0.49
tr:D0UYA6:D0UYA6_DROME	0.22	0.49	0.38	0.74	0.3	0.49
tr:D0UY91:D0UY91_DROME	0.22	0.49	0.38	0.74	0.3	0.49
tr:D0UY97:D0UY97_DROME	0.22	0.48	0.38	0.74	0.28	0.49
tr:D0UY78:D0UY78_DROME	0.22	0.49	0.38	0.74	0.3	0.49
tr:D0UY95:D0UY95_DROME	0.22	0.49	0.38	0.74	0.3	0.49
tr:D0UY94:D0UY94_DROME	0.22	0.49	0.38	0.74	0.3	0.49
tr:D0UY75:D0UY75_DROME	0.22	0.49	0.38	0.74	0.3	0.49
tr:D0UY77:D0UY77_DROME	0.22	0.49	0.38	0.74	0.3	0.49
tr:D0UY70:D0UY70_DROME	0.22	0.48	0.38	0.74	0.3	0.49
tr:D0UYA5:D0UYA5_DROME	0.22	0.49	0.38	0.74	0.3	0.49
tr:D0UYA9:D0UYA9_DROME	0.22	0.48	0.38	0.74	0.3	0.49
tr:D0UY99:D0UY99_DROME	0.22	0.48	0.38	0.74	0.3	0.49
tr:D0UYA4:D0UYA4_DROME	0.22	0.48	0.38	0.74	0.3	0.49
tr:D0UYA8:D0UYA8_DROME	0.22	0.48	0.38	0.74	0.3	0.49
tr:D0UY98:D0UY98_DROME	0.22	0.48	0.38	0.74	0.3	0.49
tr:D0UYA7:D0UYA7_DROME	0.22	0.48	0.38	0.74	0.3	0.49
tr:C7C412:C7C412_DROME	0.21	0.46	0.38	0.74	0.3	0.49
tr:C7C413:C7C413_DROME	0.21	0.46	0.38	0.74	0.3	0.49
tr:C7C410:C7C410_DROME	0.21	0.46	0.38	0.74	0.3	0.49
tr:C7C411:C7C411_DROME	0.21	0.46	0.38	0.74	0.3	0.49
tr:C7C414:C7C414_DROME	0.21	0.46	0.38	0.74	0.3	0.49
tr:C7C415:C7C415_DROME	0.21	0.46	0.38	0.74	0.3	0.49
tr:C7C417:C7C417_DROME	0.21	0.46	0.38	0.74	0.3	0.49
sp:P09208:INSR_DROME	0.21	0.46	0.38	0.74	0.3	0.49
tr:C7C416:C7C416_DROME	0.21	0.46	0.38	0.74	0.3	0.49
sp:Q968Y9:INSR_CAEEL	0.2	0.52	0.32	0.69	0.23	0.49

InR (FBgn0013984) associated phenotypes

RU486 conditional, aging defective, body size defective, cell autonomous, cell number defective, cell size defective, chemical sensitive, circadian rhythm defective, conditional, decreased cell number, decreased cell size, developmental rate defective, dominant, feeding behavior defective, heat sensitive, hyperplasia, increased cell size, lethal - all die before end of second instar larval stage, long lived, mating defective, melanotic mass phenotype, neuroanatomy defective, neurophysiology defective, oxidative stress response defective, partially, partially lethal, partially lethal - majority die, short lived, size defective, sleep defective, small body, somatic clone, some die during pupal stage, some die during second instar larval stage, starvation stress response defective, stress response defective

(Information from FlyBase)

InR (UniProt:P09208) annotation

Function: Has a ligand-stimulated tyrosine-protein kinase activity. Required for cell survival. Regulates body size and organ size by altering cell number and cell size in a cell- autonomous manner. Involved in the development of the embryonic nervous system, and is necessary for axon guidance and targeting in the visual system. Also plays a role in life-span determination. (PubMed:10455177, PubMed:11250149, PubMed:11292875, PubMed:12702880, PubMed:7628438, PubMed:8603594).

Cofactor: Mn(2+)
Enzyme regulation: Autophosphorylation activates the kinase activity.
Subunit: Tetramer of 2 alpha and 2 beta chains linked by disulfide bonds. The alpha chains contribute to the formation of the ligand- binding domain, while the beta chains carry the kinase domain. When autophosphorylated, the beta-subunit binds the SH2 and SH3 domains of the adapter protein Dock. The beta subunit also binds and tyrosine phosphorylates the insulin receptor substrate Chico.
Subcellular location: Membrane; Single-pass type I membrane protein.
Tissue specificity: Widely distributed throughout the embryo. Expressed at high levels in the embryonic nervous system. Larval expression is limited to the nervous system and all imaginal disks. Expressed at high levels in the adult nervous system and ovaries. (PubMed:2454394, PubMed:7628438).
Developmental stage: Expressed throughout development. Embryonic expression is most prominent 8 to 12 hours after egg laying. (PubMed:2454394, PubMed:3014506).
Ptm: The 280 kDa proreceptor is proteolytically processed to form a 120 kDa alpha subunit and a 170 kDa beta subunit. The beta subunit undergoes cell-specific cleavage to generate a 90 kDa beta subunit and a free 60 kDa C-terminal subunit. Both the 90 kDa and the 170 kDa beta subunits can assemble with the alpha subunits to form mature receptors. (PubMed:7628438).
Ptm: Autophosphorylated on tyrosine residues in response to exogenous insulin. (PubMed:18327897).
Ptm: Phosphorylation of Tyr-1354 is required for Chico-binding.

(Information from UniProt)

daf-2 (WBGene00000898) associated phenotypes

DMPP resistant, L2 larval development variant, aging variant, anoxia resistant, autophagy variant, brood size variant, carbohydrate metabolism variant, carbon dioxide avoidance variant, dauer arrest variant, dauer constitutive, dauer formation variant, diet induced life span variant, drug response variant, egg laying variant, extended life span, fat content increased, gene expression level high, gene expression variant, hormetic temperature induced life span extension defective, larval lethal, lethal, lipid composition variant, lipid metabolism variant, lipid synthesis increased, mRNA expression variant, mRNA levels increased, mate searching variant, mianserin resistant, no dauer recovery, organ senescence variant, organism UV resistant, organism dessication response variant, organism hypersensitive dessication, organism oxidative stress response variant, organism physiology variant, organism starvation response variant, pathogen resistance increased, protein expression increased, protein phosphorylation increased, protein phosphorylation reduced, quiescence variant, reduced brood size, reproductive longevity extended, response to food variant, roaming reduced, spontaneous spicule protraction, sterile, temperature influenced life span variant, thermotolerance increased, transgene expression increased, transgene expression reduced, transgene subcellular localization variant, yolk synthesis variant

(Information from WormBase)

daf-2 (UniProt:Q968Y9) annotation

Function: An insulin receptor-like protein which regulates metabolism, controls longevity and prevents developmental arrest at the dauer stage. Binding of INS family members may either stimulate, or antagonize, association of the receptor with downstream mediators such as pdk-1 and age-1. Required for the response to environmental stimuli such as food, pheromone, and temperature. Role in immune function and pathogen resistance. (PubMed:11274053, PubMed:18782349, PubMed:9252323, PubMed:9790527).
Cofactor: Mn(2+) Evidence=(UniProtKB:P06213);
Enzyme regulation: Autophosphorylation activates the kinase activity (By similarity). Interaction with shc-1 may inhibits its activity (PubMed:18832074). (UniProtKB:P06213, PubMed:18832074).
Subunit: Tetramer of 2 alpha and 2 beta chains linked by disulfide bonds. The alpha chains contribute to the formation of the ligand- binding domain, while the beta chains carry the kinase domain

(By similarity). Interacts (via cytoplasmic domain) with shc-1 (PID domain) (PubMed:18832074). (UniProtKB:P06213, PubMed:18832074).
Subcellular location: Membrane (UniProtKB:P06213); Single-pass type I membrane protein (UniProtKB:P06213).
Disruption phenotype: Accumulation of fat, pigmented intestine, increased life span, increased dauer formation and increased resistance to pathogens. Severe loss of function mutants display recessive early embryonic lethality. (PubMed:11274053, PubMed:18245374, PubMed:18782349, PubMed:9252323, PubMed:9790527).

(Information from UniProt)

Thymidylate synthase

Best gene implication in ageing for this target family came from gene P45352 annotated in UniProt release 2014_02. Annotation GO 7568 (aging) was Inferred from Expression Pattern

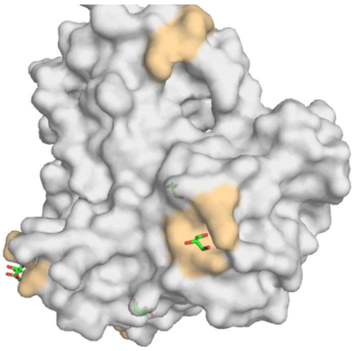

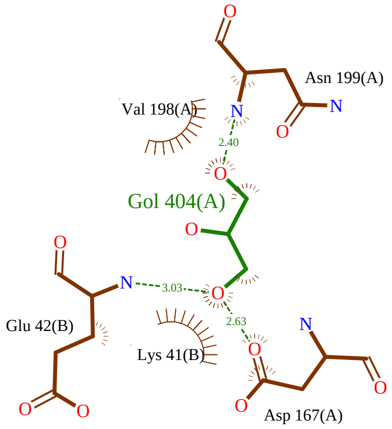

protein	amino acids contacts (binding site)
PDB:4eb4:chainA:P07607	K E R W F G D N K V N R K I Y
PDB:4ez8:chainA:P07607	K E R W F G D N K V N R K I Y
tr:Q53Y97:Q53Y97_HUMAN	K D R W F G D N R V N R K I Y
sp:P04818:TYSY_HUMAN	K D R W F G D N R V N R K I Y
sp:P45352:TYSY_RAT	K E R W F G D N K V N R K I Y
sp:P07607:TYSY_MOUSE	K E R W F G D N K V N R K I Y
tr:Q544L2:Q544L2_MOUSE	K E R W F G D N K V N R K I Y
tr:Q541C9:Q541C9_DROME	R M R F F G S N L S E R Q I Y
sp:O76511:TYSY_DROME	R M R F F G S N L S E R Q I Y
tr:Q9Y052:Q9Y052_CAEEL	R D R W F G D N S D N R D L Y
tr:Q9N588:Q9N588_CAEEL	R D R W F G D N S D N R D L Y
sp:P06785:TYSY_YEAST	R P K T F G Y N A S S R D I Y

	whole protein		domain-based		contact-based	
protein	ident	simil	ident	simil	ident	simil
PDB:4eb4:chainA:P07607	1.0	1.0	1.0	1.0	1.0	1.0
PDB:4ez8:chainA:P07607	1.0	1.0	1.0	1.0	1.0	1.0
tr:Q53Y97:Q53Y97_HUMAN	0.88	0.95	0.91	0.96	0.87	0.95
sp:P04818:TYSY_HUMAN	0.88	0.95	0.91	0.96	0.87	0.95
sp:P45352:TYSY_RAT	0.97	0.99	0.98	1.0	1.0	1.0
sp:P07607:TYSY_MOUSE	1.0	1.0	1.0	1.0	1.0	1.0
tr:Q544L2:Q544L2_MOUSE	1.0	1.0	1.0	1.0	1.0	1.0
tr:Q541C9:Q541C9_DROME	0.62	0.84	0.65	0.86	0.47	0.63
sp:O76511:TYSY_DROME	0.62	0.84	0.65	0.86	0.47	0.63
tr:Q9Y052:Q9Y052_CAEEL	0.6	0.81	0.63	0.85	0.6	0.7
tr:Q9N588:Q9N588_CAEEL	0.59	0.8	0.63	0.85	0.6	0.7
sp:P06785:TYSY_YEAST	0.58	0.79	0.61	0.83	0.4	0.5

Ts (FBgn0024920) associated phenotypes
neuroanatomy defective

(Information from FlyBase)

Ts (UniProt:O76511) annotation
Pathway: Pyrimidine metabolism; dTTP biosynthesis.
Subunit: Homodimer.

(Information from UniProt)

WBGene00022455 associated phenotypes
lethal, sterile

(Information from WormBase)

Arginase-1

Best gene implication in ageing for this target family came from gene P07824 via mapping the annotation from RGD 2150 annotated in RGD 2014-03-11. Annotation GO 7568 (aging) was Inferred from Expression Pattern

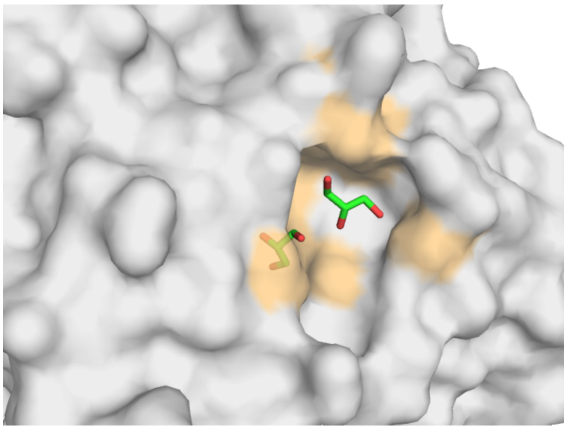

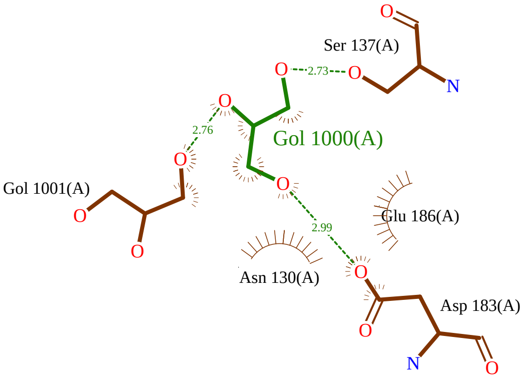

protein	amino acids contacts (binding site)
PDB:1p8n:chainA:P07824	H D H D N S H G D E A T E
PDB:1p8q:chainA:P07824	H D H D N S H G D E D T E
PDB:1ta1:chainA:P07824	H D H D N S C G D E D T E
PDB:1tbj:chainA:P07824	H D H D N S A G D E D T E
sp:P05089:ARGI1_HUMAN	H D H D N S H G D E D T E
sp:P07824:ARGI1_RAT	H D H D N S H G D E D T E
sp:Q61176:ARGI1_MOUSE	H D H D N S H G D E D T E
tr:Q9NHA5:Q9NHA5_DROME	H D H D N S H G D E D T E
tr:O76895:O76895_DROME	H D H D N S H G D E D T E
sp:P00812:ARGI_YEAST	H D H D N S H G D E D T E

	whole protein		domain-based		contact-based	
protein	ident	simil	ident	simil	ident	simil
PDB:1p8n:chainA:P07824	1.0	1.0	1.0	1.0	1.0	1.0
PDB:1p8q:chainA:P07824	1.0	1.0	1.0	1.0	0.92	0.9
PDB:1ta1:chainA:P07824	1.0	1.0	1.0	1.0	0.85	0.77
PDB:1tbj:chainA:P07824	1.0	1.0	1.0	1.0	0.85	0.84
sp:P05089:ARGI1_HUMAN	0.87	0.96	0.87	0.96	0.92	0.9
sp:P07824:ARGI1_RAT	1.0	1.0	1.0	1.0	0.92	0.9
sp:Q61176:ARGI1_MOUSE	0.94	0.98	0.93	0.98	0.92	0.9
tr:Q9NHA5:Q9NHA5_DROME	0.33	0.71	0.35	0.74	0.92	0.9
tr:O76895:O76895_DROME	0.33	0.7	0.35	0.74	0.92	0.9
sp:P00812:ARGI_YEAST	0.37	0.71	0.38	0.72	0.92	0.9

arg (FBgn0023535) associated phenotypes
partially lethal - majority die, some die during pupal stage

(Information from FlyBase)

arg (UniProt:Q9NHA5) annotation
Cofactor: Mn(2+) Evidence=(RuleBase:RU361159); Note=Binds 2 manganese ions per subunit. (RuleBase:RU361159);
Pathway: Nitrogen metabolism; urea cycle; L-ornithine and urea from L-arginine: step 1/1. (RuleBase:RU361159).

(Information from UniProt)

arg (UniProt:O76895) annotation
Cofactor: Mn(2+) Evidence=(RuleBase:RU361159); Note=Binds 2 manganese ions per subunit. (RuleBase:RU361159);
Pathway: Nitrogen metabolism; urea cycle; L-ornithine and urea from L-arginine: step 1/1. (RuleBase:RU361159).

(Information from UniProt)

Heat shock protein HSP 90-alpha

Best gene implication in ageing for this target family came from gene Q18688 annotated in UniProt release 2014_02. Annotation GO 8340 (determination of adult lifespan) was Inferred from Genetic Interaction

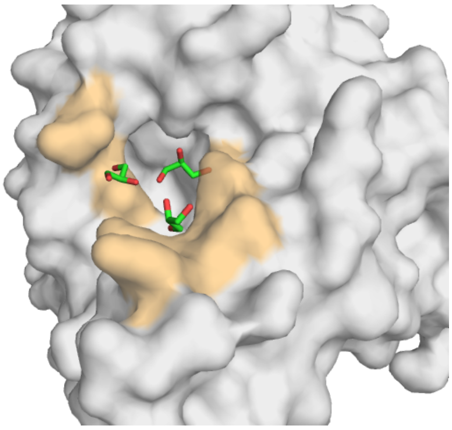

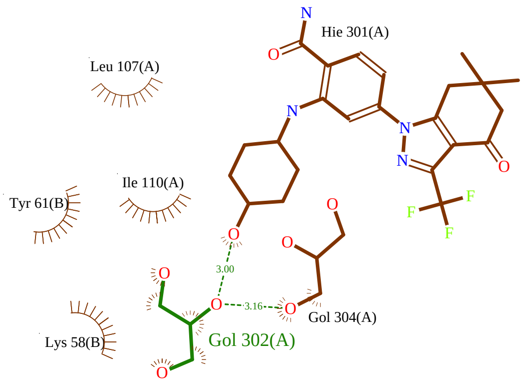

protein	amino acids contacts (binding site)
PDB:2xjj:chainA:P07900	N D D K Y N L I K S G F G V G F Y
PDB:3b28:chainA:P07900	N D D K Y N L I K S G F G V G F Y
PDB:3wha:chainA:P07900	N D D K Y N L I K S G F G V G F Y
PDB:4nh7:chainA:P07900	N D D K Y N L I K S G F G V G F Y
tr:Q86SX1:Q86SX1_HUMAN	N D D K Y N L I K S G F G V G F Y
tr:K9JA46:K9JA46_HUMAN	N D D K Y N L I K S G F G V G F Y
sp:P07900:HS90A_HUMAN	N D D K Y N L I K S G F G V G F Y
tr:Q6B437:Q6B437_RAT	N D D K Y N L I K S G F G V G F Y
sp:P82995:HS90A_RAT	N D D K Y N L I K S G F G V G F Y
tr:B7ZC50:B7ZC50_MOUSE	N D D K Y N L I K S G F G V G F Y
tr:Q8C2A9:Q8C2A9_MOUSE	N D D K Y N L I K S G F G V G F Y
tr:Q3TKB9:Q3TKB9_MOUSE	N D D K Y N L I K S G F G V G F Y
tr:Q3TKG0:Q3TKG0_MOUSE	N D D K Y N L I K S G F G V G F Y
sp:P07901:HS90A_MOUSE	N D D K Y N L I K S G F G V G F Y
tr:Q80Y52:Q80Y52_MOUSE	N D D K Y N L I K S G F G V G F Y
sp:P02828:HSP83_DROME	N D D K Y N L I K S G F G V G F Y
sp:Q18688:HSP90_CAEEL	N D D K Y N L I K S G F G V G F Y
sp:P02829:HSP82_YEAST	N D D K Y N L I K S G F G V G F Y

	whole protein		domain-based		contact-based	
protein	ident	simil	ident	simil	ident	simil
PDB:2xjj:chainA:P07900	1.0	1.0	0.99	1.0	1.0	1.0
PDB:3b28:chainA:P07900	1.0	1.0	1.0	1.0	1.0	1.0
PDB:3wha:chainA:P07900	1.0	1.0	1.0	1.0	1.0	1.0
PDB:4nh7:chainA:P07900	1.0	1.0	1.0	1.0	1.0	1.0
tr:Q86SX1:Q86SX1_HUMAN	0.16	0.16	0.54	0.54	1.0	1.0
tr:K9JA46:K9JA46_HUMAN	1.0	1.0	1.0	1.0	1.0	1.0
sp:P07900:HS90A_HUMAN	1.0	1.0	1.0	1.0	1.0	1.0
tr:Q6B437:Q6B437_RAT	0.22	0.22	0.85	0.85	1.0	1.0
sp:P82995:HS90A_RAT	0.99	1.0	1.0	1.0	1.0	1.0
tr:B7ZC50:B7ZC50_MOUSE	0.27	0.27	0.86	0.86	1.0	1.0
tr:Q8C2A9:Q8C2A9_MOUSE	0.37	0.37	0.99	1.0	1.0	1.0
tr:Q3TKB9:Q3TKB9_MOUSE	0.75	0.76	0.99	1.0	1.0	1.0
tr:Q3TKG0:Q3TKG0_MOUSE	0.75	0.76	0.99	1.0	1.0	1.0
sp:P07901:HS90A_MOUSE	0.99	1.0	0.99	1.0	1.0	1.0
tr:Q80Y52:Q80Y52_MOUSE	0.99	1.0	0.99	1.0	1.0	1.0
sp:P02828:HSP83_DROME	0.77	0.92	0.85	0.96	1.0	1.0
sp:Q18688:HSP90_CAEEL	0.73	0.89	0.76	0.94	1.0	1.0
sp:P02829:HSP82_YEAST	0.6	0.84	0.68	0.9	1.0	1.0

Hsp83 (FBgn0001233) associated phenotypes

cell cycle defective, cell polarity defective, developmental rate defective, female semi-fertile, lethal - all die before end of pupal stage, lethal - all die during third instar larval stage, partially lethal, partially lethal - majority die, short lived

(Information from FlyBase)

Hsp83 (UniProt:P02828) annotation

Function: Molecular chaperone that promotes the maturation, structural maintenance and proper regulation of specific target proteins involved for instance in cell cycle control and signal transduction. Undergoes a functional cycle that is linked to its ATPase activity. This cycle probably induces conformational changes in the client proteins, thereby causing their activation. Interacts dynamically with various co-chaperones that modulate its substrate recognition, ATPase cycle and chaperone function. Together with Hop and piwi, mediates canalization, also known as developmental robustness, likely via epigenetic silencing of existing genetic variants and suppression of transposon-induced new genetic variation. Required for piRNA biogenesis by facilitating loading of piRNAs into PIWI proteins. (PubMed:21186352, PubMed:22902557).
Subunit: Homodimer (By similarity). Forms a complex with Hop and piwi; probably Hop mediates the interaction between piwi and Hsp83. Interacts with shu. (, PubMed:21186352, PubMed:22902557).
Subcellular location: Cytoplasm.

Induction: In contrast to other major heat shock proteins, this one is also expressed at normal growth temperatures. It is also developmentally expressed during oogenesis.
Domain: The TPR repeat-binding motif mediates interaction with TPR repeat-containing proteins.

(Information from UniProt)

daf-21 (WBGene00000915) associated phenotypes
chemotaxis variant, dauer constitutive, dauer recovery variant, larval lethal, lethal, odorant chemosensory response variant, sluggish, sterile

(Information from WormBase)

daf-21 (UniProt:Q18688) annotation
Function: Molecular chaperone that promotes the maturation, structural maintenance and proper regulation of specific target proteins involved for instance in cell cycle control and signal transduction. Undergoes a functional cycle that is linked to its ATPase activity. This cycle probably induces conformational changes in the client proteins, thereby causing their activation. Interacts dynamically with various co-chaperones that modulate its substrate recognition, ATPase cycle and chaperone function. By stabilizing the receptor-type guanylate cyclase daf-11 or another signal transduction component that regulates cGMP levels, plays a role in dauer formation and chemotaxis to non-volatile and volatile attractants detected by AWC sensory neurons (PubMed:10790386, PubMed:7828815). Participates in the control of cell cycle progression at the prophase/metaphase transition in oocyte development by ensuring the activity of wee-1.3 kinase, which negatively regulates cdk-1 through its phosphorylation (PubMed:16466390). Regulates yap-1 nuclear export after heat shock treatment (PubMed:23396260). (PubMed:10790386, PubMed:16466390, PubMed:23396260, PubMed:7828815).
Subunit: Homodimer. (UniProtKB:P08238).
Subcellular location: Cytoplasm, perinuclear region (PubMed:12950278). Note=Perinuclear region of somatic cells.
Tissue specificity: In the embryo comma stage, expression is strongly detected in cells of the head region and less so in other areas. In early larvae, expressed in postembryonic germ cells derived from Z2 and Z3 cells and the head region, in both hermaphrodites and males. Under heat stress conditions, larval expression is not only detected in germ cells, but also all over the body. In adult hermaphrodites, expression is localized uniquely in the germ cells. (PubMed:12950278).
Developmental stage: Highly expressed throughout development. (PubMed:10790386).
Domain: The TPR repeat-binding motif mediates interaction with TPR repeat-containing proteins.

(Information from UniProt)

Neprilysin

Best gene implication in ageing for this target family came from gene P08473 annotated in UniProt release 2014_02. Annotation GO subterm of 7568 (aging) was Inferred from Expression Pattern

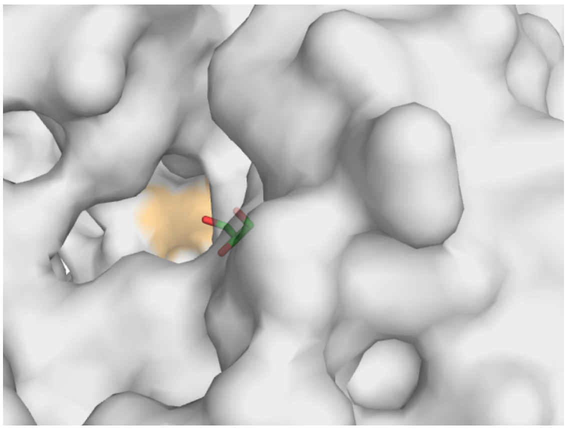

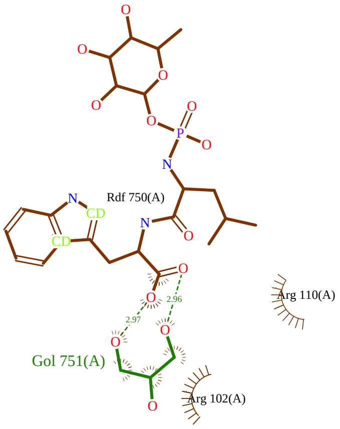

protein	amino acids contacts (binding site)
PDB:1dmt:chainA:P08473	R R N D
sp:P08473:NEP_HUMAN	R R N D
sp:P07861:NEP_RAT	R R N D
sp:Q61391:NEP_MOUSE	R R N D
tr:Q9XZ01:Q9XZ01_DROME	S S N G
tr:Q9W436:Q9W436_DROME	S S N A
tr:Q9I7I4:Q9I7I4_DROME	G R N A
tr:Q8T062:Q8T062_DROME	G R N A
sp:Q18673:NEPL1_CAEEL	S R N D
sp:O16796:NPL11_CAEEL	G R N D

	whole protein		domain-based		contact-based	
protein	ident	simil	ident	simil	ident	simil
PDB:1dmt:chainA:P08473	1.0	1.0	1.0	1.0	1.0	1.0
sp:P08473:NEP_HUMAN	1.0	1.0	1.0	1.0	1.0	1.0
sp:P07861:NEP_RAT	0.94	0.99	0.95	0.99	1.0	1.0
sp:Q61391:NEP_MOUSE	0.94	0.99	0.95	0.99	1.0	1.0
tr:Q9XZ01:Q9XZ01_DROME	0.32	0.71	0.38	0.78	0.25	0.49
tr:Q9W436:Q9W436_DROME	0.34	0.67	0.44	0.81	0.25	0.45
tr:Q9I7I4:Q9I7I4_DROME	0.27	0.56	0.35	0.64	0.5	0.42
tr:Q8T062:Q8T062_DROME	0.26	0.54	0.35	0.64	0.5	0.42
sp:Q18673:NEPL1_CAEEL	0.3	0.7	0.37	0.76	0.75	0.74
sp:O16796:NPL11_CAEEL	0.29	0.67	0.38	0.79	0.75	0.71

Nep4 (FBgn0038818) associated phenotypes

locomotor behavior defective

(Information from FlyBase)

nep-1 (UniProt:Q18673) annotation

Function: Probable cell surface protease. Required to control the neuronal innervation of pharyngeal pumping. (PubMed:16081104).
Cofactor: Zn(2+)Note=Binds 1 zinc ion per subunit. ;
Subcellular location: Membrane; Single-pass type II membrane protein.
Tissue specificity: Specifically expressed in pharyngeal cells and a single head neuron. (PubMed:16081104).
(Information from UniProt)

nep-2 (UniProt:O16796) annotation

Function: Probable cell surface protease.
Cofactor: Zn(2+)Note=Binds 1 zinc ion per subunit. ;
Subcellular location: Cell membrane ; Single-pass type II membrane protein
(Information from UniProt)

Poly [ADP-ribose] polymerase 1

Best gene implication in ageing for this target family came from gene Q921K2 via mapping the annotation from Ensembl ENSMUSG00000026496 via mapping the annotation from EntrezGene 11545 via mapping the annotation from GenAgeModels 1010 annotated in GenAge release 17.

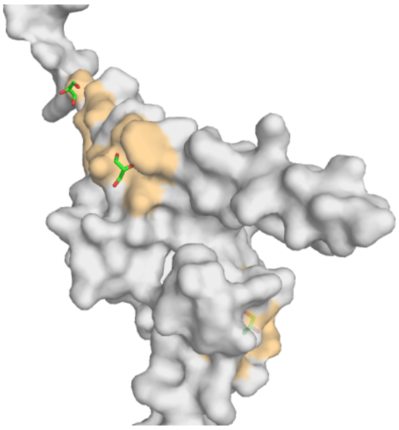

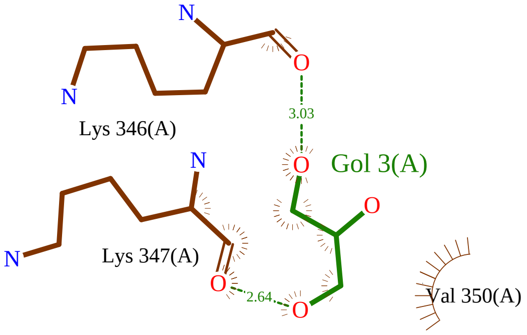

protein	amino acids contacts (binding site)
PDB:2riq:chainA:P09874	W K D K C E V T P N K K V
sp:P09874:PARP1_HUMAN	W K D K C E V T P N K K V
sp:P27008:PARP1_RAT	W K D K C K V T P S K K I
tr:Q921K2:Q921K2_MOUSE	W K D K C K V T P S K K V
sp:P35875:PARP_DROME	F R D K C S K L P T N T E
sp:Q9N4H4:PME1_CAEEL	W R Q E C Q Y S P I L T H

	whole protein		domain-based		contact-based	
protein	ident	simil	ident	simil	ident	simil
PDB:2riq:chainA:P09874	1.0	1.0	1.0	1.0	1.0	1.0
sp:P09874:PARP1_HUMAN	1.0	1.0	1.0	1.0	1.0	1.0
sp:P27008:PARP1_RAT	0.92	0.97	0.86	0.96	0.77	0.9
tr:Q921K2:Q921K2_MOUSE	0.92	0.98	0.88	0.96	0.85	0.92
sp:P35875:PARP_DROME	0.41	0.77	0.28	0.68	0.31	0.56
sp:Q9N4H4:PME1_CAEEL	0.29	0.63	0.19	0.51	0.23	0.58

Parp (FBgn0010247) associated phenotypes

aging defective, heat sensitive, heat stress response defective, immune response defective, increased cell death, lethal - all die before end of pupal stage, non-enhancer of variegation, non-suppressor of variegation, planar polarity defective, some die during pupal stage

(Information from FlyBase)

Parp (UniProt:P35875) annotation

Function: Poly[ADP-ribose] polymerase modifies various nuclear proteins by poly(ADP-ribosyl)ation. The modification is dependent on DNA and is involved in the regulation of various important cellular processes such as differentiation, proliferation, and tumor transformation and also in the regulation of the molecular events involved in the recovery of cell from DNA damage. Plays a fundamental role in organizing chromatin on a global scale; isoform e autoregulates Parp transcription by influencing the chromatin structure of its heterochromatic environment. (PubMed:12183365).
Subcellular location: Nucleus ECO:0000255—PROSITE- ProRule:PRU00264, PubMed:12183365). Nucleus, nucleolus (PubMed:12183365). Note=Highly enriched in nucleoli, heterochromatic chromosomal regions, and diverse euchromatic sites in the cells of most embryonic and adult tissues.
Tissue specificity: Expressed in adult female oocytes, anal plates of stage 12 embryos and in cells around the central nervous system in later embryos. (PubMed:9565614).
Developmental stage: Expressed both maternally and zygotically in embryos, pupae and adults. Expression is highest in embryos. (PubMed:12183365, PubMed:9565614).
Miscellaneous: The ADP-D-ribosyl group of NAD(+) is transferred to an acceptor carboxyl group

on a histone or the enzyme itself, and further ADP-ribosyl groups are transferred to the 2'-position of the terminal adenosine moiety, building up a polymer with an average chain length of 20-30 units.

(Information from UniProt)

pme-1 (UniProt:Q9N4H4) annotation

Function: Poly[ADP-ribose] polymerase modifies various nuclear proteins by poly(ADP-ribosyl)ation, a post-translational modification synthesized after DNA damage that appears as an obligatory step in a detection/signaling pathway leading to the reparation of DNA strand breaks and programmed cell death. Involved in protection of the genome against mutations, probably via some participation in DNA repair. (PubMed:12600937).

Subcellular location: Nucleus ECO:0000305.

Developmental stage: Predominantly expressed at early embryonic stages and later in L4 and adult stages. (PubMed:12145714).

(Information from UniProt)

Heat shock cognate 71 kDa protein

Best gene implication in ageing for this target family came from gene O97125 annotated in UniProt release 2014_02. Annotation GO 8340 (determination of adult lifespan) was Inferred from Mutant Phenotype

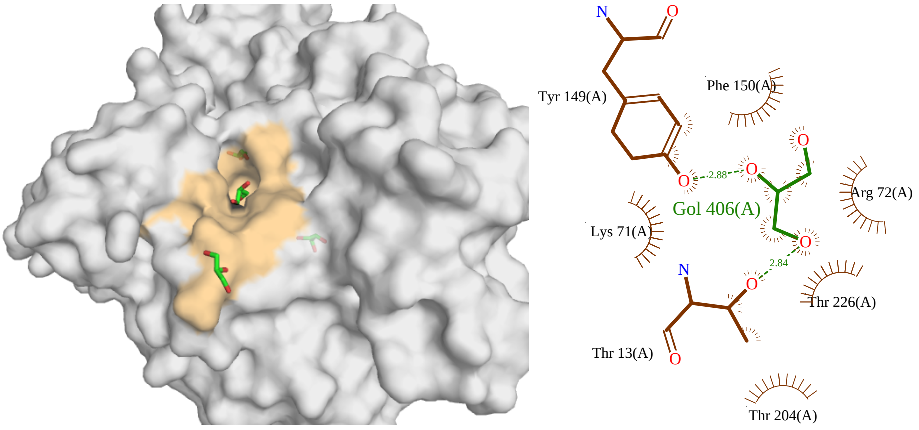

protein	amino acids contacts (binding site)
PDB:4h5n:chainA:P11142	T N R T K R R Y F N D Q T T R T K R S G S R I D
PDB:4h5r:chainA:P11142	T N R T K R R Y F N D Q T T R T K R S G S R I D
PDB:4h5v:chainA:P11142	T N R T K R R Y F N D Q T T R T K R S G S R I D
sp:P08107:HSP71_HUMAN	T N R T K R R Y F N D Q T T R T K R S G S R I D
sp:P11142:HSP7C_HUMAN	T N R T K R R Y F N D Q T T R T K R S G S R I D
tr:A8K5I0:A8K5I0_HUMAN	T N R T K R R Y F N D Q T T R T K R S G S R I D
tr:M0RCB1:M0RCB1_RAT	T N R T K R R Y F N D Q T T R T K R S G S R I D
tr:Q3TB63:Q3TB63_MOUSE	T N R T K R R Y F N D Q T T R T K R S G S R I D
sp:P63017:HSP7C_MOUSE	T N R T K R R Y F N D Q T T R T K R S G S R I D
sp:O97125:HSP68_DROME	T N R T K R R Y F N D Q T T R T K R S G S R I D
sp:P29843:HSP7A_DROME	T N R T K R R Y F N D Q T T R T K R S G S R I D
tr:F3YDI6:F3YDI6_DROME	T N R T K R R Y F N D Q T T R T K R S G S R I D
sp:P82910:HSP70_DROME	T N R T K R R Y F N D Q T T R T K R S G S R I D
sp:P02825:HSP71_DROME	T N R T K R R Y F N D Q T T R T K R S G S R I D
tr:C7LA76:C7LA76_DROME	T N R T K R R Y F N D Q T T R T K R S G S R I D
sp:P11147:HSP7D_DROME	T N R T K R R Y F N D Q T T R T K R S G S R I D
tr:C7LA75:C7LA75_DROME	T N R T K R R Y F N D Q T T R T K R S G S R I D
tr:F3YDH5:F3YDH5_DROME	T N R T K R R Y F N D Q T T R T K R S G S R I D
tr:Q8IH88:Q8IH88_DROME	T N R T K R R Y F N D Q T T R T K R S G S R I D
sp:P09446:HSP7A_CAEEL	T N R T K R R Y F N D Q T T R T K R S G S R I D
sp:P10592:HSP72_YEAST	T N R T K R R Y F N D Q T T R T K R S G S R I D

	whole protein		domain-based		contact-based	
protein	ident	simil	ident	simil	ident	simil
PDB:4h5n:chainA:P11142	1.0	1.0	1.0	1.0	1.0	1.0
PDB:4h5r:chainA:P11142	1.0	1.0	1.0	1.0	1.0	1.0
PDB:4h5v:chainA:P11142	1.0	1.0	1.0	1.0	1.0	1.0
sp:P08107:HSP71_HUMAN	0.84	0.94	0.89	0.97	1.0	1.0
sp:P11142:HSP7C_HUMAN	1.0	1.0	1.0	1.0	1.0	1.0
tr:A8K5I0:A8K5I0_HUMAN	0.84	0.94	0.89	0.97	1.0	1.0
tr:M0RCB1:M0RCB1_RAT	0.98	0.99	0.98	0.98	1.0	1.0
tr:Q3TB63:Q3TB63_MOUSE	0.71	0.71	1.0	1.0	1.0	1.0
sp:P63017:HSP7C_MOUSE	1.0	1.0	1.0	1.0	1.0	1.0
sp:O97125:HSP68_DROME	0.7	0.89	0.82	0.94	1.0	1.0
sp:P29843:HSP7A_DROME	0.8	0.93	0.88	0.97	1.0	1.0
tr:F3YDI6:F3YDI6_DROME	0.73	0.9	0.81	0.94	1.0	1.0
sp:P82910:HSP70_DROME	0.73	0.9	0.81	0.94	1.0	1.0
sp:P02825:HSP71_DROME	0.73	0.9	0.81	0.94	1.0	1.0
tr:C7LA76:C7LA76_DROME	0.73	0.9	0.81	0.94	1.0	1.0
sp:P11147:HSP7D_DROME	0.84	0.95	0.89	0.97	1.0	1.0
tr:C7LA75:C7LA75_DROME	0.84	0.95	0.89	0.97	1.0	1.0
tr:F3YDH5:F3YDH5_DROME	0.77	0.9	0.88	0.97	1.0	1.0
tr:Q8IH88:Q8IH88_DROME	0.75	0.88	0.88	0.97	1.0	1.0
sp:P09446:HSP7A_CAEEL	0.84	0.94	0.88	0.96	1.0	1.0
sp:P10592:HSP72_YEAST	0.75	0.92	0.81	0.94	1.0	1.0

Hsp68 (FBgn0001230) associated phenotypes

chemical resistant, long lived, neuroanatomy defective, partially lethal - majority die, some die during pupal stage, starvation stress response defective

(Information from FlyBase)

Hsc70-1 (FBgn0001216) associated phenotypes

partially lethal - majority die, some die during pupal stage

(Information from FlyBase)

Hsc70-1 (UniProt:P29843) annotation

Developmental stage: Heat shock cognate proteins are expressed constitutively during normal development.

(Information from UniProt)

Hsp70Aa (UniProt:P82910) annotation

Induction: Heat shock induces the synthesis of seven proteins at five otherwise inactive sites in the polytene chromosomes of fruit fly larvae. Two separate sites, producing two and three copies, respectively, code for the 70 kDa protein.

Miscellaneous: There are two copies of the gene coding for this protein at chromosome locus 87A7.

(Information from UniProt)

Hsp70Ab (UniProt:P02825) annotation

Induction: Heat shock induces the synthesis of seven proteins at five otherwise inactive sites in the polytene chromosomes of fruit fly larvae. Two separate sites, producing two and three copies, respectively, code for the 70 kDa protein.

Miscellaneous: There are two copies of the gene coding for this protein at chromosome locus 87A7.

(Information from UniProt)

Hsc70-4 (UniProt:P11147) annotation

Subcellular location: Cytoplasm, perinuclear region. Nucleus. Note=Localized to a meshwork of cytoplasmic fibers around the nucleus. Translocates to the nucleus after thermal stress.

Developmental stage: Heat shock cognate proteins are expressed constitutively during normal development.

(Information from UniProt)

hsp-1 (WBGene00002005) associated phenotypes

lethal, sterile

(Information from WormBase)

Fibroblast growth factor receptor 1

Best gene implication in ageing for this target family came from gene P11362 via mapping the annotation from Ensembl ENSG00000077782 via mapping the annotation from EntrezGene 2260 via mapping the annotation from GenAgeHuman 0169 annotated in GenAge release 17.

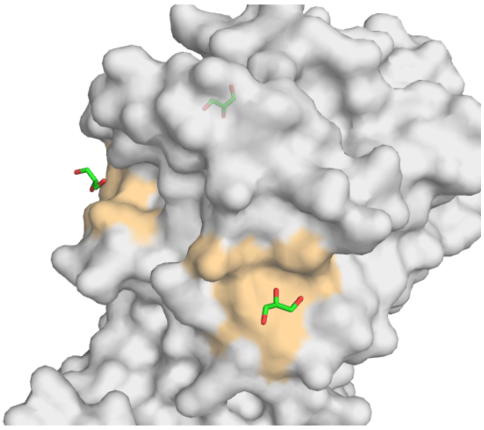

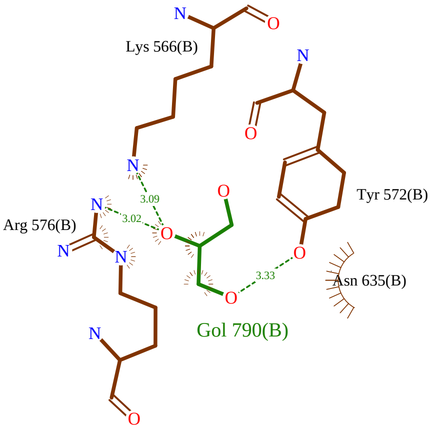

protein	amino acids contacts (binding site)
PDB:3tt0:chainB:P11362	H K Y R R R E Y N G S
tr:D3DSX2:D3DSX2_HUMAN	H K Y R R R E Y N G S
tr:E7EU09:E7EU09_HUMAN	H K Y R R R E Y N G S
tr:J3KNT4:J3KNT4_HUMAN	H K Y R R R E Y N G S
sp:P11362:FGFR1_HUMAN	H K Y R R R E Y N G S
tr:Q63827:Q63827_RAT	H K Y R R R E Y N G S
tr:F1LM54:F1LM54_RAT	H K Y R R R E Y N G S
tr:Q8CBY7:Q8CBY7_MOUSE	H K Y R R R E Y N G S
sp:P16092:FGFR1_MOUSE	H K Y R R R E Y N G S
tr:Q8CIM9:Q8CIM9_MOUSE	H K Y R R R E Y N G S
tr:J3QN85:J3QN85_MOUSE	H K Y R R R E Y N G S
sp:Q09147:FGFR2_DROME	H H F N R R E Y Y D Q
sp:Q10656:EGL15_CAEEL	H H F H R Q N F H G T

	whole protein		domain-based		contact-based	
protein	ident	simil	ident	simil	ident	simil
PDB:3tt0:chainB:P11362	1.0	1.0	1.0	1.0	1.0	1.0
tr:D3DSX2:D3DSX2_HUMAN	0.47	0.47	1.0	1.0	1.0	1.0
tr:E7EU09:E7EU09_HUMAN	0.85	0.87	1.0	1.0	1.0	1.0
tr:J3KNT4:J3KNT4_HUMAN	0.96	0.97	1.0	1.0	1.0	1.0
sp:P11362:FGFR1_HUMAN	1.0	1.0	1.0	1.0	1.0	1.0
tr:Q63827:Q63827_RAT	0.88	0.88	1.0	1.0	1.0	1.0
tr:F1LM54:F1LM54_RAT	0.98	1.0	1.0	1.0	1.0	1.0
tr:Q8CBY7:Q8CBY7_MOUSE	0.42	0.42	1.0	1.0	1.0	1.0
sp:P16092:FGFR1_MOUSE	0.98	1.0	1.0	1.0	1.0	1.0
tr:Q8CIM9:Q8CIM9_MOUSE	0.98	0.99	1.0	1.0	1.0	1.0
tr:J3QN85:J3QN85_MOUSE	0.97	0.98	1.0	1.0	1.0	1.0
sp:Q09147:FGFR2_DROME	0.25	0.54	0.57	0.84	0.45	0.61
sp:Q10656:EGL15_CAEEL	0.25	0.56	0.5	0.78	0.27	0.77

btl (FBgn0005592) associated phenotypes
cell migration defective, neuroanatomy defective, somatic clone

(Information from FlyBase)

btl (UniProt:Q09147) annotation
Function: May be required for patterning of muscle precursor cells: generation of mesodermal and endodermal layers, invaginations of various types of cells, and CNS formation. Essential for the ability of the migrating tracheal and midline cells to recognize external guiding cues. (PubMed:1325393, PubMed:1849109, PubMed:8330538).
Subcellular location: Membrane; Single-pass type I membrane protein.
Tissue specificity: During embryogenesis, expression is seen in mesoderm, endodermal precursor cells, CNS midline cells and trachea and salivary duct ectodermal cells. (PubMed:1325393, PubMed:1849109, PubMed:8330538).
Developmental stage: All stages of development. (PubMed:1849109).

(Information from UniProt)

egl-15 (WBGene00001184) associated phenotypes
L1 arrest, SM migration variant, axon midline crossing variant, bag of worms, bloated, egg laying defective, egg laying imipramine resistant, egg laying levamisole resistant, egg laying phentolamine resistant, egg laying serotonin resistant, egg laying variant, hermaphrodite sex muscle development variant, hermaphrodite sex muscle morphology variant, hermaphrodite sexual development variant, imipramine resistant, larval arrest, larval lethal, scrawny, serotonin resistant, ventral nerve cord maintenance variant

(Information from WormBase)

egl-15 (UniProt:Q10656) annotation
Function: Activation leads to the phosphorylation of SOC-1 on Tyr- 408 triggering PTP-2 recruit-

ment and/or activation. Activates the SEM-5/SOS-1/RAS/MAPK pathway (PubMed:11689700). Acts in the hypodermis to regulate axon growth (PubMed:12835392). Activates protein degradation in muscles (PubMed:14517244). Plays a role in fluid homeostasis by acting in the hypodermis and also has a role in larval development (PubMed:7585964). Isoform b has no effect on axon growth but affects the maintenance of axon position. Isoform b interaction with egl-17 is required for the guidance of sex myoblast migration during gonad development (PubMed:12835392). Isoforms a, c, d and e interaction with let-756 appears to play a role in maintaining body morphology at higher temperatures (PubMed:12835392). (PubMed:11689700, PubMed:12835392, PubMed:14517244, PubMed:7585964).
Cofactor: Mn(2+)
Subcellular location: Membrane; Single-pass type I membrane protein.
Developmental stage: Expressed in the hypodermal membrane during the larval stage. (PubMed:11689700).
Ptm: Activity is regulated by the phosphatase clr-1, however it is not known whether clr-1 acts directly on egl-15. (PubMed:9585503).
Disruption phenotype: Early arrest in larval development. Impaired guided migration of sex myoblasts. (PubMed:7585964).

(Information from UniProt)

DNA topoisomerase 2-alpha

Best gene implication in ageing for this target family came from gene F1M7A0 via mapping the annotation from RGD 62048 annotated in RGD 2014-03-11. Annotation GO 7568 (aging) was Inferred from Expression Pattern

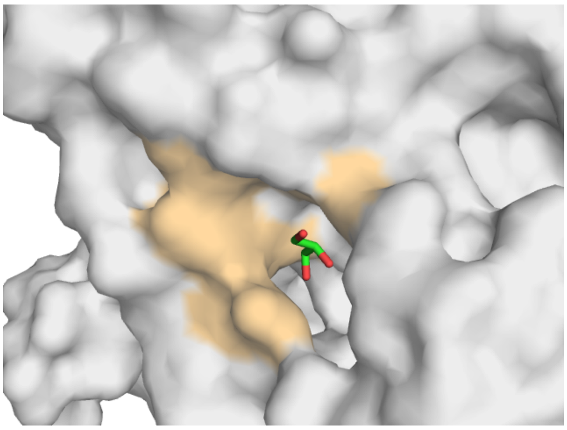

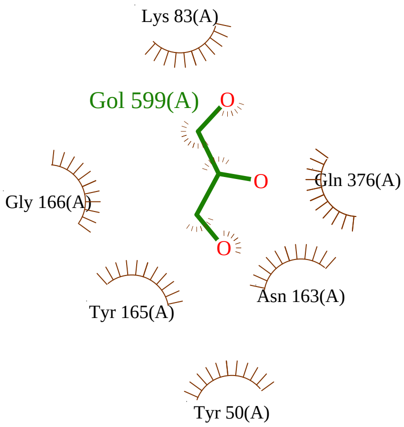

protein	amino acids contacts (binding site)
PDB:1zxn:chainA:P11388	Y K N Y G Q - - - -
PDB:4fm9:chainA:P11388	- - - - - - S S L E
sp:P11388:TOP2A_HUMAN	Y K N Y G Q S S L E
tr:F1M7A0:F1M7A0_RAT	Y K N Y G Q S S L E
sp:Q01320:TOP2A_MOUSE	Y K N Y G Q S S L E
sp:P15348:TOP2_DROME	Y K N Y G Q S S L E
sp:Q23670:TOP2_CAEEL	Y K N Y G Q S S L E
tr:E9P8H0:E9P8H0_YEASX	Y K N Y G Q S N M E
tr:E9P8H2:E9P8H2_YEASX	Y K N Y G Q S N M E
tr:E9P8H4:E9P8H4_YEASX	Y K N Y G Q S N M E
tr:E9P8H5:E9P8H5_YEASX	Y K N Y G Q S N M E
tr:E9P8H7:E9P8H7_YEASX	Y K N Y G Q S N M E
tr:E9P8H8:E9P8H8_YEASX	Y K N Y G Q S N M E
tr:Q7LMA0:Q7LMA0_YEASX	Y K N Y G Q S N M E
tr:E9P8G9:E9P8G9_YEASX	Y K N Y G Q S N M E
sp:P06786:TOP2_YEAST	Y K N Y G Q S N M E

	whole protein		domain-based		contact-based	
protein	ident	simil	ident	simil	ident	simil
PDB:1zxn:chainA:P11388	0.99	1.0	0.99	1.0	0.6	0.2
PDB:4fm9:chainA:P11388	1.0	1.0	1.0	1.0	0.4	0.0
sp:P11388:TOP2A_HUMAN	1.0	1.0	1.0	1.0	1.0	1.0
tr:F1M7A0:F1M7A0_RAT	0.89	0.96	0.97	0.99	1.0	1.0
sp:Q01320:TOP2A_MOUSE	0.89	0.96	0.97	0.99	1.0	1.0
sp:P15348:TOP2_DROME	0.48	0.74	0.69	0.9	1.0	1.0
sp:Q23670:TOP2_CAEEL	0.47	0.74	0.67	0.9	1.0	1.0
tr:E9P8H0:E9P8H0_YEASX	0.37	0.68	0.51	0.8	0.8	0.94
tr:E9P8H2:E9P8H2_YEASX	0.37	0.68	0.51	0.8	0.8	0.94
tr:E9P8H4:E9P8H4_YEASX	0.37	0.68	0.51	0.8	0.8	0.94
tr:E9P8H5:E9P8H5_YEASX	0.37	0.68	0.51	0.8	0.8	0.94
tr:E9P8H7:E9P8H7_YEASX	0.37	0.68	0.51	0.8	0.8	0.94
tr:E9P8H8:E9P8H8_YEASX	0.37	0.68	0.51	0.8	0.8	0.94
tr:Q7LMA0:Q7LMA0_YEASX	0.37	0.68	0.51	0.8	0.8	0.94
tr:E9P8G9:E9P8G9_YEASX	0.37	0.68	0.51	0.8	0.8	0.94
sp:P06786:TOP2_YEAST	0.37	0.68	0.51	0.8	0.8	0.94

Top2 (FBgn0003732) associated phenotypes

developmental rate defective, heat sensitive, lethal - all die before end of first instar larval stage, lethal - all die before end of second instar larval stage, lethal - all die during first instar larval stage, male fertile, neuroanatomy defective, partially lethal - majority die, small body, somatic clone

(Information from FlyBase)

Top2 (UniProt:P15348) annotation

Function: Control of topological states of DNA by transient breakage and subsequent rejoining of DNA strands. Topoisomerase II makes double-strand breaks.
Cofactor: Mg(2+) Evidence=(PROSITE-ProRule:PRU00995); Name=Mn(2+); Xref=ChEBI:CHEBI:29035; Evidence=(PROSITE-ProRule:PRU00995); Name=Ca(2+); Xref=ChEBI:CHEBI:29108; Evidence=(PROSITE-ProRule:PRU00995); Note=Binds two Mg(2+) per subunit. The magnesium ions form salt bridges with both the protein and the DNA. Can also accept other divalent metal cations, such as Mn(2+) or Ca(2+). (PROSITE-ProRule:PRU00995);
Subunit: Homodimer.
Subcellular location: Nucleus.
Miscellaneous: Eukaryotic topoisomerase I and II can relax both negative and positive supercoils, whereas prokaryotic enzymes relax only negative supercoils.

(Information from UniProt)

K12D12.1 (UniProt:Q23670) annotation
Function: Control of topological states of DNA by transient breakage and subsequent rejoining of DNA strands. Topoisomerase II makes double-strand breaks (By similarity).
Cofactor: Mg(2+) Evidence=(PROSITE-ProRule:PRU00995); Name=Mn(2+); Xref=ChEBI:CHEBI:29035; Evidence=(PROSITE-ProRule:PRU00995); Name=Ca(2+); Xref=ChEBI:CHEBI:29108; Evidence=(PROSITE-ProRule:PRU00995); Note=Binds two Mg(2+) per subunit. The magnesium ions form salt bridges with both the protein and the DNA. Can also accept other divalent metal cations, such as Mn(2+) or Ca(2+). (PROSITE-ProRule:PRU00995);
Subunit: Homodimer.
Subcellular location: Nucleus
Miscellaneous: Eukaryotic topoisomerase I and II can relax both negative and positive supercoils, whereas prokaryotic enzymes relax only negative supercoils.

(Information from UniProt)

Glucose-6-phosphate 1-dehydrogenase

Best gene implication in ageing for this target family came from gene P12646 via mapping the annotation from Ensembl FBgn0004057 via mapping the annotation from EntrezGene 32974 via mapping the annotation from GenAgeModels 0883 annotated in GenAge release 17.

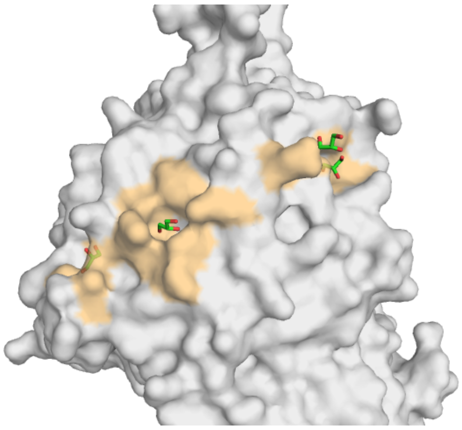

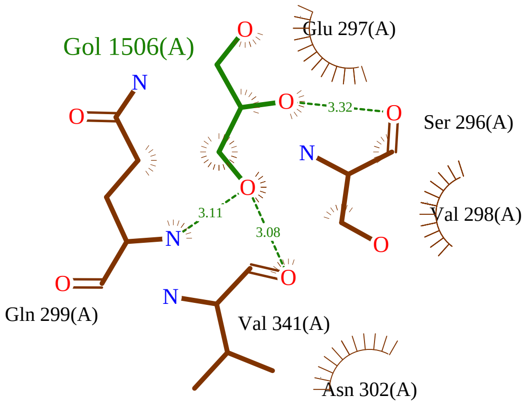

protein	amino acids contacts (binding site)
PDB:1qki:chainA:P11413	R F R G W G Y E S E V Q A N V L T V Y E E R D P Q L P
PDB:2bh9:chainA:P11413	R F R G W G Y E S E V Q A N V L T V Y E E R D P Q L P
PDB:2bhl:chainA:P11413	R F R G W G Y E S E V Q A N V L T V Y E E R D P Q L P
tr:Q2VF42:Q2VF42_HUMAN	R F R G W G Y E S E V Q A N V L T V Y E E R D P Q L P
sp:P11413:G6PD_HUMAN	R F R G W G Y E S E V Q A N V L T V Y E E R D P Q L P
sp:P05370:G6PD_RAT	R F R G W G Y E S E V E T N V L T V Y E E R D P K R P
tr:Q790Y8:Q790Y8_MOUSE	R F R G W G Y E S E V E T N V L T V Y E E R D P K R P
sp:Q00612:G6PD1_MOUSE	R F R G W G Y E S E V E T N V L T V Y E E R D P K R P
sp:P12646:G6PD_DROME	R F K S W G Y E E A L T L D M L T V K N E R D P Q K I
tr:B7FNK0:B7FNK0_DROME	R F K S W G Y E E A L T L D M L T V K N E R D P Q K I
sp:Q27464:G6PD_CAEEL	R F R A W G Y T K V V E L D V V S V H N E R E P E K V
sp:P11412:G6PD_YEAST	R F Q N W G Y S A P I D T D V L T T N E E R A P H R P

protein	whole protein ident	simil	domain-based ident	simil	contact-based ident	simil
PDB:1qki:chainA:P11413	1.0	1.0	1.0	1.0	1.0	1.0
PDB:2bh9:chainA:P11413	1.0	1.0	1.0	1.0	1.0	1.0
PDB:2bhl:chainA:P11413	1.0	1.0	1.0	1.0	1.0	1.0
tr:Q2VF42:Q2VF42_HUMAN	0.92	0.92	1.0	1.0	1.0	1.0
sp:P11413:G6PD_HUMAN	1.0	1.0	1.0	1.0	1.0	1.0
sp:P05370:G6PD_RAT	0.94	0.98	0.95	0.99	0.85	0.91
tr:Q790Y8:Q790Y8_MOUSE	0.94	0.98	0.95	0.99	0.85	0.91
sp:Q00612:G6PD1_MOUSE	0.94	0.98	0.95	0.99	0.85	0.91
sp:P12646:G6PD_DROME	0.61	0.85	0.69	0.9	0.52	0.71
tr:B7FNK0:B7FNK0_DROME	0.61	0.85	0.69	0.9	0.52	0.71
sp:Q27464:G6PD_CAEEL	0.57	0.83	0.64	0.89	0.44	0.67
sp:P11412:G6PD_YEAST	0.44	0.77	0.49	0.81	0.48	0.64

Zw (FBgn0004057) associated phenotypes
long lived, oxidative stress response defective

(Information from FlyBase)

Zw (UniProt:P12646) annotation
Function: Catalyzes the rate-limiting step of the oxidative pentose-phosphate pathway, which represents a route for the dissimilation of carbohydrates besides glycolysis. The main function of this enzyme is to provide reducing power (NADPH) and pentose phosphates for fatty acid and nucleic acid synthesis (By similarity).
Pathway: Carbohydrate degradation; pentose phosphate pathway; D- ribulose 5-phosphate from D-glucose 6-phosphate (oxidative stage): step 1/3.

(Information from UniProt)

Zw-RA (UniProt:B7FNK0) annotation
Pathway: Carbohydrate degradation; pentose phosphate pathway; D- ribulose 5-phosphate from D-glucose 6-phosphate (oxidative stage): step 1/3. (RuleBase:RU000497).

(Information from UniProt)

gspd-1 (WBGene00007108) associated phenotypes
lethal, sterile

(Information from WormBase)

gspd-1 (UniProt:Q27464) annotation
Function: Catalyzes the rate-limiting step of the oxidative pentose-phosphate pathway, which represents a route for the dissimilation of carbohydrates besides glycolysis. The main function of this enzyme is to provide reducing power (NADPH) and pentose phosphates for fatty acid and nucleic acid synthesis (By similarity).
Pathway: Carbohydrate degradation; pentose phosphate pathway; D- ribulose 5-phosphate from D-glucose 6-phosphate (oxidative stage): step 1/3.

(Information from UniProt)

Angiotensin-converting enzyme

Best gene implication in ageing for this target family came from gene M0RB66 via mapping the annotation from Ensembl ENSRNOG00000007467 via mapping the annotation from RGD 2493 annotated in RGD 2014-03-11. Annotation GO 7568 (aging) was Inferred from Expression Pattern

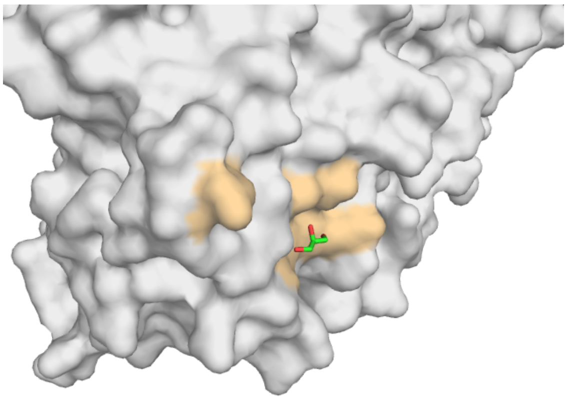

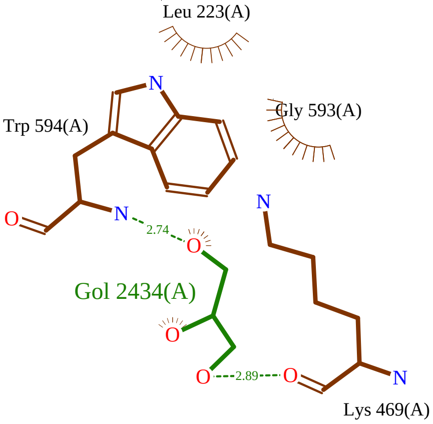

protein	amino acids contacts (binding site)
PDB:2c6f:chainA:P12821	E L K Q G W
tr:D3DU13:D3DU13_HUMAN	Q L K E G W
tr:L7MUH0:L7MUH0_HUMAN	Q L K E G W
sp:P12821:ACE_HUMAN	Q L K E G W
tr:M0RB66:M0RB66_RAT	Q L K V G W
tr:Q8K233:Q8K233_MOUSE	Q L K R G W
tr:Q3TU20:Q3TU20_MOUSE	Q L K R G W
sp:P09470:ACE_MOUSE	Q L K R G W
sp:Q10714:ACE_DROME	R Q E I G W
sp:Q9VLJ6:ACER_DROME	L R E S G W
sp:Q18581:ACN1_CAEEL	Q Q K E G W

	whole protein		domain-based		contact-based	
protein	ident	simil	ident	simil	ident	simil
PDB:2c6f:chainA:P12821	0.55	0.85	0.49	0.83	1.0	1.0
tr:D3DU13:D3DU13_HUMAN	0.47	0.47	0.93	0.93	0.67	0.91
tr:L7MUH0:L7MUH0_HUMAN	0.46	0.51	1.0	1.0	0.67	0.91
sp:P12821:ACE_HUMAN	1.0	1.0	1.0	1.0	0.67	0.91
tr:M0RB66:M0RB66_RAT	0.25	0.42	0.55	0.85	0.67	0.81
tr:Q8K233:Q8K233_MOUSE	0.65	0.74	0.79	0.94	0.67	0.89
tr:Q3TU20:Q3TU20_MOUSE	0.83	0.95	0.79	0.94	0.67	0.89
sp:P09470:ACE_MOUSE	0.83	0.95	0.79	0.94	0.67	0.89
sp:Q10714:ACE_DROME	0.2	0.37	0.38	0.77	0.33	0.57
sp:Q9VLJ6:ACER_DROME	0.18	0.37	0.37	0.77	0.33	0.52
sp:Q18581:ACN1_CAEEL	0.11	0.33	0.22	0.63	0.5	0.73

Ance (FBgn0012037) associated phenotypes

partially lethal - majority live

(Information from FlyBase)

Ance (UniProt:Q10714) annotation

Function: May be involved in the specific maturation or degradation of a number of bioactive peptides. May play a role in the contractions of the heart, gut and testes, and in spermatid differentiation. (PubMed:12591244).

Cofactor: Zn(2+)Note=Binds 1 zinc ion per subunit.;

Enzyme regulation: Inhibited by captopril and, to a lesser extent, by lisinopril, trandolaprilat, fosinoprilat and enalaprilat. (PubMed:7775412, PubMed:8761461).

Biophysicochemical properties: Kinetic parameters: KM=33.5 uM for angiotensin I (PubMed:7775412, PubMed:8761461, PubMed:9839949); KM=53.4 uM for N-acetyl-Ser-Asp-Lys-Pro (PubMed:7775412, PubMed:8761461, PubMed:9839949); KM=2.59 mM for Hip-His-Leu (PubMed:7775412, PubMed:8761461, PubMed:9839949); KM=10.26 mM for Hip-His-Leu-NH(2) (PubMed:7775412, PubMed:8761461, PubMed:9839949); KM=372 uM for (Leu5)enkephalin (PubMed:7775412, PubMed:8761461, PubMed:9839949); KM=1.88 mM for (Leu5)enkephalinamide (PubMed:7775412, PubMed:8761461, PubMed:9839949);

Subcellular location: Secreted, extracellular space.
Tissue specificity: Expressed in vesicular structures in spermatocytes and early spermatids (at protein level). (PubMed:12591244).
Developmental stage: Expressed in the amnioserosa during germ band elongation, shortening and heart morphogenesis. Expressed in midgut throughout embryogenesis.
Ptm: Glycosylated. (PubMed:8761461).
Disruption phenotype: Male flies are sterile. (PubMed:12591244).

(Information from UniProt)

Acer (FBgn0016122) associated phenotypes
circadian behavior defective, short lived, sleep defective

(Information from FlyBase)

Acer (UniProt:Q9VLJ6) annotation
Function: May be involved in the specific maturation or degradation of a number of bioactive peptides. May have a role in the specification of heart progenitors. (PubMed:12075344).
Cofactor: Zn(2+)Note=Binds 1 zinc ion per subunit. ;
Enzyme regulation: Inhibited by captopril, lisinopril, trandolaprilat, fosinoprilat and enalaprilat. (PubMed:9839949).
Biophysicochemical properties: Kinetic parameters: KM=1.2 mM for Hip-His-Leu (PubMed:9839949); KM=4.35 mM for Hip-His-Leu-NH(2) (PubMed:9839949); KM=40.6 uM for (Leu5)enkephalin (PubMed:9839949); KM=949 uM for (Leu5)enkephalinamide (PubMed:9839949); pH dependence: Optimum pH is 8.6. (PubMed:9839949);
Subcellular location: Secreted, extracellular space (PubMed:9839949).
Developmental stage: Expressed in presumptive heart cells during dorsal closure. (PubMed:8973330).
Ptm: Glycosylated. (PubMed:9839949).
Disruption phenotype: Defective heart morphogenesis leading to lethality. (PubMed:12075344).
Miscellaneous: In contrast to ance, does not hydrolyze angiotensin I.

(Information from UniProt)

acn-1 (WBGene00000039) associated phenotypes
embryonic lethal, lethal, sterile

(Information from WormBase)

acn-1 (UniProt:Q18581) annotation
Function: Required for larval molting, male tail development, and formation of adult alae. Inactive as a metallopeptidase, due to a lack of active site residues. (PubMed:14559923).
Tissue specificity: Expressed in embryonic and larval hypodermis, in the vulva during organogenesis, and in the ray papillae of the male tail. (PubMed:14559923).

(Information from UniProt)

Macrophage migration inhibitory factor

Best gene implication in ageing for this target family came from gene P34884 annotated in UniProt release 2014_02. Annotation GO subterm of 7568 (aging) was Inferred from Mutant Phenotype

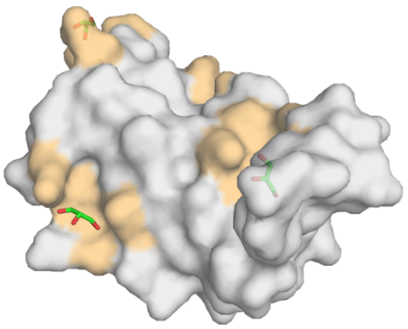

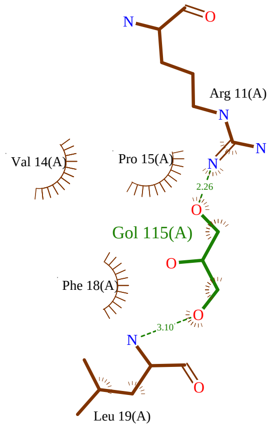

protein	amino acids contacts (binding site)
PDB:2ooh:chainA:P14174	P M R V P G F L S Q Y L G S S H S I G K D Y N M V W T F A
PDB:2oow:chainA:P14174	P M R V P G F L S Q Y L G S S H S I G K D Y N M V W T F A
PDB:2ooz:chainA:P14174	P M R V P G F L S Q Y L G S S H S I G K D Y N M V W T F A
PDB:3b9s:chainA:P14174	P M R V P G F L S Q Y L G S S H S I G K D Y N M V W T F A
PDB:3djh:chainA:P14174	P M R V P G F L S Q Y L G S S H S I G K D Y N M V W T F A
PDB:3l5p:chainA:P14174	P M R V P G F L S Q Y L G S S H S I G K D Y N M V W T F A
PDB:3l5r:chainA:P14174	P M R V P G F L S Q Y L G S S H S I G K D Y N M V W T F A
PDB:3l5v:chainA:P14174	P M R V P G F L S Q Y L G S S H S I G K D Y N M V W T F A
PDB:3u18:chainA:P14174	P M R V P G F L S Q Y L G S S H S I G K D Y N M V W T F A
PDB:3wnr:chainA:P14174	P M R V P G F L S Q Y L G S S H S I G K D Y N M V W T F A
PDB:3wns:chainA:P14174	P M R V P G F L S Q Y L G S S H S I G K D Y N M V W T F A
PDB:4oyq:chainA:P14174	P M R V P G F L S Q Y L G S S H S I G K D Y N M V W T F A
sp:P14174:MIF_HUMAN	P M R V P G F L S Q Y L G S S H S I G K D Y N M V W T F A
tr:I4AY87:I4AY87_HUMAN	P M R V P G F L S Q Y L G S S H S I G K D Y N M V W T F A
sp:P30904:MIF_RAT	P M R V P G F L S Q Y L G T S H S I G K D Y N M V W T F A
sp:P34884:MIF_MOUSE	P M R V P G F L S Q Y L G T N H S I G K D Y N M V W T F A
tr:Q545F0:Q545F0_MOUSE	P M R V P G F L S Q Y L G T N H S I G K D Y N M V W T F A
tr:Q9U228:Q9U228_CAEEL	P V P K Q E I L K Q Y G G T T K S I G G N Y E L I Y T F A
sp:Q18785:MIF2_CAEEL	P M N V P D F E I E R R A T H K S I G A D V T L V F T V A
tr:G3MU38:G3MU38_CAEEL	P M N V P D F E I E R R A T H K S I G A D V T L V F T V A

	whole protein		domain-based		contact-based	
protein	ident	simil	ident	simil	ident	simil
PDB:2ooh:chainA:P14174	1.0	1.0	1.0	1.0	1.0	1.0
PDB:2oow:chainA:P14174	1.0	1.0	1.0	1.0	1.0	1.0
PDB:2ooz:chainA:P14174	1.0	1.0	1.0	1.0	1.0	1.0
PDB:3b9s:chainA:P14174	1.0	1.0	1.0	1.0	1.0	1.0
PDB:3djh:chainA:P14174	1.0	1.0	1.0	1.0	1.0	1.0
PDB:3l5p:chainA:P14174	1.0	1.0	1.0	1.0	1.0	1.0
PDB:3l5r:chainA:P14174	1.0	1.0	1.0	1.0	1.0	1.0
PDB:3l5v:chainA:P14174	0.97	0.97	1.0	1.0	1.0	1.0
PDB:3u18:chainA:P14174	1.0	1.0	1.0	1.0	1.0	1.0
PDB:3wnr:chainA:P14174	1.0	1.0	1.0	1.0	1.0	1.0
PDB:3wns:chainA:P14174	1.0	1.0	1.0	1.0	1.0	1.0
PDB:4oyq:chainA:P14174	1.0	1.0	1.0	1.0	1.0	1.0
sp:P14174:MIF_HUMAN	1.0	1.0	1.0	1.0	1.0	1.0
tr:I4AY87:I4AY87_HUMAN	1.0	1.0	1.0	1.0	1.0	1.0
sp:P30904:MIF_RAT	0.9	0.98	0.9	0.98	0.97	0.98
sp:P34884:MIF_MOUSE	0.9	0.97	0.89	0.97	0.93	0.97
tr:Q545F0:Q545F0_MOUSE	0.9	0.97	0.89	0.97	0.93	0.97
tr:Q9U228:Q9U228_CAEEL	0.33	0.78	0.33	0.78	0.41	0.65
sp:Q18785:MIF2_CAEEL	0.32	0.73	0.32	0.76	0.41	0.61
tr:G3MU38:G3MU38_CAEEL	0.27	0.62	0.32	0.76	0.41	0.61

Matrix metalloproteinase-9

Best gene implication in ageing for this target family came from gene D3ZYK8 via mapping the annotation from RGD 621320 annotated in RGD 2014-03-11. Annotation GO 7568 (aging) was Inferred from Expression Pattern

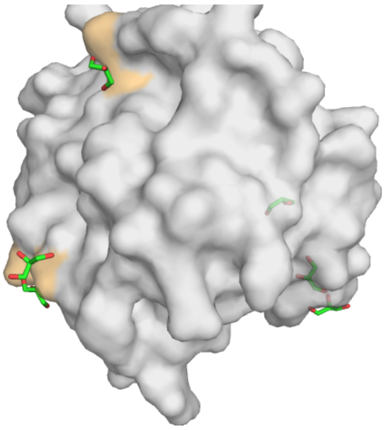

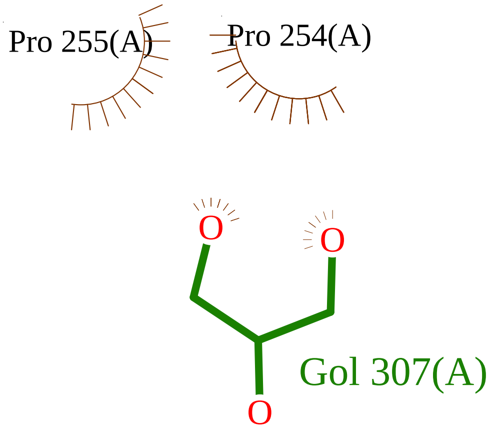

protein	amino acids contacts (binding site)
PDB:4h3x:chainA:P14780	F L N T T D G Y L H P G L D P P N G H
PDB:4hma:chainA:P14780	F L N T T D G Y L H P G L D P P N G H
PDB:4jij:chainA:P14780	F L N T T D G Y L H P G L D P P N G H
PDB:4jqg:chainA:P14780	F L N T T D G Y L H P G L D P P N G H
sp:P14780:MMP9_HUMAN	F L N T T D G Y L H P G L D P P N G H
tr:D3ZYK8:D3ZYK8_RAT	F L N T T D G Y L H P G L D S P K G H
sp:P41245:MMP9_MOUSE	F L N T T D G Y L H P G L D F P D G Y
tr:O61265:O61265_CAEEL	R F T K Q D E H V H F G L D I Q C G W
tr:Q95X57:Q95X57_CAEEL	R F T K Q D E H V H F G L D I Q C G W
tr:O44836:O44836_CAEEL	R F T K Q D E H V H F G L D I Q C G W
tr:G5EBU3:G5EBU3_CAEEL	G F R T S D P W V H M G L E P N S A A

	whole protein		domain-based		contact-based	
protein	ident	simil	ident	simil	ident	simil
PDB:4h3x:chainA:P14780	0.99	0.99	0.99	0.99	1.0	1.0
PDB:4hma:chainA:P14780	0.99	1.0	0.99	1.0	1.0	1.0
PDB:4jij:chainA:P14780	0.98	0.99	0.98	0.99	1.0	1.0
PDB:4jqg:chainA:P14780	0.98	0.99	0.98	0.99	1.0	1.0
sp:P14780:MMP9_HUMAN	1.0	1.0	1.0	1.0	1.0	1.0
tr:D3ZYK8:D3ZYK8_RAT	0.75	0.92	0.84	0.95	0.89	0.91
sp:P41245:MMP9_MOUSE	0.72	0.89	0.82	0.94	0.84	0.89
tr:O61265:O61265_CAEEL	0.11	0.29	0.24	0.5	0.32	0.54
tr:Q95X57:Q95X57_CAEEL	0.11	0.33	0.25	0.57	0.32	0.54
tr:O44836:O44836_CAEEL	0.13	0.37	0.27	0.6	0.32	0.54
tr:G5EBU3:G5EBU3_CAEEL	0.18	0.45	0.33	0.67	0.32	0.63

WBGene00019212 associated phenotypes
lethal, sterile

(Information from WormBase)

Heterogeneous nuclear ribonucleoprotein L

Best gene implication in ageing for this target family came from gene A1ZBN3 annotated in UniProt release 2014_02. Annotation GO 8340 (determination of adult lifespan) was Inferred from Mutant Phenotype

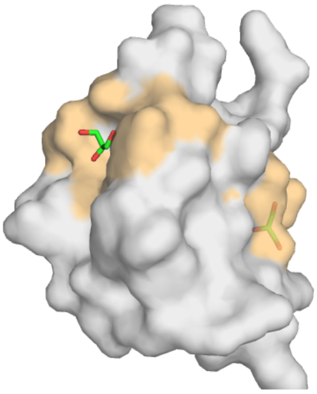

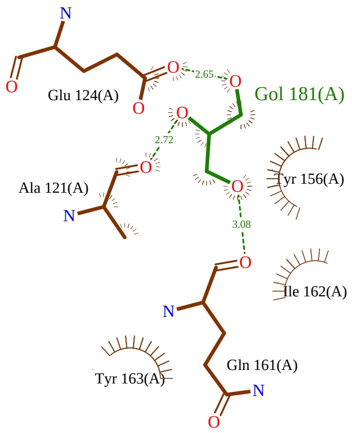

protein	amino acids contacts (binding site)
PDB:3r27:chainA:P14866	H R A E Q L Y Q I Y S T - - - - - - - - - - -
PDB:3to8:chainA:P14866	- - - - - - - - - - - - D F N Y D R T F S N H
tr:Q6NTA2:Q6NTA2_HUMAN	H R A E Q L Y Q I Y S T D F N Y D R T F S N H
sp:P14866:HNRPL_HUMAN	H R A E Q L Y Q I Y S T D F N Y D R T F S N H
tr:F2Z3R2:F2Z3R2_RAT	H R A E Q L Y Q I Y S T D F N Y D R T F S N H
tr:F1LQ48:F1LQ48_RAT	H R A E Q L Y Q I Y S T D F N Y D R T F S N H
sp:Q8R081:HNRPL_MOUSE	H R A E Q L Y Q I Y S T D F N Y D R T F S N H
tr:A1ZBN3:A1ZBN3_DROME	W V - - Q V A P R K M Q D F N V E R Q Y S N H
tr:Q8IHH3:Q8IHH3_DROME	W V - - Q V A P R K M Q D F N V E R Q Y S N H
tr:D5AEJ9:D5AEJ9_DROME	W V - - Q V A P R K M Q D F N V E R Q Y S N H
tr:Q95QR5:Q95QR5_CAEEL	H R A N M L F Q I N S T D F N E Q N D Y S N H

	whole protein		domain-based		contact-based	
protein	ident	simil	ident	simil	ident	simil
PDB:3r27:chainA:P14866	1.0	1.0	1.0	1.0	0.52	0.0
PDB:3to8:chainA:P14866	0.99	0.99	0.99	1.0	0.48	0.0
tr:Q6NTA2:Q6NTA2_HUMAN	0.97	0.97	0.99	0.99	1.0	1.0
sp:P14866:HNRPL_HUMAN	1.0	1.0	1.0	1.0	1.0	1.0
tr:F2Z3R2:F2Z3R2_RAT	0.98	0.99	0.99	1.0	1.0	1.0
tr:F1LQ48:F1LQ48_RAT	0.92	0.93	0.99	1.0	1.0	1.0
sp:Q8R081:HNRPL_MOUSE	0.98	0.99	0.99	1.0	1.0	1.0
tr:A1ZBN3:A1ZBN3_DROME	0.16	0.38	0.28	0.62	0.35	0.47
tr:Q8IHH3:Q8IHH3_DROME	0.16	0.38	0.28	0.62	0.35	0.47
tr:D5AEJ9:D5AEJ9_DROME	0.17	0.43	0.28	0.62	0.35	0.47
tr:Q95QR5:Q95QR5_CAEEL	0.26	0.51	0.36	0.69	0.61	0.71

sm (FBgn0003435) associated phenotypes

feeding behavior defective, long lived, neuroanatomy defective, partially lethal - majority die, short lived, some die during pupal stage

(Information from FlyBase)

2',3'-cyclic-nucleotide 3'-phosphodiesterase

Best gene implication in ageing for this target family came from gene P13233 annotated in UniProt release 2014_02. Annotation GO 7568 (aging) was Inferred from Expression Pattern

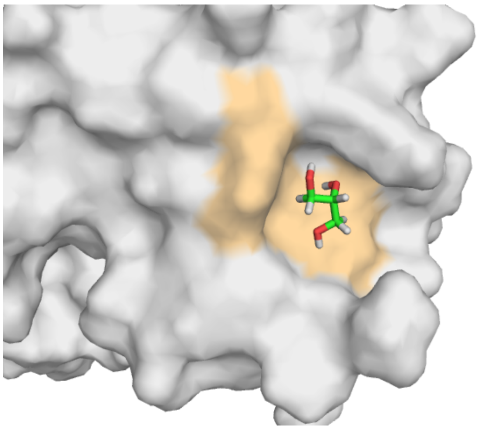

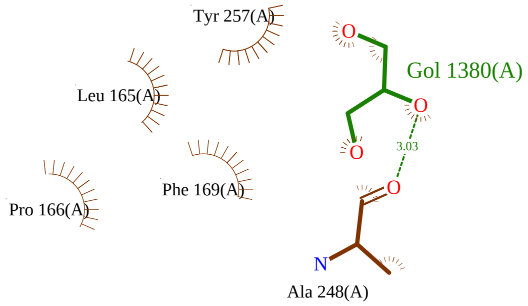

protein	amino acids contacts (binding site)
PDB:2xmi:chainA:P16330	L P F S A Y R K E
PDB:2y1p:chainA:P16330	L P F S A Y R K E
PDB:2yoz:chainA:P16330	L P F S A Y R K E
PDB:2yp0:chainA:P16330	L P F S A Y R K E
sp:P09543:CN37_HUMAN	L P F T A Y R N E
sp:P13233:CN37_RAT	L P F S A Y R K E
tr:Q3TYL9:Q3TYL9_MOUSE	L P F S A Y R K E
sp:P16330:CN37_MOUSE	L P F S A Y R K E
tr:Q3TYV5:Q3TYV5_MOUSE	L P F S A Y R K E
tr:Q19804:Q19804_CAEEL	L - - - - - - - -

	whole protein		domain-based		contact-based	
protein	ident	simil	ident	simil	ident	simil
PDB:2xmi:chainA:P16330	1.0	1.0	1.0	1.0	1.0	1.0
PDB:2y1p:chainA:P16330	1.0	1.0	1.0	1.0	1.0	1.0
PDB:2yoz:chainA:P16330	1.0	1.0	1.0	1.0	1.0	1.0
PDB:2yp0:chainA:P16330	1.0	1.0	1.0	1.0	1.0	1.0
sp:P09543:CN37_HUMAN	0.85	0.96	0.84	0.96	0.78	0.82
sp:P13233:CN37_RAT	0.95	0.99	0.95	0.99	1.0	1.0
tr:Q3TYL9:Q3TYL9_MOUSE	0.95	0.95	1.0	1.0	1.0	1.0
sp:P16330:CN37_MOUSE	1.0	1.0	1.0	1.0	1.0	1.0
tr:Q3TYV5:Q3TYV5_MOUSE	1.0	1.0	1.0	1.0	1.0	1.0
tr:Q19804:Q19804_CAEEL	0.11	0.28	0.06	0.18	0.11	0.0

Tyrosine-protein phosphatase non-receptor type 1

Best gene implication in ageing for this target family came from gene P18031 via mapping the annotation from Ensembl ENSG00000196396 via mapping the annotation from EntrezGene 5770 via mapping the annotation from GenAgeHuman 0033 annotated in GenAge release 17.

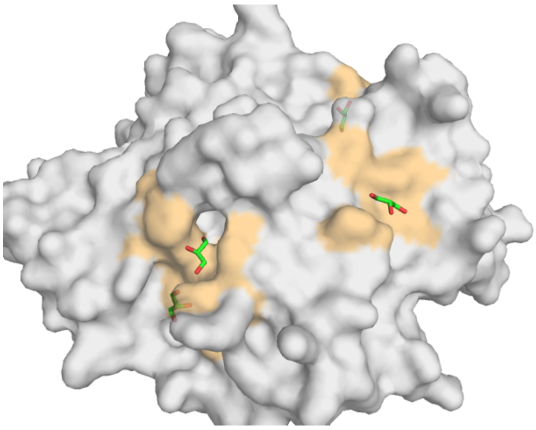

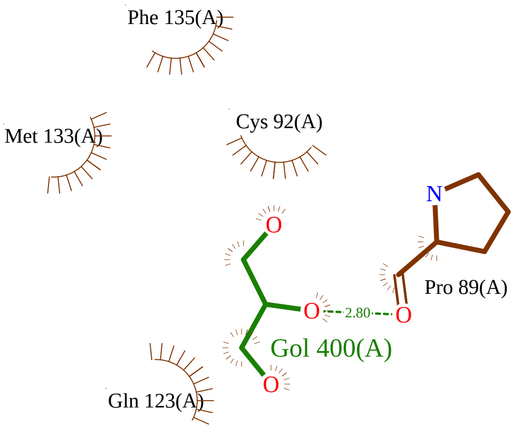

protein	amino acids contacts (binding site)																				
PDB:1sug:chainA:P18031	Y	D	V	P	C	Q	M	F	S	E	D	V	Q	E	E	F	A	I	M	G	Q
PDB:2cng:chainA:P18031	Y	D	V	P	C	Q	M	F	S	E	D	V	Q	E	E	F	A	I	M	G	Q
PDB:3i7z:chainA:P18031	Y	D	V	P	C	Q	M	F	S	E	D	V	Q	E	E	F	A	I	M	G	Q
PDB:3i80:chainA:P18031	Y	D	V	P	C	Q	M	F	S	E	D	V	Q	E	E	F	A	I	M	G	Q
PDB:3qkp:chainA:P18031	Y	D	V	P	C	Q	M	F	S	E	D	V	Q	E	E	F	A	I	M	G	Q
PDB:3qkq:chainA:P18031	Y	D	V	P	C	Q	M	F	S	E	D	V	Q	E	E	F	A	I	M	G	Q
sp:P18031:PTN1_HUMAN	Y	D	V	P	C	Q	M	F	S	E	D	V	Q	E	E	F	A	I	M	G	Q
tr:A8K3M3:A8K3M3_HUMAN	Y	D	V	P	C	Q	M	F	S	E	D	V	Q	E	E	F	A	I	M	G	Q
sp:P20417:PTN1_RAT	Y	D	V	P	C	Q	M	F	S	E	D	V	Q	E	E	F	A	I	M	G	Q
tr:Q3TB93:Q3TB93_MOUSE	Y	D	V	P	C	Q	M	F	S	E	D	V	Q	E	E	F	A	I	M	G	Q
tr:Q3TZW9:Q3TZW9_MOUSE	Y	D	V	P	C	Q	M	F	S	E	D	V	Q	E	E	F	A	I	M	G	Q
sp:P35821:PTN1_MOUSE	Y	D	V	P	C	Q	M	F	S	E	D	V	Q	E	E	F	A	I	M	G	Q
sp:Q9W0G1:PTP61_DROME	Y	D	V	V	V	L	K	L	L	V	R	R	W	K	T	F	A	I	M	G	Q
tr:D3DMI1:D3DMI1_DROME	Y	D	V	V	V	L	K	L	L	V	R	R	W	K	T	F	A	I	M	G	Q
tr:I0B1P6:I0B1P6_DROME	Y	D	V	V	V	L	K	L	L	V	R	R	W	K	T	F	A	I	M	G	Q

	whole protein		domain-based		contact-based	
protein	ident	simil	ident	simil	ident	simil
PDB:1sug:chainA:P18031	1.0	1.0	1.0	1.0	1.0	1.0
PDB:2cng:chainA:P18031	1.0	1.0	1.0	1.0	1.0	1.0
PDB:3i7z:chainA:P18031	1.0	1.0	1.0	1.0	1.0	1.0
PDB:3i80:chainA:P18031	1.0	1.0	1.0	1.0	1.0	1.0
PDB:3qkp:chainA:P18031	1.0	1.0	1.0	1.0	1.0	1.0
PDB:3qkq:chainA:P18031	1.0	1.0	1.0	1.0	1.0	1.0
sp:P18031:PTN1_HUMAN	1.0	1.0	1.0	1.0	1.0	1.0
tr:A8K3M3:A8K3M3_HUMAN	1.0	1.0	1.0	1.0	1.0	1.0
sp:P20417:PTN1_RAT	0.84	0.94	0.96	0.99	1.0	1.0
tr:Q3TB93:Q3TB93_MOUSE	0.7	0.73	0.95	0.99	1.0	1.0
tr:Q3TZW9:Q3TZW9_MOUSE	0.83	0.94	0.95	0.99	1.0	1.0
sp:P35821:PTN1_MOUSE	0.83	0.94	0.95	0.99	1.0	1.0
sp:Q9W0G1:PTP61_DROME	0.31	0.59	0.49	0.8	0.43	0.48
tr:D3DMI1:D3DMI1_DROME	0.31	0.59	0.49	0.8	0.43	0.48
tr:I0B1P6:I0B1P6_DROME	0.31	0.59	0.49	0.8	0.43	0.48

Ptp61F (UniProt:Q9W0G1) annotation

Function: Non-receptor protein tyrosine phosphatase required for maintaining Dock in its non-phosphorylated state. (PubMed:12014990, PubMed:8463208).
Subcellular location: Isoform A: Cytoplasm. Endomembrane system. Note=Associates with the membranes of the reticular network and the mitochondria.
Subcellular location: Isoform B: Nucleus.
Tissue specificity: Expressed during oogenesis and embryogenesis. Isoform A and isoform B are expressed in distinct patterns. Isoform B is expressed in the mesoderm and neuroblast layer during germband extension and later in the gut epithelia. Isoform A accumulates in 16 segmentally

repeated stripes in the ectoderm during germband extension. These stripes are flanked by, and adjacent to, the domains of engrailed and wingless gene expression in the anterior/posterior axis. In stage 10 embryos, isoform A colocalizes with the area lateral to the denticle belts that will give rise to naked cuticle. Isoform A is also expressed later in embryogenesis in the central nervous system. (PubMed:9256342).

(Information from UniProt)

Choline-phosphate cytidylyltransferase A

Best gene implication in ageing for this target family came from gene Q7K4C7 annotated in UniProt release 2014_02. Annotation GO 8340 (determination of adult lifespan) was Inferred from Mutant Phenotype

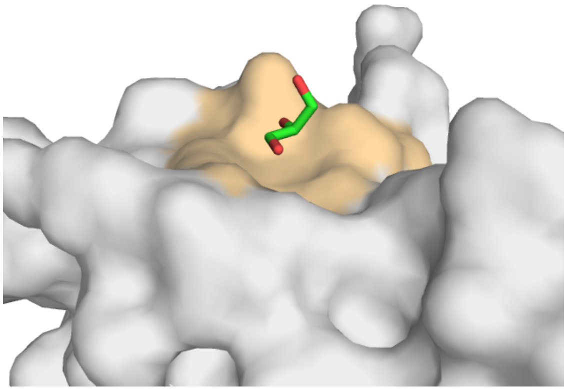

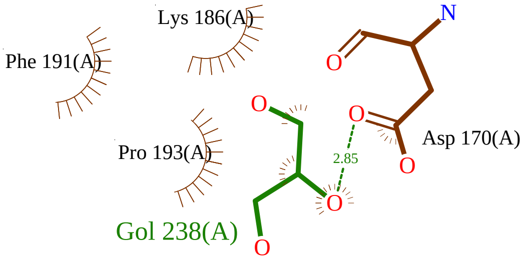

protein	amino acids contacts (binding site)
PDB:3hl4:chainA:P19836	D K F P
tr:H7BZN1:H7BZN1_HUMAN	D K F P
tr:H7C1T3:H7C1T3_HUMAN	D K F P
tr:C9J050:C9J050_HUMAN	D K F P
sp:P49585:PCY1A_HUMAN	D K F P
sp:Q9Y5K3:PCY1B_HUMAN	D K F P
tr:C9JEJ2:C9JEJ2_HUMAN	D K F P
sp:P19836:PCY1A_RAT	D K F P
tr:G3V7M5:G3V7M5_RAT	D K F P
tr:D3Z3T5:D3Z3T5_MOUSE	D K F P
sp:Q811Q9:PCY1B_MOUSE	D K F P
sp:P49586:PCY1A_MOUSE	D K F P
tr:Q7K4C7:Q7K4C7_DROME	D K F A
tr:Q8IU09:Q8IU09_CAEEL	A R F E
tr:Q3HKC4:Q3HKC4_CAEEL	A R F E
sp:P49583:PCY1_CAEEL	A R F E
sp:P13259:PCY1_YEAST	D K F T

protein	whole protein ident	simil	domain-based ident	simil	contact-based ident	simil
PDB:3hl4:chainA:P19836	1.0	1.0	1.0	1.0	1.0	1.0
tr:H7BZN1:H7BZN1_HUMAN	0.31	0.35	0.62	0.62	1.0	1.0
tr:H7C1T3:H7C1T3_HUMAN	0.47	0.51	1.0	1.0	1.0	1.0
tr:C9J050:C9J050_HUMAN	0.78	0.79	1.0	1.0	1.0	1.0
sp:P49585:PCY1A_HUMAN	0.96	0.99	1.0	1.0	1.0	1.0
sp:Q9Y5K3:PCY1B_HUMAN	0.66	0.85	0.89	0.97	1.0	1.0
tr:C9JEJ2:C9JEJ2_HUMAN	0.92	0.95	1.0	1.0	1.0	1.0
sp:P19836:PCY1A_RAT	1.0	1.0	1.0	1.0	1.0	1.0
tr:G3V7M5:G3V7M5_RAT	0.66	0.85	0.89	0.97	1.0	1.0
tr:D3Z3T5:D3Z3T5_MOUSE	0.55	0.65	0.87	0.92	1.0	1.0
sp:Q811Q9:PCY1B_MOUSE	0.66	0.85	0.89	0.97	1.0	1.0
sp:P49586:PCY1A_MOUSE	0.99	1.0	0.99	1.0	1.0	1.0
tr:Q7K4C7:Q7K4C7_DROME	0.33	0.55	0.71	0.91	0.75	0.94
tr:Q8IU09:Q8IU09_CAEEL	0.34	0.57	0.59	0.87	0.25	0.49
tr:Q3HKC4:Q3HKC4_CAEEL	0.37	0.68	0.62	0.88	0.25	0.49
sp:P49583:PCY1_CAEEL	0.36	0.67	0.62	0.88	0.25	0.49
sp:P13259:PCY1_YEAST	0.31	0.66	0.65	0.89	0.75	0.91

Cct1 (FBgn0041342) associated phenotypes

developmental rate defective, lethal - all die before end of first instar larval stage, long lived, partially lethal - majority live, planar polarity defective, somatic clone, some die during first instar larval stage, some die during third instar larval stage

(Information from FlyBase)

F08C6.2 (UniProt:P49583) annotation

Pathway: Phospholipid metabolism; phosphatidylcholine biosynthesis; phosphatidylcholine from phosphocholine: step 1/2.

(Information from UniProt)

Cyclin-A2

Best gene implication in ageing for this target family came from gene P20248 via mapping the annotation from Ensembl ENSG00000145386 via mapping the annotation from EntrezGene 890 via mapping the annotation from GenAgeHuman 0177 annotated in GenAge release 17.

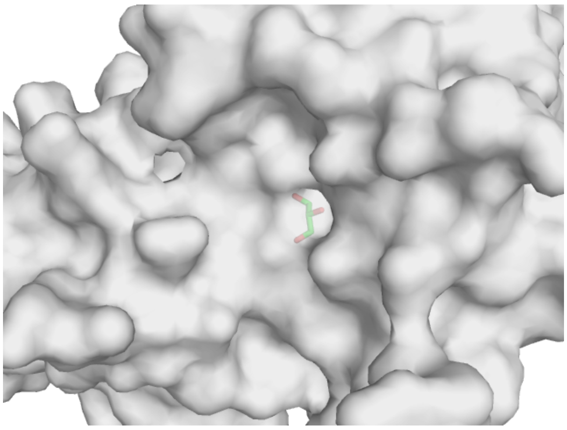

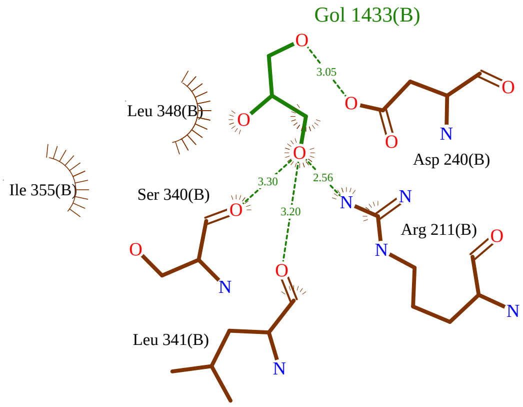

protein	amino acids contacts (binding site)						
PDB:1gy3:chainB:P20248	R	D	M	S	L	L	I
sp:P20248:CCNA2_HUMAN	R	D	M	S	L	L	I
tr:Q6GX89:Q6GX89_RAT	R	D	M	S	L	L	I
tr:G3V802:G3V802_RAT	R	D	M	S	L	L	I
sp:P51943:CCNA2_MOUSE	R	D	M	S	L	L	I
tr:F3YDG4:F3YDG4_DROME	R	D	M	S	L	L	M
tr:F3YDK3:F3YDK3_DROME	R	D	M	S	L	L	M
sp:P14785:CCNA_DROME	R	D	M	S	L	L	M

	whole protein		domain-based		contact-based	
protein	ident	simil	ident	simil	ident	simil
PDB:1gy3:chainB:P20248	1.0	1.0	1.0	1.0	1.0	1.0
sp:P20248:CCNA2_HUMAN	1.0	1.0	1.0	1.0	1.0	1.0
tr:Q6GX89:Q6GX89_RAT	0.63	0.65	0.96	0.99	1.0	1.0
tr:G3V802:G3V802_RAT	0.86	0.93	0.96	0.99	1.0	1.0
sp:P51943:CCNA2_MOUSE	0.85	0.94	0.95	0.99	1.0	1.0
tr:F3YDG4:F3YDG4_DROME	0.26	0.47	0.42	0.73	0.86	0.98
tr:F3YDK3:F3YDK3_DROME	0.31	0.61	0.42	0.73	0.86	0.98
sp:P14785:CCNA_DROME	0.3	0.61	0.42	0.73	0.86	0.98

CycA (FBgn0000404) associated phenotypes

cell migration defective, mitotic cell cycle defective, neuroanatomy defective, partially lethal - majority die, reduced, some die during immature adult stage

(Information from FlyBase)

CycA (UniProt:P14785) annotation

Function: Essential for the control of the cell cycle at the G2/M (mitosis) transition. Interacts with the CDC2 and CDK2 protein kinases to form MPF. G2/M cyclins accumulate steadily during G2 and are abruptly destroyed at mitosis.

(Information from UniProt)

Filamin-A

Best gene implication in ageing for this target family came from gene Q9VEN1 annotated in UniProt release 2014_02. Annotation GO 8340 (determination of adult lifespan) was Inferred from Mutant Phenotype

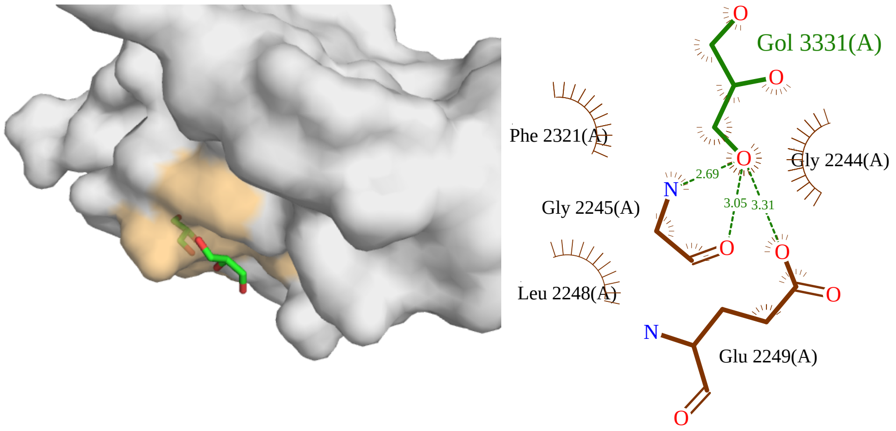

protein	amino acids contacts (binding site)														
PDB:2bp3:chainA:P21333	T	A	G	G	-	-	-	-	-	-	-	-	-	-	-
PDB:2brq:chainA:P21333	-	-	-	-	A	G	G	L	E	P	G	D	F	P	V
PDB:2jf1:chainA:P21333	-	-	-	-	A	G	G	L	E	P	G	D	F	P	V
tr:Q96C61:Q96C61_HUMAN	T	A	G	G	A	G	G	L	E	P	G	D	F	P	V
tr:Q6NXF2:Q6NXF2_HUMAN	T	A	G	G	A	G	G	L	E	P	G	D	F	P	V
tr:Q59H94:Q59H94_HUMAN	T	A	G	G	A	G	G	L	E	P	G	D	F	P	V
tr:Q8TES4:Q8TES4_HUMAN	T	A	G	G	A	G	G	L	E	P	G	D	F	P	V
tr:Q5HY54:Q5HY54_HUMAN	T	A	G	G	A	G	G	L	E	P	G	D	F	P	V
tr:Q60FE6:Q60FE6_HUMAN	T	A	G	G	A	G	G	L	E	P	G	D	F	P	V
tr:Q60FE5:Q60FE5_HUMAN	T	A	G	G	A	G	G	L	E	P	G	D	F	P	V
sp:P21333:FLNA_HUMAN	T	A	G	G	A	G	G	L	E	P	G	D	F	P	V
sp:Q14315:FLNC_HUMAN	T	A	G	G	A	G	G	L	E	P	G	D	F	P	V
tr:C0JPT7:C0JPT7_RAT	T	A	G	G	A	G	G	L	E	P	G	D	F	P	V
sp:D3ZHA0:FLNC_RAT	T	A	G	G	A	G	G	L	E	P	G	D	F	P	V
tr:B7FAV1:B7FAV1_MOUSE	T	A	G	G	A	G	G	L	E	P	G	D	F	P	V
tr:B7FAU9:B7FAU9_MOUSE	T	A	G	G	A	G	G	L	E	P	G	D	F	P	V
sp:Q8BTM8:FLNA_MOUSE	T	A	G	G	A	G	G	L	E	P	G	D	F	P	V
tr:D3YW87:D3YW87_MOUSE	T	A	G	G	A	G	G	L	E	P	G	D	F	P	V
sp:Q8VHX6:FLNC_MOUSE	T	A	G	G	A	G	G	L	E	P	G	D	F	P	V
tr:D3Z576:D3Z576_MOUSE	T	A	G	G	A	G	G	L	E	P	G	D	F	P	V
tr:Q6NN54:Q6NN54_DROME	T	A	S	G	A	G	G	L	E	P	G	E	F	Y	V
tr:C7LAD0:C7LAD0_DROME	T	A	S	G	A	G	G	L	E	P	G	E	F	Y	V
tr:A4V310:A4V310_DROME	T	A	S	G	A	G	G	L	E	P	G	E	F	Y	V
tr:C7LAD1:C7LAD1_DROME	T	A	S	G	A	G	G	L	E	P	G	E	F	Y	V
tr:B7Z0L2:B7Z0L2_DROME	T	A	S	G	A	G	G	L	E	P	G	E	F	Y	V
tr:Q8T3K7:Q8T3K7_DROME	T	A	S	G	A	G	G	L	E	P	G	E	F	Y	V
tr:Q29R34:Q29R34_DROME	T	A	S	G	A	G	G	L	E	P	G	E	F	Y	V
sp:Q9VEN1:FLNA_DROME	T	A	S	G	A	G	G	L	E	P	G	E	F	Y	V
tr:Q9GQV2:Q9GQV2_DROME	T	A	S	G	A	G	G	L	E	P	G	E	F	Y	V
tr:Q7KSF4:Q7KSF4_DROME	T	A	S	G	A	G	G	L	E	P	G	E	F	Y	V
tr:D0IMZ6:D0IMZ6_CAEEL	A	S	Q	G	A	A	G	V	V	P	G	E	F	Y	I
tr:Q3V5H2:Q3V5H2_CAEEL	A	S	Q	G	A	A	G	V	V	P	G	E	F	Y	I
tr:D0IMZ8:D0IMZ8_CAEEL	A	S	Q	G	A	A	G	V	V	P	G	E	F	Y	I
tr:D0IMZ7:D0IMZ7_CAEEL	A	S	Q	G	A	A	G	V	V	P	G	E	F	Y	I
tr:D0IMZ5:D0IMZ5_CAEEL	A	S	Q	G	A	A	G	V	V	P	G	E	F	Y	I

protein	whole protein ident	simil	domain-based ident	simil	contact-based ident	simil
PDB:2bp3:chainA:P21333	0.96	0.98	0.96	0.98	0.27	0.0
PDB:2brq:chainA:P21333	1.0	1.0	1.0	1.0	0.73	0.43
PDB:2jf1:chainA:P21333	1.0	1.0	1.0	1.0	0.73	0.43
tr:Q96C61:Q96C61_HUMAN	0.32	0.32	1.0	1.0	1.0	1.0
tr:Q6NXF2:Q6NXF2_HUMAN	0.37	0.37	1.0	1.0	1.0	1.0
tr:Q59H94:Q59H94_HUMAN	0.33	0.42	0.82	0.95	1.0	1.0
tr:Q8TES4:Q8TES4_HUMAN	0.55	0.55	1.0	1.0	1.0	1.0
tr:Q5HY54:Q5HY54_HUMAN	0.98	0.98	1.0	1.0	1.0	1.0
tr:Q60FE6:Q60FE6_HUMAN	0.99	0.99	1.0	1.0	1.0	1.0
tr:Q60FE5:Q60FE5_HUMAN	0.99	0.99	1.0	1.0	1.0	1.0
sp:P21333:FLNA_HUMAN	1.0	1.0	1.0	1.0	1.0	1.0
sp:Q14315:FLNC_HUMAN	0.7	0.88	0.82	0.95	1.0	1.0
tr:C0JPT7:C0JPT7_RAT	0.97	0.99	0.98	1.0	1.0	1.0
sp:D3ZHA0:FLNC_RAT	0.7	0.88	0.82	0.95	1.0	1.0
tr:B7FAV1:B7FAV1_MOUSE	0.95	0.97	0.97	0.99	1.0	1.0
tr:B7FAU9:B7FAU9_MOUSE	0.97	0.99	0.97	0.99	1.0	1.0
sp:Q8BTM8:FLNA_MOUSE	0.97	0.99	0.97	0.99	1.0	1.0
tr:D3YW87:D3YW87_MOUSE	0.7	0.87	0.82	0.95	1.0	1.0
sp:Q8VHX6:FLNC_MOUSE	0.7	0.88	0.82	0.95	1.0	1.0
tr:D3Z576:D3Z576_MOUSE	0.7	0.88	0.82	0.95	1.0	1.0
tr:Q6NN54:Q6NN54_DROME	0.17	0.27	0.6	0.87	0.8	0.86
tr:C7LAD0:C7LAD0_DROME	0.17	0.27	0.6	0.87	0.8	0.86
tr:A4V310:A4V310_DROME	0.17	0.27	0.6	0.87	0.8	0.86
tr:C7LAD1:C7LAD1_DROME	0.17	0.28	0.6	0.87	0.8	0.86
tr:B7Z0L2:B7Z0L2_DROME	0.17	0.28	0.6	0.87	0.8	0.86
tr:Q8T3K7:Q8T3K7_DROME	0.24	0.42	0.6	0.87	0.8	0.86
tr:Q29R34:Q29R34_DROME	0.24	0.42	0.6	0.87	0.8	0.86
sp:Q9VEN1:FLNA_DROME	0.4	0.68	0.6	0.87	0.8	0.86
tr:Q9GQV2:Q9GQV2_DROME	0.4	0.68	0.6	0.87	0.8	0.86
tr:Q7KSF4:Q7KSF4_DROME	0.43	0.73	0.6	0.87	0.8	0.86
tr:D0IMZ6:D0IMZ6_CAEEL	0.14	0.26	0.5	0.83	0.4	0.61
tr:Q3V5H2:Q3V5H2_CAEEL	0.14	0.27	0.5	0.83	0.4	0.61
tr:D0IMZ8:D0IMZ8_CAEEL	0.27	0.62	0.5	0.83	0.4	0.61
tr:D0IMZ7:D0IMZ7_CAEEL	0.27	0.62	0.5	0.83	0.4	0.61
tr:D0IMZ5:D0IMZ5_CAEEL	0.26	0.61	0.5	0.83	0.4	0.61

cher (FBgn0014141) associated phenotypes

chemical sensitive, heat sensitive, learning defective, locomotor behavior defective, long lived, male fertile, memory defective, neuroanatomy defective, short lived, uncoordinated

(Information from FlyBase)

cher (UniProt:Q9VEN1) annotation

Function: Involved in the germline ring canal formation. May tether actin microfilament within the ovarian ring canal to the cell membrane. Contributes to actin microfilaments organization. (PubMed:10477759, PubMed:10556087).
Subunit: Interacts with Ten-m. (PubMed:21857973).
Subcellular location: Cytoplasm, cytoskeleton. Cell membrane. Note=Localizes to intercellular bridges corresponding to ring canals that connect cytoplasm of nurse cells to the developing oocyte. Localizes to nurse cell plasma membranes. Detected in both the inner and outer rims of the ring canal.
Tissue specificity: Germline-specific in females (at protein level). Expressed in ovary. (PubMed:10477759, PubMed:10556087).
Developmental stage: Expressed throughout the egg-chamber development (at protein level). Expressed in the embryo. (PubMed:10477759, PubMed:10556087).
Disruption phenotype: Shows defect in ring canal assembly and female sterility. (PubMed:10477759).

(Information from UniProt)

fln-1 (WBGene00022048) associated phenotypes

reduced brood size

(Information from WormBase)

Transcription initiation factor TFIID subunit 1

Best gene implication in ageing for this target family came from gene P21675 via mapping the annotation from Ensembl ENSG00000147133 via mapping the annotation from EntrezGene 6872 via mapping the annotation from GenAgeHuman 0180 annotated in GenAge release 17.

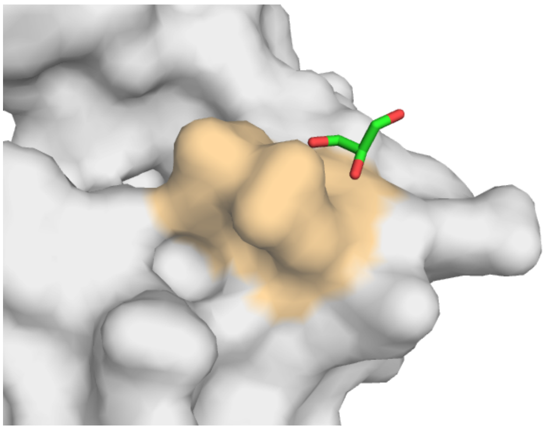

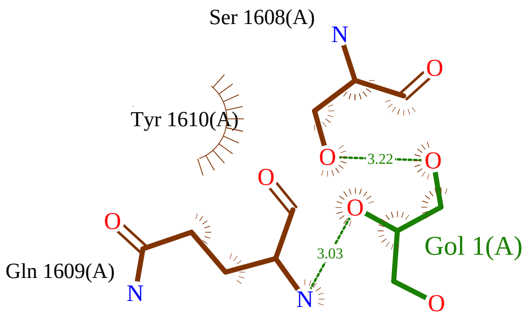

protein	amino acids contacts (binding site)		
PDB:3uv4:chainA:P21675	S	Q	Y
sp:P21675:TAF1_HUMAN	S	Q	Y
tr:D3ZJI8:D3ZJI8_RAT	S	Q	Y
tr:S4R1B9:S4R1B9_MOUSE	S	Q	Y
tr:B1Q2W7:B1Q2W7_MOUSE	S	Q	Y
sp:Q80UV9:TAF1_MOUSE	S	Q	Y
tr:D3YZK4:D3YZK4_MOUSE	S	Q	Y
tr:Q29R44:Q29R44_DROME	T	R	Y
tr:E1JJ72:E1JJ72_DROME	T	R	Y
sp:P51123:TAF1_DROME	T	R	Y
tr:G5EGM3:G5EGM3_CAEEL	S	V	Y
sp:P46677:TAF1_YEAST	-	-	-

	whole protein		domain-based		contact-based	
protein	ident	simil	ident	simil	ident	simil
PDB:3uv4:chainA:P21675	1.0	1.0	1.0	1.0	1.0	1.0
sp:P21675:TAF1_HUMAN	1.0	1.0	1.0	1.0	1.0	1.0
tr:D3ZJI8:D3ZJI8_RAT	0.93	0.97	0.99	1.0	1.0	1.0
tr:S4R1B9:S4R1B9_MOUSE	0.85	0.88	0.98	0.99	1.0	1.0
tr:B1Q2W7:B1Q2W7_MOUSE	0.95	0.97	0.98	0.99	1.0	1.0
sp:Q80UV9:TAF1_MOUSE	0.95	0.98	0.98	0.99	1.0	1.0
tr:D3YZK4:D3YZK4_MOUSE	0.94	0.97	0.98	0.99	1.0	1.0
tr:Q29R44:Q29R44_DROME	0.44	0.71	0.47	0.81	0.33	0.73
tr:E1JJ72:E1JJ72_DROME	0.42	0.68	0.47	0.81	0.33	0.73
sp:P51123:TAF1_DROME	0.42	0.68	0.47	0.81	0.33	0.73
tr:G5EGM3:G5EGM3_CAEEL	0.3	0.59	0.35	0.77	0.67	0.55
sp:P46677:TAF1_YEAST	0.15	0.36	0.0	0.0	0.0	0.0

Taf1 (FBgn0010355) associated phenotypes

cell autonomous, neuroanatomy defective, partially lethal - majority die, some die during pupal stage

(Information from FlyBase)

Taf1 (UniProt:P51123) annotation

Function: TFIID is a multimeric protein complex that plays a central role in mediating promoter

responses to various activators and repressors. Largest component and core scaffold of the complex. Contains N- and C-terminal Ser/Thr kinase domains which can autophosphorylate or transphosphorylate other transcription factors. Possesses DNA-binding activity. Essential for progression of the G1 phase of the cell cycle. Negative regulator of the TATA box-binding activity of Tbp. (PubMed:8504928, PubMed:8625415, PubMed:9741622).
Cofactor:Mg(2+)
Enzyme regulation: Autophosphorylates on Ser residues.
Subunit: Belongs to the TFIID complex which is composed of TATA binding protein (Tbp) and a number of TBP-associated factors (Tafs). Taf1 is the largest component of the TFIID complex. Interacts with Tbp, Taf2, Taf4 and Taf6. (PubMed:8178153, PubMed:8262073, PubMed:8327460, PubMed:8464492).
Subcellular location: Nucleus.
Caution: The C-terminal Ser/Thr kinase domain was reported tophosphorylate histone H2B at 'Ser-33' (PubMed:15143281). However, the paper was retracted because some data, results and conclusions in the paper are not reliable. (PubMed:15143281).

(Information from UniProt)

taf-1 (WBGene00006382) associated phenotypes
lethal, sterile

(Information from WormBase)

Phosphatidylinositol 3-kinase regulatory subunit alpha

Best gene implication in ageing for this target family came from gene G5EDP9 annotated in UniProt release 2014_02. Annotation GO 8340 (determination of adult lifespan) was Inferred from Mutant Phenotype

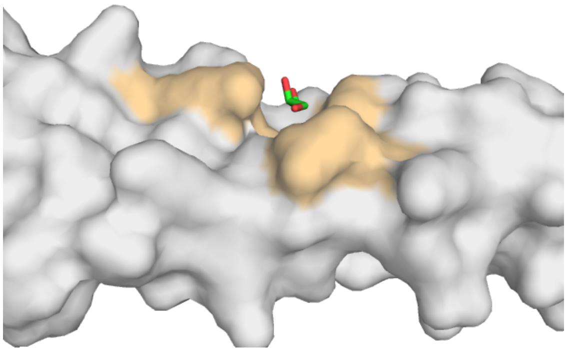

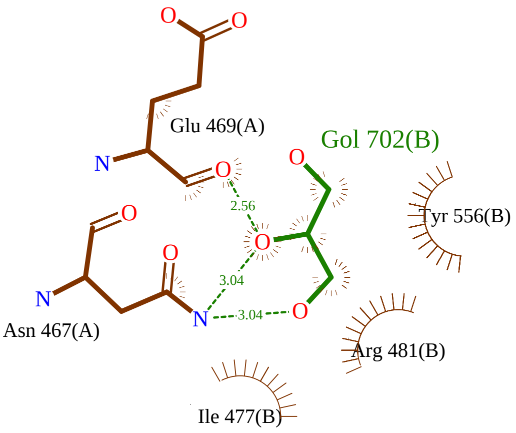

protein	amino acids contacts (binding site)			
PDB:4123:chainB:P27986	I	R	Y	R
PDB:412y:chainB:P27986	I	R	Y	R
tr:J7GXU7:J7GXU7_HUMAN	I	R	Y	R
tr:J7GU64:J7GU64_HUMAN	I	R	Y	R
tr:B3KWZ7:B3KWZ7_HUMAN	I	R	Y	R
sp:P27986:P85A_HUMAN	I	R	Y	R
tr:F1LNG1:F1LNG1_RAT	I	R	Y	R
tr:M0RC47:M0RC47_RAT	I	R	Y	R
tr:F1LNG5:F1LNG5_RAT	I	R	Y	R
tr:Q80UI5:Q80UI5_MOUSE	I	R	Y	R
sp:P26450:P85A_MOUSE	I	R	Y	R
tr:O18683:O18683_DROME	L	K	E	L
tr:G5EDP9:G5EDP9_CAEEL	I	K	M	L

	whole protein		domain-based		contact-based	
protein	ident	simil	ident	simil	ident	simil
PDB:4123:chainB:P27986	1.0	1.0	1.0	1.0	1.0	1.0
PDB:412y:chainB:P27986	1.0	1.0	1.0	1.0	1.0	1.0
tr:J7GXU7:J7GXU7_HUMAN	0.36	0.36	1.0	1.0	1.0	1.0
tr:J7GU64:J7GU64_HUMAN	0.36	0.36	1.0	1.0	1.0	1.0
tr:B3KWZ7:B3KWZ7_HUMAN	0.56	0.56	1.0	1.0	1.0	1.0
sp:P27986:P85A_HUMAN	1.0	1.0	1.0	1.0	1.0	1.0
tr:F1LNG1:F1LNG1_RAT	0.57	0.58	0.99	1.0	1.0	1.0
tr:M0RC47:M0RC47_RAT	0.59	0.61	0.99	1.0	1.0	1.0
tr:F1LNG5:F1LNG5_RAT	0.95	0.99	0.99	1.0	1.0	1.0
tr:Q80UI5:Q80UI5_MOUSE	0.59	0.62	0.99	1.0	1.0	1.0
sp:P26450:P85A_MOUSE	0.96	0.99	0.99	1.0	1.0	1.0
tr:O18683:O18683_DROME	0.17	0.39	0.14	0.64	0.0	0.48
tr:G5EDP9:G5EDP9_CAEEL	0.13	0.33	0.11	0.46	0.25	0.68

Pi3K21B (FBgn0020622) associated phenotypes

decreased cell size, small body, somatic clone

(Information from FlyBase)

aap-1 (WBGene00000001) associated phenotypes

DMPP resistant, slow development

(Information from WormBase)

Nitric oxide synthase, inducible

Best gene implication in ageing for this target family came from gene M0RDG2 via mapping the annotation from RGD 3185 annotated in RGD 2014-03-11. Annotation GO 7568 (aging) was Inferred from Expression Pattern

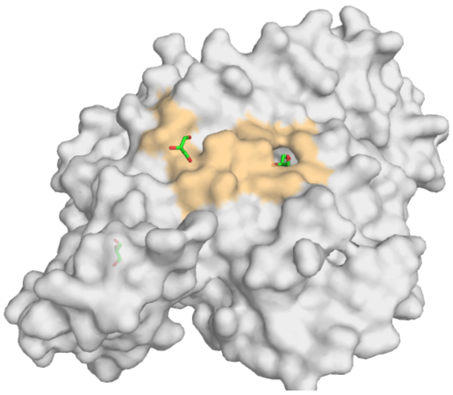

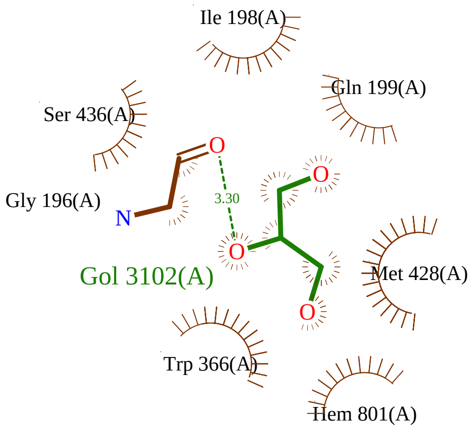

protein	amino acids contacts (binding site)
PDB:1jwj:chainA:P29477	K G I Q S N L Q Y D R Q G W M S T H I
PDB:1jwk:chainA:P29477	K G I Q S N L Q Y D R Q G W M S T H I
PDB:2y37:chainA:P29477	K G I Q S N L Q Y D R Q G W M S T H I
sp:P35228:NOS2_HUMAN	K G I Q S N L Q Y D R Q G W M S T H V
tr:F1LSH9:F1LSH9_RAT	K G I Q S N L Q Y D R Q G W M S T H I
tr:M0RDG2:M0RDG2_RAT	K G I Q S N L Q Y D R Q G W M S T H I
sp:P29477:NOS2_MOUSE	K G I Q S N L Q Y D R Q G W M S T H I
sp:Q27571:NOS_DROME	K G I Q S K L Q Y D R S G W V S T H V
sp:P16603:NCPR_YEAST	- - - - - - - - - - G - - D - - - - -

	whole protein		domain-based		contact-based	
protein	ident	simil	ident	simil	ident	simil
PDB:1jwj:chainA:P29477	1.0	1.0	1.0	1.0	1.0	1.0
PDB:1jwk:chainA:P29477	1.0	1.0	1.0	1.0	1.0	1.0
PDB:2y37:chainA:P29477	1.0	1.0	1.0	1.0	1.0	1.0
sp:P35228:NOS2_HUMAN	0.8	0.93	0.87	0.97	0.95	0.98
tr:F1LSH9:F1LSH9_RAT	0.91	0.95	0.96	0.99	1.0	1.0
tr:M0RDG2:M0RDG2_RAT	0.92	0.95	0.96	0.99	1.0	1.0
sp:P29477:NOS2_MOUSE	1.0	1.0	1.0	1.0	1.0	1.0
sp:Q27571:NOS_DROME	0.38	0.68	0.55	0.85	0.79	0.89
sp:P16603:NCPR_YEAST	0.14	0.38	0.05	0.11	0.0	0.0

Nos (FBgn0011676) associated phenotypes

chemical resistant, developmental rate defective, dominant, eclosion defective, increased cell size, large body, neuroanatomy defective, partially lethal - majority die, small body, some die during pupal stage

(Information from FlyBase)

Nos (UniProt:Q27571) annotation

Function: Produces nitric oxide (NO) which is a messenger molecule with diverse functions throughout the body. Truncated isoforms (isoform 3-isoform 6) are able to form intracellular complexes with the full length protein and serve as dominant negative inhibitors of the enzyme activity. (PubMed:11526108, PubMed:12804606, PubMed:7568075).
Cofactor: heme
Cofactor: FADNote=Binds 1 FAD. ;
Cofactor: FMNNote=Binds 1 FMN. ;
Enzyme regulation: Stimulated by calcium/calmodulin. (PubMed:7568075).
Developmental stage: Isoform 3 is expressed in larvae only. Isoform 4, isoform 5, isoform 6 and isoform 10 are expressed throughout development from embryos to adults. (PubMed:11526108).

(Information from UniProt)

Peroxiredoxin-5, mitochondrial

Best gene implication in ageing for this target family came from gene Q960M4 annotated in UniProt release 2014_02. Annotation GO 8340 (determination of adult lifespan) was Inferred from Mutant Phenotype

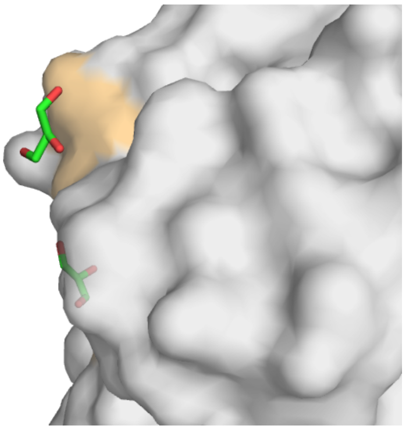

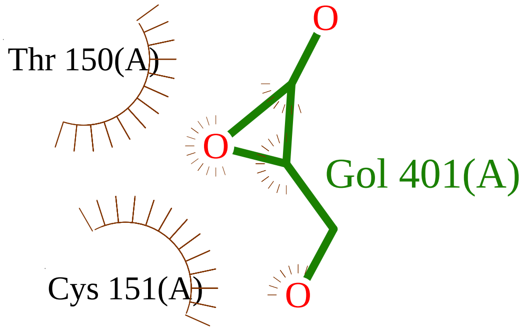

protein	amino acids contacts (binding site)
PDB:3mng:chainA:P30044	K L V T C
sp:P30044:PRDX5_HUMAN	K L V T C
tr:D3ZEN5:D3ZEN5_RAT	K L V T C
sp:Q9R063:PRDX5_RAT	K L V T C
tr:Q91YU9:Q91YU9_MOUSE	K L V T C
tr:G3UZJ4:G3UZJ4_MOUSE	K L V T C
tr:Q3UWS9:Q3UWS9_MOUSE	K L V T C
sp:P99029:PRDX5_MOUSE	K L V T C
tr:H3BJQ7:H3BJQ7_MOUSE	K L V T C
tr:Q960M4:Q960M4_DROME	K L V S C
sp:P38013:AHP1_YEAST	G A K T V

	whole protein		domain-based		contact-based	
protein	ident	simil	ident	simil	ident	simil
PDB:3mng:chainA:P30044	1.0	1.0	1.0	1.0	1.0	1.0
sp:P30044:PRDX5_HUMAN	1.0	1.0	1.0	1.0	1.0	1.0
tr:D3ZEN5:D3ZEN5_RAT	0.66	0.73	0.84	0.93	1.0	1.0
sp:Q9R063:PRDX5_RAT	0.78	0.91	0.89	0.97	1.0	1.0
tr:Q91YU9:Q91YU9_MOUSE	0.37	0.4	0.47	0.51	1.0	1.0
tr:G3UZJ4:G3UZJ4_MOUSE	0.59	0.7	0.66	0.72	1.0	1.0
tr:Q3UWS9:Q3UWS9_MOUSE	0.76	0.87	0.89	0.97	1.0	1.0
sp:P99029:PRDX5_MOUSE	0.77	0.9	0.89	0.97	1.0	1.0
tr:H3BJQ7:H3BJQ7_MOUSE	0.66	0.78	0.85	0.95	1.0	1.0
tr:Q960M4:Q960M4_DROME	0.46	0.73	0.56	0.84	0.8	0.87
sp:P38013:AHP1_YEAST	0.19	0.55	0.23	0.64	0.2	0.26

Prx5 (FBgn0038570) associated phenotypes

chemical resistant, chemical sensitive, immune response defective, increased cell death, long lived, parental effect, short lived, stress response defective

(Information from FlyBase)

RAC-alpha serine/threonine-protein kinase

Best gene implication in ageing for this target family came from gene Q17941 annotated in UniProt release 2014_02. Annotation GO 8340 (determination of adult lifespan) was Inferred from Mutant Phenotype

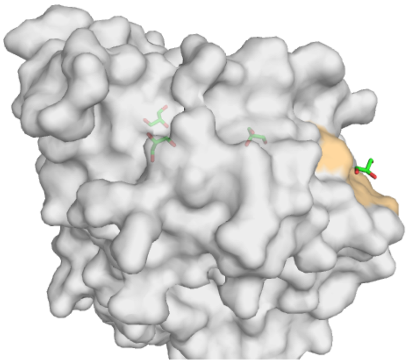

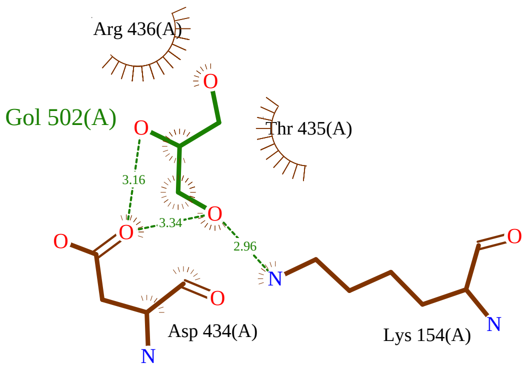

protein	amino acids contacts (binding site)
PDB:4gv1:chainA:P31749	K G K K L I E H T E E F E D G F C G T P L D T R F
tr:B3KP61:B3KP61_HUMAN	K G K K L I E H T E E F E D G F C G T P L D T R F
tr:B7Z5R1:B7Z5R1_HUMAN	K G K K L I E H T E E F E D G F C G T P L D T R F
tr:B4DG79:B4DG79_HUMAN	K G K K L I E H T E E F E D G F C G T P L D T R F
tr:M0R0P9:M0R0P9_HUMAN	K G K K L I E H T E E F E D G F C G T P L D T R F
sp:Q9Y243:AKT3_HUMAN	K G K K L I E H T E E F E D G F C G T P L D T R F
sp:P31749:AKT1_HUMAN	K G K K L I E H T E E F E D G F C G T P L D T R F
tr:B0LPE5:B0LPE5_HUMAN	K G K K L I E H T E E F E D G F C G T P L D T R F
sp:P31751:AKT2_HUMAN	K G K K L I E H T E E F E D G F C G T P L D T R F
tr:F1M6A8:F1M6A8_RAT	K G K K L I E H T E E F E D G F C G T P L D T R F
sp:P47196:AKT1_RAT	K G K K L I E H T E E F E D G F C G T P L D T R F
tr:Q3HSE5:Q3HSE5_RAT	K G K K L I E H T E E F E D G F C G T P L D T R F
tr:Q8CE74:Q8CE74_MOUSE	K G K K L I E H T E E F E D G F C G T P L D T R F
sp:Q9WUA6:AKT3_MOUSE	K G K K L I E H T E E F E D G F C G T P L D T R F
sp:Q60823:AKT2_MOUSE	K G K K L I E H T E E F E D G F C G T P L D T R F
tr:Q3TY95:Q3TY95_MOUSE	K G K K L I E H T E E F E D G F C G T P L D T R F
sp:P31750:AKT1_MOUSE	K G K K L I E H T E E F E D G F C G T P L D T R F
sp:Q8INB9:AKT1_DROME	K G K K L I E H T E E F E D G F C G T P L D T R F
tr:H1ZY64:H1ZY64_DROME	K G K K L I E H T E E F E D G F C G T P L D T R F
tr:H1ZY63:H1ZY63_DROME	K G K K L I E H T E E F E D G F C G T P L D T R F
sp:Q17941:AKT1_CAEEL	K G K K L I E H T E E F E D G F C G T P L D T S F
sp:P11792:SCH9_YEAST	R G K K L I E H T E E F E D G F C G T T L D T S F

protein	whole protein ident	simil	domain-based ident	simil	contact-based ident	simil
PDB:4gv1:chainA:P31749	0.99	1.0	0.99	1.0	1.0	1.0
tr:B3KP61:B3KP61_HUMAN	0.64	0.74	0.86	0.96	1.0	1.0
tr:B7Z5R1:B7Z5R1_HUMAN	0.87	0.87	1.0	1.0	1.0	1.0
tr:B4DG79:B4DG79_HUMAN	0.71	0.82	0.86	0.96	1.0	1.0
tr:M0R0P9:M0R0P9_HUMAN	0.65	0.76	0.79	0.9	1.0	1.0
sp:Q9Y243:AKT3_HUMAN	0.82	0.94	0.85	0.95	1.0	1.0
sp:P31749:AKT1_HUMAN	1.0	1.0	1.0	1.0	1.0	1.0
tr:B0LPE5:B0LPE5_HUMAN	1.0	1.0	1.0	1.0	1.0	1.0
sp:P31751:AKT2_HUMAN	0.81	0.94	0.86	0.96	1.0	1.0
tr:F1M6A8:F1M6A8_RAT	0.82	0.94	0.85	0.95	1.0	1.0
sp:P47196:AKT1_RAT	0.98	0.99	0.99	1.0	1.0	1.0
tr:Q3HSE5:Q3HSE5_RAT	0.81	0.94	0.86	0.96	1.0	1.0
tr:Q8CE74:Q8CE74_MOUSE	0.62	0.7	0.86	0.96	1.0	1.0
sp:Q9WUA6:AKT3_MOUSE	0.82	0.94	0.85	0.95	1.0	1.0
sp:Q60823:AKT2_MOUSE	0.81	0.94	0.86	0.96	1.0	1.0
tr:Q3TY95:Q3TY95_MOUSE	0.81	0.94	0.86	0.96	1.0	1.0
sp:P31750:AKT1_MOUSE	0.98	0.99	0.99	1.0	1.0	1.0
sp:Q8INB9:AKT1_DROME	0.48	0.68	0.69	0.9	1.0	1.0
tr:H1ZY64:H1ZY64_DROME	0.48	0.68	0.69	0.9	1.0	1.0
tr:H1ZY63:H1ZY63_DROME	0.48	0.68	0.69	0.9	1.0	1.0
sp:Q17941:AKT1_CAEEL	0.5	0.75	0.64	0.87	0.96	0.96
sp:P11792:SCH9_YEAST	0.19	0.41	0.44	0.79	0.88	0.93

Akt1 (FBgn0010379) associated phenotypes

RU486 conditional, body size defective, cell death defective, cell size defective, chemical resistant, chemical sensitive, circadian rhythm defective, decreased cell death, decreased cell number, decreased cell size, developmental rate defective, germline clone, heat sensitive, hyperplasia, increased cell death, increased cell size, lethal - all die before end of pupal stage, long lived, maternal effect, neuroanatomy defective, neurophysiology defective, nutrition conditional, oxidative stress response defective, partially lethal - majority die, planar polarity defective, polyphasic, short lived, size defective, small body, somatic clone, some die during pupal stage

(Information from FlyBase)

Akt1 (UniProt:Q8INB9) annotation

Function: Serine/threonine kinase involved in various developmental processes. During early embryogenesis, acts as a survival protein. During mid-embryogenesis, phosphorylates and activates trh, a transcription factor required for tracheal cell fate determination. Also regulates tracheal cell migration. Later in development, acts downstream of PI3K and Pk61C/PDK1 in the insulin receptor transduction pathway which regulates cell growth and organ size, by phosphorylating and antagonizing FOXO transcription factor. Controls follicle cell size during oogenesis. May also stimulate cell growth by phosphorylating Gig/Tsc2 and inactivating the Tsc complex. Dephosphorylation of 'Ser-586' by Phlpp triggers apoptosis and suppression of tumor growth. (PubMed:10587646, PubMed:10962553, PubMed:11740943, PubMed:11752451, PubMed:11872800, PubMed:12172554, PubMed:12893776, PubMed:14525946, PubMed:15466161, PubMed:15712201, PubMed:9601646).
Subcellular location: Cytoplasm, cytosol. Cell membrane. Note=Recruited to plasma membrane upon activation.
Tissue specificity: Ubiquitously expressed. Present in ovary, where it is concentrated at the basal side of follicle cells. (PubMed:7876156).
Developmental stage: Expressed both maternally and zygotically. Strongly expressed in embryo and pupa. Weakly expressed in larva. Mildly expressed in adult. (PubMed:15712201, PubMed:7876156).
Domain: Binding of the PH domain to the phosphatidylinositol 3- kinase alpha (PI(3)K) results in its targeting to the plasma membrane.
Ptm: Phosphorylated and activated by Pk61C/PDK1. Phosphorylated on Ser-586 by the rictor-Tor complex. (PubMed:10962553, PubMed:11344272, PubMed:15718470, PubMed:18327897).
Disruption phenotype: Death at the first instar larval stage. (PubMed:9601646).

(Information from UniProt)

akt-1 (WBGene00000102) associated phenotypes

carbon dioxide avoidance variant, dauer arrest variant, developmental delay, extended life span, fat content increased, gene expression level high, germ cell hypersensitive ionizing radiation, germ cell resistant ionizing radiation, hypersensitivity to mutagen, mRNA levels increased, protein expression increased, protein phosphorylation reduced, protein stabilization variant, protein subcellular localization variant, resistant to mutagens, temperature induced dauer formation increased, transgene expression increased, transgene subcellular localization variant

(Information from WormBase)

akt-1 (UniProt:Q17941) annotation

Function: Acts downstream of age-1 and pdk-1 in the daf-2/insulin receptor-like transduction pathway. Essential role in regulating developmental arrest at the dauer stage. Phosphorylates Forkhead-related daf-16 and the longevity-promoting skn-1 transcription factors, which inhibits their entry into the nucleus and antagonizes their functions. Role in immune function and pathogen resistance. (PubMed:10364160, PubMed:11381260, PubMed:11747825, PubMed:15068796, PubMed:18358814, PubMed:18782349, PubMed:9716402).

Cofactor:Mg(2+)

Enzyme regulation: Phosphorylated and activated by pdk-1. (PubMed:15068796).

Subunit: Interacts with pdk-1, sgk-1, akt-2 and daf-16. Part of a complex containing sgk-1, akt-1 and akt-2. (PubMed:15068796).

Tissue specificity: Expressed in neurons, muscle cells of the pharynx, rectal gland cells, and spermatheca. (PubMed:9716402).

Developmental stage: Expressed in late stage embryos and throughout life. (PubMed:9716402).

Disruption phenotype: Increased resistance to pathogens. Simultaneous knockdown of akt-1 and akt-2 result in dauer formation and a weak extension to life span. (PubMed:15068796, PubMed:18782349).

(Information from UniProt)

Endoplasmic reticulum mannosyl-oligosaccharide 1,2-alpha-mannosidase

Best gene implication in ageing for this target family came from gene P53625 annotated in UniProt release 2014_02. Annotation GO 8340 (determination of adult lifespan) was Inferred from Mutant Phenotype

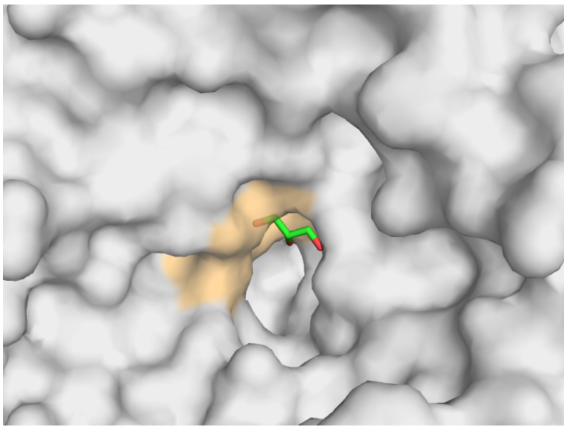

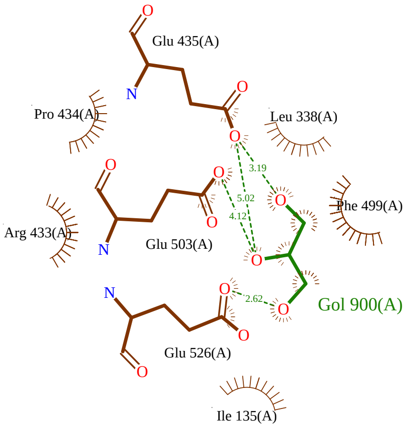

protein	amino acids contacts (binding site)
PDB:1dl2:chainA:P32906	I L R P E F E E
tr:A6NGN6:A6NGN6_HUMAN	I L R P E F E E
tr:B1AJZ5:B1AJZ5_HUMAN	I L R P E F E E
sp:Q9NR34:MA1C1_HUMAN	I L R P E F E E
sp:O60476:MA1A2_HUMAN	I L R P E F E E
sp:P33908:MA1A1_HUMAN	I L R P E F E E
tr:D3Z979:D3Z979_RAT	I L R P E F E E
tr:D3ZR49:D3ZR49_RAT	I L R P E F E E
tr:G3V9N9:G3V9N9_RAT	I L R P E F E E
tr:Q6NXK9:Q6NXK9_MOUSE	I L R P E F E E
sp:P39098:MA1A2_MOUSE	I L R P E F E E
sp:P45700:MA1A1_MOUSE	I L R P E F E E
tr:Q544T7:Q544T7_MOUSE	I L R P E F E E
sp:P53625:MA122_DROME	I L R P E F E E
sp:P53624:MA121_DROME	I L R P E F E E
tr:Q8IRL4:Q8IRL4_DROME	I L R P E F E E
tr:P90787:P90787_CAEEL	I L R P E F E E
sp:Q18788:MAN12_CAEEL	I L R P E F E E
sp:P32906:MNS1_YEAST	I L R P E F E E

	whole protein		domain-based		contact-based	
protein	ident	simil	ident	simil	ident	simil
PDB:1dl2:chainA:P32906	1.0	1.0	1.0	1.0	1.0	1.0
tr:A6NGN6:A6NGN6_HUMAN	0.26	0.55	0.28	0.59	1.0	1.0
tr:B1AJZ5:B1AJZ5_HUMAN	0.28	0.62	0.3	0.66	1.0	1.0
sp:Q9NR34:MA1C1_HUMAN	0.23	0.54	0.31	0.68	1.0	1.0
sp:O60476:MA1A2_HUMAN	0.26	0.54	0.34	0.69	1.0	1.0
sp:P33908:MA1A1_HUMAN	0.24	0.53	0.33	0.69	1.0	1.0
tr:D3Z979:D3Z979_RAT	0.24	0.54	0.31	0.68	1.0	1.0
tr:D3ZR49:D3ZR49_RAT	0.25	0.54	0.33	0.69	1.0	1.0
tr:G3V9N9:G3V9N9_RAT	0.25	0.53	0.33	0.69	1.0	1.0
tr:Q6NXK9:Q6NXK9_MOUSE	0.24	0.54	0.31	0.68	1.0	1.0
sp:P39098:MA1A2_MOUSE	0.26	0.54	0.34	0.7	1.0	1.0
sp:P45700:MA1A1_MOUSE	0.25	0.53	0.33	0.69	1.0	1.0
tr:Q544T7:Q544T7_MOUSE	0.25	0.53	0.33	0.69	1.0	1.0
sp:P53625:MA122_DROME	0.24	0.54	0.31	0.69	1.0	1.0
sp:P53624:MA121_DROME	0.23	0.53	0.31	0.69	1.0	1.0
tr:Q8IRL4:Q8IRL4_DROME	0.22	0.51	0.31	0.69	1.0	1.0
tr:P90787:P90787_CAEEL	0.23	0.58	0.27	0.66	1.0	1.0
sp:Q18788:MAN12_CAEEL	0.24	0.56	0.3	0.68	1.0	1.0
sp:P32906:MNS1_YEAST	1.0	1.0	1.0	1.0	1.0	1.0

alpha-Man-I (FBgn0259170) associated phenotypes

chemical sensitive, dominant, immune response defective, long lived

(Information from FlyBase)

alpha-Man-I (UniProt:P53625) annotation

Function: Involved in the maturation of Asn-linked oligosaccharides. Progressively trim alpha-1,2-linked mannose residues from Man(9)GlcNAc(2) to produce Man(5)GlcNAc(2).
Cofactor: Ca(2+)
Pathway: Protein modification; protein glycosylation.
Subcellular location: Golgi apparatus membrane; Single-pass type II membrane protein.
Tissue specificity: Complex spatial distribution during embryogenesis, including expression in lobula plate giant neurons. Also expressed in adult wing and eyes.
Developmental stage: Expressed both maternally and zygotically during embryonic stages.

(Information from UniProt)

alpha-Man-I (UniProt:P53624) annotation

Function: Involved in the maturation of Asn-linked oligosaccharides. Progressively trim alpha-1,2-

linked mannose residues from Man(9)GlcNAc(2) to produce Man(5)GlcNAc(2).
Cofactor: Ca(2+)
Pathway: Protein modification; protein glycosylation.
Subcellular location: Golgi apparatus membrane; Single-pass type II membrane protein.
Tissue specificity: Complex spatial distribution during embryogenesis, including expression in lobula plate giant neurons. Also expressed in adult wing and eyes.
Developmental stage: Expressed both maternally and zygotically during embryonic stages.

(Information from UniProt)

C52E4.5 (UniProt:Q18788) annotation
Function: Involved in the maturation of Asn-linked oligosaccharides. Progressively trim alpha-1,2-linked mannose residues from Man(9)GlcNAc(2) to produce Man(5)GlcNAc(2) (By similarity).
Cofactor: Ca(2+)
Pathway: Protein modification; protein glycosylation.
Subcellular location: Membrane; Single-pass type II membrane protein.

(Information from UniProt)

Heat shock 70 kDa protein 1-like

Best gene implication in ageing for this target family came from gene O97125 annotated in UniProt release 2014_02. Annotation GO 8340 (determination of adult lifespan) was Inferred from Mutant Phenotype

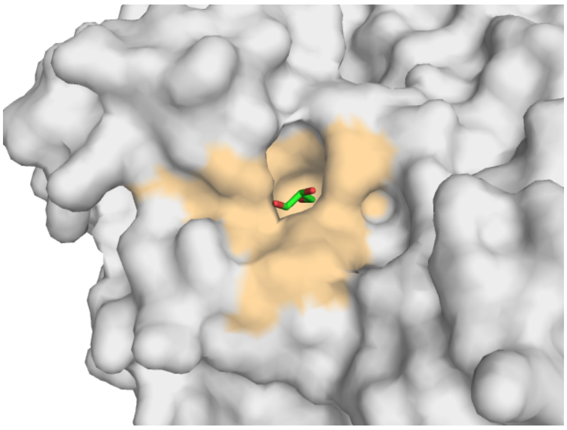

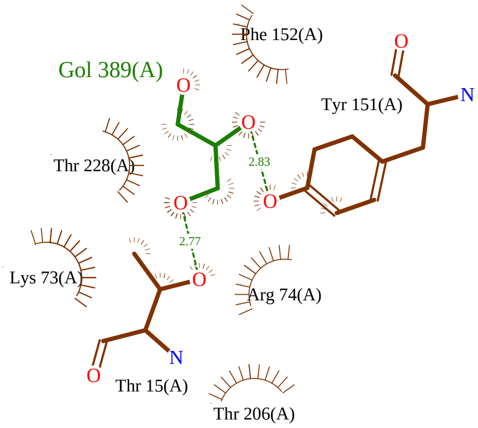

protein	amino acids contacts (binding site)								
PDB:3gdq:chainA:P34931	T	K	R	R	Y	F	Q	T	T
sp:P34931:HS71L_HUMAN	T	K	R	R	Y	F	Q	T	T
tr:M0R8M9:M0R8M9_RAT	T	K	C	R	Y	F	Q	T	T
sp:P17879:HS71B_MOUSE	T	K	R	R	Y	F	Q	T	T
sp:O97125:HSP68_DROME	T	K	R	R	Y	F	Q	T	T
tr:C7LA76:C7LA76_DROME	T	K	R	R	Y	F	Q	T	T
sp:P82910:HSP70_DROME	T	K	R	R	Y	F	Q	T	T
sp:P02825:HSP71_DROME	T	K	R	R	Y	F	Q	T	T
tr:F3YDI6:F3YDI6_DROME	T	K	R	R	Y	F	Q	T	T
tr:G5ECU5:G5ECU5_CAEEL	T	K	R	R	Y	F	Q	T	T
sp:P09446:HSP7A_CAEEL	T	K	R	R	Y	F	Q	T	T
sp:P10592:HSP72_YEAST	T	K	R	R	Y	F	Q	T	T

protein	whole protein ident	simil	domain-based ident	simil	contact-based ident	simil
PDB:3gdq:chainA:P34931	1.0	1.0	1.0	1.0	1.0	1.0
sp:P34931:HS71L_HUMAN	1.0	1.0	1.0	1.0	1.0	1.0
tr:M0R8M9:M0R8M9_RAT	0.81	0.93	0.85	0.96	0.89	0.79
sp:P17879:HS71B_MOUSE	0.87	0.95	0.91	0.98	1.0	1.0
sp:O97125:HSP68_DROME	0.71	0.89	0.83	0.95	1.0	1.0
tr:C7LA76:C7LA76_DROME	0.73	0.9	0.83	0.95	1.0	1.0
sp:P82910:HSP70_DROME	0.73	0.9	0.83	0.95	1.0	1.0
sp:P02825:HSP71_DROME	0.73	0.9	0.83	0.95	1.0	1.0
tr:F3YDI6:F3YDI6_DROME	0.73	0.9	0.83	0.95	1.0	1.0
tr:G5ECU5:G5ECU5_CAEEL	0.67	0.88	0.74	0.92	1.0	1.0
sp:P09446:HSP7A_CAEEL	0.8	0.93	0.87	0.96	1.0	1.0
sp:P10592:HSP72_YEAST	0.72	0.9	0.79	0.94	1.0	1.0

Hsp68 (FBgn0001230) associated phenotypes
chemical resistant, long lived, neuroanatomy defective, partially lethal - majority die, some die during pupal stage, starvation stress response defective

(Information from FlyBase)

Hsp70Aa (UniProt:P82910) annotation
Induction: Heat shock induces the synthesis of seven proteins at five otherwise inactive sites in the polytene chromosomes of fruit fly larvae. Two separate sites, producing two and three copies, respectively, code for the 70 kDa protein.
Miscellaneous: There are two copies of the gene coding for this protein at chromosome locus 87A7.

(Information from UniProt)

Hsp70Ab (UniProt:P02825) annotation
Induction: Heat shock induces the synthesis of seven proteins at five otherwise inactive sites in the polytene chromosomes of fruit fly larvae. Two separate sites, producing two and three copies, respectively, code for the 70 kDa protein.
Miscellaneous: There are two copies of the gene coding for this protein at chromosome locus 87A7.

(Information from UniProt)

hsp-1 (WBGene00002005) associated phenotypes
lethal, sterile

(Information from WormBase)

Catenin beta-1

Best gene implication in ageing for this target family came from gene P35222 via mapping the annotation from Ensembl ENSG00000168036 via mapping the annotation from EntrezGene 1499 via mapping the annotation from GenAgeHuman 0223 annotated in GenAge release 17.

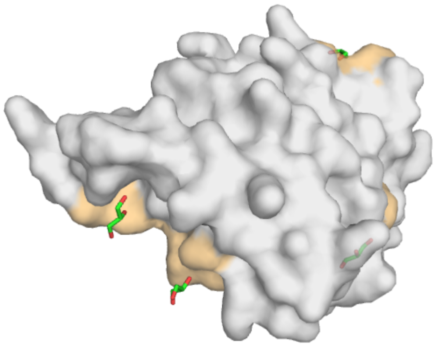

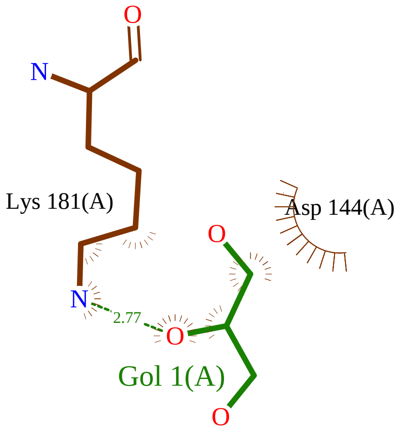

protein	amino acids contacts (binding site)
PDB:3sla:chainA:P35222	D V M Q K K S G A K M R
tr:B4DGU4:B4DGU4_HUMAN	D V M Q K K S G A K M R
sp:P35222:CTNB1_HUMAN	D V M Q K K S G A K M R
sp:Q9WU82:CTNB1_RAT	D V M Q K K S G A K M R
tr:E9Q6A9:E9Q6A9_MOUSE	D V M Q K K S G A K M R
sp:Q02248:CTNB1_MOUSE	D V M Q K K S G A K M R
sp:P18824:ARM_DROME	D M M Q K K S G S K M R

	whole protein		domain-based		contact-based	
protein	ident	simil	ident	simil	ident	simil
PDB:3sla:chainA:P35222	1.0	1.0	1.0	1.0	1.0	1.0
tr:B4DGU4:B4DGU4_HUMAN	0.99	0.99	1.0	1.0	1.0	1.0
sp:P35222:CTNB1_HUMAN	1.0	1.0	1.0	1.0	1.0	1.0
sp:Q9WU82:CTNB1_RAT	1.0	1.0	1.0	1.0	1.0	1.0
tr:E9Q6A9:E9Q6A9_MOUSE	0.74	0.76	0.82	0.83	1.0	1.0
sp:Q02248:CTNB1_MOUSE	1.0	1.0	1.0	1.0	1.0	1.0
sp:P18824:ARM_DROME	0.65	0.83	0.79	0.94	0.83	0.91

arm (FBgn0000117) associated phenotypes

RU486 conditional, cell autonomous, cell migration defective, cell non-autonomous, cell polarity defective, cell shape defective, germline clone, heat sensitive, hyperplasia, increased cell death, lethal - all die before end of pupal stage, memory defective, mitotic cell cycle defective, neuroanatomy defective, partially lethal - majority die, planar polarity defective, segment polarity phenotype, size defective, somatic clone, some die during pupal stage

(Information from FlyBase)

arm (UniProt:P18824) annotation

Function: Isoform neural may associate with CadN and participate in the transmission of developmental information. Can associate with alpha-catenin. Isoform cytoplasmic accumulates through wg signaling; arm function in wg signal transduction is required early in development for determination of neuroblast fate. Arm and Abl proteins function cooperatively at adherens junctions in both the CNS and epidermis. (PubMed:9635189).

Subunit: Interacts with Mer and Moe at the adherens junction. Interacts with Inx2. (PubMed:15047872, PubMed:8666669).

Subcellular location: Cytoplasm ECO:0000269—PubMed:8666669. Cell membrane (PubMed:8666669); Peripheral membrane protein (PubMed:8666669); Cytoplasmic side (PubMed:8666669). Cell junction, adherens junction Note=Inner surface of cell membrane and adherens junction.

Tissue specificity: Isoform cytoplasmic accumulates at low levels in axons, at high levels in specific cells along the CNS midline and in leg and eye imaginal disks. Isoform neural accumulates in the axon tracts of the CNS. Both isoforms accumulate in the peripheral nervous system. (PubMed:9635189).

Developmental stage: Present at all stages, but reaches the highest levels during early to mid-

embryogenesis. Isoform cytoplasmic is the predominant one from the cellular blastoderm stage until germ-band retraction. Isoform neural is first seen after germ band retraction.
Ptm: Phosphorylated on Ser, Thr and Tyr residues. Level of phosphorylation varies both during embryonic development and from embryonic tissue to tissue. Sgg is required for phosphorylation and wg signal negatively regulates arm phosphorylation. Hypophosphorylated form of arm increases in steady-state levels. (PubMed:18327897, PubMed:7529201).

(Information from UniProt)

TGF-beta receptor type-2

Best gene implication in ageing for this target family came from gene P38438 annotated in UniProt release 2014_02. Annotation GO 7568 (aging) was Inferred from Expression Pattern

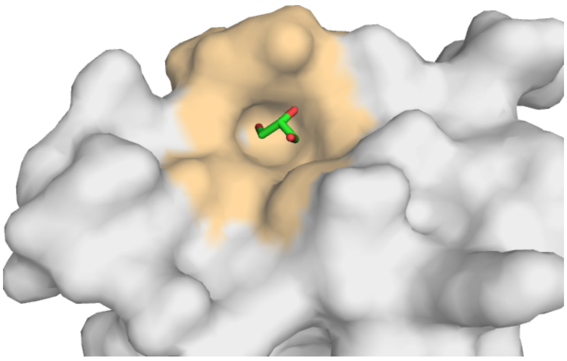

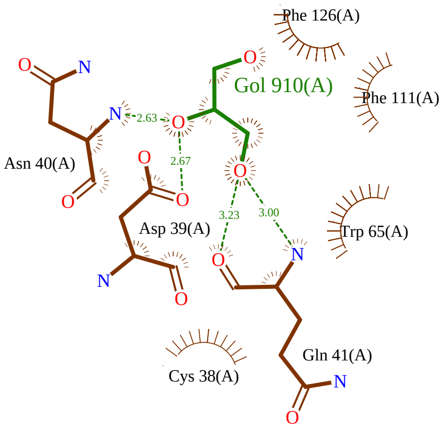

protein	amino acids contacts (binding site)
PDB:1m9z:chainA:P37173	C D N Q W F F
tr:A3QNQ0:A3QNQ0_HUMAN	C D N Q W F F
sp:P37173:TGFR2_HUMAN	C D N Q W F F
tr:D2JYI1:D2JYI1_HUMAN	C D N Q W F F
sp:P38438:TGFR2_RAT	C D N Q W F F
tr:D0VED2:D0VED2_RAT	C D N Q W F F
sp:Q62312:TGFR2_MOUSE	C D N Q W F F
tr:Q543C0:Q543C0_MOUSE	C D N Q W F F
sp:Q09488:SMA6_CAEEL	C G N N E - P

	whole protein		domain-based		contact-based	
protein	ident	simil	ident	simil	ident	simil
PDB:1m9z:chainA:P37173	0.98	0.99	0.99	1.0	1.0	1.0
tr:A3QNQ0:A3QNQ0_HUMAN	1.0	1.0	1.0	1.0	1.0	1.0
sp:P37173:TGFR2_HUMAN	1.0	1.0	1.0	1.0	1.0	1.0
tr:D2JYI1:D2JYI1_HUMAN	0.96	0.96	1.0	1.0	1.0	1.0
sp:P38438:TGFR2_RAT	0.92	0.98	0.83	0.96	1.0	1.0
tr:D0VED2:D0VED2_RAT	0.92	0.98	0.83	0.96	1.0	1.0
sp:Q62312:TGFR2_MOUSE	0.88	0.94	0.86	0.96	1.0	1.0
tr:Q543C0:Q543C0_MOUSE	0.88	0.94	0.86	0.96	1.0	1.0
sp:Q09488:SMA6_CAEEL	0.23	0.57	0.18	0.44	0.29	0.25

sma-6 (WBGene00004860) associated phenotypes
cuticle fragile, levamisole hypersensitive, mRNA levels increased, male tail morphology variant, nicotine hypersensitive, rays fused, reduced brood size, short, small

(Information from WormBase)

sma-6 (UniProt:Q09488) annotation
Function: Involved in TGF-beta pathway. May be a receptor for daf- 7.

Subcellular location: Membrane ECO:0000305; Single-pass type I membrane protein

(Information from UniProt)

Serine/threonine-protein kinase TEL1

Best gene implication in ageing for this target family came from gene Q13315 annotated in UniProt release 2014_02. Annotation GO subterm of 7568 (aging) was Inferred from Mutant Phenotype

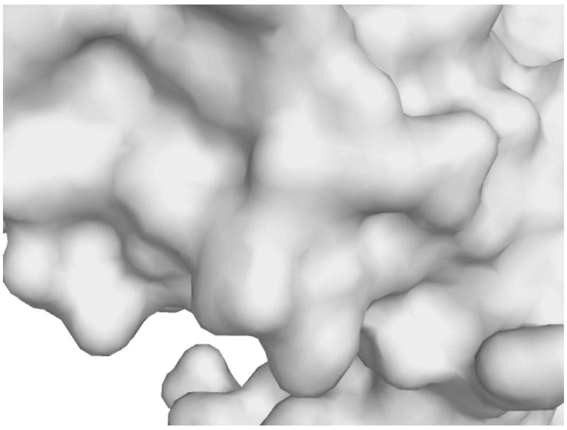

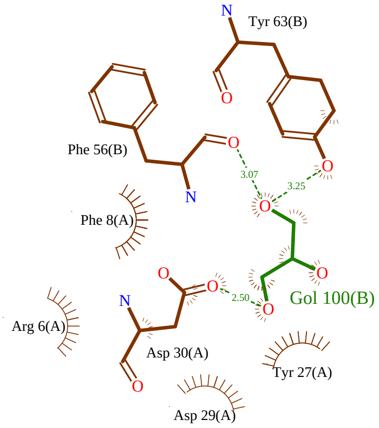

protein	amino acids contacts (binding site)
PDB:3h9h:chainA:P38110	R F Y D D
tr:E9PRG7:E9PRG7_HUMAN	- - - - -
tr:M0QXY8:M0QXY8_HUMAN	- - - - -
tr:E9PIQ5:E9PIQ5_HUMAN	- - - - -
tr:H0YDU7:H0YDU7_HUMAN	- - - - -
tr:E9PIN0:E9PIN0_HUMAN	- - - - -
sp:Q13315:ATM_HUMAN	- - - - -
tr:D4ACL8:D4ACL8_RAT	- - - - -
tr:D3Z0Q2:D3Z0Q2_MOUSE	- - - - -
tr:Q6NX74:Q6NX74_MOUSE	- - - - -
tr:B9EHX4:B9EHX4_MOUSE	- - - - -
sp:Q62388:ATM_MOUSE	- - - - -
tr:A2RVG1:A2RVG1_DROME	L R - - -
sp:Q9N3Q4:ATM_CAEEL	- - A I F
sp:P38110:ATM_YEAST	- - - - -

	whole protein		domain-based		contact-based	
protein	ident	simil	ident	simil	ident	simil
PDB:3h9h:chainA:P38110	0.09	0.53	0.04	0.46	1.0	1.0
tr:E9PRG7:E9PRG7_HUMAN	0.0	0.02	0.08	0.33	0.0	0.0
tr:M0QXY8:M0QXY8_HUMAN	0.01	0.03	0.13	0.5	0.0	0.0
tr:E9PIQ5:E9PIQ5_HUMAN	0.01	0.03	0.09	0.41	0.0	0.0
tr:H0YDU7:H0YDU7_HUMAN	0.01	0.02	0.0	0.0	0.0	0.0
tr:E9PIN0:E9PIN0_HUMAN	0.06	0.23	0.15	0.62	0.0	0.0
sp:Q13315:ATM_HUMAN	0.15	0.51	0.15	0.62	0.0	0.0
tr:D4ACL8:D4ACL8_RAT	0.15	0.51	0.16	0.62	0.0	0.0
tr:D3Z0Q2:D3Z0Q2_MOUSE	0.01	0.03	0.14	0.52	0.0	0.0
tr:Q6NX74:Q6NX74_MOUSE	0.08	0.2	0.0	0.0	0.0	0.0
tr:B9EHX4:B9EHX4_MOUSE	0.15	0.51	0.16	0.62	0.0	0.0
sp:Q62388:ATM_MOUSE	0.15	0.51	0.16	0.62	0.0	0.0
tr:A2RVG1:A2RVG1_DROME	0.04	0.16	0.15	0.56	0.0	0.0
sp:Q9N3Q4:ATM_CAEEL	0.12	0.45	0.1	0.39	0.0	0.0
sp:P38110:ATM_YEAST	1.0	1.0	1.0	1.0	0.0	0.0

atm-1 (WBGene00000227) associated phenotypes

radiation induced reproductive cell death variant, tunicamycin hypersensitive

(Information from WormBase)

atm-1 (UniProt:Q9N3Q4) annotation

Function: Serine/threonine protein kinase which activates checkpoint signaling in the presence of DNA double strand breaks (DSBs) and other forms of DNA damage induced by ionizing radiation and other genotoxic stresses such as UV. Plays a role in maintaining genome stability. (PubMed:16824923, PubMed:17347667, PubMed:22350747).

Subcellular location: Nucleus ECO:0000250—UniProtKB:Q13315.

Disruption phenotype: Increased sensitivity to ionizing radiation with reduced survival compared to wild-type animals. Loss of induction of apoptosis following UV-C or ionizing radiation treatment but no effect on apoptotic response induced by X-ray exposure. Low brood size, reduced viability and sterility, appearance of a high-incidence-of-males (Him) phenotype, and reduced chromosome number due to chromosome fusions. (PubMed:17347667, PubMed:22350747).

(Information from UniProt)

DNA mismatch repair protein MLH1

Best gene implication in ageing for this target family came from gene Q5GJ64 via mapping the annotation from Ensembl ENSG00000076242 via mapping the annotation from EntrezGene 4292 via mapping the annotation from GenAgeHuman 0243 annotated in GenAge release 17.

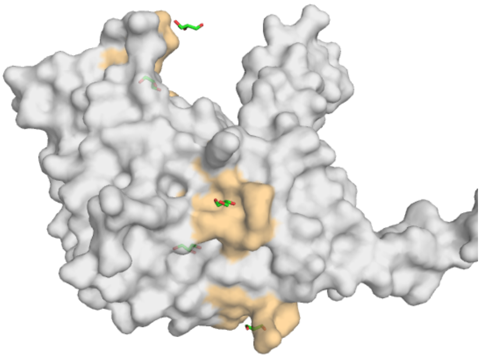

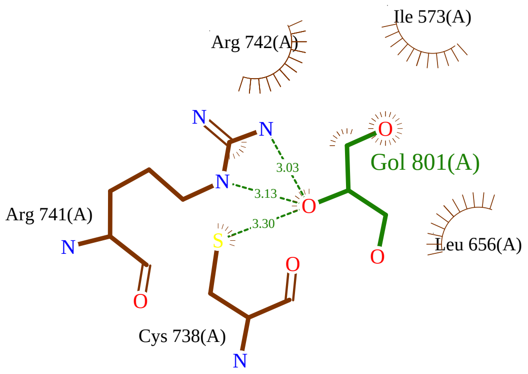

protein	amino acids contacts (binding site)
PDB:4e4w:chainA:P38920	I H R E Y E Y I N D D I V N L E L D R C R R R H
PDB:4fmn:chainA:P38920	I H R E Y E Y I N D D I V N L E L D R C R R R H
tr:Q5GJ64:Q5GJ64_HUMAN	G H E V E E Y I A F D L A A L P I E K A S H K H
sp:P40692:MLH1_HUMAN	G H E V E E Y I A F D L A A L P I E K A S H K H
tr:F1LSD8:F1LSD8_RAT	G H E T E E Y I A F D L A A L P I E K A S H K H
sp:Q9JK91:MLH1_MOUSE	C H E T E E Y I A F D L A A L P I E K A S H K H
tr:Q8TON1:Q8TON1_DROME	C S V Q E E Y R L K E L L S L E L E R A K Y P R
tr:A1Z7C1:A1Z7C1_DROME	C S V Q E E Y R L K E L L S L E L E R A K Y P R
tr:Q9XU10:Q9XU10_CAEEL	S S Q S R E Y I P I - I E E L E V R R L R K E H
sp:P38920:MLH1_YEAST	I H R E Y E Y I N D D I V N L E L D R C R R R H

protein	whole protein ident	whole protein simil	domain-based ident	domain-based simil	contact-based ident	contact-based simil
PDB:4e4w:chainA:P38920	1.0	1.0	1.0	1.0	1.0	1.0
PDB:4fmn:chainA:P38920	1.0	1.0	1.0	1.0	1.0	1.0
tr:Q5GJ64:Q5GJ64_HUMAN	0.36	0.73	0.31	0.72	0.29	0.48
sp:P40692:MLH1_HUMAN	0.35	0.73	0.31	0.72	0.29	0.48
tr:F1LSD8:F1LSD8_RAT	0.35	0.73	0.31	0.72	0.29	0.5
sp:Q9JK91:MLH1_MOUSE	0.36	0.73	0.31	0.72	0.29	0.48
tr:Q8T0N1:Q8T0N1_DROME	0.2	0.49	0.28	0.65	0.25	0.49
tr:A1Z7C1:A1Z7C1_DROME	0.32	0.66	0.28	0.65	0.25	0.49
tr:Q9XU10:Q9XU10_CAEEL	0.28	0.7	0.25	0.66	0.38	0.47
sp:P38920:MLH1_YEAST	1.0	1.0	1.0	1.0	1.0	1.0

Mitogen-activated protein kinase 8

Best gene implication in ageing for this target family came from gene Q8WQG9 annotated in UniProt release 2014_02. Annotation GO 8340 (determination of adult lifespan) was Inferred from Mutant Phenotype

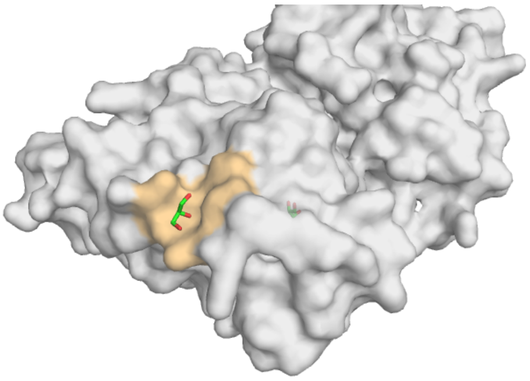

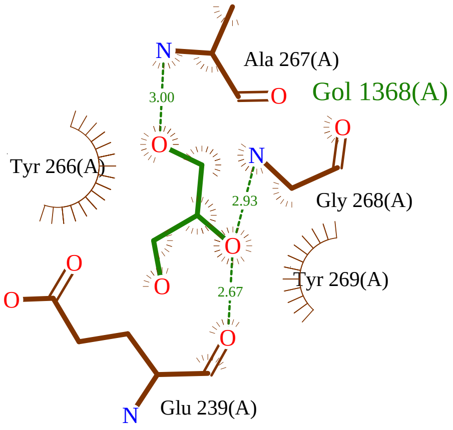

protein	amino acids contacts (binding site)
PDB:2h96:chainA:P45983	V N E L M H I L Y I E T Y A G Y P P P
PDB:2xrw:chainA:P45983	V N E L M H I L H I E T Y A G Y P P P
PDB:3vuh:chainA:P45983	V N E L M H I L H I E T Y A G Y P P P
sp:P45983:MK08_HUMAN	V N E L M H I L Y I E T Y A G Y P P P
tr:F1LP66:F1LP66_RAT	V N E L M H I L N I E T Y A G Y P P P
tr:D4A5V8:D4A5V8_RAT	V N E L M H I L Y I E T Y P G I P P P
tr:B0VXR6:B0VXR6_RAT	V N E L M H I L Y I E T Y A G L P P P
tr:D3ZQ33:D3ZQ33_RAT	V N E L M H I L Y I E T Y A G L P P P
sp:Q91Y86:MK08_MOUSE	V N E L M H I L Y I E T Y A G Y P P P
tr:Q544A0:Q544A0_MOUSE	V N E L M H I L Y I E T Y A G Y P P P
tr:Q7TSJ7:Q7TSJ7_MOUSE	V N E L M H I L Y I E T Y A G Y P P P
tr:E1JHD6:E1JHD6_DROME	V N E L M H I L H I E T Y T G Y P A P
sp:P92208:JNK_DROME	V N E L M H I L H I E T Y T G Y P A P
sp:Q8WQG9:JNK1_CAEEL	V N E L M H I L H I E T Y Q A T P P P
sp:P32485:HOG1_YEAST	L R E L Q Y M L H V D N R D P I A D A

protein	whole protein ident	simil	domain-based ident	simil	contact-based ident	simil
PDB:2h96:chainA:P45983	0.99	1.0	0.99	1.0	1.0	1.0
PDB:2xrw:chainA:P45983	0.97	0.99	0.97	0.99	0.95	0.96
PDB:3vuh:chainA:P45983	0.97	0.99	0.96	0.98	0.95	0.96
sp:P45983:MK08_HUMAN	1.0	1.0	1.0	1.0	1.0	1.0
tr:F1LP66:F1LP66_RAT	0.91	0.94	0.93	0.96	0.95	0.93
tr:D4A5V8:D4A5V8_RAT	0.82	0.94	0.87	0.97	0.89	0.97
tr:B0VXR6:B0VXR6_RAT	0.91	0.97	0.94	0.98	0.95	0.98
tr:D3ZQ33:D3ZQ33_RAT	0.83	0.89	0.94	0.98	0.95	0.98
sp:Q91Y86:MK08_MOUSE	0.89	0.9	1.0	1.0	1.0	1.0
tr:Q544A0:Q544A0_MOUSE	0.89	0.9	1.0	1.0	1.0	1.0
tr:Q7TSJ7:Q7TSJ7_MOUSE	0.99	1.0	1.0	1.0	1.0	1.0
tr:E1JHD6:E1JHD6_DROME	0.66	0.81	0.79	0.94	0.84	0.91
sp:P92208:JNK_DROME	0.66	0.81	0.79	0.94	0.84	0.91
sp:Q8WQG9:JNK1_CAEEL	0.48	0.64	0.69	0.9	0.79	0.83
sp:P32485:HOG1_YEAST	0.31	0.69	0.35	0.71	0.16	0.6

bsk (FBgn0000229) associated phenotypes

RU486 conditional, cell death defective, chemical resistant, chemical sensitive, conditional, decreased cell growth, dominant, heat sensitive, increased cell death, long lived, neuroanatomy defective, neurophysiology defective, nutrition conditional, oxidative stress response defective, planar polarity defective, progressive, short lived, somatic clone, starvation stress response defective, stress response defective, wound healing defective

(Information from FlyBase)

bsk (UniProt:P92208) annotation

Function: Responds to activation by environmental stress by phosphorylating a number of transcription factors, primarily components of AP-1 such as Jra and also the transcriptional repressor aop, and thus regulates transcriptional activity. Component of the immune response activated by bacterial infection, and is involved in wound healing and in dorsal closure, a morphogenetic movement during embryogenesis. Controls the expression of a phosphatase, puckered, at the edges of wounded epidermal tissue and in the dorsal epithelium during dorsal closure. (PubMed:10433922, PubMed:11784101, PubMed:8946915, PubMed:9224720).

Cofactor:Mg(2+)

Enzyme regulation: Activated by threonine and tyrosine phosphorylation by the dual specificity kinase, hep. Inhibited by dual specificity phosphatase, puckered. (PubMed:10433922).

Subcellular location: Cytoplasm

Tissue specificity: During gastrulation, expression is seen in cells undergoing morphogenetic movements. By stage 9 of embryonic development, expression is ubiquitous. At stages 12-14, expression occurs in epidermis and central nervous system. At stage 15, expression is restricted to ventral nerve cord, brain and some peripheral neurons. In larvae, expression is seen in all imaginal disks, with highest levels in wing and eye disks, and in the CNS. Adults express the protein in fat body and hemocytes. (PubMed:8946915, PubMed:8946916).

Developmental stage: Expressed maternally and zygotically through to adult (male and female). (PubMed:8946915).

Domain: The TXY motif contains the threonine and tyrosine residues whose phosphorylation activates the MAP kinases.

Ptm: Dually phosphorylated on Thr-181 and Tyr-183, which activates the enzyme.

(Information from UniProt)

jnk-1 (WBGene00002178) associated phenotypes

aldicarb hypersensitive, pathogen resistance increased, protein phosphorylation reduced, shortened life span, thermotolerance reduced

(Information from WormBase)

jnk-1 (UniProt:Q8WQG9) annotation

Function: Responds to activation by environmental stress by phosphorylating a number of transcription factors, and thus regulates transcriptional activity (By similarity). May coordinate locomotion by acting via type-D GABAergic motor neurons and regulate synaptic vesicle transport in conjunction with unc-16. (, PubMed:10393177, PubMed:11738026).

Cofactor:Mg(2+) Evidence=(PubMed:10393177);

Enzyme regulation: Activated by threonine and tyrosine phosphorylation by either of the dual specificity kinases, jkk-1 and sek-1. (PubMed:10393177).

Subunit: Binds to the scaffolding protein, unc-16. Unc-16 also binds other components of the JNK signaling pathway. (PubMed:11738026).

Domain: The TXY motif contains the threonine and tyrosine residues whose phosphorylation activates the MAP kinases.

Ptm: Dually phosphorylated on Thr-276 and Tyr-278, which activates the enzyme.

(Information from UniProt)

Neurogenic locus notch homolog protein 1

Best gene implication in ageing for this target family came from gene P07207 annotated in UniProt release 2014_02. Annotation GO 8340 (determination of adult lifespan) was Inferred from Mutant Phenotype

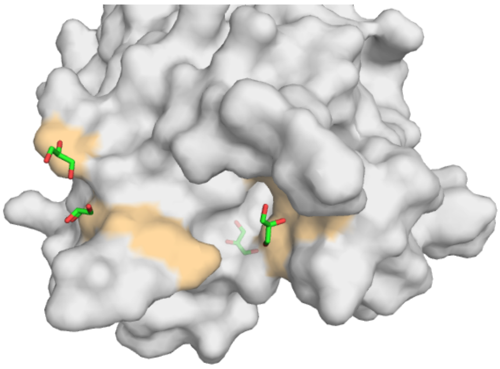

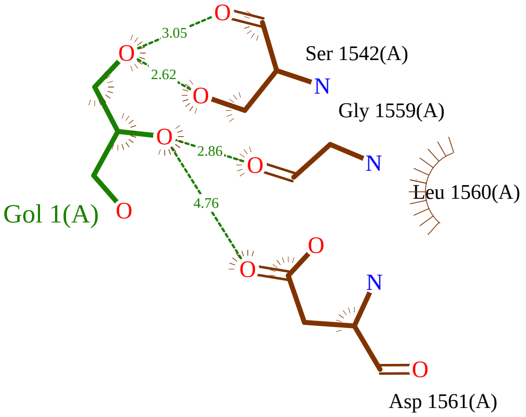

protein	amino acids contacts (binding site)
PDB:3eto:chainA:P46531	S G L D R M I P Y K V
sp:P46531:NOTC1_HUMAN	S G L D R M I P Y K V
sp:Q07008:NOTC1_RAT	S G L D R M I P Y K V
sp:Q01705:NOTC1_MOUSE	S G L D R M I P Y K V
tr:Q4QQB7:Q4QQB7_DROME	- - - - N I Q I H S D
sp:P07207:NOTCH_DROME	- - - - N I Q I H S D

	whole protein		domain-based		contact-based	
protein	ident	simil	ident	simil	ident	simil
PDB:3eto:chainA:P46531	1.0	1.0	1.0	1.0	1.0	1.0
sp:P46531:NOTC1_HUMAN	1.0	1.0	1.0	1.0	1.0	1.0
sp:Q07008:NOTC1_RAT	0.91	0.97	0.93	0.98	1.0	1.0
sp:Q01705:NOTC1_MOUSE	0.91	0.97	0.93	0.98	1.0	1.0
tr:Q4QQB7:Q4QQB7_DROME	0.12	0.3	0.05	0.45	0.0	0.06
sp:P07207:NOTCH_DROME	0.3	0.63	0.05	0.45	0.0	0.06

N (FBgn0004647) associated phenotypes

cell adhesion defective, cell non-autonomous, cell shape defective, chemosensitive behavior defective, cold sensitive, courtship behavior defective, decreased cell death, decreased cell number, dominant, flightless, germline clone, gravitaxis defective, heat sensitive, homeotic, hyperplasia, immune re-

sponse defective, increased cell death, increased cell number, lethal - all die before end of P-stage, lethal - all die before end of pupal stage, lethal - all die during pharate adult stage, maternal effect, memory defective, mitotic cell cycle defective, neuroanatomy defective, neurogenic phenotype, partially, partially lethal - majority die, planar polarity defective, poor, reduced, short lived, size defective, somatic clone, some die during pharate adult stage, some die during pupal stage, sterile, temperature conditional

(Information from FlyBase)

N (UniProt:P07207) annotation

Function: Signaling protein, which regulates, with both positive and negative signals, the differentiation of at least central and peripheral nervous system and eye, wing disk, oogenesis, segmental appendages such as antennae and legs, and muscles, through lateral inhibition or induction. Functions as a receptor for membrane- bound ligands Delta and Serrate to regulate cell-fate determination. Upon ligand activation, and releasing from the cell membrane, the Notch intracellular domain (NICD) forms a transcriptional activator complex with Su(H) (Suppressor of hairless) and activates genes of the E(spl) complex. Essential for proper differentiation of ectoderm. Fringe (fng) acts in the Golgi to determine the type of O-linked fucose on the EGF modules in N, altering the ability of N to bind with Delta (Dl). O-fut1 also has a role in modulating the interaction. Rumi acts in the endoplasmic reticulum to glucosylate the EGF modules in N, this is required for correct folding and cleavage of N. (PubMed:10935637, PubMed:18243100).
Subunit: Homomer. Interacts with Su(H) when activated. Interacts with Dx via its ANK repeats. Interacts with Dl via the EGF repeats and the Dl EGF repeats. Interacts with Nedd4 and Su(dx). Interacts with O-fut1; the interaction glycosylates N and transports N to early endosomes. (PubMed:10935637, PubMed:14573873, PubMed:15620649, PubMed:15620650, PubMed:17329366, PubMed:7671825, PubMed:8162848).
Subcellular location: Cell membrane ECO:0000269—PubMed:17329366; Single-pass type I membrane protein (PubMed:17329366). Endosome (PubMed:17329366). Note=Transported to early endosomes by O-fut1.
Subcellular location: Processed neurogenic locus Notch protein: Nucleus. Note=Upon activation and S3 cleavage, it is released from the cell membrane and enters into the nucleus in conjunction with Su(H).
Domain: Crystal structure of the ANK repeat domain shows that there are 7 repeats and the stabilizing C-terminal repeat enhances the protein stability by extending the ankyrin domain. (PubMed:14573873).
Ptm: Upon binding its ligands such as Delta or Serrate, it is cleaved (S2 cleavage) in its extracellular domain, close to the transmembrane domain. S2 cleavage is probably mediated by Kuz. It is then cleaved (S3 cleavage) downstream of its transmembrane domain, releasing it from the cell membrane. S3 cleavage requires Psn.
Ptm: O-glycosylated. O-fucosylated by O-fut1 in the EGF repeat doamin which inhibits both Serrate/Ser- and Delta/Dl-binding. (PubMed:12909620).
Ptm: Ubiquitinated by Nedd4; which promotes ligand-independent endocytosis and proteasomal degradation. May also be ubiquitinated by Su(dx). (PubMed:15620649).

(Information from UniProt)

Transcription initiation factor TFIID subunit 1

Best gene implication in ageing for this target family came from gene P21675 via mapping the annotation from Ensembl ENSG00000147133 via mapping the annotation from EntrezGene 6872 via mapping the annotation from GenAgeHuman 0180 annotated in GenAge release 17.

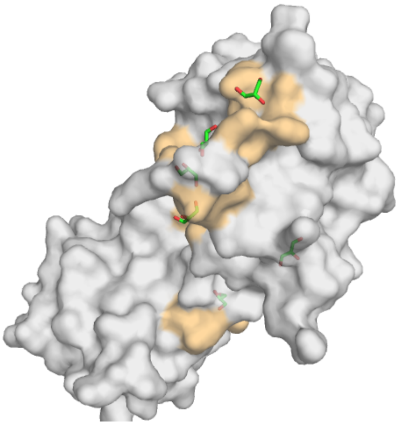

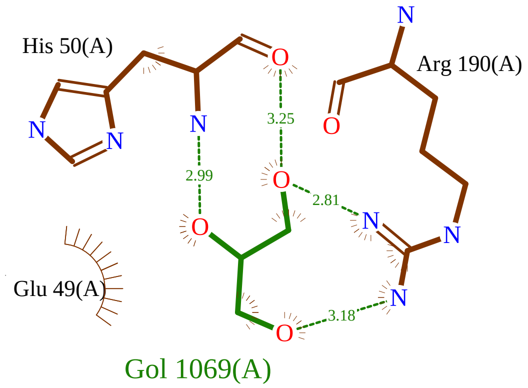

protein	amino acids contacts (binding site)
PDB:4b0a:chainA:P46677	Y F E F I G I E A N D Y E H N R K T T T G Q K V T G
sp:P21675:TAF1_HUMAN	H - G L L G I A L N G W R S C P - E K A E G E C T G
tr:D3ZJI8:D3ZJI8_RAT	H - G L L G I T L N G W R S C P S E K S E G E C T G
tr:S4R1B9:S4R1B9_MOUSE	H - G L L G I A L N G W R S C P S E K S E G E C T G
tr:B1Q2W7:B1Q2W7_MOUSE	H - G L L G I A L N G W R S C P S E K S E G E C T G
sp:Q80UV9:TAF1_MOUSE	H - G L L G I A L N G W R S C P S E K S E G E C T G
tr:Q29R44:Q29R44_DROME	N - S L L D L G V R G A K A - P - - - S K Q E C T G
sp:P51123:TAF1_DROME	N - S L L D L G V R G A K A - P - - - S K Q E C T G
tr:G5EGM3:G5EGM3_CAEEL	C S G S M E V - - - E F E Y - - - - - - - N E C T G
sp:P46677:TAF1_YEAST	Y F E F I G I E G N D Y E H T Q T E T G G G E I T G

	whole protein		domain-based		contact-based	
protein	ident	simil	ident	simil	ident	simil
PDB:4b0a:chainA:P46677	0.43	0.79	0.44	0.8	1.0	1.0
sp:P21675:TAF1_HUMAN	0.15	0.37	0.24	0.58	0.19	0.42
tr:D3ZJI8:D3ZJI8_RAT	0.15	0.37	0.24	0.6	0.19	0.47
tr:S4R1B9:S4R1B9_MOUSE	0.17	0.41	0.24	0.6	0.19	0.46
tr:B1Q2W7:B1Q2W7_MOUSE	0.15	0.38	0.24	0.6	0.19	0.46
sp:Q80UV9:TAF1_MOUSE	0.15	0.38	0.24	0.6	0.19	0.46
tr:Q29R44:Q29R44_DROME	0.12	0.35	0.19	0.54	0.12	0.27
sp:P51123:TAF1_DROME	0.11	0.33	0.19	0.54	0.12	0.27
tr:G5EGM3:G5EGM3_CAEEL	0.12	0.33	0.19	0.5	0.12	0.04
sp:P46677:TAF1_YEAST	1.0	1.0	1.0	1.0	0.65	0.81

Taf1 (FBgn0010355) associated phenotypes

cell autonomous, neuroanatomy defective, partially lethal - majority die, some die during pupal stage

(Information from FlyBase)

Taf1 (UniProt:P51123) annotation

Function: TFIID is a multimeric protein complex that plays a central role in mediating promoter responses to various activators and repressors. Largest component and core scaffold of the complex. Contains N- and C-terminal Ser/Thr kinase domains which can autophosphorylate or transphosphorylate other transcription factors. Possesses DNA-binding activity. Essential for progression of the G1 phase of the cell cycle. Negative regulator of the TATA box-binding activity of Tbp. (PubMed:8504928, PubMed:8625415, PubMed:9741622).
Cofactor:Mg(2+)
Enzyme regulation: Autophosphorylates on Ser residues.
Subunit: Belongs to the TFIID complex which is composed of TATA binding protein (Tbp) and a number of TBP-associated factors (Tafs). Taf1 is the largest component of the TFIID complex. Interacts with Tbp, Taf2, Taf4 and Taf6. (PubMed:8178153, PubMed:8262073, PubMed:8327460, PubMed:8464492).
Subcellular location: Nucleus.

Caution: The C-terminal Ser/Thr kinase domain was reported tophosphorylate histone H2B at 'Ser-33' (PubMed:15143281). However, the paper was retracted because some data, results and conclusions in the paper are not reliable. (PubMed:15143281).

(Information from UniProt)

taf-1 (WBGene00006382) associated phenotypes

lethal, sterile

(Information from WormBase)

Xanthine dehydrogenase/oxidase

Best gene implication in ageing for this target family came from gene P10351 annotated in UniProt release 2014_02. Annotation GO 8340 (determination of adult lifespan) was Inferred from Mutant Phenotype

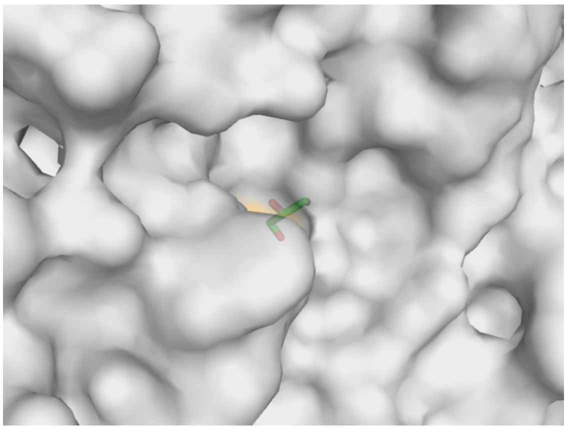

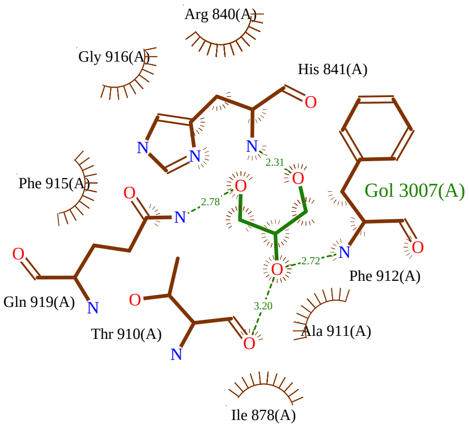

protein	amino acids contacts (binding site)
PDB:2ckj:chainA:P47989	R H I T A F F G Q
sp:P47989:XDH_HUMAN	R H I T A F F G Q
tr:F1LQS6:F1LQS6_RAT	R H I T A F F G Q
sp:Q00519:XDH_MOUSE	R H I T A F F G Q
sp:P10351:XDH_DROME	R H V T A F F G Q
tr:O17892:O17892_CAEEL	R H V T A F F G Q

	whole protein		domain-based		contact-based	
protein	ident	simil	ident	simil	ident	simil
PDB:2ckj:chainA:P47989	1.0	1.0	1.0	1.0	1.0	1.0
sp:P47989:XDH_HUMAN	1.0	1.0	1.0	1.0	1.0	1.0
tr:F1LQS6:F1LQS6_RAT	0.9	0.97	0.91	0.97	1.0	1.0
sp:Q00519:XDH_MOUSE	0.89	0.97	0.89	0.97	1.0	1.0
sp:P10351:XDH_DROME	0.52	0.83	0.56	0.86	0.89	0.97
tr:O17892:O17892_CAEEL	0.46	0.78	0.48	0.81	0.89	0.97

ry (FBgn0003308) associated phenotypes

chemical conditional, chemical sensitive, heat sensitive, partially, partially lethal, partially lethal - majority die, radiation sensitive, short lived

(Information from FlyBase)

ry (UniProt:P10351) annotation

Function: Key enzyme in purine degradation. Catalyzes the oxidation of hypoxanthine to xanthine. Catalyzes the oxidation of xanthine to uric acid (By similarity).
Cofactor: [2Fe-2S] cluster Evidence=; Note=Binds 2 [2Fe-2S] clusters. ;
Cofactor: FAD

Cofactor: Mo-molybdopterin Evidence=; Note=Binds 1 Mo-molybdopterin (Mo-MPT) cofactor per subunit. ;
Subunit: Homodimer.
Subcellular location: Peroxisome

(Information from UniProt)

Glycogen synthase kinase-3 beta

Best gene implication in ageing for this target family came from gene P38615 via mapping the annotation from Ensembl YMR139W via mapping the annotation from EntrezGene 855170 via mapping the annotation from GenAgeModels 1320 annotated in GenAge release 17.

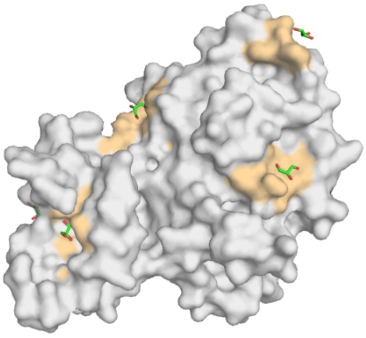

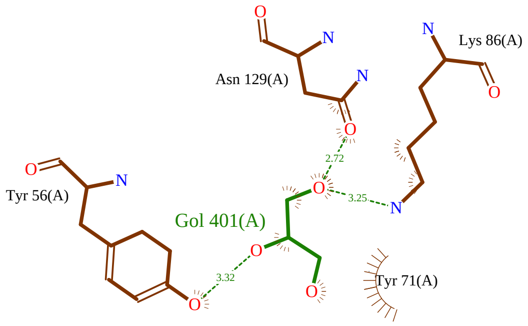

protein	amino acids contacts (binding site)
PDB:3zrk:chainA:P49841	- D R P Y T Y K R S N R A P V R K N V S Y L I G L Q P I K K F P H V R P R R T
PDB:3zrl:chainA:P49841	- D R P Y T Y K R S N R A P V R K N V S Y L I G L Q P I K K F P H V R P R R T
PDB:3zrm:chainA:P49841	- D R P Y T Y K R S N R A P V R K N V S Y L I G L Q P I K K F P H V R P R R T
PDB:4afj:chainA:P49841	- D R P Y T Y K R S N R A P V R K N V S Y L I G L Q P I K K F P H V R P R R T
PDB:4nm0:chainA:P49841	M D R P Y T Y K R S N R A P V R K N V S Y L I G L Q P I K K F P H V R P R R T
PDB:4nm3:chainA:P49841	M D R P Y T Y K R S N R A P V R K N V S Y L I G L Q P I K K F P H V R P R R T
PDB:4nm5:chainA:P49841	M D R P Y T Y K R S N R A P V R K N V S Y L I G L Q P I K K F P H V R P R R T
PDB:4nm7:chainA:P49841	M D R P Y T Y K R S N R A P V R K N V S Y L I G L Q P I K K F P H V R P R R T
tr:Q6FI27:Q6FI27_HUMAN	M D R P Y T Y K R S N R A P V R K N V S Y L I G L Q P I K K F P H V R P R R T
sp:P49841:GSK3B_HUMAN	M D R P Y T Y K R S N R A P V R K N V S Y L I G L Q P I K K F P H V R P R R T
sp:P18266:GSK3B_RAT	M D R P Y T Y K R S N R A P V R K N V S Y L I G L Q P I K K F P H V R P R R T
tr:Q5KU03:Q5KU03_MOUSE	M D R P Y T Y K R S N R A P V R K N V S Y L I G L Q P I K K F P H V R P R R T
sp:Q9WV60:GSK3B_MOUSE	M D R P Y T Y K R S N R A P V R K N V S Y L I G L Q P I K K F P H V R P R R T
tr:E9QAQ5:E9QAQ5_MOUSE	M D R P Y T Y K R S N R A P V R K N V S Y L I G L Q P I K K F P H V R P R R T
tr:B6IDN4:B6IDN4_DROME	G D R V Y T F K R S N K T P I R K N V S Y L I G L Q P I K K F P H V R I R L S
sp:P18431:SGG_DROME	G D R V Y T F K R S N K T P I R K N V S Y L I G L Q P I K K F P H V R I R L S
tr:A4V3W2:A4V3W2_DROME	G D R V Y T F K R S N K T P I R K N V S Y L I G L Q P I K K F P H V R I R L S
tr:A4V3W1:A4V3W1_DROME	G D R V Y T F K R S N K T P I R K N V S Y L I G L Q P I K K F P H V R I R L S
tr:Q0KHW6:Q0KHW6_DROME	A D R V Y T F K R S N K T P I R K N V S Y L I G L Q P I K K F P H V R I R L S
tr:H5V852:H5V852_DROME	F D R V Y T F K R S N K T P I R K N V S Y L I G L Q P I K K F P H V R I R L S
sp:Q9U2Q9:GSK3_CAEEL	L D Q Q Y Q F K R S N K Q P M R K N V S Y L I G L Q P I K K F P H V R V H K T
sp:P38615:RIM11_YEAST	P N D P F T F K R E N H Q S R R K N V S Y L I G L Q P M K K F P I V K - K D L

protein	whole protein ident	simil	domain-based ident	simil	contact-based ident	simil
PDB:3zrk:chainA:P49841	1.0	1.0	1.0	1.0	0.98	0.95
PDB:3zrl:chainA:P49841	1.0	1.0	1.0	1.0	0.98	0.95
PDB:3zrm:chainA:P49841	1.0	1.0	1.0	1.0	0.98	0.95
PDB:4afj:chainA:P49841	1.0	1.0	1.0	1.0	0.98	0.95
PDB:4nm0:chainA:P49841	1.0	1.0	1.0	1.0	1.0	1.0
PDB:4nm3:chainA:P49841	0.98	0.99	0.98	0.99	1.0	1.0
PDB:4nm5:chainA:P49841	1.0	1.0	1.0	1.0	1.0	1.0
PDB:4nm7:chainA:P49841	1.0	1.0	1.0	1.0	1.0	1.0
tr:Q6FI27:Q6FI27_HUMAN	1.0	1.0	1.0	1.0	1.0	1.0
sp:P49841:GSK3B_HUMAN	1.0	1.0	1.0	1.0	1.0	1.0
sp:P18266:GSK3B_RAT	0.99	1.0	1.0	1.0	1.0	1.0
tr:Q5KU03:Q5KU03_MOUSE	0.99	1.0	1.0	1.0	1.0	1.0
sp:Q9WV60:GSK3B_MOUSE	0.99	1.0	1.0	1.0	1.0	1.0
tr:E9QAQ5:E9QAQ5_MOUSE	0.96	0.97	0.96	0.96	1.0	1.0
tr:B6IDN4:B6IDN4_DROME	0.58	0.72	0.78	0.91	0.77	0.86
sp:P18431:SGG_DROME	0.58	0.73	0.78	0.92	0.77	0.86
tr:A4V3W2:A4V3W2_DROME	0.58	0.73	0.78	0.92	0.77	0.86
tr:A4V3W1:A4V3W1_DROME	0.52	0.65	0.78	0.92	0.77	0.86
tr:Q0KHW6:Q0KHW6_DROME	0.5	0.63	0.78	0.93	0.77	0.87
tr:H5V852:H5V852_DROME	0.28	0.35	0.72	0.85	0.77	0.88
sp:Q9U2Q9:GSK3_CAEEL	0.6	0.76	0.72	0.88	0.7	0.85
sp:P38615:RIM11_YEAST	0.43	0.7	0.51	0.8	0.53	0.69

sgg (FBgn0003371) associated phenotypes

cell non-autonomous, cell polarity defective, chemical sensitive, circadian rhythm defective, developmental rate defective, germline clone, heat sensitive, lethal - all die before end of P-stage, lethal - all die before end of pupal stage, maternal effect, meiotic cell cycle defective, mitotic cell cycle defective, neuroanatomy defective, neurophysiology defective, non-rescuable maternal effect, planar polarity defective, rescuable maternal effect, short lived, smell perception defective, somatic clone, some die during pharate adult stage, some die during pupal stage, some die during third instar larval stage

(Information from FlyBase)

sgg (UniProt:P18431) annotation

Function: Required for several developmental events such as syncytial blastoderm formation and embryonic segmentation. Is involved in transcriptional regulation. Required for arm phosphorylation. Wg signaling operates by inactivating the sgg repression of en autoactivation. Negatively controls the neuromuscular junction (NMJ) growth in presynaptic motoneurons. Plays a role in the regulation of microtubule dynamics and actin cytoskeleton during embryogenesis. Required for phosphorylation of sra in activated eggs. Essential for completion of meiosis, possibly by triggering calcineurin activation via sra phosphorylation. Phosphorylates microtubule-associated protein futsch in axons. (PubMed:15269269, PubMed:16570248, PubMed:16949836, PubMed:2113617, PubMed:2118107, PubMed:22421435, PubMed:7529201, PubMed:8467811).
Subunit: Interacts with cos. (PubMed:1335365,PubMed:15691767).
Subcellular location: Cytoplasm. Nucleus. Cytoplasm, cytoskeleton, microtubule organizing center, centrosome. Cytoplasm, cell cortex. Cell junction, synapse. Cell projection, axon. Note=In syncytial embryos, detected at the centrosomes throughout the cell cycle, and in the mitotic spindle and pseudocleavage furrows invaginating from the cell cortex during mitosis. Concentrated at the growing end of membranes during the cellularization process. After cellularization, localized to the centrosomes during mitosis and to the nucleus at the end of telophase. Enriched in the presynaptic side of the neuromuscular junction, with some signal detected also in axonal branches.
Tissue specificity: Expressed in ovaries and activated eggs (at protein level). Expression is over all the embryo at all stages, no local accumulation is observed. (PubMed:2118107, PubMed:22421435, PubMed:8467811).
Developmental stage: Isoform SGG46 is expressed at low levels in 12-24 hours embryos. Isoform Zygotic and isoform SGG39 are expressed in 12-24 hours embryos and present throughout the larval,

pupal and adult stages (at protein level). Isoform Zygotic is expressed maternally and zygotically but reduced throughout later embryonic development. Expression persists throughout larval stages. (PubMed:2118107, PubMed:8467811).
Disruption phenotype: Mutants display an overdeveloped neuromuscular junction (NMJ), with the number of boutons greatly increased. (PubMed:15269269).

(Information from UniProt)

gsk-3 (WBGene00001746) associated phenotypes

axon morphology variant, larval lethal, lethal, locomotion variant, sterile

(Information from WormBase)

gsk-3 (UniProt:Q9U2Q9) annotation

Function: Phosphorylates oma-1, a regulator of the oocyte-to- embryo transition, enabling its degradation. Phosphorylates skn-1, preventing it from accumulating in nuclei and thus inhibiting phase II gene expression in the oxidative stress defense. Involved in mesendoderm specification and mitotic spindle orientation in EMS blastomeres. Thought to be a branch point in these processes as proteins downstream are not required. Negatively regulates Wnt signaling in vulval precursor cells and acts as a Wnt-independent repressor of med-1 and med-2 in the C lineage inhibiting mesoderm development. Required for normal lifespan and LiCl-induced lifespan extension. (PubMed:10444600, PubMed:11463373, PubMed:15572126, PubMed:16251270, PubMed:16289132, PubMed:16343905, PubMed:16930586, PubMed:17959600).
Subunit: Monomer (By similarity). Interacts with axl-1. (UniProtKB:Q9WV60, PubMed:17601533).
Disruption phenotype: Worms exhibit defects in endoderm specification and mitotic spindle alignment. Mutants show reduced degradation of oma-1 and have shortened lifespan. (PubMed:10444600).

(Information from UniProt)

Cyclin-A2

Best gene implication in ageing for this target family came from gene P20248 via mapping the annotation from Ensembl ENSG00000145386 via mapping the annotation from EntrezGene 890 via mapping the annotation from GenAgeHuman 0177 annotated in GenAge release 17.

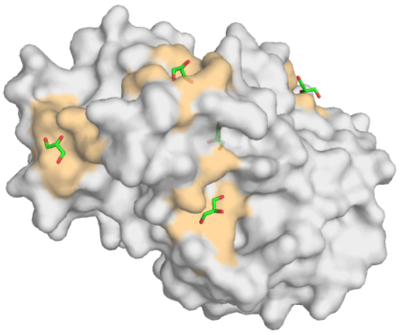

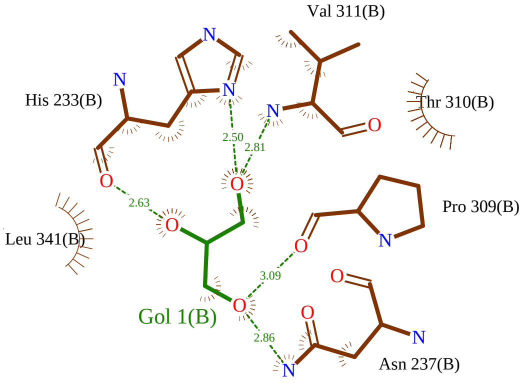

protein	amino acids contacts (binding site)
PDB:3qhr:chainB:P51943	T R Y R Q P N S A I L E Y H N L S S M S G K Q Y E T Y Q P T V L D Q T H Q
PDB:4i3z:chainB:P51943	T R Y R Q P N S A I L E Y H N L S S M S G K Q Y E T Y Q P T V L D Q T H Q
PDB:4ii5:chainB:P51943	T R Y R Q P N S A I L E Y H N L S S M S G K Q Y E T Y Q P T V L D Q T H Q
sp:P20248:CCNA2_HUMAN	T R Y K Q P N S A I L E Y H N L S S M S G K Q Y E T Y Q P T V L D K T H Q
tr:Q6GX89:Q6GX89_RAT	T R Y R Q P N S A I L E Y H N L S S M S G K Q Y E T Y Q P T V L D K T H Q
tr:G3V802:G3V802_RAT	T R Y R Q P N S A I L E Y H N L S S M S G K Q Y E T Y Q P T V L D K T H Q
sp:P51943:CCNA2_MOUSE	T R Y R Q P N S A I L E Y H N L S S M S G K Q Y E T Y Q P T V L D Q T H Q
tr:F3YDG4:F3YDG4_DROME	E R Y R Q K H N S I L E Y Y F L S Q M A S K Q Y E S Y Q P T A L E I T L T
tr:F3YDK3:F3YDK3_DROME	E R Y R Q K H N S I L E Y Y F L S Q M A S K Q Y E S Y Q P T A L E I T L T
sp:P14785:CCNA_DROME	E R Y R Q K H N S I L E Y Y F L S Q M A S K Q Y E S Y Q P T A L E I T L T

	whole protein		domain-based		contact-based	
protein	ident	simil	ident	simil	ident	simil
PDB:3qhr:chainB:P51943	1.0	1.0	1.0	1.0	1.0	1.0
PDB:4i3z:chainB:P51943	1.0	1.0	1.0	1.0	1.0	1.0
PDB:4ii5:chainB:P51943	1.0	1.0	1.0	1.0	1.0	1.0
sp:P20248:CCNA2_HUMAN	0.85	0.94	0.94	0.98	0.95	0.98
tr:Q6GX89:Q6GX89_RAT	0.65	0.67	0.97	0.99	0.97	0.99
tr:G3V802:G3V802_RAT	0.94	0.97	0.97	0.99	0.97	0.99
sp:P51943:CCNA2_MOUSE	1.0	1.0	1.0	1.0	1.0	1.0
tr:F3YDG4:F3YDG4_DROME	0.27	0.47	0.39	0.68	0.57	0.67
tr:F3YDK3:F3YDK3_DROME	0.33	0.62	0.4	0.68	0.57	0.67
sp:P14785:CCNA_DROME	0.33	0.62	0.39	0.68	0.57	0.67

CycA (FBgn0000404) associated phenotypes

cell migration defective, mitotic cell cycle defective, neuroanatomy defective, partially lethal - majority die, reduced, some die during immature adult stage

(Information from FlyBase)

CycA (UniProt:P14785) annotation

Function: Essential for the control of the cell cycle at the G2/M (mitosis) transition. Interacts with the CDC2 and CDK2 protein kinases to form MPF. G2/M cyclins accumulate steadily during G2 and are abruptly destroyed at mitosis.

(Information from UniProt)

Dipeptidyl peptidase 1

Best gene implication in ageing for this target family came from gene P80067 annotated in UniProt release 2014_02. Annotation GO 7568 (aging) was Inferred from Expression Pattern

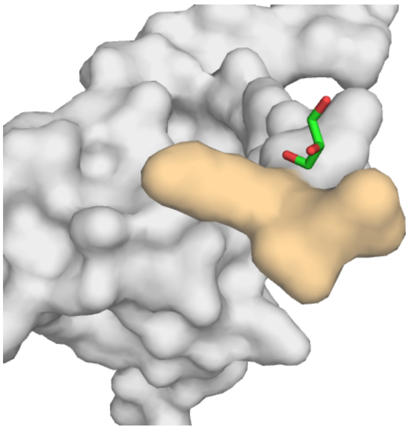

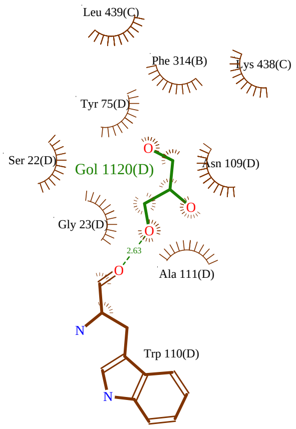

protein	amino acids contacts (binding site)		
PDB:4cdc:chainB:P53634	F	-	-
PDB:4cdc:chainC:P53634	-	K	L
sp:P53634:CATC_HUMAN	F	K	L
sp:P80067:CATC_RAT	L	K	L
tr:Q3U9B7:Q3U9B7_MOUSE	L	K	L
sp:P97821:CATC_MOUSE	L	K	L
tr:Q3UBY5:Q3UBY5_MOUSE	L	K	L
tr:P92005:P92005_CAEEL	T	-	-

	whole protein		domain-based		contact-based	
protein	ident	simil	ident	simil	ident	simil
PDB:4cdc:chainB:P53634	1.0	1.0	1.0	1.0	0.33	0.0
PDB:4cdc:chainC:P53634	1.0	1.0	1.0	1.0	0.67	0.0
sp:P53634:CATC_HUMAN	1.0	1.0	1.0	1.0	1.0	1.0
sp:P80067:CATC_RAT	0.79	0.93	0.83	0.94	0.67	0.9
tr:Q3U9B7:Q3U9B7_MOUSE	0.76	0.9	0.81	0.95	0.67	0.9
sp:P97821:CATC_MOUSE	0.78	0.93	0.81	0.95	0.67	0.9
tr:Q3UBY5:Q3UBY5_MOUSE	0.78	0.93	0.81	0.95	0.67	0.9
tr:P92005:P92005_CAEEL	0.21	0.5	0.28	0.61	0.0	0.0

NAD-dependent protein deacetylase HST2

Best gene implication in ageing for this target family came from gene Q9I7I7 annotated in UniProt release 2014_02. Annotation GO 8340 (determination of adult lifespan) was Inferred from Mutant Phenotype

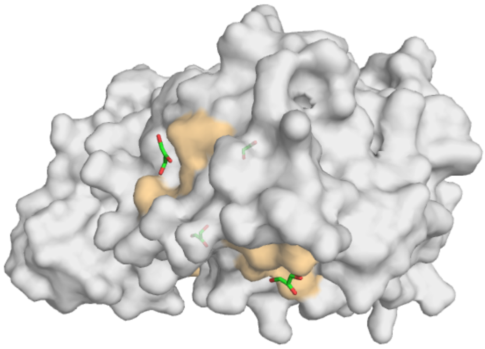

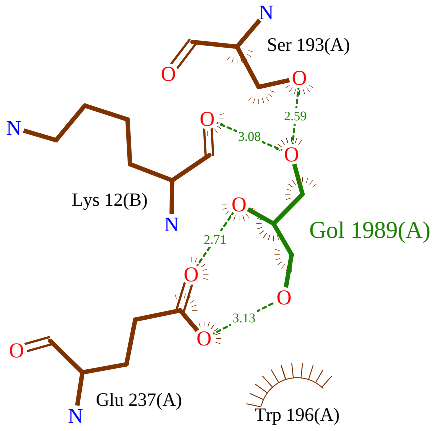

protein	amino acids contacts (binding site)															
PDB:1szc:chainA:P53686	K	T	G	K	Y	N	K	K	L	A	S	W	W	K	S	E
PDB:1szd:chainA:P53686	K	T	G	K	Y	N	K	K	L	A	S	W	W	K	S	E
PDB:2od2:chainA:P53686	K	T	G	K	Y	N	K	K	L	A	S	W	W	K	S	E
sp:Q8IXJ6:SIR2_HUMAN	R	T	G	K	Y	Q	L	K	I	F	F	M	-	-	S	K
sp:Q5RJQ4:SIR2_RAT	R	T	G	K	Y	Q	L	K	I	F	F	M	-	-	S	K
tr:Q3UJK6:Q3UJK6_MOUSE	K	T	G	K	Y	Q	L	K	I	F	F	M	-	-	S	K
sp:Q8VDQ8:SIR2_MOUSE	K	T	G	K	Y	Q	L	K	I	F	F	M	-	-	S	K
sp:Q9I7I7:SIRT2_DROME	K	S	G	K	Y	S	Q	K	I	F	Y	P	-	-	S	R
sp:P53686:HST2_YEAST	K	T	G	K	Y	N	K	K	L	A	S	W	W	K	S	E

protein	whole protein ident	simil	domain-based ident	simil	contact-based ident	simil
PDB:1szc:chainA:P53686	0.99	0.99	1.0	1.0	1.0	1.0
PDB:1szd:chainA:P53686	0.99	0.99	1.0	1.0	1.0	1.0
PDB:2od2:chainA:P53686	0.99	0.99	1.0	1.0	1.0	1.0
sp:Q8IXJ6:SIR2_HUMAN	0.3	0.58	0.41	0.7	0.38	0.43
sp:Q5RJQ4:SIR2_RAT	0.32	0.63	0.4	0.7	0.38	0.43
tr:Q3UJK6:Q3UJK6_MOUSE	0.32	0.63	0.4	0.7	0.44	0.43
sp:Q8VDQ8:SIR2_MOUSE	0.29	0.58	0.4	0.7	0.44	0.43
sp:Q9I7I7:SIRT2_DROME	0.31	0.62	0.4	0.71	0.38	0.42
sp:P53686:HST2_YEAST	1.0	1.0	1.0	1.0	1.0	1.0

Sirt2 (FBgn0038788) associated phenotypes
heat sensitive, lethal - all die before end of pupal stage, some die during pupal stage

(Information from FlyBase)

Sirt2 (UniProt:Q9I7I7) annotation
Function: NAD-dependent protein deacetylase (By similarity). May be involved in the regulation of life span. (, PubMed:17159295).
Cofactor: Zn(2+)Note=Binds 1 zinc ion per subunit. ;
Disruption phenotype: Causes lethality during development. Induced silencing shortens life span. (PubMed:17159295).

(Information from UniProt)

Mitogen-activated protein kinase 10

Best gene implication in ageing for this target family came from gene Q8WQG9 annotated in UniProt release 2014_02. Annotation GO 8340 (determination of adult lifespan) was Inferred from Mutant Phenotype

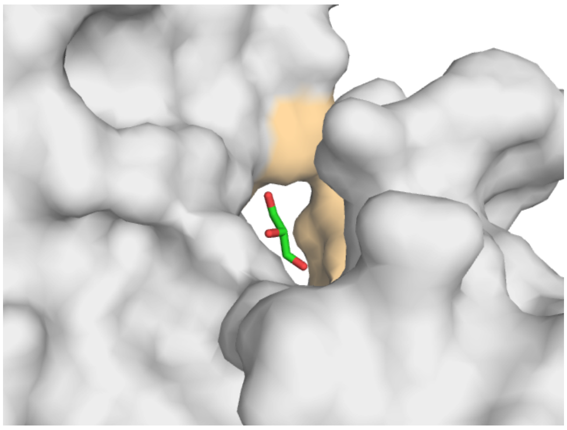

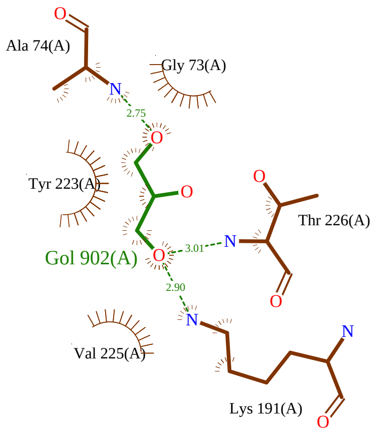

protein	amino acids contacts (binding site)
PDB:2zdt:chainA:P53779	G A D R K N Y V T
PDB:3tti:chainA:P53779	G A D R K N Y V T
sp:P53779:MK10_HUMAN	G A D R K N Y V T
tr:B0VXR6:B0VXR6_RAT	G A D R K N Y V T
tr:D3ZQ33:D3ZQ33_RAT	G A D R K N Y V T
tr:Q3TQZ7:Q3TQZ7_MOUSE	G A D R K N Y V T
tr:Q80W82:Q80W82_MOUSE	G A D R K N Y V T
tr:Q78GB8:Q78GB8_MOUSE	G A D R K N Y V T
tr:Q80W80:Q80W80_MOUSE	G A D R K N Y V T
tr:Q8C9D4:Q8C9D4_MOUSE	G A D R K N Y V T
tr:E9QN59:E9QN59_MOUSE	G A D R K N Y V T
tr:E1JHD6:E1JHD6_DROME	G A Q Q K N Y V T
sp:P92208:JNK_DROME	G A Q Q K N Y V T
sp:Q8WQG9:JNK1_CAEEL	G A N E K N Y V T
sp:P32485:HOG1_YEAST	G A S Q K N Y S T

	whole protein		domain-based		contact-based	
protein	ident	simil	ident	simil	ident	simil
PDB:2zdt:chainA:P53779	1.0	1.0	1.0	1.0	1.0	1.0
PDB:3tti:chainA:P53779	1.0	1.0	1.0	1.0	1.0	1.0
sp:P53779:MK10_HUMAN	1.0	1.0	1.0	1.0	1.0	1.0
tr:B0VXR6:B0VXR6_RAT	0.92	0.92	1.0	1.0	1.0	1.0
tr:D3ZQ33:D3ZQ33_RAT	0.99	1.0	1.0	1.0	1.0	1.0
tr:Q3TQZ7:Q3TQZ7_MOUSE	0.89	0.9	1.0	1.0	1.0	1.0
tr:Q80W82:Q80W82_MOUSE	0.89	0.9	1.0	1.0	1.0	1.0
tr:Q78GB8:Q78GB8_MOUSE	0.92	0.92	1.0	1.0	1.0	1.0
tr:Q80W80:Q80W80_MOUSE	0.98	0.99	0.97	0.99	1.0	1.0
tr:Q8C9D4:Q8C9D4_MOUSE	1.0	1.0	1.0	1.0	1.0	1.0
tr:E9QN59:E9QN59_MOUSE	0.94	0.94	1.0	1.0	1.0	1.0
tr:E1JHD6:E1JHD6_DROME	0.62	0.74	0.84	0.95	0.78	0.88
sp:P92208:JNK_DROME	0.62	0.74	0.84	0.95	0.78	0.88
sp:Q8WQG9:JNK1_CAEEL	0.45	0.6	0.8	0.94	0.78	0.91
sp:P32485:HOG1_YEAST	0.29	0.64	0.47	0.81	0.67	0.73

bsk (FBgn0000229) associated phenotypes

RU486 conditional, cell death defective, chemical resistant, chemical sensitive, conditional, decreased cell growth, dominant, heat sensitive, increased cell death, long lived, neuroanatomy defective, neurophysiology defective, nutrition conditional, oxidative stress response defective, planar polarity defective, progressive, short lived, somatic clone, starvation stress response defective, stress response defective, wound healing defective

(Information from FlyBase)

bsk (UniProt:P92208) annotation

Function: Responds to activation by environmental stress by phosphorylating a number of transcription factors, primarily components of AP-1 such as Jra and also the transcriptional repressor aop, and thus regulates transcriptional activity. Component of the immune response activated by bacterial infection, and is involved in wound healing and in dorsal closure, a morphogenetic movement during embryogenesis. Controls the expression of a phosphatase, puckered, at the edges of wounded epidermal tissue and in the dorsal epithelium during dorsal closure. (PubMed:10433922, PubMed:11784101, PubMed:8946915, PubMed:9224720).
Cofactor:Mg(2+)
Enzyme regulation: Activated by threonine and tyrosine phosphorylation by the dual specificity kinase, hep. Inhibited by dual specificity phosphatase, puckered. (PubMed:10433922).
Subcellular location: Cytoplasm
Tissue specificity: During gastrulation, expression is seen in cells undergoing morphogenetic movements. By stage 9 of embryonic development, expression is ubiquitous. At stages 12-14, expression occurs in epidermis and central nervous system. At stage 15, expression is restricted to ventral nerve cord, brain and some peripheral neurons. In larvae, expression is seen in all imaginal

disks, with highest levels in wing and eye disks, and in the CNS. Adults express the protein in fat body and hemocytes. (PubMed:8946915, PubMed:8946916).
Developmental stage: Expressed maternally and zygotically through to adult (male and female). (PubMed:8946915).
Domain: The TXY motif contains the threonine and tyrosine residues whose phosphorylation activates the MAP kinases.
Ptm: Dually phosphorylated on Thr-181 and Tyr-183, which activates the enzyme.

(Information from UniProt)

jnk-1 (WBGene00002178) associated phenotypes

aldicarb hypersensitive, pathogen resistance increased, protein phosphorylation reduced, shortened life span, thermotolerance reduced

(Information from WormBase)

jnk-1 (UniProt:Q8WQG9) annotation

Function: Responds to activation by environmental stress by phosphorylating a number of transcription factors, and thus regulates transcriptional activity (By similarity). May coordinate locomotion by acting via type-D GABAergic motor neurons and regulate synaptic vesicle transport in conjunction with unc-16. (, PubMed:10393177, PubMed:11738026).
Cofactor:Mg(2+) Evidence=(PubMed:10393177);
Enzyme regulation: Activated by threonine and tyrosine phosphorylation by either of the dual specificity kinases, jkk-1 and sek-1. (PubMed:10393177).
Subunit: Binds to the scaffolding protein, unc-16. Unc-16 also binds other components of the JNK signaling pathway. (PubMed:11738026).
Domain: The TXY motif contains the threonine and tyrosine residues whose phosphorylation activates the MAP kinases.
Ptm: Dually phosphorylated on Thr-276 and Tyr-278, which activates the enzyme.

(Information from UniProt)

Transitional endoplasmic reticulum ATPase

Best gene implication in ageing for this target family came from gene P54811 via mapping the annotation from Ensembl WBGene00007352 via mapping the annotation from EntrezGene 174624 via mapping the annotation from GenAgeModels 1759 annotated in GenAge release 17.

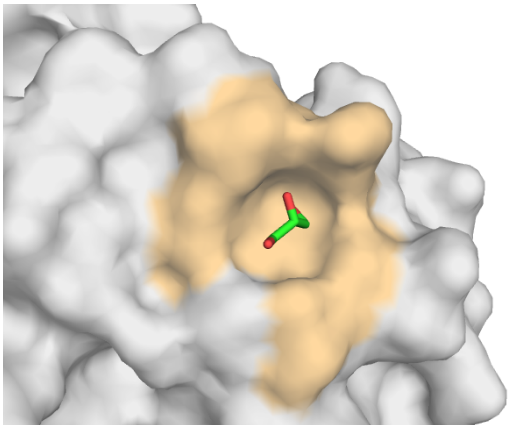

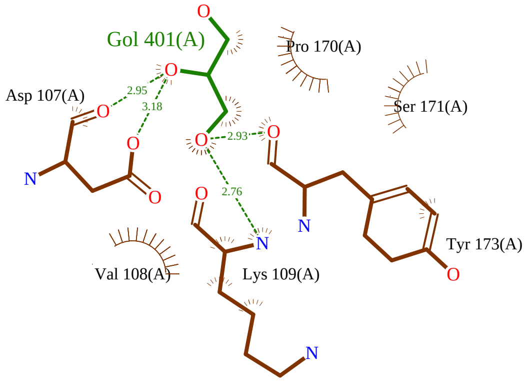

protein	amino acids contacts (binding site)
PDB:3qq7:chainA:P55072	D V K P S Y
tr:C9IZA5:C9IZA5_HUMAN	D V K P S Y
sp:P55072:TERA_HUMAN	D V K P S Y
sp:P46462:TERA_RAT	D V K P S Y
tr:Q8BNF8:Q8BNF8_MOUSE	D V K P S Y
sp:Q01853:TERA_MOUSE	D V K P S Y
sp:Q7KN62:TERA_DROME	D V K P E Y
tr:D0IQG4:D0IQG4_DROME	D V K P E Y
sp:P54811:TERA1_CAEEL	N L S P A A
sp:P54812:TERA2_CAEEL	Q L E P A A
sp:P25694:CDC48_YEAST	D I K P E Y

	whole protein		domain-based		contact-based	
protein	ident	simil	ident	simil	ident	simil
PDB:3qq7:chainA:P55072	1.0	1.0	1.0	1.0	1.0	1.0
tr:C9IZA5:C9IZA5_HUMAN	0.2	0.2	0.78	0.78	1.0	1.0
sp:P55072:TERA_HUMAN	1.0	1.0	1.0	1.0	1.0	1.0
sp:P46462:TERA_RAT	1.0	1.0	1.0	1.0	1.0	1.0
tr:Q8BNF8:Q8BNF8_MOUSE	0.9	0.9	0.58	0.58	1.0	1.0
sp:Q01853:TERA_MOUSE	1.0	1.0	1.0	1.0	1.0	1.0
sp:Q7KN62:TERA_DROME	0.83	0.94	0.77	0.94	0.83	0.88
tr:D0IQG4:D0IQG4_DROME	0.8	0.92	0.75	0.93	0.83	0.88
sp:P54811:TERA1_CAEEL	0.76	0.9	0.7	0.9	0.17	0.49
sp:P54812:TERA2_CAEEL	0.76	0.9	0.69	0.89	0.17	0.58
sp:P25694:CDC48_YEAST	0.66	0.87	0.63	0.88	0.67	0.83

TER94 (FBgn0261014) associated phenotypes

RU486 conditional, decreased cell death, flightless, increased cell death, learning defective, neuroanatomy defective, partially lethal - majority die, somatic clone, some die during pupal stage, stress response defective, temperature conditional

(Information from FlyBase)

TER94 (UniProt:Q7KN62) annotation

Function: Necessary for the fragmentation of Golgi stacks during mitosis and for their reassembly after mitosis. Involved in the formation of the transitional endoplasmic reticulum (tER). The transfer of membranes from the endoplasmic reticulum to the Golgi apparatus occurs via 50-70 nm transition vesicles which derive from part-rough, part-smooth transitional elements of the endoplasmic reticulum (tER). Vesicle budding from the tER is an ATP-dependent process. Involved in the ubiquitin-proteasome system. Important for oskar mRNA localization and/or anchoring during oogenesis. Involved in germ cell formation. (PubMed:14657277, PubMed:18590813).
Subunit: Homohexamer. Interacts with tud, vas, papi and AGO3.(PubMed:10564274, PubMed:18590813, PubMed:21447556).
Subcellular location: Cytoplasm. Nucleus. Note=Component of the meiotic nuage, also named P granule, a germ-cell-specific organelle required to repress transposon activity during meiosis.
Tissue specificity: Present in the mushroom bodies of the protocerebrum and in the glomeruli of the antennal lobe. Present in nurse cells, oocytes and sperm bundles (at protein level). (PubMed:10564274, PubMed:10656772, PubMed:9639875).
Developmental stage: Present in egg, pupa and imago but not larva (at protein level). (PubMed:9639875).

(Information from UniProt)

cdc-48.1 (WBGene00007352) associated phenotypes

tunicamycin hypersensitive, unfolded protein response variant

(Information from WormBase)

cdc-48.2 (WBGene00008053) associated phenotypes

reduced brood size

(Information from WormBase)

Cell division control protein 42 homolog

Best gene implication in ageing for this target family came from gene P60953 via mapping the annotation from Ensembl ENSG00000070831 via mapping the annotation from EntrezGene 998 via mapping the annotation from GenAgeHuman 0250 annotated in GenAge release 17.

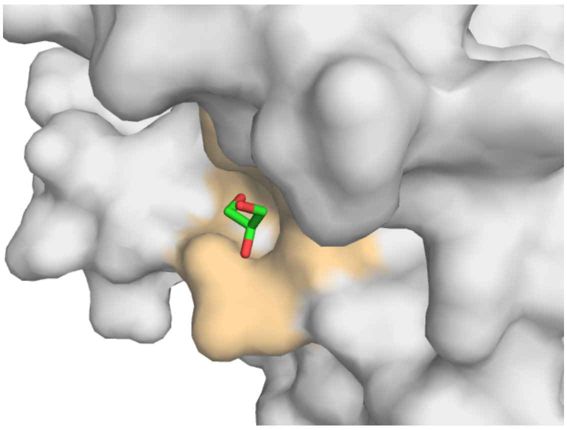

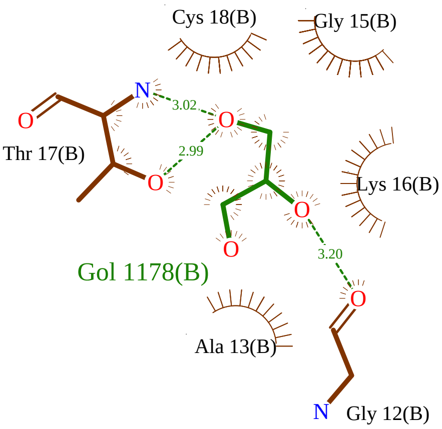

protein	amino acids contacts (binding site)
PDB:2dfk:chainB:P60953	G A V G K T C S Y T N Q K
PDB:2wm9:chainB:P60953	G A V G K T C S Y T N Q K
sp:P60953:CDC42_HUMAN	G A V G K T C S Y T N Q K
sp:Q8CFN2:CDC42_RAT	G A V G K T C S Y T N Q K
sp:P60766:CDC42_MOUSE	G A V G K T C S Y T N Q K
sp:P40793:CDC42_DROME	G A V G K T C S Y T N Q K
sp:Q05062:CDC42_CAEEL	G A V G K T C S Y T N Q K
sp:P19073:CDC42_YEAST	G A V G K T C S Y T N Q K

	whole protein		domain-based		contact-based	
protein	ident	simil	ident	simil	ident	simil
PDB:2dfk:chainB:P60953	1.0	1.0	1.0	1.0	1.0	1.0
PDB:2wm9:chainB:P60953	0.99	0.99	1.0	1.0	1.0	1.0
sp:P60953:CDC42_HUMAN	1.0	1.0	1.0	1.0	1.0	1.0
sp:Q8CFN2:CDC42_RAT	1.0	1.0	1.0	1.0	1.0	1.0
sp:P60766:CDC42_MOUSE	1.0	1.0	1.0	1.0	1.0	1.0
sp:P40793:CDC42_DROME	0.93	0.98	0.95	0.99	1.0	1.0
sp:Q05062:CDC42_CAEEL	0.86	0.97	0.9	0.98	1.0	1.0
sp:P19073:CDC42_YEAST	0.8	0.94	0.82	0.95	1.0	1.0

Cdc42 (FBgn0010341) associated phenotypes

cell polarity defective, cell shape defective, chemical resistant, chemical sensitive, decreased cell size, heat sensitive, immune response defective, lethal - all die before end of pupal stage, maternal effect, neuroanatomy defective, neurophysiology defective, non-rescuable maternal effect, partially, partially lethal - majority die, planar polarity defective, size defective, somatic clone, some die during pupal stage, touch sensitivity defective, wound healing defective

(Information from FlyBase)

Cdc42 (UniProt:P40793) annotation

Function: Regulates mbt kinase activity and is also required to recruit mbt to adherens junctions. Together with mbt, regulates photoreceptor cell morphogenesis. (PubMed:12490550).
Subunit: The GTP-bound but not the GDP-bound form interacts with mbt and gek. (PubMed:12490550, PubMed:9371783).
Subcellular location: Cell junction, adherens junction (PubMed:12490550). Cell membrane

(PubMed:12490550); Lipid-anchor (PubMed:12490550). Note=Adherens junctions of developing photoreceptor cells.

(Information from UniProt)

cdc-42 (UniProt:Q05062) annotation

Function: Plays an essential role in spindle orientation and organizing cellular and embryonic polarity by controlling the localization and activity of PAR (partitioning-defective) proteins. Required for maintaining the asymmetric cortical localization of the anterior complex proteins par-3 and par-6, the posterior cortical protein par-2, and pkc-3 (PubMed:11412996, PubMed:11412997). Involved in hypodermal cell fusion, together with pak-1 and ced-10, leading to embryonic body elongation, which involves dramatic cytoskeletal reorganization (PubMed:8824291). During gonad morphogenesis, plays a role in distal tip cell (DTC)- mediated guidance of gonad elongation, probably by activating max- 2 (PubMed:19797046, PubMed:19023419). (PubMed:11412996, PubMed:11412997, PubMed:19023419, PubMed:19797046, PubMed:8824291).
Subunit: The GTP-bound, but not the GDP-bound, form binds to the p21-activated kinase (pak-1). Interaction with par-6 required for activation of the par-3/par-6/pkc-3 complex. (PubMed:11412997, PubMed:8824291).
Subcellular location: Cell membrane (PubMed:8824291); Lipid-anchor ; Cytoplasmic side (PubMed:8824291). Note=Co-localizes with ced-10/rac-1 and pak-1 at hypodermal cell boundaries during embryo elongation (PubMed:8824291).
Tissue specificity: Colocalizes with pak-1 to hypodermal cell boundaries during embryo elongation throughout the second phase of embryogenesis. (PubMed:8824291).
Developmental stage: Highest levels at the embryonic stage, decreasing progressively during development, except for an increase at the L3 stage. (PubMed:8514766).
Disruption phenotype: RNAi-mediated knockdown in distal tip cells (DTC) causes additional turns during their migration. (PubMed:19023419).

(Information from UniProt)

Serine/threonine-protein phosphatase PP1-alpha catalytic subunit

Best gene implication in ageing for this target family came from gene P62136 via mapping the annotation from Ensembl ENSG00000172531 via mapping the annotation from EntrezGene 5499 via mapping the annotation from GenAgeHuman 0227 annotated in GenAge release 17.

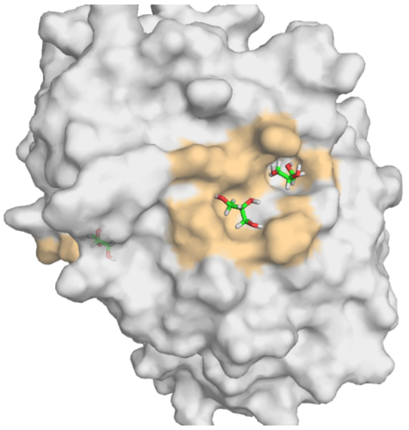

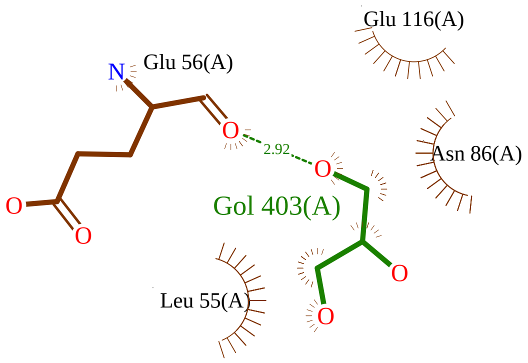

protein	amino acids contacts (binding site)
PDB:3e7a:chainA:P62136	L Q K S Q P L E L E P L K F S N E F C S V P D C W V S V
PDB:3e7b:chainA:P62136	L Q K S Q P L E L E P L K F S N E F C S V P D C W V S V
PDB:3egg:chainA:P62136	L Q K S Q P L E L E P L K F S N E F C S V P D C W V S V
PDB:3egh:chainA:P62136	L Q K S Q P L E L E P L K F S N E F C S V P D C W V S V
PDB:3hvq:chainA:P62136	L Q K S Q P L E L E P L K F S N E F C S V P D C W V S V
PDB:3v4y:chainA:P62136	L Q K S Q P L E L E P L K F S N E F C S V P D C W V S V
PDB:4moy:chainA:P62136	L Q K S Q P L E L E P L K F S N E F C S V P D C W V S V
PDB:4mp0:chainA:P62136	L Q K S Q P L E L E P L K F S N E F C S V P D C W V S V
tr:E9PMD7:E9PMD7_HUMAN	L Q K S Q P L E L E P L K F S N E F C S V P D C W V S V
sp:P62136:PP1A_HUMAN	L Q K S Q P L E L E P L K F S N E F C S V P D C W V S V
sp:P62138:PP1A_RAT	L Q K S Q P L E L E P L K F S N E F C S V P D C W V S V
sp:P62137:PP1A_MOUSE	L Q K S Q P L E L E P L K F S N E F C S V P D C W V S V
sp:P12982:PP12_DROME	L R K S Q P L E L E P L K F S N E F C S V P D C W V S V
sp:P48461:PP11_DROME	L R K S Q P L E L E P L K F S N E F C S V P D C W V S V
tr:Q4W5Q9:Q4W5Q9_CAEEL	- Q N K E P L E I E P V T F S R Q F S P I A D S W A Q V
sp:P32598:PP12_YEAST	L R Q K Q P L E L E P I K F S N E F C S I P D C W V S V

	whole protein		domain-based		contact-based	
protein	ident	simil	ident	simil	ident	simil
PDB:3e7a:chainA:P62136	1.0	1.0	1.0	1.0	1.0	1.0
PDB:3e7b:chainA:P62136	1.0	1.0	1.0	1.0	1.0	1.0
PDB:3egg:chainA:P62136	1.0	1.0	1.0	1.0	1.0	1.0
PDB:3egh:chainA:P62136	1.0	1.0	1.0	1.0	1.0	1.0
PDB:3hvq:chainA:P62136	1.0	1.0	1.0	1.0	1.0	1.0
PDB:3v4y:chainA:P62136	1.0	1.0	1.0	1.0	1.0	1.0
PDB:4moy:chainA:P62136	1.0	1.0	1.0	1.0	1.0	1.0
PDB:4mp0:chainA:P62136	1.0	1.0	1.0	1.0	1.0	1.0
tr:E9PMD7:E9PMD7_HUMAN	0.77	0.77	0.83	0.83	1.0	1.0
sp:P62136:PP1A_HUMAN	1.0	1.0	1.0	1.0	1.0	1.0
sp:P62138:PP1A_RAT	1.0	1.0	1.0	1.0	1.0	1.0
sp:P62137:PP1A_MOUSE	1.0	1.0	1.0	1.0	1.0	1.0
sp:P12982:PP12_DROME	0.85	0.9	0.93	0.98	0.96	0.99
sp:P48461:PP11_DROME	0.86	0.95	0.92	0.98	0.96	0.99
tr:Q4W5Q9:Q4W5Q9_CAEEL	0.45	0.75	0.47	0.77	0.43	0.62
sp:P32598:PP12_YEAST	0.79	0.9	0.85	0.96	0.82	0.92

Pp1-87B (FBgn0004103) associated phenotypes

dominant, mitotic cell cycle defective, non-suppressor of variegation, partially lethal - majority die, somatic clone, suppressor of variegation

(Information from FlyBase)

Pp1-87B (UniProt:P12982) annotation

Function: Is essential for the regulation of mitotic chromosomal segregation as well as regulation of chromatin condensation during interphase. (PubMed:1330679, PubMed:2175717).
Cofactor: Mn(2+)Note=Binds 2 manganese ions per subunit. ;
Subunit: Interacts with Nop17l. (PubMed:17007873).
Subcellular location: Cytoplasm.

(Information from UniProt)

Pp1alpha-96A (FBgn0003134) associated phenotypes

conditional, immune response defective

(Information from FlyBase)

Pp1alpha-96A (UniProt:P48461) annotation

Cofactor: Mn(2+)Note=Binds 2 manganese ions per subunit. ;
Subunit: Interacts with Nop17l. (PubMed:17007873).

(Information from UniProt)

Growth factor receptor-bound protein 2

Best gene implication in ageing for this target family came from gene P62994 annotated in UniProt release 2014_02. Annotation GO 7568 (aging) was Inferred from Physical Interaction

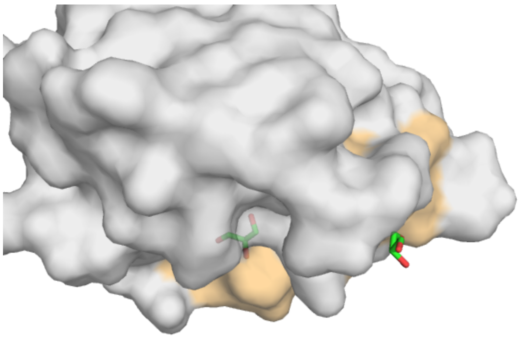

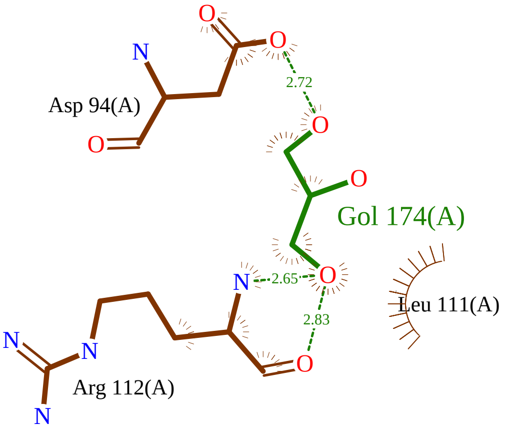

protein	amino acids contacts (binding site)
PDB:2h46:chainE:P62993	R K S Q R D R S E S D F K F G L R Y F W K F N E H T S S R - - - - -
PDB:2vvk:chainA:P62993	- G V V N P
PDB:3n84:chainA:P62993	R K S Q R D R S E S D F K F G L R Y F W K F N E H T S S R T V - - -
PDB:3n84:chainD:P62993	R K S Q R D R S E S D F K F G L R Y F W K F N E H T S S R - - - - -
PDB:3n8m:chainA:P62993	R K S Q R D R S E S D F K F G L R Y F W K F N E H T S S R - - - - -
PDB:3ov1:chainA:P62993	R K S Q R D R S E S D F K F G L R Y F W K F N E H T S S R - - - - -
PDB:3s8l:chainA:P62993	R K S Q R D R S E S D F K F G L R Y F W K F N E H T S S R - - - - -
PDB:3s8n:chainA:P62993	R K S Q R D R S E S D F K F G L R Y F W K F N E H T S S R - - - - -
PDB:4p9z:chainA:P62993	R K S Q R D R S E S D F K F G L R Y F W K F N E H T S S R T V - - -
sp:P62993:GRB2_HUMAN	R K S Q R D R S E S D F K F G L R Y F W K F N E H T S S R T V V N P
tr:B0LPF3:B0LPF3_HUMAN	R K S Q R D R S E S D F K F G L R Y F W K F N E H T S S R T V V N P
sp:P62994:GRB2_RAT	R K S Q R D R S E S D F K F G L R Y F W K F N E H T S S R T V V N P
sp:Q60631:GRB2_MOUSE	R K S Q R D R S E S D F K F G L R Y F W K F N E H T S S R T V V N P
tr:Q3U5I5:Q3U5I5_MOUSE	R K S Q R D R S E S D F K F G L R Y F W K F N E H T S S R T V V N P
sp:Q08012:DRK_DROME	R D S K - E R S E S D F K C P L R F F W K F N E H A S S R M V V R P
sp:P29355:SEM5_CAEEL	R D K P T D R C E S E F R F Q L R Y Y W K F N E H A S S R K V L K P

	whole protein		domain-based		contact-based	
protein	ident	simil	ident	simil	ident	simil
PDB:2h46:chainE:P62993	1.0	1.0	1.0	1.0	0.85	0.71
PDB:2vvk:chainA:P62993	0.96	0.98	0.96	0.98	0.15	0.0
PDB:3n84:chainA:P62993	1.0	1.0	1.0	1.0	0.88	0.81
PDB:3n84:chainD:P62993	1.0	1.0	1.0	1.0	0.85	0.71
PDB:3n8m:chainA:P62993	1.0	1.0	1.0	1.0	0.85	0.71
PDB:3ov1:chainA:P62993	1.0	1.0	1.0	1.0	0.85	0.71
PDB:3s8l:chainA:P62993	1.0	1.0	1.0	1.0	0.85	0.71
PDB:3s8n:chainA:P62993	1.0	1.0	1.0	1.0	0.85	0.71
PDB:4p9z:chainA:P62993	1.0	1.0	1.0	1.0	0.88	0.81
sp:P62993:GRB2_HUMAN	1.0	1.0	1.0	1.0	0.97	0.98
tr:B0LPF3:B0LPF3_HUMAN	1.0	1.0	1.0	1.0	0.97	0.98
sp:P62994:GRB2_RAT	1.0	1.0	1.0	1.0	0.97	0.98
sp:Q60631:GRB2_MOUSE	1.0	1.0	0.99	1.0	0.97	0.98
tr:Q3U5I5:Q3U5I5_MOUSE	1.0	1.0	0.99	1.0	0.97	0.98
sp:Q08012:DRK_DROME	0.64	0.86	0.61	0.85	0.71	0.74
sp:P29355:SEM5_CAEEL	0.55	0.81	0.56	0.85	0.62	0.73

drk (FBgn0004638) associated phenotypes
dominant, endocytosis defective, fully, learning defective, lethal - all die before end of pupal stage, memory defective, neuroanatomy defective, rescuable maternal effect, some die during pupal stage

(Information from FlyBase)

drk (UniProt:Q08012) annotation
Function: Required for proper signaling by sevenless. May act to stimulate the ability of Sos to catalyze Ras1 activation by linking sevenless and Sos in a signaling complex. (PubMed:8462097, PubMed:8462098).
Subunit: Interacts with autophosphorylated sev via SH2 domain and Sos, Dos and Dab via SH3 domains. Binds to tyrosine phosphorylated Dab via the SH2 domain. (PubMed:12128212, PubMed:8462097, PubMed:8462098, PubMed:9671493).
Subcellular location: Membrane (PubMed:8462098); Peripheral membrane protein (PubMed:8462098).
Tissue specificity: Found mainly in the developing eye and in the antennal disk. Also observed in other imaginal disks tested and in the embryo.

(Information from UniProt)

sem-5 (WBGene00004774) associated phenotypes
cell migration variant, egg laying variant, hermaphrodite sex muscle development variant, maternal effect lethal emb, rod like larval lethal, sex muscle morphology variant, vulval cell induction variant, vulvaless

(Information from WormBase)

sem-5 (UniProt:P29355) annotation
Function: Acts both in vulval induction and sex myoblast migration. Presumably interacts with the kinase receptor let-23 and with a target that modifies the Ras-like protein let-60.

(Information from UniProt)

14-3-3 protein zeta/delta

Best gene implication in ageing for this target family came from gene N1NV25 via mapping the annotation from WormBase WBGene00003920 annotated in WormBase 2014-03-11. Annotation GO 8340 (determination of adult lifespan) was Inferred from Mutant Phenotype

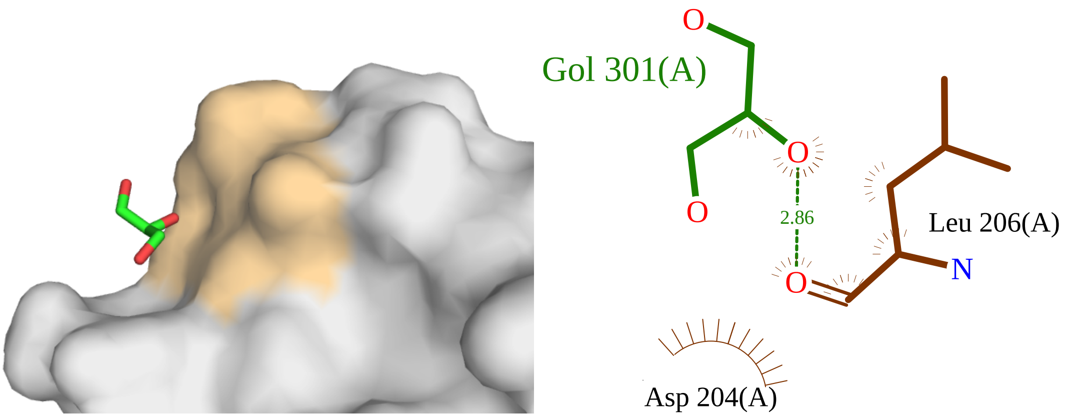

protein	amino acids contacts (binding site)
PDB:4hkc:chainA:P63104	L D L
tr:Q2F831:Q2F831_HUMAN	L D L
tr:B7Z2E6:B7Z2E6_HUMAN	L D L
tr:H0YB80:H0YB80_HUMAN	L D L
tr:B0AZS6:B0AZS6_HUMAN	L D L
tr:E7EX29:E7EX29_HUMAN	L D L
sp:P63104:1433Z_HUMAN	L D L
tr:D0PNI1:D0PNI1_HUMAN	L D L
sp:P63102:1433Z_RAT	L D L
sp:P63101:1433Z_MOUSE	L D L
sp:P29310:1433Z_DROME	L D L
tr:N1NV25:N1NV25_CAEEL	L D L
sp:P41932:14331_CAEEL	L D L
sp:Q20655:14332_CAEEL	L D L

	whole protein		domain-based		contact-based	
protein	ident	simil	ident	simil	ident	simil
PDB:4hkc:chainA:P63104	1.0	1.0	1.0	1.0	1.0	1.0
tr:Q2F831:Q2F831_HUMAN	0.43	0.43	0.39	0.39	1.0	1.0
tr:B7Z2E6:B7Z2E6_HUMAN	0.51	0.51	0.48	0.48	1.0	1.0
tr:H0YB80:H0YB80_HUMAN	0.53	0.53	0.5	0.5	1.0	1.0
tr:B0AZS6:B0AZS6_HUMAN	0.69	0.69	0.67	0.67	1.0	1.0
tr:E7EX29:E7EX29_HUMAN	0.94	0.94	0.97	0.97	1.0	1.0
sp:P63104:1433Z_HUMAN	1.0	1.0	1.0	1.0	1.0	1.0
tr:D0PNI1:D0PNI1_HUMAN	1.0	1.0	1.0	1.0	1.0	1.0
sp:P63102:1433Z_RAT	1.0	1.0	1.0	1.0	1.0	1.0
sp:P63101:1433Z_MOUSE	1.0	1.0	1.0	1.0	1.0	1.0
sp:P29310:1433Z_DROME	0.8	0.94	0.82	0.95	1.0	1.0
tr:N1NV25:N1NV25_CAEEL	0.37	0.46	0.38	0.45	1.0	1.0
sp:P41932:14331_CAEEL	0.76	0.91	0.8	0.94	1.0	1.0
sp:Q20655:14332_CAEEL	0.79	0.92	0.83	0.95	1.0	1.0

14-3-3zeta (FBgn0004907) associated phenotypes

learning defective, maternal effect, memory defective, mitotic cell cycle defective, neurophysiology defective

(Information from FlyBase)

14-3-3zeta (UniProt:P29310) annotation

Function: Required in Raf-dependent cell proliferation and photoreceptor differentiation during eye development. Acts upstream of Raf and downstream of Ras, and is essential for viability. Acts as a negative regulator by decreasing its voltage sensitivity. Inhibits yki activity by restricting its nuclear localization. (PubMed:10230800, PubMed:19900439).

Subunit: Homodimer; homodimerization is not essential for modulating the activity of Slo. Interacts with phosphorylated Slob; the interaction with Slob mediates an indirect interaction with Slo. Interacts with phosphorylated yki. (PubMed:10230800, PubMed:18256197, PubMed:19900439).

Subcellular location: Cytoplasm ECO:0000250.

Tissue specificity: Predominantly expressed in the ventral nerve cord of the embryo, and in the neural tissues of the head. Also found in the region posterior to the morphogenetic furrow of the eye imaginal disk where cells differentiate as photoreceptors.

Developmental stage: Expressed throughout all stages of embryonic and larval development.

(Information from UniProt)

par-5 (WBGene00003920) associated phenotypes

P granule localization defective, P0 spindle position defective early emb, antibody staining reduced, aster defective early emb, asymmetric cell division defective early emb, cell cleavage variant emb, embryonic polarity variant, gut granule biogenesis reduced, hermaphrodite fertility reduced, maternal effect lethal emb, maternal effect sterile, no Intestine, pharyngeal development variant, pronuclei meet centrally early emb, protein expression reduced, protein subcellular localization variant, pseu-

docleavage exaggerated early emb, relative cell cycle timing defective early emb, spindle orientation defective early emb, spindle orientation variant AB or P1 early emb, synchronous second division early emb

(Information from WormBase)

par-5 (UniProt:P41932) annotation
Subunit: Interacts with daf-16 and sir-2.1. (PubMed:16777605).
Subcellular location: Cytoplasm ECO:0000269—PubMed:16777605. Nucleus (PubMed:16777605).

(Information from UniProt)

ftt-2 (WBGene00001502) associated phenotypes
lethal, sterile

(Information from WormBase)

ftt-2 (UniProt:Q20655) annotation
Function: Required for extension of life-span by sir-2.1 (PubMed:16777605). Promotes nuclear export of yap-1 (PubMed:23396260). (PubMed:16777605, PubMed:23396260).
Subunit: Interacts with daf-16 and sir-2.1. (PubMed:16777605).
Subcellular location: Cytoplasm ECO:0000269—PubMed:16777605. Nucleus (PubMed:16777605).

(Information from UniProt)

Dual specificity mitogen-activated protein kinase kinase 1

Best gene implication in ageing for this target family came from gene Q02750 annotated in UniProt release 2014_02. Annotation GO subterm of 7568 (aging) was Inferred from Mutant Phenotype

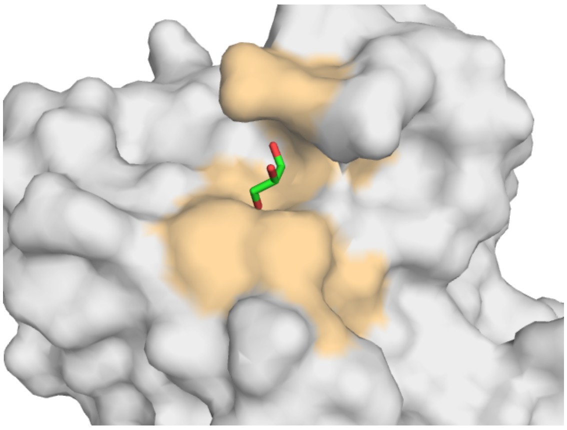

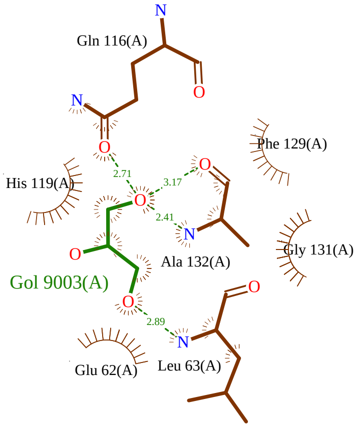

protein	amino acids contacts (binding site)
PDB:3dv3:chainA:Q02750	E L Q H F G A
sp:Q02750:MP2K1_HUMAN	E L Q H F G A
tr:A4QPA9:A4QPA9_HUMAN	E L Q H F G A
sp:Q01986:MP2K1_RAT	E L Q H F G A
tr:Q3TMJ8:Q3TMJ8_MOUSE	E L Q H F G A
sp:P31938:MP2K1_MOUSE	E L Q H F G A
sp:Q24324:DSOR1_DROME	E L K H F G A
sp:Q10664:MEK2_CAEEL	E L A H F G A
sp:P08018:PBS2_YEAST	- I E H F G A

protein	whole protein ident	whole protein simil	domain-based ident	domain-based simil	contact-based ident	contact-based simil
PDB:3dv3:chainA:Q02750	1.0	1.0	1.0	1.0	1.0	1.0
sp:Q02750:MP2K1_HUMAN	1.0	1.0	1.0	1.0	1.0	1.0
tr:A4QPA9:A4QPA9_HUMAN	1.0	1.0	1.0	1.0	1.0	1.0
sp:Q01986:MP2K1_RAT	0.99	1.0	1.0	1.0	1.0	1.0
tr:Q3TMJ8:Q3TMJ8_MOUSE	0.99	1.0	1.0	1.0	1.0	1.0
sp:P31938:MP2K1_MOUSE	0.99	1.0	1.0	1.0	1.0	1.0
sp:Q24324:DSOR1_DROME	0.59	0.82	0.75	0.93	0.86	0.92
sp:Q10664:MEK2_CAEEL	0.48	0.74	0.62	0.88	0.86	0.86
sp:P08018:PBS2_YEAST	0.2	0.38	0.48	0.79	0.57	0.61

Dsor1 (FBgn0010269) associated phenotypes

dominant, lethal - all die before end of pupal stage, neuroanatomy defective, polyphasic, some die during immature adult stage, some die during pupal stage

(Information from FlyBase)

Dsor1 (UniProt:Q24324) annotation

Function: Required downstream of Raf in the sevenless (sev), torso (tor), and Drosophila EGF receptor homolog (DER) signal transduction pathways. Involved in both positive regulation (at the posterior terminus) and negative regulation (at the anterior domain) of tll, as in other terminal class gene products, maybe via the ERK-A kinase. (PubMed:8381718).

Developmental stage: Expressed both maternally and zygotically. (PubMed:8381718).

Ptm: Phosphorylation on Ser/Thr by MAP kinase kinase kinases regulates positively the kinase activity.

(Information from UniProt)

mek-2 (WBGene00003186) associated phenotypes

AWA odorant chemotaxis defective, AWC odorant chemotaxis defective, bacterially unswollen, constipated, germ cell arrest, germ nuclei rachis, lethal, metabolic pathway variant, no oocytes, pachytene region organization variant, pathogen susceptibility increased, rod like larval lethal, sterile, vulval cell lineage variant, vulvaless

(Information from WormBase)

mek-2 (UniProt:Q10664) annotation

Function: Functions in the let-60 Ras signaling pathway; acts downstream of lin-45 raf kinase, but before the sur-1/mpK-1 gene product in controlling vulval cell differentiation.

Enzyme regulation: Activated by tyrosine and threoninephosphorylation catalyzed by MAP kinase kinase kinases.

(Information from UniProt)

Neurogenic locus notch homolog protein 2

Best gene implication in ageing for this target family came from gene P07207 annotated in UniProt release 2014_02. Annotation GO 8340 (determination of adult lifespan) was Inferred from Mutant Phenotype

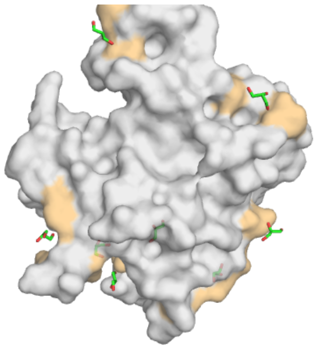

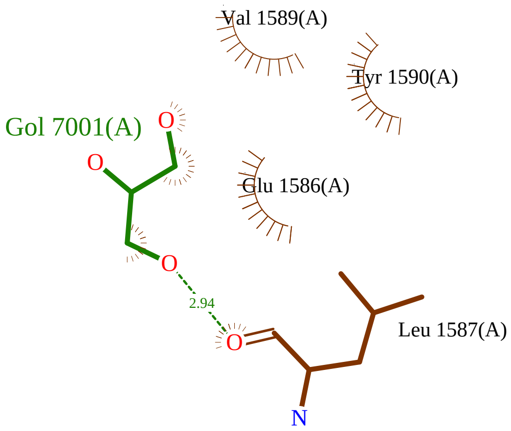

protein	amino acids contacts (binding site)
PDB:2oo4:chainA:Q04721	D A S P W F K N G G Q A L R G T N L I R E L V Y Q Q D D
sp:Q04721:NOTC2_HUMAN	D A S P W F K N G G Q A L R G T N L I R E L V Y Q Q D D
tr:G3V8G9:G3V8G9_RAT	D A S S W F K N G G Q S L R G T N L I Q A L V Y Q Q D D
tr:Q3U1W7:Q3U1W7_MOUSE	D A S T W F K N G G Q S L R G T N L I Q A L V Y Q Q D D
tr:G5E8J0:G5E8J0_MOUSE	D A S T W F K N G G Q S L R G T N L I Q A L V Y Q Q D D
tr:Q4QQB7:Q4QQB7_DROME	E Q T A W Y G N S G T Q L R S T T V L K H D I I Q L E R
sp:P07207:NOTCH_DROME	E Q T A W Y G N S G T Q L R S T T V L K H D I I Q L E R

	whole protein		domain-based		contact-based	
protein	ident	simil	ident	simil	ident	simil
PDB:2oo4:chainA:Q04721	1.0	1.0	1.0	1.0	1.0	1.0
sp:Q04721:NOTC2_HUMAN	1.0	1.0	1.0	1.0	1.0	1.0
tr:G3V8G9:G3V8G9_RAT	0.92	0.98	0.9	0.97	0.86	0.89
tr:Q3U1W7:Q3U1W7_MOUSE	0.43	0.45	0.89	0.96	0.86	0.9
tr:G5E8J0:G5E8J0_MOUSE	0.92	0.98	0.89	0.96	0.86	0.9
tr:Q4QQB7:Q4QQB7_DROME	0.15	0.31	0.29	0.69	0.25	0.64
sp:P07207:NOTCH_DROME	0.36	0.66	0.29	0.69	0.25	0.64

N (FBgn0004647) associated phenotypes

cell adhesion defective, cell non-autonomous, cell shape defective, chemosensitive behavior defective, cold sensitive, courtship behavior defective, decreased cell death, decreased cell number, dominant, flightless, germline clone, gravitaxis defective, heat sensitive, homeotic, hyperplasia, immune response defective, increased cell death, increased cell number, lethal - all die before end of P-stage, lethal - all die before end of pupal stage, lethal - all die during pharate adult stage, maternal effect, memory defective, mitotic cell cycle defective, neuroanatomy defective, neurogenic phenotype, partially, partially lethal - majority die, planar polarity defective, poor, reduced, short lived, size defective, somatic clone, some die during pharate adult stage, some die during pupal stage, sterile, temperature conditional

(Information from FlyBase)

N (UniProt:P07207) annotation

Function: Signaling protein, which regulates, with both positive and negative signals, the differentiation of at least central and peripheral nervous system and eye, wing disk, oogenesis, segmental appendages such as antennae and legs, and muscles, through lateral inhibition or induction. Functions as a receptor for membrane- bound ligands Delta and Serrate to regulate cell-fate determination. Upon ligand activation, and releasing from the cell membrane, the Notch intracellular domain (NICD) forms a transcriptional activator complex with Su(H) (Suppressor of hairless) and activates genes of the E(spl) complex. Essential for proper differentiation of ectoderm. Fringe (fng) acts in the Golgi to determine the type of O-linked fucose on the EGF modules in N, altering the ability of N to bind with Delta (Dl). O-fut1 also has a role in modulating the interaction. Rumi acts in the endoplasmic reticulum to glucosylate the EGF modules in N, this is required for correct folding and cleavage of N. (PubMed:10935637, PubMed:18243100).

Subunit: Homomer. Interacts with Su(H) when activated. Interacts with Dx via its ANK repeats. Interacts with Dl via the EGF repeats and the Dl EGF repeats. Interacts with Nedd4 and Su(dx). Interacts with O-fut1; the interaction glycosylates N and transports N to early endosomes. (PubMed:10935637, PubMed:14573873, PubMed:15620649, PubMed:15620650, PubMed:17329366, PubMed:7671825, PubMed:8162848).
Subcellular location: Cell membrane ECO:0000269—PubMed:17329366; Single-pass type I membrane protein (PubMed:17329366). Endosome (PubMed:17329366). Note=Transported to early endosomes by O-fut1.
Subcellular location: Processed neurogenic locus Notch protein: Nucleus. Note=Upon activation and S3 cleavage, it is released from the cell membrane and enters into the nucleus in conjunction with Su(H).
Domain: Crystal structure of the ANK repeat domain shows that there are 7 repeats and the stabilizing C-terminal repeat enhances the protein stability by extending the ankyrin domain. (PubMed:14573873).
Ptm: Upon binding its ligands such as Delta or Serrate, it is cleaved (S2 cleavage) in its extracellular domain, close to the transmembrane domain. S2 cleavage is probably mediated by Kuz. It is then cleaved (S3 cleavage) downstream of its transmembrane domain, releasing it from the cell membrane. S3 cleavage requires Psn.
Ptm: O-glycosylated. O-fucosylated by O-fut1 in the EGF repeat doamin which inhibits both Serrate/Ser- and Delta/Dl-binding. (PubMed:12909620).
Ptm: Ubiquitinated by Nedd4; which promotes ligand-independent endocytosis and proteasomal degradation. May also be ubiquitinated by Su(dx). (PubMed:15620649).

(Information from UniProt)

Histone acetyltransferase ESA1

Best gene implication in ageing for this target family came from gene Q92794 annotated in UniProt release 2014_02. Annotation GO subterm of 7568 (aging) was Inferred from Mutant Phenotype

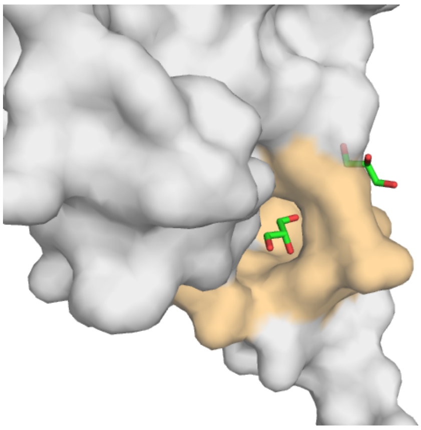

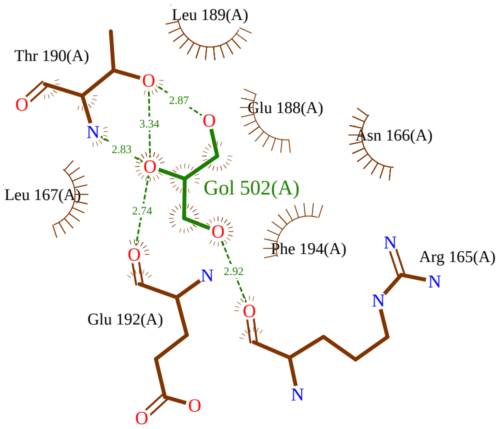

protein	amino acids contacts (binding site)
PDB:3to7:chainA:Q08649	R N L F Y I E L T E F
tr:C9K0F9:C9K0F9_HUMAN	R C P S Y Q E Y S L K
tr:A5PLL3:A5PLL3_HUMAN	R C P S Y Q E Y S L K
tr:A5PKX7:A5PKX7_HUMAN	R C P S Y Q E Y S L K
sp:Q92794:KAT6A_HUMAN	R C P S Y Q E Y S L K
tr:Q3V1G6:Q3V1G6_MOUSE	R C P S Y Q E Y S L K
tr:Q3UPM9:Q3UPM9_MOUSE	R C P S Y Q E Y S L K
tr:G3X940:G3X940_MOUSE	R C P S Y Q E Y S L K
tr:D0IQI5:D0IQI5_DROME	Q S P S F Q E Y A L K
tr:Q9W1A9:Q9W1A9_DROME	Q S P S F Q E Y A L K
tr:L8E6K3:L8E6K3_CAEEL	R L P G F A E F I V K
tr:Q21789:Q21789_CAEEL	R L P G F A E F I V K
tr:D7SFM3:D7SFM3_CAEEL	R L P G F A E F I V K
tr:K8ESF3:K8ESF3_CAEEL	R L P G F A E F I V K
tr:G5EGU3:G5EGU3_CAEEL	R L P G F A E F I V K
tr:K8ES81:K8ES81_CAEEL	R L P G F A E F I V K
tr:G5EGP0:G5EGP0_CAEEL	R L P G F A E F I V K
tr:K8F7U3:K8F7U3_CAEEL	R L P G F A E F I V K
tr:G5EBH5:G5EBH5_CAEEL	R L P G F A E F I V K
tr:K8ERR8:K8ERR8_CAEEL	R L P G F A E F I V K
sp:Q08649:ESA1_YEAST	R N L F Y I E L T E F

	whole protein		domain-based		contact-based	
protein	ident	simil	ident	simil	ident	simil
PDB:3to7:chainA:Q08649	1.0	1.0	1.0	1.0	1.0	1.0
tr:C9K0F9:C9K0F9_HUMAN	0.16	0.29	0.41	0.79	0.27	0.4
tr:A5PLL3:A5PLL3_HUMAN	0.18	0.39	0.41	0.79	0.27	0.4
tr:A5PKX7:A5PKX7_HUMAN	0.13	0.28	0.41	0.79	0.27	0.4
sp:Q92794:KAT6A_HUMAN	0.08	0.16	0.41	0.79	0.27	0.4
tr:Q3V1G6:Q3V1G6_MOUSE	0.13	0.28	0.41	0.79	0.27	0.4
tr:Q3UPM9:Q3UPM9_MOUSE	0.11	0.25	0.41	0.79	0.27	0.4
tr:G3X940:G3X940_MOUSE	0.07	0.16	0.41	0.79	0.27	0.4
tr:D0IQI5:D0IQI5_DROME	0.13	0.3	0.32	0.76	0.09	0.44
tr:Q9W1A9:Q9W1A9_DROME	0.06	0.14	0.32	0.76	0.09	0.44
tr:L8E6K3:L8E6K3_CAEEL	0.24	0.52	0.3	0.75	0.18	0.42
tr:Q21789:Q21789_CAEEL	0.23	0.5	0.3	0.75	0.18	0.42
tr:D7SFM3:D7SFM3_CAEEL	0.11	0.24	0.3	0.75	0.18	0.42
tr:K8ESF3:K8ESF3_CAEEL	0.11	0.23	0.3	0.75	0.18	0.42
tr:G5EGU3:G5EGU3_CAEEL	0.11	0.24	0.3	0.75	0.18	0.42
tr:K8ES81:K8ES81_CAEEL	0.11	0.23	0.3	0.75	0.18	0.42
tr:G5EGP0:G5EGP0_CAEEL	0.09	0.21	0.3	0.75	0.18	0.42
tr:K8F7U3:K8F7U3_CAEEL	0.09	0.21	0.3	0.75	0.18	0.42
tr:G5EBH5:G5EBH5_CAEEL	0.09	0.2	0.3	0.75	0.18	0.42
tr:K8ERR8:K8ERR8_CAEEL	0.09	0.2	0.3	0.75	0.18	0.42
sp:Q08649:ESA1_YEAST	1.0	1.0	1.0	1.0	1.0	1.0

enok-RA (UniProt:D0IQI5) annotation
Subcellular location: Nucleus ECO:0000256—RuleBase:RU361211.

(Information from UniProt)

enok (FBgn0034975) associated phenotypes
female semi-sterile, germline clone, neuroanatomy defective, size defective, somatic clone

(Information from FlyBase)

enok (UniProt:Q9W1A9) annotation
Subcellular location: Nucleus ECO:0000256—RuleBase:RU361211.

(Information from UniProt)

lsy-12 (WBGene00045419) associated phenotypes
egg laying defective, locomotion variant, loss of asymmetry ASE, protruding vulva, reduced brood size, sick

(Information from WormBase)

lsy-12 (UniProt:L8E6K3) annotation
Subcellular location: Nucleus ECO:0000256—RuleBase:RU361211.

(Information from UniProt)

lsy-12 (UniProt:Q21789) annotation
Subcellular location: Nucleus ECO:0000256—RuleBase:RU361211.

(Information from UniProt)

lsy-12 (UniProt:D7SFM3) annotation
Subcellular location: Nucleus ECO:0000256—RuleBase:RU361211.

(Information from UniProt)

lsy-12 (UniProt:K8ESF3) annotation
Subcellular location: Nucleus ECO:0000256—RuleBase:RU361211.

(Information from UniProt)

lsy-12 (UniProt:G5EGU3) annotation
Subcellular location: Nucleus ECO:0000256—RuleBase:RU361211.

(Information from UniProt)

lsy-12 (UniProt:K8ES81) annotation
Subcellular location: Nucleus ECO:0000256—RuleBase:RU361211.

(Information from UniProt)

lsy-12 (UniProt:G5EGP0) annotation
Subcellular location: Nucleus ECO:0000256—RuleBase:RU361211.

(Information from UniProt)

lsy-12 (UniProt:K8F7U3) annotation
Subcellular location: Nucleus ECO:0000256—RuleBase:RU361211.

(Information from UniProt)

lsy-12 (UniProt:G5EBH5) annotation
Subcellular location: Nucleus ECO:0000256—RuleBase:RU361211.

(Information from UniProt)

lsy-12 (UniProt:K8ERR8) annotation
Subcellular location: Nucleus ECO:0000256—RuleBase:RU361211.

(Information from UniProt)

Tyrosine-protein kinase ITK/TSK

Best gene implication in ageing for this target family came from gene P08630 annotated in UniProt release 2014_02. Annotation GO 8340 (determination of adult lifespan) was Inferred from Mutant Phenotype

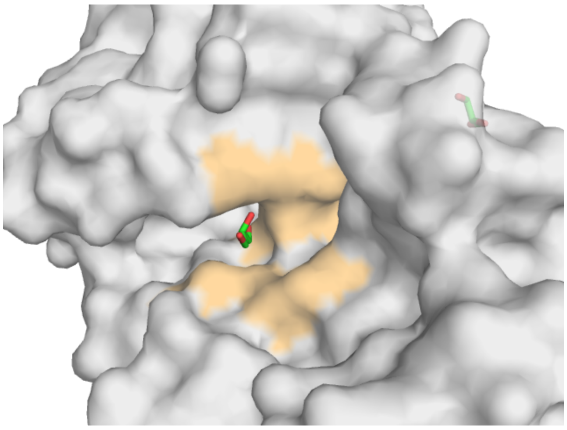

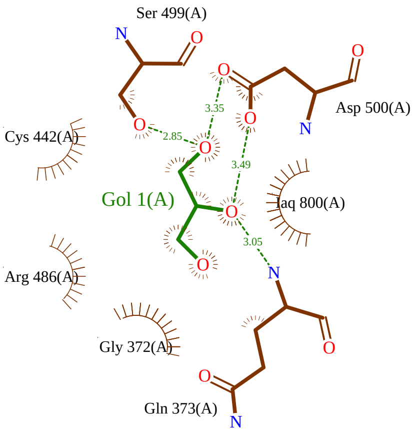

protein	amino acids contacts (binding site)
PDB:3t9t:chainA:Q08881	G Q K C R S D T W F L Y
PDB:4kio:chainC:Q08881	G Q K C R S D - W F L Y
sp:Q08881:ITK_HUMAN	G Q K C R S D T W F L Y
tr:D4A7W7:D4A7W7_RAT	G Q K C R S D T W F L Y
tr:Q8CAW3:Q8CAW3_MOUSE	G Q K C R S D T W F L Y
tr:A1A560:A1A560_MOUSE	G Q K C R S D T W F L Y
tr:Q5STT8:Q5STT8_MOUSE	G Q K C R S D T W F L Y
sp:Q03526:ITK_MOUSE	G Q K C R S D T W F L Y
sp:P08630:BTKL_DROME	G Q K S R A D T W I L E

	whole protein		domain-based		contact-based	
protein	ident	simil	ident	simil	ident	simil
PDB:3t9t:chainA:Q08881	0.99	1.0	0.99	1.0	1.0	1.0
PDB:4kio:chainC:Q08881	0.99	0.99	1.0	1.0	0.92	0.83
sp:Q08881:ITK_HUMAN	1.0	1.0	1.0	1.0	1.0	1.0
tr:D4A7W7:D4A7W7_RAT	0.94	0.97	0.97	0.99	1.0	1.0
tr:Q8CAW3:Q8CAW3_MOUSE	0.53	0.55	0.97	0.99	1.0	1.0
tr:A1A560:A1A560_MOUSE	0.94	0.98	0.97	0.99	1.0	1.0
tr:Q5STT8:Q5STT8_MOUSE	0.94	0.98	0.97	0.99	1.0	1.0
sp:Q03526:ITK_MOUSE	0.93	0.97	0.97	0.99	1.0	1.0
sp:P08630:BTKL_DROME	0.35	0.62	0.55	0.85	0.67	0.73

Btk29A (FBgn0003502) associated phenotypes

cell shape defective, lethal - all die before end of first instar larval stage, mating defective, partially, short lived, size defective, some die during first instar larval stage

(Information from FlyBase)

Btk29A (UniProt:P08630) annotation

Function: Required for proper ring canal development. Also required for the development of male genitalia and for adult survival. (PubMed:10330180, PubMed:9655810).
Cofactor: Zn(2+)Note=Binds 1 zinc ion per subunit. ;
Subcellular location: Note=Ring canals.
Tissue specificity: Ring canals in the egg chambers and imaginal disks of third-instar larvae. (PubMed:10330180, PubMed:3110602, PubMed:9655810).
Developmental stage: Expressed both maternally and zygotically. Predominantly in early to middle embryogenesis, in larvae and adult females. (PubMed:10330180, PubMed:3110602).
Disruption phenotype: Flies exhibit shortened copulatory duration (due to incomplete fusion of the left and right halves of the apodeme that holds the penis during copulation) and reduced adult-stage life span. (PubMed:10330180).

(Information from UniProt)

Ubiquitin-like protein SMT3

Best gene implication in ageing for this target family came from gene P63165 via mapping the annotation from Ensembl ENSG00000116030 via mapping the annotation from EntrezGene 7341 via mapping the annotation from GenAgeHuman 0211 annotated in GenAge release 17.

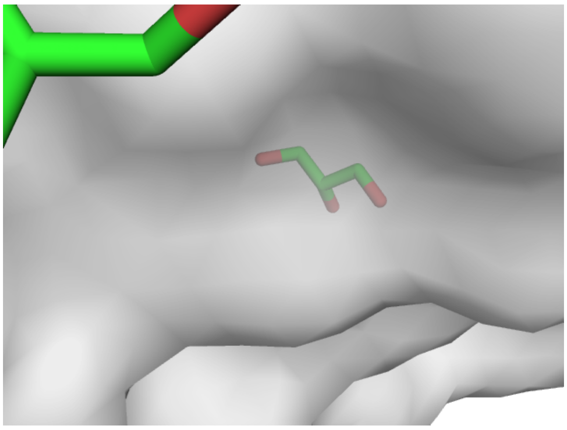

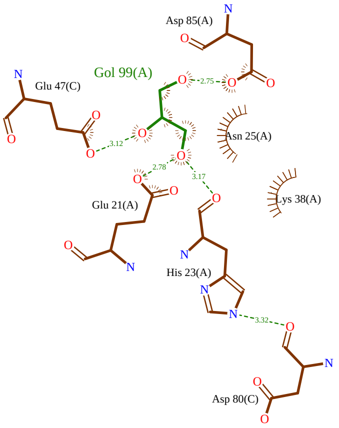

protein	amino acids contacts (binding site)
PDB:3qht:chainA:Q12306	E H N S S E F K Y D I D D D I
PDB:3qht:chainB:Q12306	- H N S S E F K Y D I D D D I
sp:P63165:SUMO1_HUMAN	G Y K S S E H K F E Q N E D V
tr:B8ZZN6:B8ZZN6_HUMAN	G Y K S S E H K F E Q N E D V
sp:Q5I0H3:SUMO1_RAT	G Y K S S E H K F E Q N E D V
sp:P63166:SUMO1_MOUSE	G Y K S S E H K F E Q N E D V
sp:P55853:SUMO_CAEEL	A Y K S N E H R F D R D D D V
sp:Q12306:SMT3_YEAST	E H N S S E F K Y D I D D D I

	whole protein		domain-based		contact-based	
protein	ident	simil	ident	simil	ident	simil
PDB:3qht:chainA:Q12306	1.0	1.0	1.0	1.0	1.0	1.0
PDB:3qht:chainB:Q12306	1.0	1.0	1.0	1.0	0.93	0.86
sp:P63165:SUMO1_HUMAN	0.45	0.79	0.45	0.8	0.33	0.62
tr:B8ZZN6:B8ZZN6_HUMAN	0.31	0.55	0.3	0.54	0.33	0.62
sp:Q5I0H3:SUMO1_RAT	0.45	0.79	0.45	0.8	0.33	0.62
sp:P63166:SUMO1_MOUSE	0.45	0.79	0.45	0.8	0.33	0.62
sp:P55853:SUMO_CAEEL	0.37	0.73	0.38	0.74	0.4	0.63
sp:Q12306:SMT3_YEAST	1.0	1.0	1.0	1.0	1.0	1.0

smo-1 (WBGene00004888) associated phenotypes

cell junction variant, desynapsis defective, germ cell development variant, protein aggregation variant, protein expression ectopic, protein subcellular localization variant, protein transport variant, somatic gonad morphology variant, sterile, synapsis defective, vulva morphology variant

(Information from WormBase)

smo-1 (UniProt:P55853) annotation

Function: Ubiquitin-like protein which can be covalently attached to target lysines as a monomer. Does not seem to be involved in protein degradation and may function as an antagonist of ubiquitin in the degradation process. Plays a role in a number of cellular processes such as nuclear transport, DNA replication and repair, mitosis and signal transduction. Covalent attachment to its substrates requires prior activation by the E1 complex aos-1-uba-2 and linkage to the E2 enzyme ubc-9, and can be promoted by an E3 ligase such as gei-17. Required for embryonic development, fertility, vulval morphogenesis and inhibition of vulval cell fates. (PubMed:11806825, PubMed:15107848, PubMed:15466489, PubMed:15689373, PubMed:15990876, PubMed:16701625).
Subunit: Covalently attached to a number of proteins such as tbx- 2, lin-1, lin-11 and sop-2.
Subcellular location: Cytoplasm ECO:0000269—PubMed:25475837. Nucleus (PubMed:25475837).

Cytoplasm, cytoskeleton, spindle (PubMed:25475837). Chromosome (PubMed:25475837). Cytoplasm, cytoskeleton, microtubule organizing center, centrosome (PubMed:25475837). Note=At the first embryonic mitotic division, enriched in the nucleus and released to the cytoplasm when the nuclear envelope breaks down at the start of mitosis. During mitosis, localizes to the metaphase plate and to the centrosomal region. Also localizes to segregating chromosomes at the beginning of anaphase. At late anaphase and telophase, observed in the spindle midzone and in the two daughter nuclei. (PubMed:25475837).
Ptm: Cleavage of precursor form by ulp-1 is necessary for function.
Disruption phenotype: RNAi-mediated knockdown causes chromosome misalignment and anaphase bridges during the first embryonic mitotic division. (PubMed:25475837).

(Information from UniProt)

Filamin-C

Best gene implication in ageing for this target family came from gene Q9VEN1 annotated in UniProt release 2014_02. Annotation GO 8340 (determination of adult lifespan) was Inferred from Mutant Phenotype

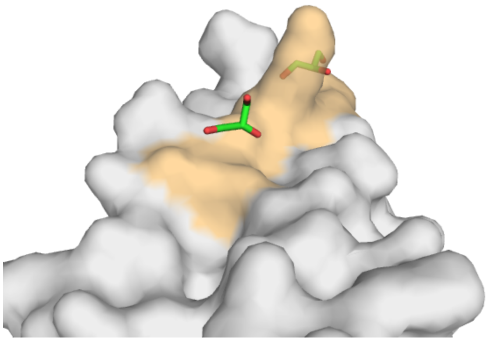

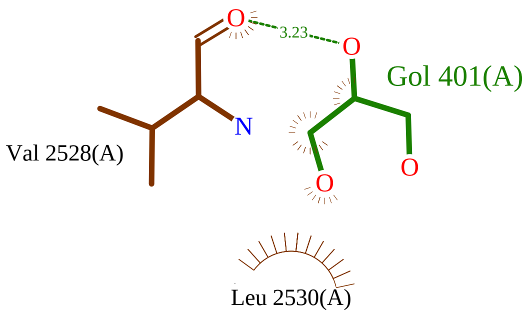

protein	amino acids contacts (binding site)
PDB:2nqc:chainA:Q14315	P S K V L R
tr:Q59H94:Q59H94_HUMAN	P S K V L R
sp:Q14315:FLNC_HUMAN	P S K V L R
sp:D3ZHA0:FLNC_RAT	P S K V L R
tr:Q3UXJ8:Q3UXJ8_MOUSE	P S K V L R
tr:D3YW87:D3YW87_MOUSE	P S K V L R
sp:Q8VHX6:FLNC_MOUSE	P S K V L R
tr:D3Z576:D3Z576_MOUSE	P S K V L R
tr:Q6NN54:Q6NN54_DROME	P S K V M K
tr:C7LAD0:C7LAD0_DROME	P S K V M K
tr:A4V310:A4V310_DROME	P S K V M K
tr:C7LAD1:C7LAD1_DROME	P S K V M K
tr:B7Z0L2:B7Z0L2_DROME	P S K V M K
tr:Q8T3K7:Q8T3K7_DROME	P S K V M K
tr:Q29R34:Q29R34_DROME	P S K V M K
sp:Q9VEN1:FLNA_DROME	P S K V M K
tr:Q9GQV2:Q9GQV2_DROME	P S K V M K
tr:Q7KSF4:Q7KSF4_DROME	P S K V M K
tr:D0IMZ6:D0IMZ6_CAEEL	P S K A L E
tr:Q3V5H2:Q3V5H2_CAEEL	P S K A L E
tr:D0IMZ8:D0IMZ8_CAEEL	P S K A L E
tr:D0IMZ7:D0IMZ7_CAEEL	P S K A L E
tr:D0IMZ5:D0IMZ5_CAEEL	P S K A L E

	whole protein		domain-based		contact-based	
protein	ident	simil	ident	simil	ident	simil
PDB:2nqc:chainA:Q14315	1.0	1.0	1.0	1.0	1.0	1.0
tr:Q59H94:Q59H94_HUMAN	0.49	0.49	1.0	1.0	1.0	1.0
sp:Q14315:FLNC_HUMAN	1.0	1.0	1.0	1.0	1.0	1.0
sp:D3ZHA0:FLNC_RAT	0.98	0.99	0.99	0.99	1.0	1.0
tr:Q3UXJ8:Q3UXJ8_MOUSE	0.28	0.28	0.99	0.99	1.0	1.0
tr:D3YW87:D3YW87_MOUSE	0.97	0.98	0.99	0.99	1.0	1.0
sp:Q8VHX6:FLNC_MOUSE	0.98	0.99	0.99	0.99	1.0	1.0
tr:D3Z576:D3Z576_MOUSE	0.98	0.99	0.99	0.99	1.0	1.0
tr:Q6NN54:Q6NN54_DROME	0.17	0.26	0.51	0.81	0.67	0.94
tr:C7LAD0:C7LAD0_DROME	0.17	0.27	0.51	0.81	0.67	0.94
tr:A4V310:A4V310_DROME	0.17	0.27	0.51	0.81	0.67	0.94
tr:C7LAD1:C7LAD1_DROME	0.17	0.28	0.51	0.81	0.67	0.94
tr:B7Z0L2:B7Z0L2_DROME	0.17	0.28	0.51	0.81	0.67	0.94
tr:Q8T3K7:Q8T3K7_DROME	0.24	0.42	0.51	0.81	0.67	0.94
tr:Q29R34:Q29R34_DROME	0.24	0.42	0.51	0.81	0.67	0.94
sp:Q9VEN1:FLNA_DROME	0.4	0.67	0.51	0.81	0.67	0.94
tr:Q9GQV2:Q9GQV2_DROME	0.4	0.67	0.51	0.81	0.67	0.94
tr:Q7KSF4:Q7KSF4_DROME	0.43	0.72	0.51	0.81	0.67	0.94
tr:D0IMZ6:D0IMZ6_CAEEL	0.13	0.25	0.46	0.79	0.67	0.82
tr:Q3V5H2:Q3V5H2_CAEEL	0.13	0.26	0.46	0.79	0.67	0.82
tr:D0IMZ8:D0IMZ8_CAEEL	0.26	0.59	0.46	0.79	0.67	0.82
tr:D0IMZ7:D0IMZ7_CAEEL	0.26	0.59	0.46	0.79	0.67	0.82
tr:D0IMZ5:D0IMZ5_CAEEL	0.26	0.6	0.46	0.79	0.67	0.82

cher (FBgn0014141) associated phenotypes

chemical sensitive, heat sensitive, learning defective, locomotor behavior defective, long lived, male fertile, memory defective, neuroanatomy defective, short lived, uncoordinated

(Information from FlyBase)

cher (UniProt:Q9VEN1) annotation

Function: Involved in the germline ring canal formation. May tether actin microfilament within the ovarian ring canal to the cell membrane. Contributes to actin microfilaments organization. (PubMed:10477759, PubMed:10556087).

Subunit: Interacts with Ten-m. (PubMed:21857973).

Subcellular location: Cytoplasm, cytoskeleton. Cell membrane. Note=Localizes to intercellular bridges corresponding to ring canals that connect cytoplasm of nurse cells to the developing oocyte. Localizes to nurse cell plasma membranes. Detected in both the inner and outer rims of the ring canal.
Tissue specificity: Germline-specific in females (at protein level). Expressed in ovary. (PubMed:10477759, PubMed:10556087).
Developmental stage: Expressed throughout the egg-chamber development (at protein level). Expressed in the embryo. (PubMed:10477759, PubMed:10556087).
Disruption phenotype: Shows defect in ring canal assembly and female sterility. (PubMed:10477759).

(Information from UniProt)

fln-1 (WBGene00022048) associated phenotypes
reduced brood size

(Information from WormBase)

ELAV-like protein 1

Best gene implication in ageing for this target family came from gene Q24474 via mapping the annotation from Ensembl FBgn0010263 via mapping the annotation from EntrezGene 33498 via mapping the annotation from GenAgeModels 0877 annotated in GenAge release 17.

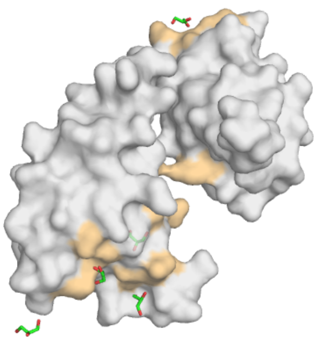

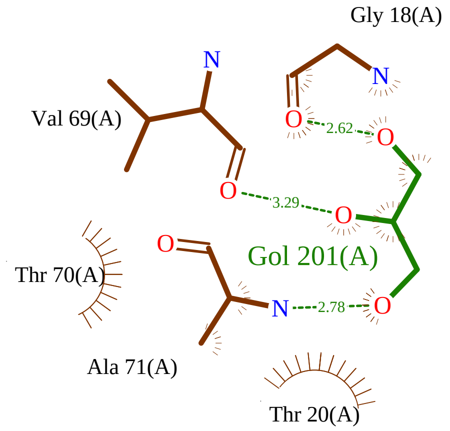

protein	amino acids contacts (binding site)
PDB:4ed5:chainA:Q15717	G R T Y E S A V T A R P S S G T Q N S R V V S R F E T
PDB:4egl:chainA:Q15717	G R T Y E S A V T A R P S S G T Q N S R V V S R F E T
sp:Q15717:ELAV1_HUMAN	G R T Y E S A V T A R P S S G T Q N S R V V S R F E T
tr:B4DVB8:B4DVB8_HUMAN	G R T Y E S A V T A R P S S G T Q N S R V V S R F E T
tr:B5DF91:B5DF91_RAT	G R T Y E S A V T A R P S S G T Q N S R V V S R F E T
sp:P70372:ELAV1_MOUSE	G R T Y E S A V T A R P S S G T Q N S R V V S R F E T
tr:Q24474:Q24474_DROME	P K T Y E S C V K Q K P S S G T Q T S R I C S K F E T
tr:Q26293:Q26293_DROME	P K T Y E S C V K Q K P S S G T Q T S R I C S K F E T
tr:Q9VQJ0:Q9VQJ0_DROME	P K T Y E S C V K Q K P S S G T Q T S R I C S K F E T
sp:P16914:ELAV_DROME	T R T Y E S V V R P Q P S S G T Q T S R I Q T K F D V
tr:A2RVE5:A2RVE5_DROME	P K T Y E S C V K Q K P S S G T Q T S R I C S K F E T
tr:Q20084:Q20084_CAEEL	S K T Y E S C V R E R P S S G T L T S R I S S K F E T

protein	whole protein ident	simil	domain-based ident	simil	contact-based ident	simil
PDB:4ed5:chainA:Q15717	1.0	1.0	1.0	1.0	1.0	1.0
PDB:4egl:chainA:Q15717	0.99	1.0	0.99	1.0	1.0	1.0
sp:Q15717:ELAV1_HUMAN	1.0	1.0	1.0	1.0	1.0	1.0
tr:B4DVB8:B4DVB8_HUMAN	0.92	0.92	1.0	1.0	1.0	1.0
tr:B5DF91:B5DF91_RAT	0.98	1.0	0.99	1.0	1.0	1.0
sp:P70372:ELAV1_MOUSE	0.98	1.0	0.99	1.0	1.0	1.0
tr:Q24474:Q24474_DROME	0.44	0.63	0.71	0.91	0.63	0.72
tr:Q26293:Q26293_DROME	0.44	0.62	0.69	0.89	0.63	0.72
tr:Q9VQJ0:Q9VQJ0_DROME	0.44	0.62	0.69	0.89	0.63	0.72
sp:P16914:ELAV_DROME	0.36	0.57	0.57	0.81	0.56	0.77
tr:A2RVE5:A2RVE5_DROME	0.3	0.43	0.69	0.89	0.63	0.72
tr:Q20084:Q20084_CAEEL	0.35	0.59	0.64	0.89	0.63	0.71

Rbp9 (FBgn0010263) associated phenotypes
conditional, locomotor behavior defective, partially, partially lethal - majority die, short lived

(Information from FlyBase)

elav (FBgn0260400) associated phenotypes
flight defective, flightless, heat sensitive, locomotor behavior defective, neuroanatomy defective, neurophysiology defective, partially lethal - majority die, progressive, sterile, uncoordinated, visual behavior defective

(Information from FlyBase)

elav (UniProt:P16914) annotation
Function: Required for the proper development and maintenance of neurons presumably by affecting RNA metabolism. (PubMed:3144044).

(Information from UniProt)

exc-7 (WBGene00001368) associated phenotypes
aldicarb resistant, excretory canal morphology variant, pale

(Information from WormBase)

Mitogen-activated protein kinase 14

Best gene implication in ageing for this target family came from gene O61443 annotated in UniProt release 2014_02. Annotation GO 8340 (determination of adult lifespan) was Inferred from Mutant Phenotype

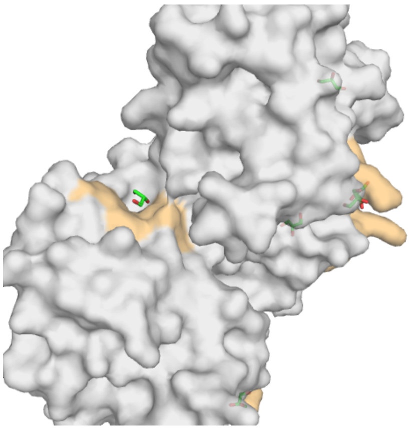

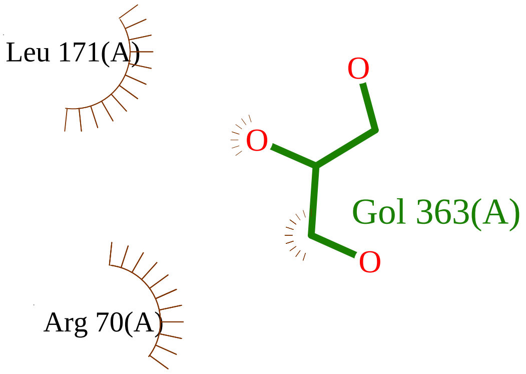

protein	amino acids contacts (binding site)
PDB:1ove:chainA:Q16539	E Q N S F Y R R K M K H V I G H M V Q K L D H F A N D E K F G L D Y M L N E L
PDB:1wbs:chainA:Q16539	E Q N S F Y R R K M K H V I G H M V Q K L D H F A N D E K F G L D Y M L N E L
PDB:1wbv:chainA:Q16539	E Q N S F Y R R K M K H V I G H M V Q K L D H F A N D E K F G L D Y M L N E L
PDB:2i0h:chainA:Q16539	E Q N S F Y R R K M K H V I G H M V Q K L D H F A N D E K F G L D Y M L N E L
PDB:2zaz:chainA:Q16539	E Q N - F Y R R K M K H V I G H M V Q K L D H F A N D E K F G L D Y M L N E L
PDB:2zb0:chainA:Q16539	E Q N S F Y R R K M K H V I G H M V Q K L D H F A N D E K F G L D Y M L N E L
PDB:2zb1:chainA:Q16539	E Q N - F Y R R K M K H V I G H M V Q K L D H F A N D E K F G L D Y M L N E L
PDB:3d7z:chainA:Q16539	E Q N S F Y R R K M K H V I G H M V Q K L D H F A N D E K F G L D Y M L N E L
PDB:3d83:chainA:Q16539	E Q N S F Y R R K M K H V I G H M V Q K L D H F A N D E K F G L D Y M L N E L
PDB:3e92:chainA:Q16539	E Q N S F Y R R K M K H V I G H M V Q K L D H F A N D E K F G L D Y M L N E L
PDB:3e93:chainA:Q16539	E Q N S F Y R R K M K H V I G H M V Q K L D H F A N D E K F G L D Y M L N E L
PDB:3hv3:chainA:Q16539	E Q N S F Y R R K M K H V I G H M V Q K L D H F A N D E K F G - - - M L N E L
PDB:3iph:chainA:Q16539	E Q N S F Y R R K M K H V I G H M V Q K L D H F A N D E K F G L D Y M L N E L
PDB:3mpt:chainA:Q16539	E Q N S F Y R R K M K H V I G H M V Q K L D H F A N D E K F G L D Y M L N E L
PDB:4ewq:chainA:Q16539	E Q N S F Y R R K M K H V I G H M V Q K L D H F A N D E K F - - D Y M L N E L
PDB:4f9w:chainA:Q16539	E Q N S F Y R R K M K H V I G H M V Q K L D H F A N D E K F G L - - M L N E L
sp:Q16539:MK14_HUMAN	E Q N S F Y R R K M K H V I G H M V Q K L D H F A N D E K F G L D Y M L N E L
tr:G3V617:G3V617_RAT	E Q N S F Y R R K M K H V I G H M V Q K L D H F A N D E K F G L D Y M L N E L
sp:P47811:MK14_MOUSE	E Q N S F Y R R K M K H V I G H M V Q K L D H F A N D E K F G L D Y M L N E L
sp:O61443:MK14B_DROME	E Q N Q V Y R R K M D H V I G H M I Q K L D H F A N D E R F G L E Y M L N E L
sp:Q17446:PMK1_CAEEL	N I N T E Y R R R M C H I I D M M L Q R L D H F A N D E K F G L D Y M L N E L
sp:P32485:HOG1_YEAST	N N D M T Y R K K L R H L I C E Q L R P L Q F Y L N N D K F G L D Y M L T E M

protein	whole protein ident	whole protein simil	domain-based ident	domain-based simil	contact-based ident	contact-based simil
PDB:1ove:chainA:Q16539	1.0	1.0	1.0	1.0	1.0	1.0
PDB:1wbs:chainA:Q16539	1.0	1.0	1.0	1.0	1.0	1.0
PDB:1wbv:chainA:Q16539	1.0	1.0	1.0	1.0	1.0	1.0
PDB:2i0h:chainA:Q16539	1.0	1.0	1.0	1.0	1.0	1.0
PDB:2zaz:chainA:Q16539	1.0	1.0	1.0	1.0	0.99	0.97
PDB:2zb0:chainA:Q16539	1.0	1.0	1.0	1.0	1.0	1.0
PDB:2zb1:chainA:Q16539	1.0	1.0	1.0	1.0	0.99	0.97
PDB:3d7z:chainA:Q16539	1.0	1.0	1.0	1.0	1.0	1.0
PDB:3d83:chainA:Q16539	0.99	1.0	0.99	1.0	1.0	1.0
PDB:3e92:chainA:Q16539	1.0	1.0	1.0	1.0	1.0	1.0
PDB:3e93:chainA:Q16539	1.0	1.0	1.0	1.0	1.0	1.0
PDB:3hv3:chainA:Q16539	0.99	1.0	0.99	1.0	0.94	0.91
PDB:3iph:chainA:Q16539	0.99	1.0	0.99	1.0	1.0	1.0
PDB:3mpt:chainA:Q16539	1.0	1.0	1.0	1.0	1.0	1.0
PDB:4ewq:chainA:Q16539	1.0	1.0	1.0	1.0	0.97	0.94
PDB:4f9w:chainA:Q16539	1.0	1.0	1.0	1.0	0.97	0.94
sp:Q16539:MK14_HUMAN	1.0	1.0	1.0	1.0	1.0	1.0
tr:G3V617:G3V617_RAT	0.99	1.0	0.99	1.0	1.0	1.0
sp:P47811:MK14_MOUSE	0.99	1.0	0.99	1.0	1.0	1.0
sp:O61443:MK14B_DROME	0.68	0.89	0.72	0.91	0.76	0.86
sp:Q17446:PMK1_CAEEL	0.62	0.85	0.67	0.9	0.65	0.77
sp:P32485:HOG1_YEAST	0.41	0.68	0.52	0.85	0.42	0.69

p38b (FBgn0024846) associated phenotypes

RU486 conditional, circadian rhythm defective, decreased cell size, developmental rate defective, drug conditional, heat stress response defective, immune response defective, locomotor behavior defective, long lived, melanotic mass phenotype, neuroanatomy defective, nutrition conditional, osmotic stress response defective, oxidative stress response defective, partially lethal - majority die, progressive, short lived, small body, starvation stress response defective

(Information from FlyBase)

p38b (UniProt:O61443) annotation

Function: Kinase involved in dpp signal transduction pathway in the process of wing morphogen-

esis when the levels of dpp are enhanced or inhibited. May down-regulate insect immunity gene expression after prolonged infection. (PubMed:10022918, PubMed:9584193).
Cofactor:Mg(2+)
Enzyme regulation: Activated by threonine and tyrosine phosphorylation by Mkk3. (PubMed:9584193).
Subcellular location: Nucleus.
Tissue specificity: At mid-embryogenesis, highest expression is seen in developing anterior and posterior midguts. Almost ubiquitous expression throughout all development.
Developmental stage: Expressed both maternally and zygotically in the embryo, expression seen in all developmental stages.
Domain: The TXY motif contains the threonine and tyrosine residues whose phosphorylation activates the MAP kinases.
Ptm: Dually phosphorylated on Thr-183 and Tyr-185, which activates the enzyme.

(Information from UniProt)

pmk-1 (WBGene00004055) associated phenotypes

cell stress response variant, drug induced gene expression variant, gene expression level reduced, lethal, life span variant, organism oxidative stress response hypersensitive, pathogen induced gene expression variant, pathogen susceptibility increased, sterile

(Information from WormBase)

pmk-1 (UniProt:Q17446) annotation

Function: Responds to activation by environmental stress and pro- inflammatory cytokines by phosphorylating downstream targets. Functions downstream of the MAPKK sek-1 and the MAPKKK nsy-1 as the MAP kinase required for pathogen resistance. (PubMed:11703092, PubMed:12142542).
Cofactor: Mg(2+) Evidence=(PubMed:11703092);
Enzyme regulation: Activated by phosphorylation on threonine and tyrosine. Inhibited by pyridinyl-imidazole related compounds. (PubMed:11703092).
Domain: The TXY motif contains the threonine and tyrosine residues whose phosphorylation activates the MAP kinases.
Ptm: Dually phosphorylated on Thr-191 and Tyr-193, which activates the enzyme.

(Information from UniProt)

BDNF/NT-3 growth factors receptor

Best gene implication in ageing for this target family came from gene Q63604 annotated in UniProt release 2014_02. Annotation GO 7568 (aging) was Inferred from Expression Pattern

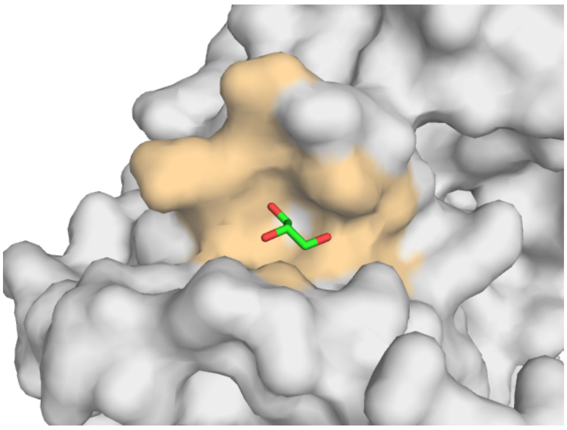

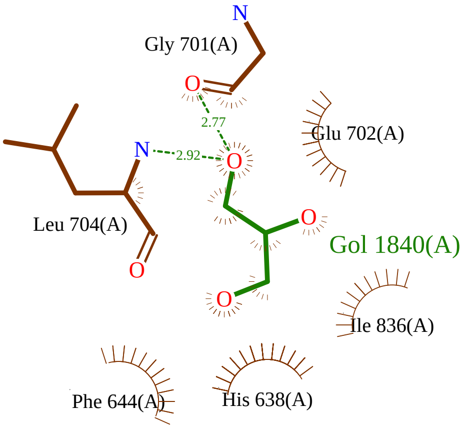

protein	amino acids contacts (binding site)
PDB:4at5:chainA:Q16620	H F G E N L I
tr:Q548C2:Q548C2_HUMAN	H F G E N L I
sp:Q16620:NTRK2_HUMAN	H F G E N L I
sp:Q63604:NTRK2_RAT	H F G E N L I
sp:P15209:NTRK2_MOUSE	H F G E N L I
tr:G4RZJ9:G4RZJ9_CAEEL	H Y A G D S S

	whole protein		domain-based		contact-based	
protein	ident	simil	ident	simil	ident	simil
PDB:4at5:chainA:Q16620	1.0	1.0	1.0	1.0	1.0	1.0
tr:Q548C2:Q548C2_HUMAN	1.0	1.0	1.0	1.0	1.0	1.0
sp:Q16620:NTRK2_HUMAN	1.0	1.0	1.0	1.0	1.0	1.0
sp:Q63604:NTRK2_RAT	0.94	0.98	0.99	1.0	1.0	1.0
sp:P15209:NTRK2_MOUSE	0.94	0.98	0.99	1.0	1.0	1.0
tr:G4RZJ9:G4RZJ9_CAEEL	0.18	0.53	0.32	0.64	0.14	0.4

Programmed cell death protein 4

Best gene implication in ageing for this target family came from gene Q53EL6 annotated in UniProt release 2014_02. Annotation GO subterm of 7568 (aging) was Inferred from Direct Assay

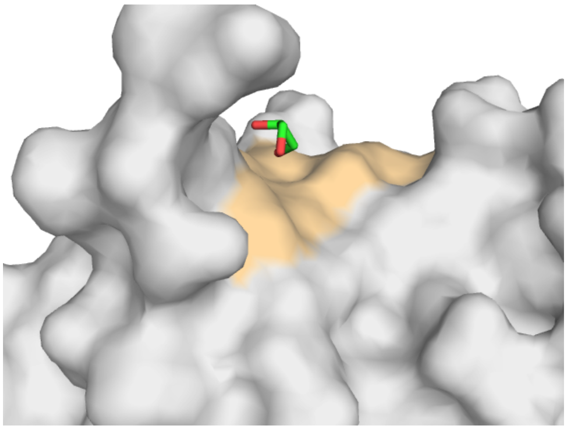

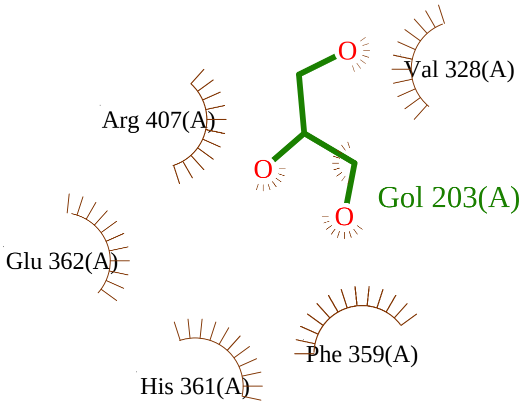

protein	amino acids contacts (binding site)
PDB:2ion:chainA:Q61823	V E D E F H E R
PDB:2nsz:chainA:Q61823	V E D E F H E R
sp:Q53EL6:PDCD4_HUMAN	V E D E F H E R
sp:Q9JID1:PDCD4_RAT	V E D E F H E R
tr:B3DM93:B3DM93_RAT	V E D E F H E R
sp:Q61823:PDCD4_MOUSE	V E D E F H E R
tr:Q9VY91:Q9VY91_DROME	T E D E Y H E R

	whole protein		domain-based		contact-based	
protein	ident	simil	ident	simil	ident	simil
PDB:2ion:chainA:Q61823	1.0	1.0	1.0	1.0	1.0	1.0
PDB:2nsz:chainA:Q61823	1.0	1.0	1.0	1.0	1.0	1.0
sp:Q53EL6:PDCD4_HUMAN	0.97	0.99	0.98	1.0	1.0	1.0
sp:Q9JID1:PDCD4_RAT	0.99	1.0	0.98	1.0	1.0	1.0
tr:B3DM93:B3DM93_RAT	0.99	1.0	0.98	1.0	1.0	1.0
sp:Q61823:PDCD4_MOUSE	1.0	1.0	1.0	1.0	1.0	1.0
tr:Q9VY91:Q9VY91_DROME	0.37	0.72	0.44	0.82	0.75	0.89

Pdcd4 (FBgn0030520) associated phenotypes

locomotor behavior defective, long lived, male fertile, starvation stress response defective, temperature response defective

(Information from FlyBase)

Aprataxin

Best gene implication in ageing for this target family came from gene Q08702 via mapping the annotation from Ensembl YOR258W via mapping the annotation from EntrezGene 854432 via mapping the annotation from GenAgeModels 1072 annotated in GenAge release 17.

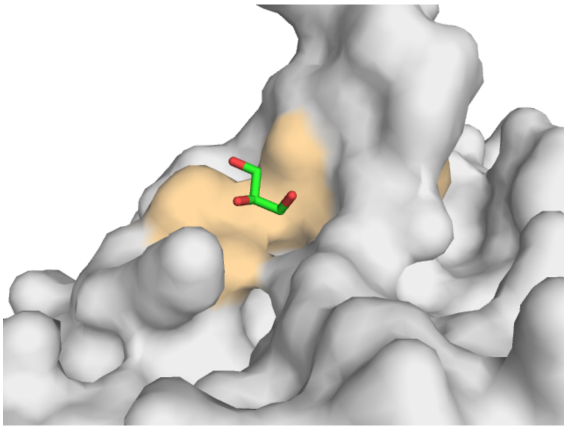

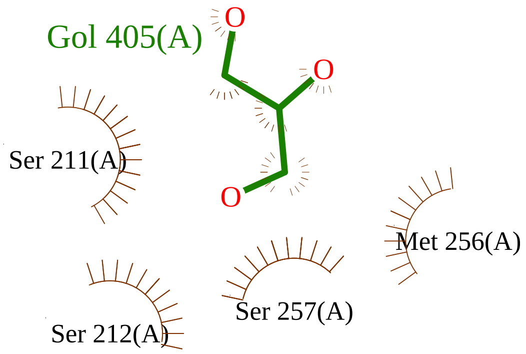

protein	amino acids contacts (binding site)			
PDB:4ndi:chainA:Q7Z2E3	S	S	M	S
tr:F5HRF8:F5HRF8_HUMAN	S	S	M	S
sp:Q7Z2E3:APTX_HUMAN	S	S	M	S
sp:Q8K4H4:APTX_RAT	S	S	M	S
sp:Q7TQC5:APTX_MOUSE	S	S	M	S
sp:Q8MSG8:APTX_DROME	P	S	M	Q
sp:Q08702:APTX_YEAST	-	-	M	A

	whole protein		domain-based		contact-based	
protein	ident	simil	ident	simil	ident	simil
PDB:4ndi:chainA:Q7Z2E3	0.97	0.99	0.99	1.0	1.0	1.0
tr:F5HRF8:F5HRF8_HUMAN	0.81	0.81	1.0	1.0	1.0	1.0
sp:Q7Z2E3:APTX_HUMAN	1.0	1.0	1.0	1.0	1.0	1.0
sp:Q8K4H4:APTX_RAT	0.78	0.88	0.91	0.97	1.0	1.0
sp:Q7TQC5:APTX_MOUSE	0.82	0.91	0.91	0.97	1.0	1.0
sp:Q8MSG8:APTX_DROME	0.09	0.17	0.41	0.76	0.5	0.67
sp:Q08702:APTX_YEAST	0.14	0.31	0.23	0.54	0.25	0.0

CG5316 (UniProt:Q8MSG8) annotation
Function: DNA-binding protein involved in single-strand DNA break repair, double-strand DNA break repair and base excision repair. Resolves abortive DNA ligation intermediates formed either at base excision sites, or when DNA ligases attempt to repair non- ligatable breaks induced by reactive oxygen species. Catalyzes the release of adenylate groups covalently linked to 5'-phosphate termini, resulting in the production of 5'-phosphate termini that can be efficiently rejoined (By similarity).
Subcellular location: Nucleus
Domain: The HIT domain is required for enzymatic activity.
Domain: The C2H2-type zinc finger mediates DNA-binding.

(Information from UniProt)

NAD-dependent protein deacetylase sirtuin-6

Best gene implication in ageing for this target family came from gene Q9VH08 annotated in UniProt release 2014_02. Annotation GO 8340 (determination of adult lifespan) was Inferred from Mutant Phenotype

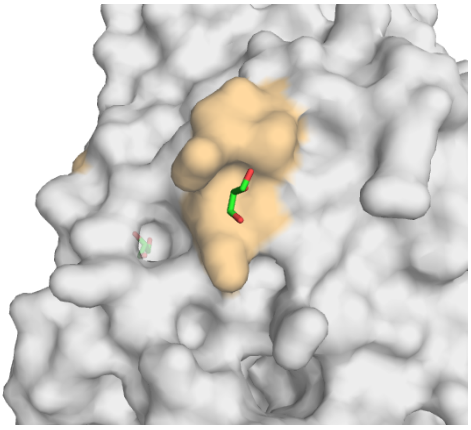

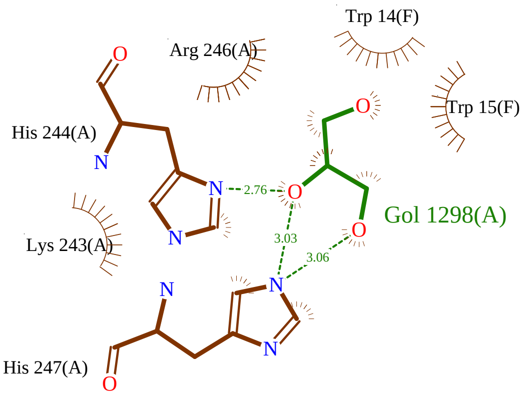

protein	amino acids contacts (binding site)
PDB:3zg6:chainA:Q8N6T7	E T D G P K H R H
sp:Q8N6T7:SIR6_HUMAN	E T D G P K H R H
tr:Q4FZY2:Q4FZY2_RAT	E T D G P K H R Q
sp:P59941:SIR6_MOUSE	E T D G P K H R Q
sp:Q9VH08:SIR6_DROME	E T D G P K H K K
sp:Q95Q89:SIR24_CAEEL	E T D G K A H K I

	whole protein		domain-based		contact-based	
protein	ident	simil	ident	simil	ident	simil
PDB:3zg6:chainA:Q8N6T7	1.0	1.0	1.0	1.0	1.0	1.0
sp:Q8N6T7:SIR6_HUMAN	1.0	1.0	1.0	1.0	1.0	1.0
tr:Q4FZY2:Q4FZY2_RAT	0.83	0.9	0.93	0.98	0.89	0.97
sp:P59941:SIR6_MOUSE	0.83	0.91	0.95	0.99	0.89	0.97
sp:Q9VH08:SIR6_DROME	0.42	0.71	0.52	0.83	0.78	0.93
sp:Q95Q89:SIR24_CAEEL	0.3	0.62	0.37	0.76	0.56	0.62

Sirt6 (FBgn0037802) associated phenotypes

short lived

(Information from FlyBase)

Sirt6 (UniProt:Q9VH08) annotation

Function: NAD-dependent protein deacetylase (By similarity). May be involved in the regulation of life span. (, PubMed:17159295).
Cofactor: Zn(2+)Note=Binds 1 zinc ion per subunit. ;
Disruption phenotype: Causes lethality during development. Induced silencing shortens life span. (PubMed:17159295).

(Information from UniProt)

sir-2.4 (WBGene00004803) associated phenotypes

lethal, sterile

(Information from WormBase)

sir-2.4 (UniProt:Q95Q89) annotation

Function: NAD-dependent protein deacetylase.
Cofactor: Zn(2+)Note=Binds 1 zinc ion per subunit. ;

(Information from UniProt)

Heterogeneous nuclear ribonucleoprotein L

Best gene implication in ageing for this target family came from gene Q0E914 annotated in UniProt release 2014_02. Annotation GO 8340 (determination of adult lifespan) was Inferred from Mutant

Phenotype

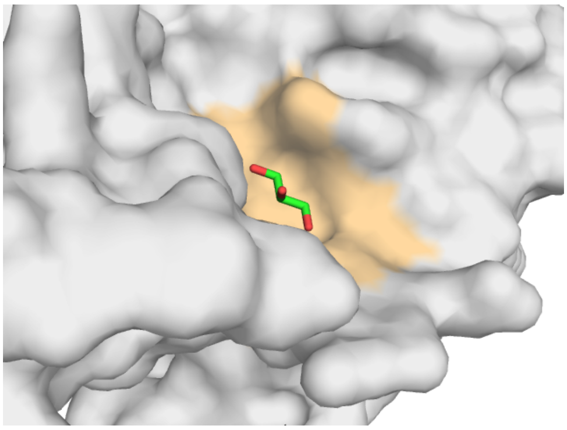

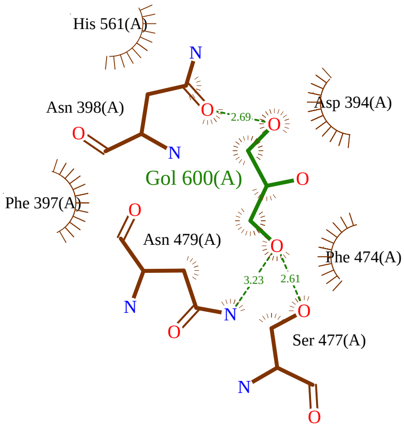

protein	amino acids contacts (binding site)
PDB:3s01:chainA:Q8R081	D F N F S N H
tr:Q6NTA2:Q6NTA2_HUMAN	D F N F S N H
sp:P14866:HNRPL_HUMAN	D F N F S N H
tr:Q5U1Y5:Q5U1Y5_RAT	D F N F S N H
tr:F1M040:F1M040_RAT	D F N F S N H
tr:F2Z3R2:F2Z3R2_RAT	D F N F S N H
tr:F1LQ48:F1LQ48_RAT	D F N F S N H
tr:G3UY38:G3UY38_MOUSE	D F N F S N H
sp:Q8R081:HNRPL_MOUSE	D F N F S N H
tr:Q0E914:Q0E914_DROME	D F N Y S N H
tr:A1ZBN3:A1ZBN3_DROME	D F N Y S N H
tr:Q8IHH3:Q8IHH3_DROME	D F N Y S N H
tr:D5AEJ9:D5AEJ9_DROME	D F N Y S N H
tr:Q7JMZ7:Q7JMZ7_DROME	D F N Y S N H
tr:Q6NND8:Q6NND8_DROME	D F N Y S N H
tr:Q95QR5:Q95QR5_CAEEL	D F N Y S N H

	whole protein		domain-based		contact-based	
protein	ident	simil	ident	simil	ident	simil
PDB:3s01:chainA:Q8R081	1.0	1.0	1.0	1.0	1.0	1.0
tr:Q6NTA2:Q6NTA2_HUMAN	0.95	0.96	0.99	1.0	1.0	1.0
sp:P14866:HNRPL_HUMAN	0.98	0.99	0.99	1.0	1.0	1.0
tr:Q5U1Y5:Q5U1Y5_RAT	0.42	0.42	1.0	1.0	1.0	1.0
tr:F1M040:F1M040_RAT	0.65	0.72	0.89	0.95	1.0	1.0
tr:F2Z3R2:F2Z3R2_RAT	1.0	1.0	1.0	1.0	1.0	1.0
tr:F1LQ48:F1LQ48_RAT	0.94	0.94	1.0	1.0	1.0	1.0
tr:G3UY38:G3UY38_MOUSE	0.78	0.78	1.0	1.0	1.0	1.0
sp:Q8R081:HNRPL_MOUSE	1.0	1.0	1.0	1.0	1.0	1.0
tr:Q0E914:Q0E914_DROME	0.15	0.29	0.39	0.72	0.86	0.97
tr:A1ZBN3:A1ZBN3_DROME	0.16	0.38	0.39	0.72	0.86	0.97
tr:Q8IHH3:Q8IHH3_DROME	0.16	0.39	0.39	0.72	0.86	0.97
tr:D5AEJ9:D5AEJ9_DROME	0.17	0.43	0.39	0.72	0.86	0.97
tr:Q7JMZ7:Q7JMZ7_DROME	0.25	0.49	0.39	0.72	0.86	0.97
tr:Q6NND8:Q6NND8_DROME	0.25	0.5	0.39	0.72	0.86	0.97
tr:Q95QR5:Q95QR5_CAEEL	0.25	0.5	0.32	0.7	0.86	0.97

sm (FBgn0003435) associated phenotypes
feeding behavior defective, long lived, neuroanatomy defective, partially lethal - majority die, short lived, some die during pupal stage

(Information from FlyBase)

NAD-dependent protein deacetylase sirtuin-1

Best gene implication in ageing for this target family came from gene Q9VK34 annotated in UniProt release 2014_02. Annotation GO 8340 (determination of adult lifespan) was Inferred from Mutant Phenotype

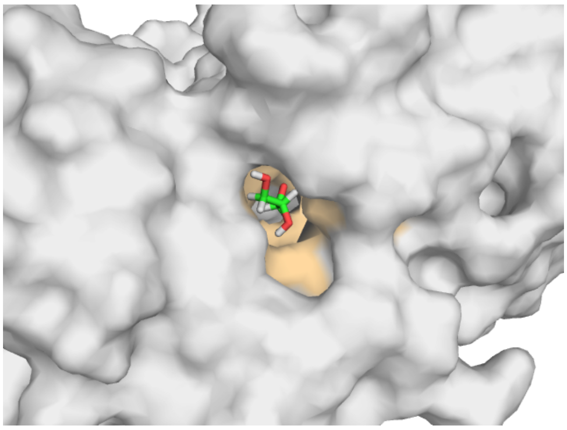

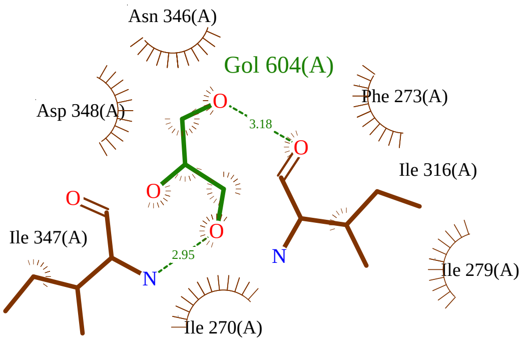

protein	amino acids contacts (binding site)
PDB:4kxq:chainA:Q96EB6	I F I I N I D
tr:A8K128:A8K128_HUMAN	I F I I N I D
sp:Q96EB6:SIR1_HUMAN	I F I I N I D
tr:F1M3P2:F1M3P2_RAT	I F I I N I D
tr:F1LTP2:F1LTP2_RAT	I F I I N I D
tr:Q3UNI1:Q3UNI1_MOUSE	I F I I N I D
tr:Q53Z05:Q53Z05_MOUSE	I F I I N I D
sp:Q923E4:SIR1_MOUSE	I F I I N I D
tr:Q86LE9:Q86LE9_DROME	I F I I N I D
sp:Q9VK34:SIR2_DROME	I F I I N I D
tr:I1V510:I1V510_DROME	I F I I N I D
tr:D3YT50:D3YT50_CAEEL	I F I I N I D
sp:Q21921:SIR2_CAEEL	I F I I N I D
sp:P53685:HST1_YEAST	I F F V N I D
sp:P06700:SIR2_YEAST	I F F V N I D

	whole protein		domain-based		contact-based	
protein	ident	simil	ident	simil	ident	simil
PDB:4kxq:chainA:Q96EB6	0.99	1.0	1.0	1.0	1.0	1.0
tr:A8K128:A8K128_HUMAN	0.74	0.74	1.0	1.0	1.0	1.0
sp:Q96EB6:SIR1_HUMAN	1.0	1.0	1.0	1.0	1.0	1.0
tr:F1M3P2:F1M3P2_RAT	0.53	0.55	1.0	1.0	1.0	1.0
tr:F1LTP2:F1LTP2_RAT	0.74	0.78	1.0	1.0	1.0	1.0
tr:Q3UNI1:Q3UNI1_MOUSE	0.79	0.89	0.99	1.0	1.0	1.0
tr:Q53Z05:Q53Z05_MOUSE	0.84	0.94	0.99	1.0	1.0	1.0
sp:Q923E4:SIR1_MOUSE	0.84	0.94	0.99	1.0	1.0	1.0
tr:Q86LE9:Q86LE9_DROME	0.27	0.44	0.74	0.93	1.0	1.0
sp:Q9VK34:SIR2_DROME	0.29	0.59	0.74	0.93	1.0	1.0
tr:I1V510:I1V510_DROME	0.29	0.59	0.74	0.93	1.0	1.0
tr:D3YT50:D3YT50_CAEEL	0.24	0.48	0.65	0.9	1.0	1.0
sp:Q21921:SIR2_CAEEL	0.24	0.5	0.65	0.9	1.0	1.0
sp:P53685:HST1_YEAST	0.16	0.34	0.47	0.8	0.71	0.92
sp:P06700:SIR2_YEAST	0.15	0.37	0.45	0.8	0.71	0.92

Sir2 (FBgn0024291) associated phenotypes

RU486 conditional, chemical resistant, conditional, dominant, enhancer of variegation, feeding behavior defective, hypoactive, increased cell death, long lived, male limited, non-suppressor of variegation, partially lethal - majority die, short lived, some die during pupal stage, suppressor of variegation

(Information from FlyBase)

Sir2 (UniProt:Q9VK34) annotation
Function: NAD-dependent histone deacetylase involved in heterochromatic silencing. Mildly suppresses the heterochromatin- mediated silencing phenomenon known as position-effect variegation (PEV). Required for epigenetic silencing of the polycomb group proteins. Has histone H4 deacetylase activity in vitro. Required maternally for establishing proper segmentation of the embryo. Involved in sex determination. May be involved in the regulation of life span. (PubMed:11281647, PubMed:12086602, PubMed:12663533, PubMed:15498488, PubMed:15520384, PubMed:17159295).
Cofactor: Zn(2+)Note=Binds 1 zinc ion per subunit. ;
Biophysicochemical properties: pH dependence: Optimum pH is 9. (PubMed:11281647);
Subunit: Interacts with the transcriptional repressors hairy (h) and deadpan (dpn); via basic domains. Associates with the Esc/E(z) histone methyltransferase complex. Interacts directly with E(z) and Rpd3. (PubMed:12086602, PubMed:15498488).
Subcellular location: Cytoplasm. Nucleus.
Disruption phenotype: Causes lethality during development. Induced silencing shortens life span. (PubMed:17159295).

(Information from UniProt)

sir-2.1 (WBGene00004800) associated phenotypes
drug response variant, fat content increased, organism oxidative stress response variant, protein expression increased, shortened life span

(Information from WormBase)

sir-2.1 (UniProt:Q21921) annotation
Function: NAD-dependent deacetylase (By similarity). Functions upstream of daf-16 in the insulin-like signaling pathway, promoting daf-16 mediated transcriptional activation and increased life-span. May also regulate life-span independently of daf-16 by modulating the transcription of genes involved in the stress response of the endoplasmic reticulum (ER). (, PubMed:11242085, PubMed:15793589, PubMed:16256736, PubMed:16280150, PubMed:16777605).
Cofactor: Zn(2+)Note=Binds 1 zinc ion per subunit. ;
Subunit: Interacts with ftt-2 and par-5. Interacts with daf-16 following heat-shock, which causes daf-16 to accumulate in the nucleus. Interaction with daf-16 is promoted by ftt-2. (PubMed:16777605).
Subcellular location: Nucleus ECO:0000269—PubMed:16280150, PubMed:16777605).
Developmental stage: Expressed in neurons of the head and tail from embryo to adult. Expressed in the hypodermis from the three- fold stage of embryogenesis; expression in the hypodermis subsequently decreases at L3 and is undetectable in adults. (PubMed:16280150).

(Information from UniProt)

Protein deglycase DJ-1

Best gene implication in ageing for this target family came from gene Q6NMT9 via mapping the annotation from Ensembl FBgn0033885 via mapping the annotation from EntrezGene 36543 via mapping the annotation from GenAgeModels 0178 annotated in GenAge release 17.

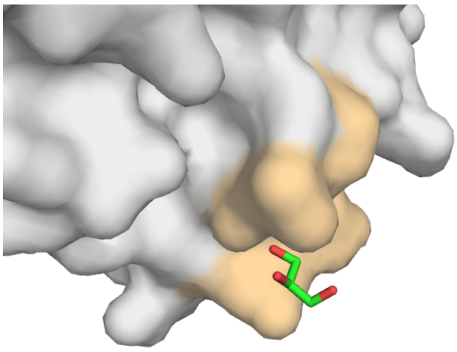

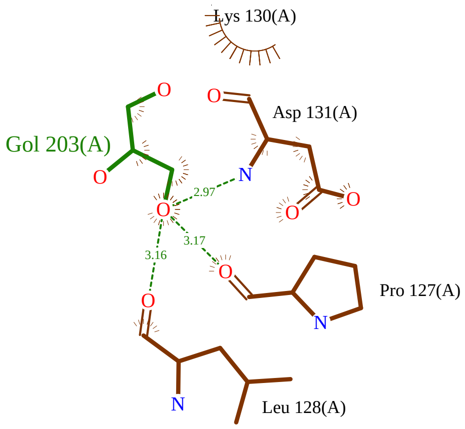

protein	amino acids contacts (binding site)			
PDB:4p35:chainA:Q99497	P	L	K	D
tr:K7ELW0:K7ELW0_HUMAN	P	L	K	D
tr:K7EN27:K7EN27_HUMAN	P	L	K	D
sp:Q99497:PARK7_HUMAN	P	L	K	D
sp:O88767:PARK7_RAT	P	L	K	D
tr:A2A815:A2A815_MOUSE	P	L	K	D
tr:A2A813:A2A813_MOUSE	P	L	K	D
sp:Q99LX0:PARK7_MOUSE	P	L	K	D
tr:Q6NMT9:Q6NMT9_DROME	P	D	K	P
tr:Q9VA37:Q9VA37_DROME	P	S	K	P
tr:A1Z9J4:A1Z9J4_DROME	P	D	K	P
tr:P90994:P90994_CAEEL	P	S	K	E
tr:O16228:O16228_CAEEL	Y	T	K	D

	whole protein		domain-based		contact-based	
protein	ident	simil	ident	simil	ident	simil
PDB:4p35:chainA:Q99497	1.0	1.0	1.0	1.0	1.0	1.0
tr:K7ELW0:K7ELW0_HUMAN	0.89	0.89	0.89	0.89	1.0	1.0
tr:K7EN27:K7EN27_HUMAN	0.75	0.75	0.76	0.76	1.0	1.0
sp:Q99497:PARK7_HUMAN	1.0	1.0	1.0	1.0	1.0	1.0
sp:O88767:PARK7_RAT	0.92	0.98	0.91	0.98	1.0	1.0
tr:A2A815:A2A815_MOUSE	0.67	0.7	0.67	0.71	1.0	1.0
tr:A2A813:A2A813_MOUSE	0.83	0.9	0.84	0.91	1.0	1.0
sp:Q99LX0:PARK7_MOUSE	0.92	0.98	0.91	0.98	1.0	1.0
tr:Q6NMT9:Q6NMT9_DROME	0.35	0.5	0.42	0.6	0.5	0.35
tr:Q9VA37:Q9VA37_DROME	0.47	0.74	0.52	0.82	0.5	0.41
tr:A1Z9J4:A1Z9J4_DROME	0.46	0.69	0.54	0.81	0.5	0.35
tr:P90994:P90994_CAEEL	0.52	0.82	0.52	0.82	0.5	0.56
tr:O16228:O16228_CAEEL	0.43	0.78	0.44	0.79	0.5	0.53

dj-1beta (FBgn0039802) associated phenotypes

chemical resistant, chemical sensitive, locomotor behavior defective, long lived, oxidative stress response defective, radiation sensitive, short lived, stress response defective

(Information from FlyBase)

DJ-1alpha (FBgn0033885) associated phenotypes

chemical resistant, chemical sensitive, increased cell death, locomotor behavior defective, short lived, stress response defective

(Information from FlyBase)

djr-1.1 (WBGene00015184) associated phenotypes

solitary feeding increased

(Information from WormBase)

djr-1.1 (UniProt:P90994) annotation
Function: Catalyzes the conversion of methylglyoxal (MG) or glyoxal (GO) to D-lactate or glycolic acid respectively in a single glutathione (GSH)-independent step. May play a role in detoxifying endogenously produced glyoxals. Involved in protection against glyoxal-induced cell death. (PubMed:22523093).
Biophysicochemical properties: Kinetic parameters: KM=0.3 mM for methylglyoxal (PubMed:22523093); KM=3.61 mM for glyoxal (PubMed:22523093); Note=kcat is 13.8 min(-1) with methylglyoxal as substrate and 356.4 min(-1) with glyoxal as substrate. (PubMed:22523093);
Subcellular location: Cytoplasm (PubMed:22523093). Nucleus (PubMed:22523093). Note=Ubiquitously localized throughout the whole cells with higher expression in the nucleus. (PubMed:22523093).
Tissue specificity: Expressed exclusively in the intestine. (PubMed:22523093).
(Information from UniProt)

djr-1.2 (UniProt:O16228) annotation
Function: Catalyzes the conversion of methylglyoxal (MG) or glyoxal (GO) to D-lactate or glycolic acid respectively in a single glutathione (GSH)-independent step. May play a role in detoxifying endogenously produced glyoxals. Involved in protection against glyoxal-induced cell death. Protects dopaminergic neurons from glyoxal-dependent neuronal degeneration. (PubMed:22523093, PubMed:23624124).
Biophysicochemical properties: Kinetic parameters: KM=0.39 mM for methylglyoxal (PubMed:22523093); KM=0.78 mM for glyoxal (PubMed:22523093); Note=kcat is 60.0 min(-1) with methylglyoxal as substrate and 146.4 min(-1) with glyoxal as substrate. (PubMed:22523093);
Subcellular location: Cytoplasm (PubMed:22523093).
Tissue specificity: Expressed in various tissues, including pharyngeal muscles, pharynx-intestinal valve, ventral nerve cord, spermatheca, rectal gland, inner labial (IL) cells of head neurons, phasmid (PHA/PHB) neurons in tail and supporting sheath/socket cells, as well as in head mesodermal cells (HMC), excretory canals and coelomocytes. (PubMed:22523093).
Induction: Induced by DAF-16 during starvation as well as in the dauer stage. (PubMed:23624124).
(Information from UniProt)

Mitogen-activated protein kinase kinase kinase 5

Best gene implication in ageing for this target family came from gene P23561 via mapping the annotation from Ensembl YLR362W via mapping the annotation from EntrezGene 851076 via mapping the annotation from GenAgeModels 1609 annotated in GenAge release 17.

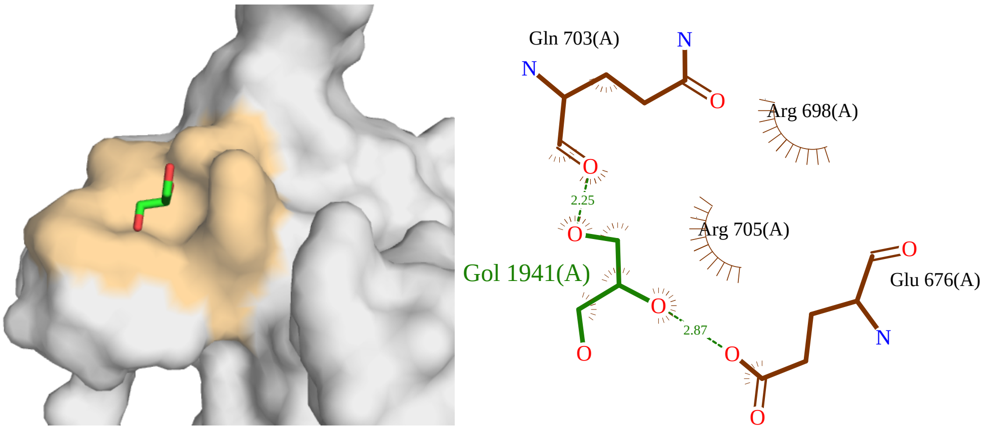

protein	amino acids contacts (binding site)
PDB:4bf2:chainA:Q99683	E R Q R H L F E
PDB:4bhn:chainA:Q99683	E R Q R H L F E
PDB:4bib:chainA:Q99683	E R Q R H L F E
PDB:4bic:chainA:Q99683	E R Q R H L F E
PDB:4bie:chainA:Q99683	E R Q R H L F E
sp:Q99683:M3K5_HUMAN	E R Q R H L F E
tr:D3ZW27:D3ZW27_RAT	E R Q R H L F E
tr:D3ZZH6:D3ZZH6_RAT	E R Q R H L F E
tr:E9PWG9:E9PWG9_MOUSE	E R Q R H L F E
sp:O35099:M3K5_MOUSE	E R Q R H L F E
tr:Q24458:Q24458_DROME	D R Q R H L F A
tr:Q7KSB7:Q7KSB7_DROME	D R Q R H L F A
tr:Q8I6M5:Q8I6M5_DROME	D R Q R H L F A
tr:Q9VDS9:Q9VDS9_DROME	D R Q R H L F A
tr:Q4QQD0:Q4QQD0_DROME	D R Q R H L F A
tr:B9EQQ2:B9EQQ2_DROME	D R Q R H L F A
tr:Q21029:Q21029_CAEEL	E R Q Q H K F L
sp:P23561:STE11_YEAST	D M G L T R F E

	whole protein		domain-based		contact-based	
protein	ident	simil	ident	simil	ident	simil
PDB:4bf2:chainA:Q99683	1.0	1.0	1.0	1.0	1.0	1.0
PDB:4bhn:chainA:Q99683	1.0	1.0	1.0	1.0	1.0	1.0
PDB:4bib:chainA:Q99683	1.0	1.0	1.0	1.0	1.0	1.0
PDB:4bic:chainA:Q99683	0.99	1.0	0.99	1.0	1.0	1.0
PDB:4bie:chainA:Q99683	0.99	1.0	0.99	1.0	1.0	1.0
sp:Q99683:M3K5_HUMAN	1.0	1.0	1.0	1.0	1.0	1.0
tr:D3ZW27:D3ZW27_RAT	0.91	0.96	0.97	0.99	1.0	1.0
tr:D3ZZH6:D3ZZH6_RAT	0.92	0.97	0.97	0.99	1.0	1.0
tr:E9PWG9:E9PWG9_MOUSE	0.94	0.97	0.98	0.99	1.0	1.0
sp:O35099:M3K5_MOUSE	0.94	0.98	0.98	0.99	1.0	1.0
tr:Q24458:Q24458_DROME	0.22	0.38	0.65	0.88	0.75	0.82
tr:Q7KSB7:Q7KSB7_DROME	0.33	0.59	0.65	0.88	0.75	0.82
tr:Q8I6M5:Q8I6M5_DROME	0.36	0.68	0.65	0.88	0.75	0.82
tr:Q9VDS9:Q9VDS9_DROME	0.36	0.68	0.65	0.88	0.75	0.82
tr:Q4QQD0:Q4QQD0_DROME	0.36	0.68	0.65	0.88	0.75	0.82
tr:B9EQQ2:B9EQQ2_DROME	0.36	0.68	0.65	0.88	0.75	0.82
tr:Q21029:Q21029_CAEEL	0.31	0.65	0.56	0.82	0.63	0.67
sp:P23561:STE11_YEAST	0.1	0.28	0.28	0.59	0.25	0.56

Pk92B (FBgn0014006) associated phenotypes

heat sensitive

(Information from FlyBase)

nsy-1 (WBGene00003822) associated phenotypes

aldicarb hypersensitive, anoxia resistant, benzaldehyde chemotaxis variant, butanone chemotaxis variant, cell stress response variant, egg laying defective, egg laying response to food variant, gene expression level reduced, genetic pathway activation variant, lethal, levamisole hypersensitive, loss of asymmetry AWC, odorant adaptation variant, organism high sodium chloride hypersensitive, organism stress response variant, pathogen induced gene expression variant, pathogen susceptibility increased, pentanedione chemotaxis variant, protein phosphorylation reduced, pyrazine chemotaxis variant, sterile, transgene expression reduced

(Information from WormBase)

3-hydroxyacyl-CoA dehydrogenase type-2

Best gene implication in ageing for this target family came from gene B0BMW2 via mapping the annotation from RGD 69231 annotated in RGD 2014-03-11. Annotation GO subterm of 7568 (aging) was Inferred from Expression Pattern

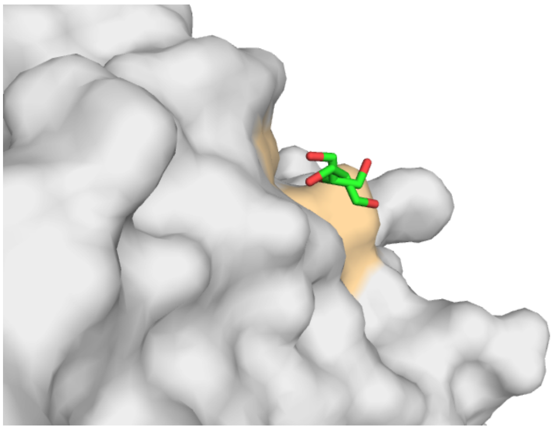

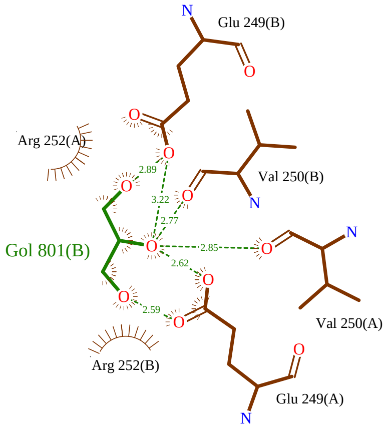

protein	amino acids contacts (binding site)		
PDB:2o23:chainA:Q99714	E	V	R
sp:Q99714:HCD2_HUMAN	E	V	R
tr:B0BMW2:B0BMW2_RAT	E	V	R
tr:Q99N15:Q99N15_MOUSE	E	V	R
tr:A2AFQ2:A2AFQ2_MOUSE	E	V	R
tr:D5A7S2:D5A7S2_DROME	E	V	R
sp:O18404:HCD2_DROME	E	V	R
tr:Q19102:Q19102_CAEEL	E	T	R

	whole protein		domain-based		contact-based	
protein	ident	simil	ident	simil	ident	simil
PDB:2o23:chainA:Q99714	0.99	0.99	1.0	1.0	1.0	1.0
sp:Q99714:HCD2_HUMAN	1.0	1.0	1.0	1.0	1.0	1.0
tr:B0BMW2:B0BMW2_RAT	0.88	0.96	0.88	0.96	1.0	1.0
tr:Q99N15:Q99N15_MOUSE	0.88	0.96	0.88	0.97	1.0	1.0
tr:A2AFQ2:A2AFQ2_MOUSE	0.84	0.93	0.85	0.93	1.0	1.0
tr:D5A7S2:D5A7S2_DROME	0.36	0.45	0.37	0.45	1.0	1.0
sp:O18404:HCD2_DROME	0.67	0.88	0.68	0.89	1.0	1.0
tr:Q19102:Q19102_CAEEL	0.59	0.86	0.6	0.87	0.67	0.68

scu (FBgn0021765) associated phenotypes

germline clone, lethal - all die before end of pupal stage, neurophysiology defective, polyphasic, some die during pupal stage

(Information from FlyBase)

scu (UniProt:O18404) annotation

Function: May function in mitochondrial tRNA maturation. Catalyzes the beta-oxidation at position 17 of androgens and estrogens, and has 3-alpha-hydroxysteroid dehydrogenase activity with androsterone. Catalyzes the third step in the beta-oxidation of fatty acids. Carries out oxidative conversions of 7-beta- hydroxylated bile acids. Also exhibits 20-beta-OH and 21-OH dehydrogenase activities with C21 steroids. Required for cell survival during embryonic development. May play a role in germline formation. (PubMed:12917011, PubMed:9585418).

Biophysicochemical properties: Kinetic parameters: KM=33.7 uM for acetoacetyl-CoA (in the presence of 0.2 mM NADH, at pH 6.4 and 25 degrees Celsius) (PubMed:12917011); KM=101 uM for beta-hydroxybutyryl-CoA (in the presence of 1 mM NAD, at pH 9.3 and 25 degrees Celsius) (PubMed:12917011); KM=37.3 uM for androsterone (in the presence of 1 mM NAD, at pH 9.3 and 25 degrees Celsius) (PubMed:12917011); KM=12.3 uM for 5-alpha-dihydrotestosterone (in the presence

of 0.2 mM NADH, at pH 6.4 and 25 degrees Celsius) (PubMed:12917011); KM=11.1 uM for 17-beta-estradiol (in the presence of 0.2 mM NADH, at pH 9.3 and 25 degrees Celsius) (PubMed:12917011); KM=9 uM for 5-alpha-pregnan-20-beta-ol-3-one (in the presence of 1 mM NAD, at pH 9.3 and 25 degrees Celsius) (PubMed:12917011); KM=3 uM for isoursodeoxycholic acid (in the presence of 1 mM NAD, at pH 9.3 and 25 degrees Celsius) (PubMed:12917011); KM=32.5 uM for NADH (in the presence of acetoacetyl-CoA, at pH 7.0 and 25 degrees Celsius) (PubMed:12917011); KM=64.4 uM for NAD (in the presence of beta-hydroxybutyryl-CoA, at pH 9.3 and 25 degrees Celsius) (PubMed:12917011); KM=124 uM for NAD (in the presence of aldosterone, at pH 9.3 and 25 degrees Celsius) (PubMed:12917011); pH dependence: Optimum pH is 9.3 for the dehydrogenase reaction, and 6.4 for the reductase reaction. (PubMed:12917011);
Subunit: Multimer.
Subcellular location: Mitochondrion Note=Localizes to the lipid droplet fraction in early embryos. (PubMed:16979555).
Tissue specificity: Found in many tissues including CNS, imaginal disks and salivary glands. Highest expression in both embryonic gonadal primordia and mature ovaries and testes. (PubMed:9585418).
Developmental stage: Expressed throughout embryonic development. In adults, expression is higher in females than in males. (PubMed:9585418).
Induction: By 20-hydroxyecdysone. (PubMed:17924685).
Disruption phenotype: Embryonic and pupal lethal. Male mutants show small testes and degenerating spermatocytes with a large accumulation of small fat-containing vesicles in the cytoplasm and almost no mitochondria. Null mutant photoreceptors fail to differentiate normally, are unable to form proper rhabdomeres and present smaller mitochondria with fewer, but swollen crestae, than wild-type cells. (PubMed:9585418).

(Information from UniProt)

Rap guanine nucleotide exchange factor 4

Best gene implication in ageing for this target family came from gene E7EVE5 via mapping the annotation from Ensembl ENSG00000091428 via mapping the annotation from EntrezGene 2 via mapping the annotation from GenAgeHuman 0139 annotated in GenAge release 17.

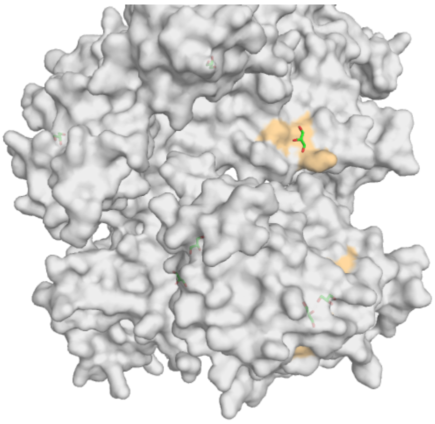

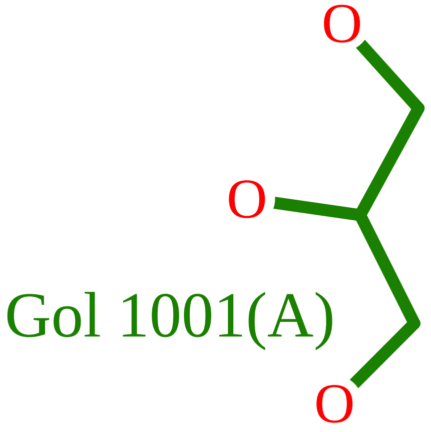

protein	amino acids contacts (binding site)
PDB:4f7z:chainA:Q9EQZ6	F F G E S R H A Y T Q G M K Y G P H V V C P R S S N C V H K K H K T K
tr:E7EVE5:E7EVE5_HUMAN	F F G E S R H A Y T Q G M K Y G P H V V C P R S S N C V H K K H K T K
sp:Q8WZA2:RPGF4_HUMAN	F F G E S R H A Y T Q G M K Y G P H V V C P R S S N C V H K K H K T K
tr:D3KR63:D3KR63_RAT	F F G E S R H A Y T Q G M K Y G P H V V C P R S S N C V H K K H K T K
tr:A2ASW4:A2ASW4_MOUSE	F F G E S R H A Y T Q G M K Y G P H V V C P R S S N C V H K K H K T K
sp:Q9EQZ6:RPGF4_MOUSE	F F G E S R H A Y T Q G M K Y G P H V V C P R S S N C V H K K H K T K
tr:Q571A8:Q571A8_MOUSE	F F G E S R H A Y T Q G M K Y G P H V V C P R S S N C V H K K H K T K
tr:A1Z6P8:A1Z6P8_DROME	F F G E S R D S V A Q G M K C G P T T V A V R S T W A V H K K H K T K
sp:P34578:RPGF1_CAEEL	F F G D - H S C S I Q A I K T A P L I V C P L D S R S T D K S H K S K

	whole protein		domain-based		contact-based	
protein	ident	simil	ident	simil	ident	simil
PDB:4f7z:chainA:Q9EQZ6	1.0	1.0	1.0	1.0	1.0	1.0
tr:E7EVE5:E7EVE5_HUMAN	0.97	0.99	0.97	0.99	1.0	1.0
sp:Q8WZA2:RPGF4_HUMAN	0.97	0.99	0.98	0.99	1.0	1.0
tr:D3KR63:D3KR63_RAT	0.99	1.0	0.99	1.0	1.0	1.0
tr:A2ASW4:A2ASW4_MOUSE	1.0	1.0	1.0	1.0	1.0	1.0
sp:Q9EQZ6:RPGF4_MOUSE	1.0	1.0	1.0	1.0	1.0	1.0
tr:Q571A8:Q571A8_MOUSE	0.98	0.98	1.0	1.0	1.0	1.0
tr:A1Z6P8:A1Z6P8_DROME	0.47	0.79	0.52	0.83	0.66	0.65
sp:P34578:RPGF1_CAEEL	0.32	0.69	0.34	0.72	0.4	0.52

Lysine-specific demethylase 7 homolog

Best gene implication in ageing for this target family came from gene Q9GYI0 via mapping the annotation from WormBase WBGene00017920 annotated in WormBase 2014-03-11. Annotation GO 8340 (determination of adult lifespan) was Inferred from Mutant Phenotype

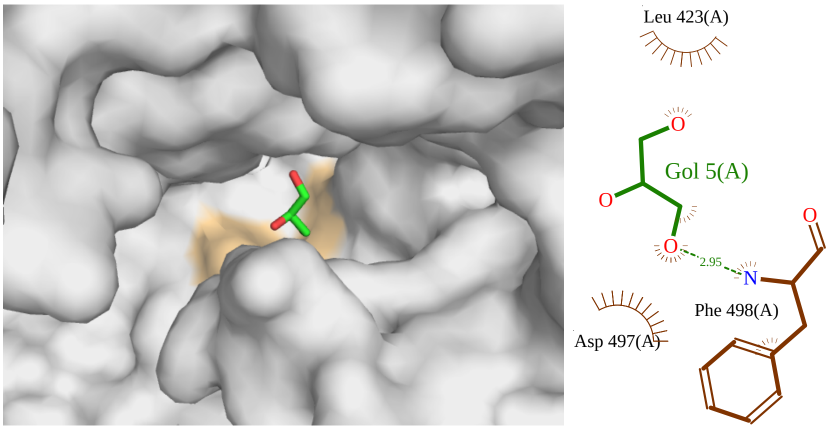

protein	amino acids contacts (binding site)
PDB:3puq:chainA:Q9GYI0	L D F
sp:Q9GYI0:KDM7_CAEEL	L D F

	whole protein		domain-based		contact-based	
protein	ident	simil	ident	simil	ident	simil
PDB:3puq:chainA:Q9GYI0	1.0	1.0	1.0	1.0	1.0	1.0
sp:Q9GYI0:KDM7_CAEEL	1.0	1.0	1.0	1.0	1.0	1.0

jmjd-1.2 (UniProt:Q9GYI0) annotation

Function: Histone demethylase required for nervous system development. Specifically demethylates dimethylated 'Lys-9' and 'Lys-27' (H3K9me2 and H3K27me2, respectively) of histone H3, thereby playing a central role in histone code. (PubMed:20346720, PubMed:20567261, PubMed:20567262).
Cofactor: Fe(2+) Evidence=(PubMed:20567261); Note=Binds 1 Fe(2+) ion per subunit. (PubMed:20567261);
Enzyme regulation: Competitively inhibited by 2-hydroxyglutarate. (PubMed:21251613).
Subcellular location: Nucleus (PubMed:20346720).

Tissue specificity: Mainly expressed in neurons. Also weakly expressed in some muscle, intestinal and hypodermal cells. (PubMed:20346720).
Domain: The PHD-type zinc finger mediates binding to H3K4me3. (PubMed:20567261, PubMed:20567262).
Disruption phenotype: Impaired locomotion. While wild-type animals move by alternating dorsal and ventral flexions along the body length, producing a regular sinusoidal track on bacteria, the track pattern left mutants is irregular, with increased wavelength (distance between successive peaks of a wave) but unchanged amplitude of the wave. An increase of H3K9me2 and H3K27me2 levels is observed. (PubMed:20346720).

(Information from UniProt)

Pleckstrin homology domain-containing family A member 3

Best gene implication in ageing for this target family came from gene Q9Y128 via mapping the annotation from Ensembl FBgn0027569 via mapping the annotation from EntrezGene 38928 via mapping the annotation from GenAgeModels 0117 annotated in GenAge release 17.

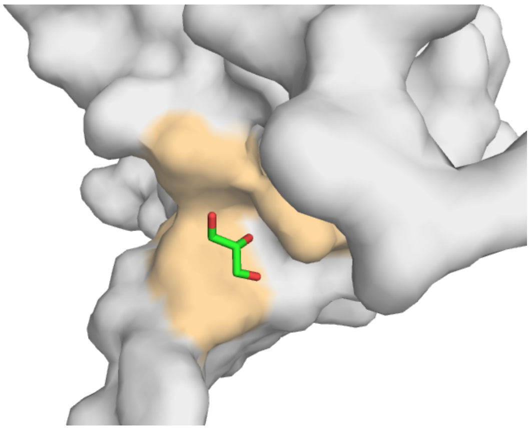

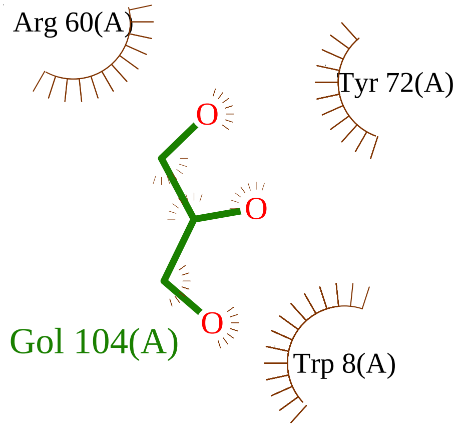

protein	amino acids contacts (binding site)
PDB:3rcp:chainA:Q9HB20	W R Y
sp:Q9HB20:PKHA3_HUMAN	W R Y
tr:B5MDU3:B5MDU3_HUMAN	W R Y
sp:Q96JA3:PKHA8_HUMAN	W R Y
tr:J3KQS5:J3KQS5_HUMAN	W R Y
sp:Q9Y5P4:C43BP_HUMAN	W R Y
tr:E9PSY1:E9PSY1_RAT	W R Y
sp:D3ZY60:PKHA8_RAT	W R Y
tr:D4ACN6:D4ACN6_RAT	W R Y
sp:Q9ERS4:PKHA3_MOUSE	W R Y
sp:Q80W71:PKHA8_MOUSE	W R Y
sp:Q9EQG9:C43BP_MOUSE	W R Y
tr:Q9Y128:Q9Y128_DROME	W R C
tr:Q93569:Q93569_CAEEL	W E Y

protein	whole protein ident	simil	domain-based ident	simil	contact-based ident	simil
PDB:3rcp:chainA:Q9HB20	0.95	0.98	0.95	0.98	1.0	1.0
sp:Q9HB20:PKHA3_HUMAN	1.0	1.0	1.0	1.0	1.0	1.0
tr:B5MDU3:B5MDU3_HUMAN	0.32	0.53	0.78	0.93	1.0	1.0
sp:Q96JA3:PKHA8_HUMAN	0.27	0.45	0.78	0.93	1.0	1.0
tr:J3KQS5:J3KQS5_HUMAN	0.26	0.43	0.78	0.93	1.0	1.0
sp:Q9Y5P4:C43BP_HUMAN	0.09	0.31	0.24	0.54	1.0	1.0
tr:E9PSY1:E9PSY1_RAT	0.91	0.97	0.98	0.99	1.0	1.0
sp:D3ZY60:PKHA8_RAT	0.27	0.45	0.78	0.93	1.0	1.0
tr:D4ACN6:D4ACN6_RAT	0.09	0.31	0.24	0.54	1.0	1.0
sp:Q9ERS4:PKHA3_MOUSE	0.91	0.96	0.98	0.99	1.0	1.0
sp:Q80W71:PKHA8_MOUSE	0.28	0.45	0.78	0.93	1.0	1.0
sp:Q9EQG9:C43BP_MOUSE	0.1	0.31	0.24	0.54	1.0	1.0
tr:Q9Y128:Q9Y128_DROME	0.1	0.32	0.22	0.51	0.67	0.1
tr:Q93569:Q93569_CAEEL	0.1	0.34	0.24	0.59	0.67	0.75

cert (FBgn0027569) associated phenotypes

heat stress response defective, locomotor behavior defective, short lived, stress response defective

(Information from FlyBase)

NAD-dependent protein deacetylase sirtuin-3, mitochondrial

Best gene implication in ageing for this target family came from gene Q9I7I7 annotated in UniProt release 2014_02. Annotation GO 8340 (determination of adult lifespan) was Inferred from Mutant Phenotype

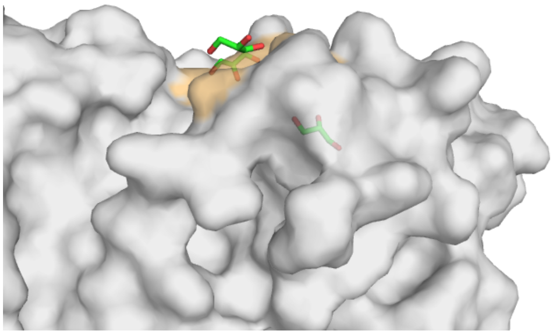

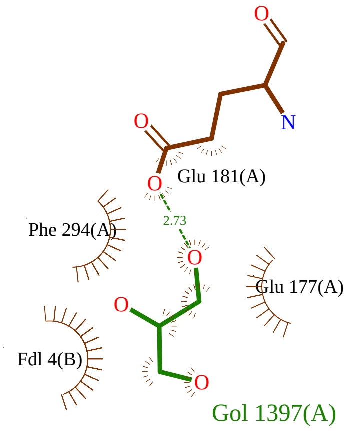

protein	amino acids contacts (binding site)
PDB:3glr:chainA:Q9NTG7	G A S P I P F R E E K N H G L Q N I D K H T C V R K P D I V F G E P L P Q R
PDB:4bn5:chainA:Q9NTG7	G A S P I P F R E E K N H G L Q N I D K H T C V R K P D I V F G E P L P Q R
PDB:4bv3:chainA:Q9NTG7	G A S P I P F R E E K N H G L Q N I D K H T C V R K P D I V F G E P L P Q R
PDB:4bvg:chainA:Q9NTG7	G A S P I P F R E E K N H G L Q N I D K H T C V R K P D I V F G E P L P Q R
PDB:4c7b:chainA:Q9NTG7	G A S P I P F R E E K N H G L Q N I D K H T C V R K P D I V F G E P L P Q R
PDB:4fvt:chainA:Q9NTG7	G A S P I P F R E E K N H G L Q N I D K H T C V R K P D I V F G E P L P Q R
PDB:4jt9:chainA:Q9NTG7	G A S P I P F R E E K N H G L Q N I D K H T C V R K P D I V F G E P L P Q R
tr:E9PK80:E9PK80_HUMAN	G A S P I P F R E E K N H G L Q N I D K H T C V R K P D I V F G E P L P Q R
sp:Q9NTG7:SIR3_HUMAN	G A S P I P F R E E K N H G L Q N I D K H T C V R K P D I V F G E P L P Q R
tr:B2RZ31:B2RZ31_RAT	G A S P I P F R E E K N H E L Q N I D K H T C V R K P D I V F G E Q L P A R
tr:C6ZII9:C6ZII9_RAT	G A S P I P F R E E K N H E L Q N I D K H T C V R K P D I V F G E Q L P A R
tr:Q4FJK3:Q4FJK3_MOUSE	G A S P I P F R E E K N H E L Q N I D K H T C V R K P D I V F G E Q L P A R
tr:D3YTK6:D3YTK6_MOUSE	G A S P I P F R E E K N H E L Q N I D K H T C V R K P D I V F G E Q L P A R
sp:Q8R104:SIR3_MOUSE	G A S P I P F R E E K N H E L Q N I D K H T C V R K P D I V F G E Q L P A R
sp:Q9I7I7:SIRT2_DROME	G A S S I P F R T D A T N G L Q N I D K H H C L K K P D I V F G E N L P K R
sp:P53686:HST2_YEAST	G A S S I P F R E D L S Q D L Q N I D L H H C I K K P A I V F G E D L P D S

	whole protein		domain-based		contact-based	
protein	ident	simil	ident	simil	ident	simil
PDB:3glr:chainA:Q9NTG7	1.0	1.0	1.0	1.0	1.0	1.0
PDB:4bn5:chainA:Q9NTG7	1.0	1.0	1.0	1.0	1.0	1.0
PDB:4bv3:chainA:Q9NTG7	1.0	1.0	1.0	1.0	1.0	1.0
PDB:4bvg:chainA:Q9NTG7	1.0	1.0	1.0	1.0	1.0	1.0
PDB:4c7b:chainA:Q9NTG7	1.0	1.0	1.0	1.0	1.0	1.0
PDB:4fvt:chainA:Q9NTG7	1.0	1.0	1.0	1.0	1.0	1.0
PDB:4jt9:chainA:Q9NTG7	1.0	1.0	1.0	1.0	1.0	1.0
tr:E9PK80:E9PK80_HUMAN	0.79	0.8	0.98	0.98	1.0	1.0
sp:Q9NTG7:SIR3_HUMAN	1.0	1.0	1.0	1.0	1.0	1.0
tr:B2RZ31:B2RZ31_RAT	0.55	0.62	0.79	0.89	0.92	0.94
tr:C6ZII9:C6ZII9_RAT	0.63	0.74	0.86	0.96	0.92	0.94
tr:Q4FJK3:Q4FJK3_MOUSE	0.55	0.62	0.79	0.89	0.92	0.94
tr:D3YTK6:D3YTK6_MOUSE	0.48	0.55	0.65	0.72	0.92	0.94
sp:Q8R104:SIR3_MOUSE	0.62	0.72	0.85	0.96	0.92	0.94
sp:Q9I7I7:SIRT2_DROME	0.34	0.55	0.46	0.7	0.71	0.84
sp:P53686:HST2_YEAST	0.26	0.49	0.35	0.64	0.63	0.75

Sirt2 (FBgn0038788) associated phenotypes

heat sensitive, lethal - all die before end of pupal stage, some die during pupal stage

(Information from FlyBase)

Sirt2 (UniProt:Q9I7I7) annotation

Function: NAD-dependent protein deacetylase (By similarity). May be involved in the regulation of life span. (, PubMed:17159295).

Cofactor: Zn(2+)Note=Binds 1 zinc ion per subunit. ;

Disruption phenotype: Causes lethality during development. Induced silencing shortens life span. (PubMed:17159295).

(Information from UniProt)

Peptidoglycan-recognition protein LE

Best gene implication in ageing for this target family came from gene Q9VXN9 annotated in UniProt release 2014_02. Annotation GO 8340 (determination of adult lifespan) was Inferred from Mutant Phenotype

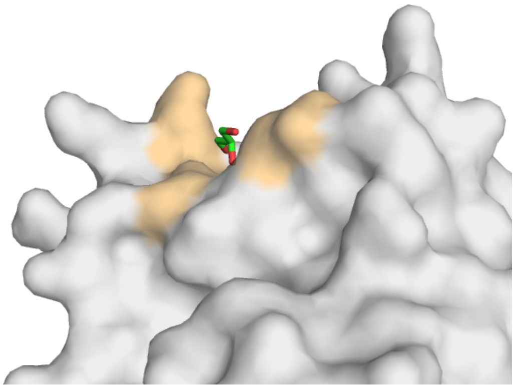

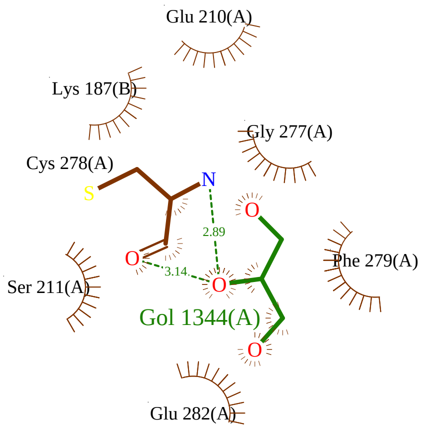

protein	amino acids contacts (binding site)					
PDB:2cb3:chainA:Q9VXN9	E	S	G	C	F	E
sp:Q96PD5:PGRP2_HUMAN	P	A	G	N	Y	A
tr:M0R485:M0R485_RAT	P	A	-	S	F	P
tr:Q54A77:Q54A77_MOUSE	P	A	G	N	Y	S
sp:Q8VCS0:PGRP2_MOUSE	P	A	G	N	Y	S
sp:Q9VXN9:PGPLE_DROME	E	S	G	C	F	E

	whole protein		domain-based		contact-based	
protein	ident	simil	ident	simil	ident	simil
PDB:2cb3:chainA:Q9VXN9	0.99	1.0	0.99	1.0	1.0	1.0
sp:Q96PD5:PGRP2_HUMAN	0.14	0.42	0.18	0.36	0.17	0.47
tr:M0R485:M0R485_RAT	0.08	0.28	0.08	0.18	0.17	0.34
tr:Q54A77:Q54A77_MOUSE	0.14	0.4	0.19	0.37	0.17	0.49
sp:Q8VCS0:PGRP2_MOUSE	0.14	0.4	0.19	0.37	0.17	0.49
sp:Q9VXN9:PGPLE_DROME	1.0	1.0	1.0	1.0	1.0	1.0

PGRP-LE (FBgn0030695) associated phenotypes

heat sensitive, immune response defective, melanotic mass phenotype, short lived

(Information from FlyBase)

PGRP-LE (UniProt:Q9VXN9) annotation

Function: Peptidoglycan-recognition protein that plays a key role in innate immnunity by binding to murein peptidoglycans (PGN) of Gram-negative bacteria and activating the imd/Relish pathway. Has no activity against on Gram-positive bacteria. Binds to diaminopimelic acid-type PGN (DAP-type PGN), an activator of the imd/Relish pathway. Functions synergistically with PGRP-LC in producing resistance to E.coli and B.megaterium infections, which have the DAP-type peptidoglycan. Acts both upstream and in parallel with PGRP-LC in the imd/Relish pathway, and is required for infection-dependent activation of melanization. Required for Relish processing and nuclear translocation following proteolytic cleavage. Its localization suggests a role in the recognition and subsequent activation of the signaling at the first point of contact with invading bacteria. (PubMed:12359879, PubMed:15538387).
Subunit: Monomer. Peptidoglycan binding induces oligomerization. (PubMed:16428381).
Subcellular location: Secreted ECO:0000269—PubMed:15538387. Note=However, no signal sequence are predicted by sequence analysis tools.
Tissue specificity: Expressed in hemolymph. Localizes at the lumenal surface of the trachea (at protein level). (PubMed:15538387).
Developmental stage: Highly expressed in 0-5 hours embryos and in adult females, suggesting that it is expressed maternally. (PubMed:11106397).

(Information from UniProt)

Glycogen synthase kinase-3 beta

Best gene implication in ageing for this target family came from gene P38615 via mapping the annotation from Ensembl YMR139W via mapping the annotation from EntrezGene 855170 via mapping the annotation from GenAgeModels 1320 annotated in GenAge release 17.

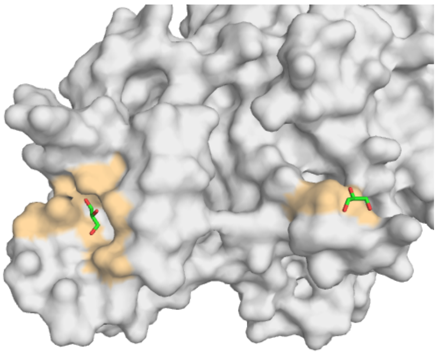

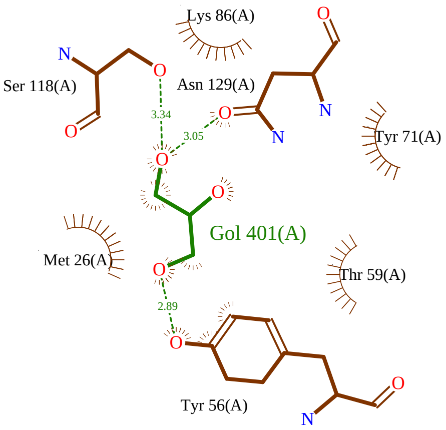

protein	amino acids contacts (binding site)								
PDB:4nu1:chainA:Q9WV60	M	Y	T	Y	K	S	N	R	S
tr:Q6FI27:Q6FI27_HUMAN	M	Y	T	Y	K	S	N	R	S
sp:P49841:GSK3B_HUMAN	M	Y	T	Y	K	S	N	R	S
sp:P18266:GSK3B_RAT	M	Y	T	Y	K	S	N	R	S
tr:Q5KU03:Q5KU03_MOUSE	M	Y	T	Y	K	S	N	R	S
sp:Q9WV60:GSK3B_MOUSE	M	Y	T	Y	K	S	N	R	S
tr:E9QAQ5:E9QAQ5_MOUSE	M	Y	T	Y	K	S	N	R	S
tr:B6IDN4:B6IDN4_DROME	G	Y	T	F	K	S	N	R	A
sp:P18431:SGG_DROME	G	Y	T	F	K	S	N	R	A
tr:A4V3W2:A4V3W2_DROME	G	Y	T	F	K	S	N	R	A
tr:A4V3W1:A4V3W1_DROME	G	Y	T	F	K	S	N	R	A
tr:Q0KHW6:Q0KHW6_DROME	A	Y	T	F	K	S	N	R	A
tr:E6PBX9:E6PBX9_DROME	F	Y	T	F	K	S	N	R	A
tr:H5V852:H5V852_DROME	F	Y	T	F	K	S	N	R	A
sp:Q9U2Q9:GSK3_CAEEL	L	Y	Q	F	K	S	N	R	S
sp:P38615:RIM11_YEAST	P	F	T	F	K	E	N	R	V
sp:P50873:MRK1_YEAST	R	Y	T	V	K	E	N	R	V

	whole protein		domain-based		contact-based	
protein	ident	simil	ident	simil	ident	simil
PDB:4nu1:chainA:Q9WV60	1.0	1.0	1.0	1.0	1.0	1.0
tr:Q6FI27:Q6FI27_HUMAN	0.99	1.0	1.0	1.0	1.0	1.0
sp:P49841:GSK3B_HUMAN	0.99	1.0	1.0	1.0	1.0	1.0
sp:P18266:GSK3B_RAT	1.0	1.0	1.0	1.0	1.0	1.0
tr:Q5KU03:Q5KU03_MOUSE	1.0	1.0	1.0	1.0	1.0	1.0
sp:Q9WV60:GSK3B_MOUSE	1.0	1.0	1.0	1.0	1.0	1.0
tr:E9QAQ5:E9QAQ5_MOUSE	0.97	0.97	1.0	1.0	1.0	1.0
tr:B6IDN4:B6IDN4_DROME	0.58	0.71	0.69	0.83	0.67	0.71
sp:P18431:SGG_DROME	0.61	0.74	0.76	0.92	0.67	0.71
tr:A4V3W2:A4V3W2_DROME	0.61	0.74	0.76	0.92	0.67	0.71
tr:A4V3W1:A4V3W1_DROME	0.54	0.66	0.76	0.92	0.67	0.71
tr:Q0KHW6:Q0KHW6_DROME	0.5	0.63	0.69	0.9	0.67	0.76
tr:E6PBX9:E6PBX9_DROME	0.19	0.26	0.62	0.79	0.67	0.83
tr:H5V852:H5V852_DROME	0.28	0.35	0.6	0.74	0.67	0.83
sp:Q9U2Q9:GSK3_CAEEL	0.59	0.72	0.6	0.77	0.67	0.91
sp:P38615:RIM11_YEAST	0.43	0.72	0.4	0.72	0.44	0.64
sp:P50873:MRK1_YEAST	0.32	0.58	0.31	0.62	0.56	0.59

sgg (FBgn0003371) associated phenotypes

cell non-autonomous, cell polarity defective, chemical sensitive, circadian rhythm defective, devel-

opmental rate defective, germline clone, heat sensitive, lethal - all die before end of P-stage, lethal - all die before end of pupal stage, maternal effect, meiotic cell cycle defective, mitotic cell cycle defective, neuroanatomy defective, neurophysiology defective, non-rescuable maternal effect, planar polarity defective, rescuable maternal effect, short lived, smell perception defective, somatic clone, some die during pharate adult stage, some die during pupal stage, some die during third instar larval stage

(Information from FlyBase)

sgg (UniProt:P18431) annotation

Function: Required for several developmental events such as syncytial blastoderm formation and embryonic segmentation. Is involved in transcriptional regulation. Required for arm phosphorylation. Wg signaling operates by inactivating the sgg repression of en autoactivation. Negatively controls the neuromuscular junction (NMJ) growth in presynaptic motoneurons. Plays a role in the regulation of microtubule dynamics and actin cytoskeleton during embryogenesis. Required for phosphorylation of sra in activated eggs. Essential for completion of meiosis, possibly by triggering calcineurin activation via sra phosphorylation. Phosphorylates microtubule-associated protein futsch in axons. (PubMed:15269269, PubMed:16570248, PubMed:16949836, PubMed:2113617, PubMed:2118107, PubMed:22421435, PubMed:7529201, PubMed:8467811).

Subunit: Interacts with cos. (PubMed:1335365,PubMed:15691767).

Subcellular location: Cytoplasm. Nucleus. Cytoplasm, cytoskeleton, microtubule organizing center, centrosome. Cytoplasm, cell cortex. Cell junction, synapse. Cell projection, axon. Note=In syncytial embryos, detected at the centrosomes throughout the cell cycle, and in the mitotic spindle and pseudocleavage furrows invaginating from the cell cortex during mitosis. Concentrated at the growing end of membranes during the cellularization process. After cellularization, localized to the centrosomes during mitosis and to the nucleus at the end of telophase. Enriched in the presynaptic side of the neuromuscular junction, with some signal detected also in axonal branches.

Tissue specificity: Expressed in ovaries and activated eggs (at protein level). Expression is over all the embryo at all stages, no local accumulation is observed. (PubMed:2118107, PubMed:22421435, PubMed:8467811).

Developmental stage: Isoform SGG46 is expressed at low levels in 12-24 hours embryos. Isoform Zygotic and isoform SGG39 are expressed in 12-24 hours embryos and present throughout the larval, pupal and adult stages (at protein level). Isoform Zygotic is expressed maternally and zygotically but reduced throughout later embryonic development. Expression persists throughout larval stages. (PubMed:2118107, PubMed:8467811).

Disruption phenotype: Mutants display an overdeveloped neuromuscular junction (NMJ), with the number of boutons greatly increased. (PubMed:15269269).

(Information from UniProt)

gsk-3 (WBGene00001746) associated phenotypes

axon morphology variant, larval lethal, lethal, locomotion variant, sterile

(Information from WormBase)

gsk-3 (UniProt:Q9U2Q9) annotation

Function: Phosphorylates oma-1, a regulator of the oocyte-to- embryo transition, enabling its degradation. Phosphorylates skn-1, preventing it from accumulating in nuclei and thus inhibiting phase II gene expression in the oxidative stress defense. Involved in mesendoderm specification and mitotic spindle orientation in EMS blastomeres. Thought to be a branch point in these processes as proteins downstream are not required. Negatively regulates Wnt signaling in vulval precursor cells and acts as a Wnt-independent repressor of med-1 and med-2 in the C lineage inhibiting mesoderm development. Required for normal lifespan and LiCl-induced lifespan extension. (PubMed:10444600, PubMed:11463373, PubMed:15572126, PubMed:16251270, PubMed:16289132, PubMed:16343905, PubMed:16930586, PubMed:17959600).

Subunit: Monomer (By similarity). Interacts with axl-1. (UniProtKB:Q9WV60, PubMed:17601533).
Disruption phenotype: Worms exhibit defects in endoderm specification and mitotic spindle alignment. Mutants show reduced degradation of oma-1 and have shortened lifespan. (PubMed:10444600).
(Information from UniProt)

Potassium/sodium hyperpolarization-activated cyclic nucleotide-gated channel 4

Best gene implication in ageing for this target family came from gene Q8IGX2 annotated in UniProt release 2014_02. Annotation GO 8340 (determination of adult lifespan) was Inferred from Mutant Phenotype

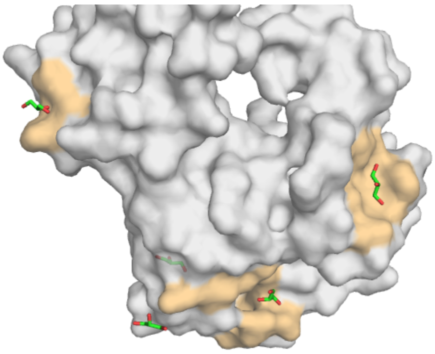

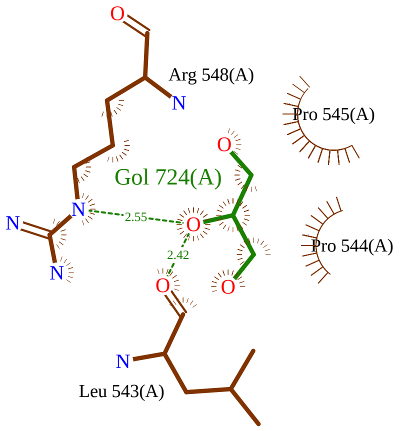

protein	amino acids contacts (binding site)
PDB:3u11:chainA:Q9Y3Q4	L P P R N V F A A P N V T E G K L S D R R
PDB:4kl1:chainA:Q9Y3Q4	L P P R N V F A A P N V T E G K L S D R R
sp:Q9Y3Q4:HCN4_HUMAN	L P P R N V F A A P N V T E G K L S D R R
sp:Q9JKA7:HCN4_RAT	L P P R N V F A A P N V T E G K L S D R R
tr:B2RY58:B2RY58_MOUSE	L P P R N V F A A P N V T E G K L S D R R
tr:Q8IGX2:Q8IGX2_DROME	L P R R N V F A A S N V S E G S L S E R K
tr:A1Z9P0:A1Z9P0_DROME	L P R R N V F A A S N V S E G S L S E R K
tr:Q56JH9:Q56JH9_DROME	L P R R N V F A A S N V S E G S L S E R K
tr:Q56JH8:Q56JH8_DROME	L P R R N V F A A S N V S E G S L S E R K
tr:F7VJU3:F7VJU3_DROME	L P R R N V F A A S N V S E G S L S E R K
tr:Q9Y1J9:Q9Y1J9_DROME	L P R R N V F A A S N V S E G S L S E R K
tr:Q56JI0:Q56JI0_DROME	L P R R N V F A A S N V S E G S L S E R K
tr:B7YZE7:B7YZE7_DROME	L P R R N V F A A S N V S E G S L S E R K
tr:A1Z9N8:A1Z9N8_DROME	L P R R N V F A A S N V S E G S L S E R K
tr:Q56JH7:Q56JH7_DROME	L P R R N V F A A S N V S E G S L S E R K
tr:B7YZE8:B7YZE8_DROME	L P R R N V F A A S N V S E G S L S E R K
tr:A1Z9N7:A1Z9N7_DROME	L P R R N V F A A S N V S E G S L S E R K

protein	whole protein ident	simil	domain-based ident	simil	contact-based ident	simil
PDB:3u11:chainA:Q9Y3Q4	0.99	1.0	0.99	1.0	1.0	1.0
PDB:4kl1:chainA:Q9Y3Q4	0.99	1.0	0.99	1.0	1.0	1.0
sp:Q9Y3Q4:HCN4_HUMAN	1.0	1.0	1.0	1.0	1.0	1.0
sp:Q9JKA7:HCN4_RAT	0.96	0.98	1.0	1.0	1.0	1.0
tr:B2RY58:B2RY58_MOUSE	0.95	0.98	1.0	1.0	1.0	1.0
tr:Q8IGX2:Q8IGX2_DROME	0.19	0.28	0.7	0.92	0.71	0.8
tr:A1Z9P0:A1Z9P0_DROME	0.28	0.42	0.7	0.92	0.71	0.8
tr:Q56JH9:Q56JH9_DROME	0.27	0.41	0.7	0.92	0.71	0.8
tr:Q56JH8:Q56JH8_DROME	0.27	0.41	0.7	0.92	0.71	0.8
tr:F7VJU3:F7VJU3_DROME	0.27	0.41	0.7	0.92	0.71	0.8
tr:Q9Y1J9:Q9Y1J9_DROME	0.26	0.47	0.7	0.92	0.71	0.8
tr:Q56JI0:Q56JI0_DROME	0.21	0.38	0.7	0.92	0.71	0.8
tr:B7YZE7:B7YZE7_DROME	0.21	0.38	0.7	0.92	0.71	0.8
tr:A1Z9N8:A1Z9N8_DROME	0.21	0.38	0.7	0.92	0.71	0.8
tr:Q56JH7:Q56JH7_DROME	0.21	0.37	0.7	0.92	0.71	0.8
tr:B7YZE8:B7YZE8_DROME	0.21	0.37	0.7	0.92	0.71	0.8
tr:A1Z9N7:A1Z9N7_DROME	0.21	0.37	0.7	0.92	0.71	0.8

Ih (FBgn0263397) associated phenotypes

conditional, hypoactive, neurophysiology defective, optomotor response defective, proboscis extension reflex defective, short lived, sleep defective, uncoordinated

(Information from FlyBase)

Collagen type IV alpha-3-binding protein

Best gene implication in ageing for this target family came from gene Q9Y128 via mapping the annotation from Ensembl FBgn0027569 via mapping the annotation from EntrezGene 38928 via mapping the annotation from GenAgeModels 0117 annotated in GenAge release 17.

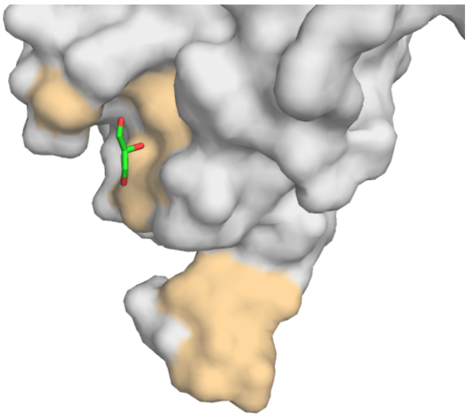

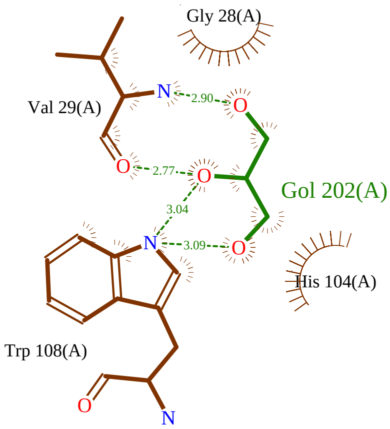

protein	amino acids contacts (binding site)
PDB:4hhv:chainA:Q9Y5P4	G V T E Y H W
sp:Q9Y5P4:C43BP_HUMAN	G V T E Y H W
tr:D4ACN6:D4ACN6_RAT	G V T E Y H W
sp:Q9EQG9:C43BP_MOUSE	G V T E Y H W
tr:Q9Y128:Q9Y128_DROME	G Y S D F D W
tr:Q93569:Q93569_CAEEL	G V K S H S W

protein	whole protein ident	simil	domain-based ident	simil	contact-based ident	simil
PDB:4hhv:chainA:Q9Y5P4	0.99	1.0	1.0	1.0	1.0	1.0
sp:Q9Y5P4:C43BP_HUMAN	1.0	1.0	1.0	1.0	1.0	1.0
tr:D4ACN6:D4ACN6_RAT	0.96	0.99	0.98	1.0	1.0	1.0
sp:Q9EQG9:C43BP_MOUSE	0.97	0.99	0.97	0.99	1.0	1.0
tr:Q9Y128:Q9Y128_DROME	0.42	0.74	0.47	0.79	0.29	0.7
tr:Q93569:Q93569_CAEEL	0.26	0.63	0.37	0.66	0.43	0.61

cert (FBgn0027569) associated phenotypes

heat stress response defective, locomotor behavior defective, short lived, stress response defective

(Information from FlyBase)
